# Synergistic Copper‐Aminocatalysis for Direct Tertiary α‐Alkylation of Ketones with Electron‐Deficient Alkanes

**DOI:** 10.1002/advs.202402255

**Published:** 2024-06-17

**Authors:** Qi‐Chao Shan, You‐Wei Wu, Mu‐Xiang Chen, Xuefei Zhao, Teck‐Peng Loh, Xu‐Hong Hu

**Affiliations:** ^1^ Institute of Advanced Synthesis School of Chemistry and Molecular Engineering Nanjing Tech University 30 South Puzhu Road Nanjing 211816 China; ^2^ College of Advanced Interdisciplinary Science and Technology Henan University of Technology 100 Lianhua Street Zhengzhou 450001 China; ^3^ Division of Chemistry and Biological Chemistry School of Chemistry Chemical Engineering and Biotechnology Nanyang Technological University 21 Nanyang Link Singapore 637371 Singapore

**Keywords:** C(sp^3^)─C(sp^3^) coupling, copper, enamine, ketones, tertiary alkylation

## Abstract

In this study, a novel approach for the tertiary α‐alkylation of ketones using alkanes with electron‐deficient C─H bonds is presented, employing a synergistic catalytic system combining inexpensive copper salts with aminocatalysis. This methodology addresses the limitations of traditional alkylation methods, such as the need for strong metallic bases, regioselectivity issues, and the risk of over alkylation, by providing a high reactivity and chemoselectivity without the necessity for pre‐functionalized substrates. The dual catalytic strategy enables the direct functionalization of C(sp^3^)─H bonds, demonstrating remarkable selectivity in the presence of conventional C(sp^3^)─H bonds that are adjacent to heteroatoms or *π* systems, which are typically susceptible to single‐electron transfer processes. The findings contribute to the advancement of alkylation techniques, offering a practical and efficient route for the construction of C(sp^3^)─C(sp^3^) bonds, and paving the way for further developments in the synthesis of complex organic molecules.

## Graphical abstract

A tertiary α‐alkylation of ketones with alkanes having electron‐deficient C(sp^3^)─H bonds is reported with the aid of triple catalytic systems of inexpensive transition metal, aminocatalysis, and Lewis acid. The oxidative coupling proceeds in an exclusive regioselectivity even in the presence of conventional C(sp^3^)−H bonds that are susceptive to undergo single electron oxidation, including those located at allylic and benzylic postions or adjacent to heteroatoms.

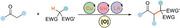

## 1 Introduction

The presence of a larger fraction of sp^3^‐hybridized carbons within molecular structures is beneficial in drug discovery, prompting synthetic chemists to focus on the complexity‐ and diversity‐oriented synthesis of sp^3^‐rich scaffolds.^[^ ^1^ ^]^ The construction of C(sp^3^)─C(sp^3^) bonds through modular assembly offers a direct route to increase the saturation of drug candidates. Notably, C(sp^3^)─C(sp^3^) cross‐coupling via metal catalysis, enhanced by the advent of new techniques (for selected reviews, see ref. [2]), is recognized as an efficient strategy to rapidly form such bonds. However, this often requires substrate pre‐functionalization. A significant challenge in these processes is the unwanted β‐hydride elimination, particularly limiting the use of bulky alkyl subunits.^[^ ^3^ ^]^

**Figure 1:**
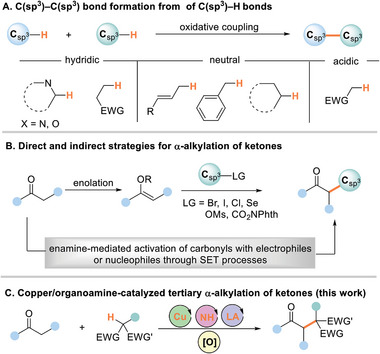
Oxidative alkylation of C(sp^3^)─H bonds through SET process.

The introduction of cross‐dehydrogenative coupling (CDC) by the Li laboratory presents an appealing approach for the formation of C(sp^3^)─C(sp^3^) bonds,^[^ ^4^ ^]^ emphasizing step‐ and atom‐economy. This strategy facilitates the abstraction of H‐atoms from C(sp^3^)─H bonds through hydrogen atom transfer (HAT) processes, primarily targeting hydridic and electron‐neutral C─H bonds. Site selectivity in this method is influenced by polar and enthalpy effects, especially favoring C(sp^3^)─H bonds adjacent to heteroatoms,^[^ ^5^ ^]^ as well as those in allylic^[^ ^6^ ^]^ and benzylic positions (**Figure** **1**A).^[^ ^7^ ^]^ In contrast, the oxidative functionalization of electron‐deficient C─H bonds with alkanes for dehydrogenative alkylation remains largely unexplored, despite significant progress in the hydroalkylation of alkenes.^[^ ^8^ ^]^ This gap highlights an area ripe for development, offering potential advancements in the synthesis of sp^3^‐rich compounds for pharmaceutical applications.

The α‐alkylation of ketones via reactive nucleophilic enolates represents a classic method for creating C(sp^3^)─C(sp^3^) bonds.^[^ ^9^ ^]^ Despite its widespread use, limitations such as the need for strong metallic bases, issues with regioselectivity, and the risk of overalkylation have restricted its practical applications. As an alternative, a radical methodology utilizing bench‐stable enols and their analogs has been explored to achieve high reactivity and chemoselectivity.^[^ ^10^ ^]^ Concurrently, enamine‐mediated activation of ketones with electrophiles,^[^ ^11^ ^]^ such as halides or Michael acceptors (illustrated by the Stork enamine reaction),^[^ ^12^ ^]^ has offered a reliable platform for the alkylation of carbonyl compounds (Figure 1B). Notably, catalytic strategies developed by the research groups of MacMillan^[^ ^13^ ^]^ and Melchiorre,^[^ ^14^ ^]^ which involve the single‐electron transfer (SET) oxidation of enamines derived from cyclic ketones and aldehydes, have also shown promise. Those methods typically depend on pre‐functionalized substrates as alkyl radical precursors, including halides, N‐(acyloxy)phthalimide derivatives, and selenides.

An elegant umpolung strategy that utilizes oxidative coupling of enamines with nucleophiles has enabled α‐functionalization of carbonyls through the formation of electrophilic iminium ion intermediates.^[^ ^15^ ^]^ The concept of oxidative enamine catalysis has introduced a novel reactivity mode for the direct functionalization of C(sp^3^)─H bonds,^[^ ^16^ ^]^ facilitating the alkylation of ketones. While SET oxidation of hydridic C(sp^3^)─H bonds for ketone alkylation is established,^[^ ^17^ ^]^ direct coupling with simple ketones and electron‐deficient C(sp^3^)─H bonds remains a challenge. Moreover, the incorporation of a tertiary alkyl group to carbonyl compounds has emerged as a particularly difficult objective in C(sp^3^)─C(sp^3^) cross‐coupling reactions.^[^ ^18^ ^]^ In the last decade, the synergy between transition metal and aminocatalysis has emerged as a potent strategy for ketone functionalization.^[^ ^19^ ^]^ Inspired by the significant progress in radical enamine chemistry, our laboratory envisioned that oxidative coupling of ketones with C(sp^3^)─H bonds could be achieved through a HAT process, facilitated by suitable metal‐organic cooperative catalytic systems.^[^ ^20^ ^]^

Recently, our team reported the oxidative alkylation of unsaturated systems with 1,3‐dicarbonyl compounds^[^ ^21^ ^]^ via the in situ generation of a dicarbonyl radical.^[^ ^22^ ^]^ Buoyed by the successful formation of C(sp^3^)─C(sp^2^) and C(sp^3^)─C(sp) bonds, we explored whether this oxidative coupling strategy could be adapted for the challenging formation of C(sp^3^)─C(sp^3^) bonds through direct ketone functionalization. We describe a general methodology for the tertiary α‐alkylation of ketones using alkanes with electron‐deficient C─H bonds, catalyzed by a combination of inexpensive copper salts and aminocatalysis (Figure 1C). This dual catalytic system circumvents the need for pre‐functionalized substrates as alkylating agents and achieves high selectivity, even in cases where conventional C(sp^3^)─H bonds adjacent to heteroatoms or π systems are typically prone to undergo SET processes. In addition, the cost‐effective copper catalysis in the absence of ligand allows oxidative coupling of a range of enol derivatives with tertiary C(sp^3^)─H bonds, further demonstrating the potential of this approach for ketone alkylation.

## 2 Results and Discussion

We commenced the study by evaluating the oxidative alkylation of acetophenone 1 with commercially available diethyl methylmalonate 2 as a radical precursor (as detailed in **Table** **1**; also see Tables S1–S10, Supporting Information). Initially, the alkylation product 3 could be obtained in the presence of CuCN (20 mol%), pyrrolidine A1 (50 mol%), FeCl_3_ (50 mol%), and DTBP in DMSO at 100 °C for 15 h. The presence of FeCl_3_ as Lewis acid is presumed to promote the formation of enamine (entry 1). It was subsequently determined that ZnCl_2_ exhibited superior reactivity among some common Lewis acids (entries 2–6). Switching other copper salts and solvents did not improve the isolated yield of 3 (entries 7 and 8). Next a systematic screening of bioisosteric amines was examined. Acyclic dibenzylamine A2 only gave an inferior result (entry 9). Therefore, we focused our investigation on diverse cyclic amines to develop a more effective catalytic platform. It was of interest to find that the ring size of aza‐heterocyclic scaffolds had a significant effect on the coupling efficiency. The employment of azetidine (A6) as a co‐catalyst led to a dramatic improvement in the yield of product 3 (entry 13). In contrast, six‐membered aza‐heterocyclic scaffolds, including piperidine (A3), piperazine (A4), and morpholine (A5), proved to be less effective (entries 10–12). In fact, the application of azetidines in nitrogen‐based enamine catalysis is relatively underexplored in the literatures. The four‐membered ring skeleton exhibits higher catalytic efficacy, probably due to its inherent greater strain and conformational rigidity, which results in a higher reaction rate with 1 and a reduced steric congestion of enamine intermediate, making it more accessible to be attacked by electrophiles.^[^ ^23^ ^]^ Encouraged by the high efficiency of azetidine, we explored the possibility of reducing the aminocatalyst loading. To our delight, a decrease to a 20 mol% loading of A6 led to 73% yield (entry 14). Further reducing the loading to 10 mol% was detrimental to the efficiency (entry 15). In addition, evaluation of other reaction parameters including temperature and time (entries 16–20), revealed an optimal result with 84% isolated yield of the cross‐coupling product 3 (entry 19). It was observed that the presence of Lewis acid had a substantial influence on the conversion (entry 21). Careful exclusion of either the aminocatalyst or the oxidant completely impeded the reaction (entries 22 and 23). In total, the optimized reaction conditions include a combination of CuCN (20 mol%), A6 (20 mol%), ZnCl_2_ (50 mol%), and DTBP in DMSO (0.1 m) at 80 °C for 12 h.

**Table 1:** Optimization of reaction conditions.

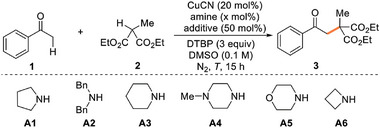					
Entry^a)^	Amine	x [mol%]	Additive	*T* [°C]	Yield [%]^b)^
1	**A1**	50	FeCl_3_	100	9
2	**A1**	50	AlCl_3_	100	29
3	**A1**	50	BF_3_•OEt	100	0
4	**A1**	50	Zn(OAc)_2_	100	31
5	**A1**	50	ZnBr_2_	100	28
6	**A1**	50	ZnCl_2_	100	43
7^c)^	**A1**	50	ZnCl_2_	100	12−23
8^d)^	**A1**	50	ZnCl_2_	100	7−33
9	**A2**	50	ZnCl_2_	100	16
10	**A3**	50	ZnCl_2_	100	14
11	**A4**	50	ZnCl_2_	100	5
12	**A5**	50	ZnCl_2_	100	11
13	**A6**	50	ZnCl_2_	100	69
14	**A6**	20	ZnCl_2_	100	73
15	**A6**	10	ZnCl_2_	100	61
16	**A6**	20	ZnCl_2_	90	74
17	**A6**	20	ZnCl_2_	80	81
18	**A6**	20	ZnCl_2_	70	53
19^e)^	**A6**	20	ZnCl_2_	80	84
20^f)^	**A6**	20	ZnCl_2_	80	67
21	**A6**	20	‐	80	68
22	‐	‐	ZnCl_2_	80	0
23^g)^	**A6**	20	ZnCl_2_	80	0

^a)^ Reaction conditions: **1** (0.15 mmol), **2** (2.0 equiv), CuCN (20 mol%), DTBP (3.0 equiv), amine, additive (50 mol%), DMSO (1.5 mL), N_2_, 15 h;

^b)^ Isolated yields are given;

^c)^ Other copper salts instead of CuCN;

^d)^ Other solvents instead of DMSO;

^e)^ 12 h;

^f)^ 8 h;

^g)^ Without CuCN or DTBP.

Subsequently, we investigated the scope and selectivity of tertiary functionalized alkanes bearing electron‐deficient C(sp^3^)─H bonds by reacting with the model substrate (1) (**Table** **2**). A diverse range of α‐substituted malonates readily participated in the oxidative coupling to afford the β‐keto malonates (4–13) in moderate to good yields (65–81%). Noteworthily, many desirable functional groups, such as alkyl, benzyl, ester, cycloalkyl, ether, alkenyl, and alkynyl groups, could be installed on the chain at α‐position of malonates. In particular, the functionalization of C(sp^3^)─H bonds proceeded with specific chemo‐ and site‐selectivity, even in the presence of conventional C─H bonds that are susceptible to HAT processes under oxidizing conditions. This is evidenced by maintaining the exclusive selectivity among other C─H bonds that are located at benzylic and allylic positions or adjacent to oxygen atom. It is believed that the high site‐selectivity, targeting electron‐deficient C─H bonds to generate tertiary carbon‐centered radicals stabilized by SOMO‐*π* delocalization and hyperconjugation, does not imply reactivity based on acidity, and is instead governed by the enthalpy and radical philicity.^[^ ^24^ ^]^ The diester moieties are not mandatory for the reactivity, as documented by the successful execution of other active methine substrates including α‐cyano ester (14), *β*‐keto ester (15), malononitrile (16), and 1,3‐dione (17). Moreover, a variety of substrates having biologically relevant (hetero)arenes including cyanobenzene (18), pyridine (19), thiophene (20), indole (21), carbazole (24), and adamantine (25) moieties all were competent coupling partners. Likewise, useful substituents such as fluorine (22) and acetal (23) were also tolerated. These examples together with complete regioselectivity highlighted the robustness of these dual catalytic systems in accessing complex *β*‐multifunctionalized ketones, which are often elusive through conventional nucleophilic substitution strategies with α‐halocarbonyl compounds. The use of diethyl malonates, however, did not result in the formation of the desired product, indicating that this oxidative coupling occurred exclusively at the methine site.^[^ ^25^ ^]^

**Table 2:** Substrate scope of tertiary alkyl radical precursors^a)^.

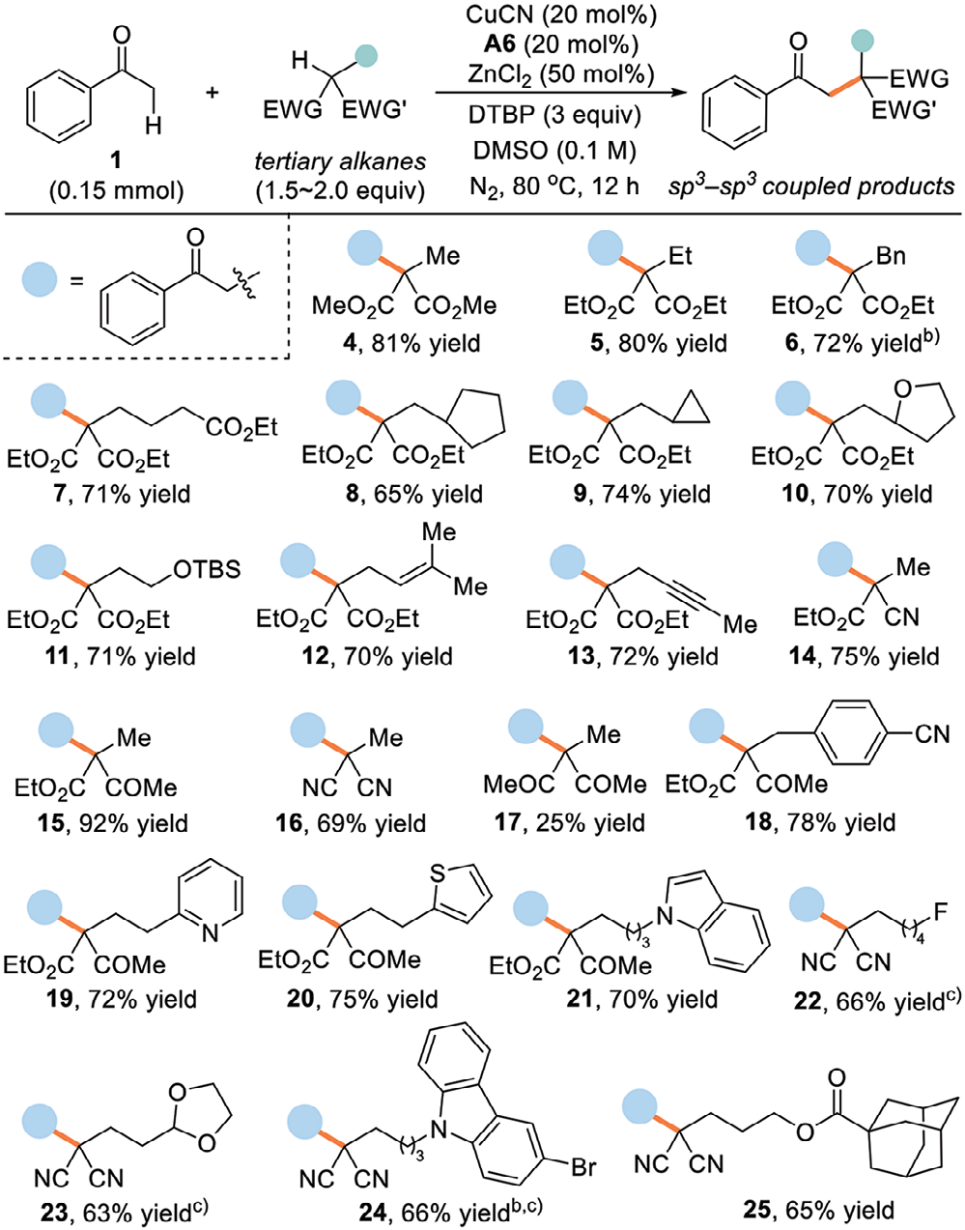

^a)^ Unless otherwise noted, reactions were performed with **1** (0.15 mmol), tertiary alkanes (1.5 – 2.0 equiv), CuCN (20 mol%), **A6** (20 mol%), ZnCl_2_ (50 mol%), DTBP (3.0 equiv), DMSO (1.5 mL), N_2_, 80 °C, 12 h;

^b)^ 90 °C;

^c)^ 18 h.

We then moved our attention to explore the generality and limitations of this cross‐coupling reaction with respect to ketones (**Table** **3**). Acetophenone derivatives featuring electronically varied substituents on the different positions of the phenyl ring were efficiently coupled with 2, giving a series of the dehydrogenated coupling products (26–45) in 50–89% yields. The introduction of alkyl, ether, amine, halogens, cyano, ester, boronate, amide, and acetal moieties on the aromatic ring was well tolerated, thus paving an opportunity for further synthetic elaborations. Moreover, we found that the methylketone scope could be extended to other aromatic systems, such as naphthalene (46), pyridine (47), thiophene (48), and quinoline (49), delivering the corresponding products in satisfactory yields. Of note, a good yield could be obtained for methyl ketones featuring a styryl motif (50). In addition, the reaction was applicable to other enolizable aryl acyclic and cyclic alkyl ketones, successfully yielding the α‐branched C(sp^3^)─C(sp^3^) coupled products (51–53) in synthetically useful yields. Then, we turned to examine the reactivity of more challenging dialkylketones. Delightfully, even unactivated methyl ketones were found to be compatible with the oxidative coupling process, delivering the alkylated products (54 and 55) at less hindered sites, albeit with somewhat reduced yields under slightly modified reaction conditions. Lastly, to showcase the general applicability of our protocol, we applied the reaction to a number of complex bioactive molecules, including l‐menthol (56), diacetone‐d‐glucose (57), nerol (58), celestolide (59), and several amino acid derivatives (60–62). These tertiary α‐alkylation reactions of ketones proceeded smoothly under the standard conditions, allowing for the installation of the multiple functionalities on the substrates without adversely affecting the reaction efficiency.

**Table 3:** Substrate scope with regard to ketones^a)^.

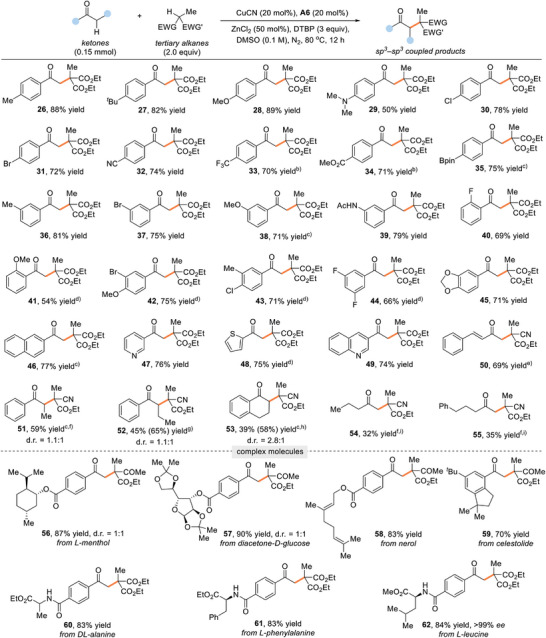

^a)^ Unless otherwise noted, reactions were performed with ketones (0.15 mmol), tertiary alkanes (2.0 equiv), CuCN (20 mol%), **A6** (20 mol%), ZnCl_2_ (50 mol%), DTBP (3.0 equiv), DMSO (1.5 mL), N_2_, 80 °C, 12 h. Isolated yields are reported. Yields in parentheses are based on revovered starting materials. The value of d.r. was determined by ^1^H NMR analysis;

^b)^ 75 °C;

^c)^ 90 °C;

^d)^ 100 °C;

^e)^ Using tertiary alkane (0.15 mmol) and ketone (3.0 equiv) for 24 h;

^f)^ With 40 mol% **A6** for 18 h;

^g)^ With 40 mol% **A6** and 7 equiv DTBP at 60 °C for 24 h;

^h)^ With 40 mol% **A6** for 24 h;

^i)^ Using tertiary alkane (0.15 mmol) and ketone (2.0 equiv) at 70 °C.

Based on the fruitful results of enamine catalysis for this newly developed radical alkylation of ketones, we envisioned an extended strategy where enamines or their equivalents could serve as radical acceptors to facilely implement the construction of C(sp^3^)─C(sp^3^) bonds with electron‐deficient C(sp^3^)─H bonds. More importantly, if successful, this approach would not only enrich the existing oxidative cross‐nucleophile coupling reactions, but also provide a new route to diverse dioxygenation frameworks that are otherwise inaccessible by direct functionalization of ketones. To validate this concept, a variety of enolate derivatives (1a−1e) were readily prepared from acetophenone and subjected to the oxidative conditions with the omission of an amine catalyst (**Table** **4**). Gratifyingly, the desired product 3 could be produced with enamines (1a and 1b), vinyl acetate (1c), and silyl enol ethers (1d−1e), where the use of 1d bearing a TBS group gave the best result. Inspired by these findings, we extended the investigation to silyl enol ethers derived from less reactive ketones, such as 1‐tetralone and cyclopentanone. Both examples gave promising results (53 and 63), contrasting with the previous dual catalytic systems by which the use of cyclopentanone as the substrate resulted in only a trace amount of the coupling product.^[^ ^24^ ^]^ Moreover, dienol ethers proved to be viable substrates for this coupling, furnishing γ‐substituted enones with a quaternary carbon center (64–67). These structures are extremely difficult to access by existing synthetic methods.^[^ ^26^ ^]^

**Table 4:** Exploration of oxidative coupling of enols or their equivalents with C(sp^3^)−H bonds^a)^.

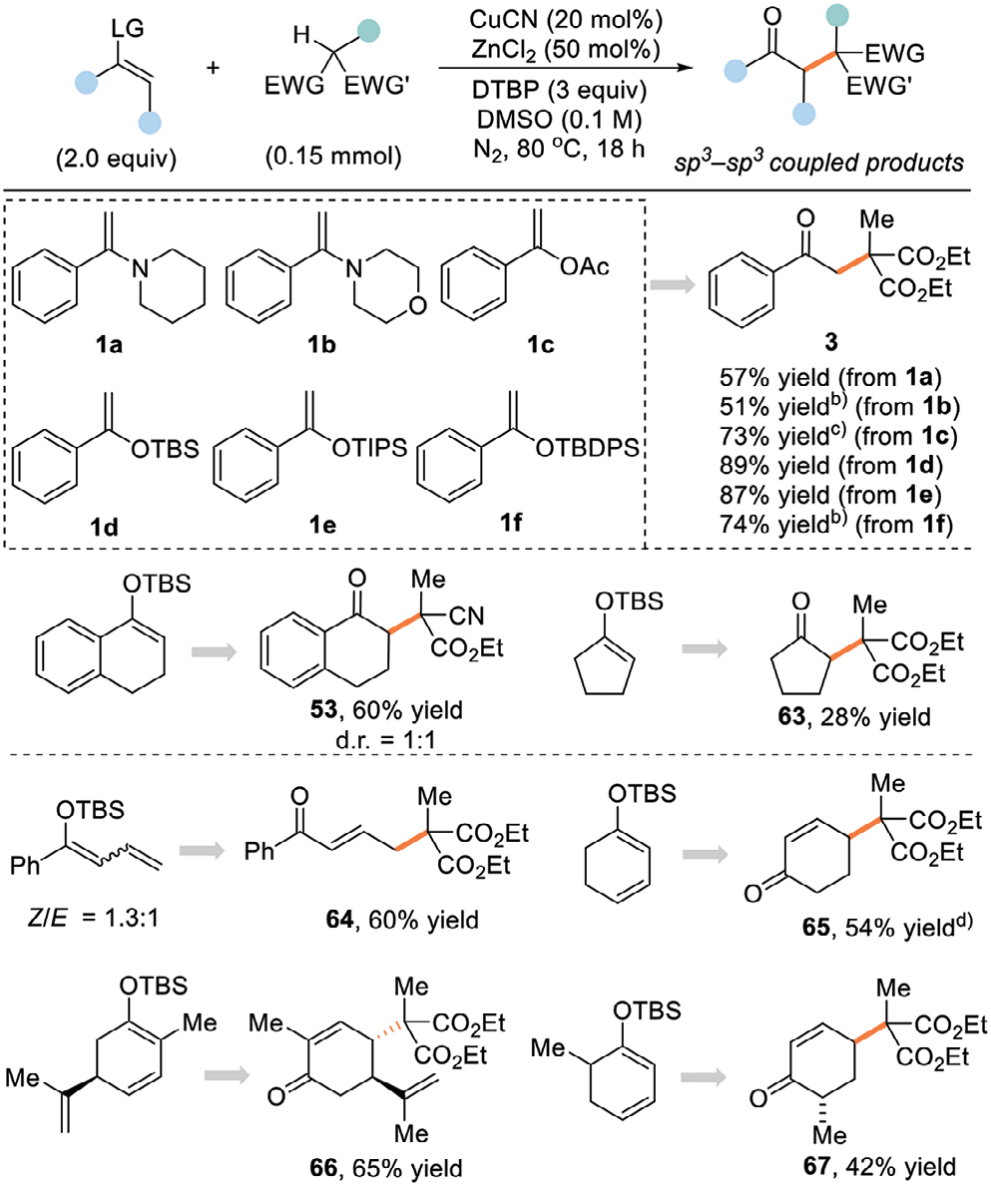

^a)^ Unless otherwise noted, reactions were performed with tertiary alkanes (0.15 mmol), alkenes (2.0 equiv), CuCN (20 mol%), ZnCl_2_ (50 mol%), DTBP (3.0 equiv), DMSO (1.5 mL), N_2_, 80 °C, 18 h;

^b)^ 24 h;

^c)^ 100 °C;

^d)^ Using 3.0 equiv enol.

**Scheme 1:**
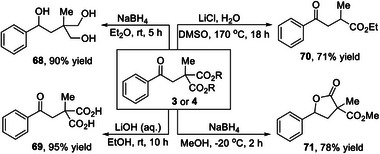
Post synthetic modifications.

To illustrate the synthetic utility of this developed protocol, a series of post‐synthetic transformations of the product were carried out (**Scheme** **1**). The diester moiety present in product 3 was readily modified through reduction and hydrolysis to afford valuable 1,3‐diol 68 and 1,3‐dicarboxylic acid 69, respectively. Selective decarboxylation took place in the presence of LiCl and water to yield γ‐keto ester 70. Interestingly, selective reduction of the product 4 using NaBH_4_ at low temperature gave γ‐lactone 71, which has great relevance to natural products and bioactive molecules.^[^ ^27^ ^]^

**Scheme 2:**
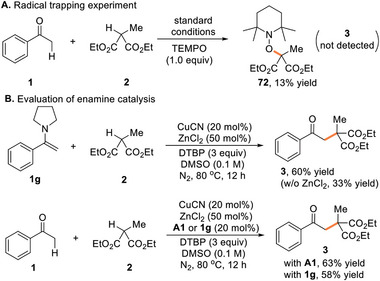
Mechanistic considerations.

Several preliminary mechanistic experiments were performed to elucidate the catalytic pathway. The addition of an equimolar amount of TEMPO (2,2,6,6‐tetramethyl‐1‐piperidi‐nyloxy), a renowned radical scavenger, completely inhibited the oxidative coupling (**Scheme** **2**A). Instead, the TEMPO‐methylmalonate radical adduct 72 was formed, implying the intermediacy of a tertiary carbon‐centered radical species in the process. Endeavors to synthesize an azetidine‐derived enamine met with failure. Instead, pyrrolidine‐derived enamine 1 g could be handled on the benchtop and was subjected to the standard conditions, giving rise to the formation of the desired product 3 in 60% yield (Scheme 2B). The omission of ZnCl_2_ resulted in the reduced yield (33%) of 3, which is consistent with our previous observation. The model reaction by employing pyrrolidine (A1) or enamine (1 g) as the co‐catalyst led to comparable yields of 3. These combined results revealed that the in situ generated enamine was the crucial intermediate to participate in the C(sp^3^)─C(sp^3^) bond forming step of the oxidative coupling.

**Figure 2:**
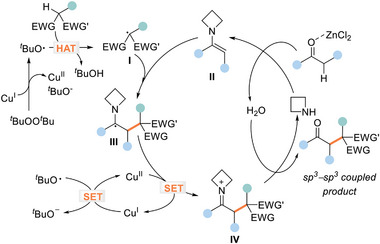
Plausible catalytic pathway.

On the basis of the above experimental observations and literature precedents,^[^ ^10^, ^22^ ^]^ a plausible mechanistic pathway is proposed in **Figure** **2**. Initially, Cu(I) is thought to catalyze the dissociation of DTBP for the generation of the *tert*‐butoxyl radical and the *tert*‐butoxyl anion.^[^ ^28^ ^]^ The highly potent *tert*‐butoxyl radical then participates in a HAT process with an alkane tethering an electron‐deficient C(sp^3^)─H bond to form a tertiary carbon‐centered radical species I. Of note, the coordination of Lewis acid with the 1,3‐diesters of malonates can form an enolate, which might facilitate the formation of the tertiary carbon‐centered radical species through a sequential deprotonation and SET event. We can not preclude the possibility of this alternative radical generation pathway at current state. Concurrently, Lewis acid‐mediated condensation between ketone and azetidine occurs to give enamine intermediate II. The addition of radical I to the transiently generated enamine leads to α‐amino radical III, which is then oxidized by Cu^II^ salt to afford iminium ion IV through a SET process. It can be rationalized that the presence of aromatic or vinyl substituents is beneficial to the stabilization of these intermediate species. Finally, the hydrolysis of the resulting intermediate IV furnishes the α‐alkylation ketone product along with the regeneration of the aminocatalyst to close the catalytic cycle.

## 3 Conclusion

In conclusion, we have developed a radical oxidative coupling of ketones with tertiary alkanes bearing electron‐deficient C(sp^3^)─H bonds by merging the concepts of transition metal, organocatalysis, and Lewis acid. Under the dual catalytic systems, this approach enables the direct forge of C(sp^3^)─C(sp^3^) through a CDC‐type process that circumvents the prerequisite for using pre‐functionalized substrates, giving rise to a diverse spectrum of tertiary alkylated ketones. The methodology has an excellent functional group compatibility and exhibits a broad substrate scope with respect to the functionalized alkanes and ketones. Furthermore, the oxidative functionalization proceeds with exclusive regiocontrol, completely eluding the direct oxidation of conventional reactive C(sp^3^)─H bonds. In addition, the protocol can be extended to various ketone‐derived nucleophiles, further ensuring broad applicability of subtrates. Mechanistic studies reveal the existence of a tertiary carbon‐centered radical species in the reaction. We anticipate versatile C(sp^3^)─C(sp^3^) construction with hydrocarbon feedstocks by environmentally benign and mild oxidative strategies and the development of their asymmetric variants in the future.

## Conflict of Interest

The authors declare no conflict of interest.

## Supporting information

Supporting Information

## Data Availability

The data that support the findings of this study are available from the corresponding author upon reasonable request.

## References

[1] a) F. Lovering , J. Bikker , C. Humblet , J. Med. Chem. 2009, 52, 6752; 19827778 10.1021/jm901241e

[2] a) J. Choi , G. C. Fu , Science 2017, 356, eaaf7230; 28408546 10.1126/science.aaf7230 PMC5611817

[3] a) L.‐J. Cheng , N. P. Mankad , Chem. Soc. Rev. 2020, 49, 8036; 32458840 10.1039/d0cs00316f

[4] a) S. A. Girard , T. Knauber , C.‐J. Li , Angew. Chem., Int. Ed. 2014, 53, 74; 10.1002/anie.201304268 24214829

[5] a) Z. Li , C.‐J. Li , J. Am. Chem. Soc. 2005, 127, 3672; 15771482 10.1021/ja050058j

[6] a) Z. Li , C.‐J. Li , J. Am. Chem. Soc. 2006, 128, 56; 16390119 10.1021/ja056541b

[7] a) Z. Li , L. Cao , C.‐J. Li , Angew. Chem., Int. Ed. 2007, 46, 6505; 10.1002/anie.200701782 17654644

[8] a) Z. Li , Y. Xiao , Z.‐Q. Liu , Chem. Commun. 2015, 51, 9969; 10.1039/c5cc02968f 25997410

[9] a) U. Kazmaier , Org. Chem. Front. 2016, 3, 1541;

[10] a) K. Miura , N. Fujisawa , H. Saito , D. Wang , A. Hosomi , Org. Lett. 2001, 3, 2591; 11483068 10.1021/ol016268s

[11] a) D. J. WeiX , J. F. Hartwig , J. Am. Chem. Soc. 2007, 129, 7720; 17542586 10.1021/ja071455s

[12] a) G. Stork , R. Terrell , J. Szmuszkovicz , J. Am. Chem. Soc. 1954, 76, 2029;

[13] D. A. Nicewicz , D. W. C. MacMillan , Science 2008, 322, 77. 18772399 10.1126/science.1161976 PMC2723798

[14] a) E. Arceo , I. D. Jurberg , A. Álvarez‐Fernández , P. Melchiorre , Nat. Chem. 2013, 5, 750; 23965676 10.1038/nchem.1727

[15] a) J.‐S. Tian , T.‐P. Loh , Angew. Chem., Int. Ed. 2010, 49, 8417; 10.1002/anie.201003646 20878822

[16] L. Zhu , D. Wang , Z. Jia , Q. Lin , M. Huang , S. Luo , ACS Catal. 2018, 8, 5466.

[17] a) A. Sud , D. Sureshkumar , M. Klussmann , Chem. Commun. 2009, 2009, 3169; 10.1039/b901282f 19587902

[18] a) C. C. Nawrat , C. R. Jamison , Y. Slutskyy , D. W. C. MacMillan , L. E. Overman , J. Am. Chem. Soc. 2015, 137, 11270; 26322524 10.1021/jacs.5b07678 PMC4632490

[19] a) I. Ibrahem , A. Córdova , Angew. Chem., Int. Ed. 2003, 45, 1952; 10.1002/anie.200504021 16521099

[20] a) A. Studer , D. P. Curran , Angew. Chem., Int. Ed. 2016, 55, 58; 10.1002/anie.201505090 26459814

[21] a) C. Wang , R.‐H. Liu , M.‐Q. Tian , X.‐H. Hu , T.‐P. Loh , Org. Lett. 2018, 20, 4032; 29943570 10.1021/acs.orglett.8b01600

[22] a) M. A. Dombroski , S. A. Kates , B. B. Snider , J. Am. Chem. Soc. 1990, 112, 2759;

[23] a) H. W. Thompson , J. Swistok , J. Org. Chem. 1981, 46, 4907;

[24] a) J. M. Tedder , Angew Chem. Int. Ed. Engl. 1982, 21, 401;

[25] For complete examples of currently unsuccessful substrates that we have attempted, see the Supporting Information.

[26] X. Chen , X. Liu , J. T. Mohr , J. Am. Chem. Soc. 2016, 138, 6364. 27159549 10.1021/jacs.6b02565

[27] a) M. Seitz , O. Reiser , Curr. Opin. Chem. Biol. 2005, 9, 285; 15939330 10.1016/j.cbpa.2005.03.005

[28] R. T. Gephart , C. L. McMullin , N. G. Sapiezynski , E. S. Jang , M. J. B. Aguila , T. R. Cundari , T. H. Warren , J. Am. Chem. Soc. 2012, 134, 17350. 23009158 10.1021/ja3053688

