# Supplementary material for: Synergistic Copper‐Aminocatalysis for Direct Tertiary α‐Alkylation of Ketones with Electron‐Deficient Alkanes
Source: Adv Sci (Weinh). 2024 Jun 17;11(31):2402255. doi: 10.1002/advs.202402255 (PMC11336924; doi:10.1002/advs.202402255)

ADVANCED SCIENCE
Open Access

Supporting Information

for *Adv. Sci.*, DOI 10.1002/advs.202402255

Synergistic Copper-Aminocatalysis for Direct Tertiary α-Alkylation of Ketones with Electron-Deficient Alkanes

Qi-Chao Shan, You-Wei Wu, Mu-Xiang Chen, Xuefei Zhao, Teck-Peng Loh and Xu-Hong Hu**

Synergistic Copper-Aminocatalysis for Direct Tertiary α-Alkylation of Ketones with Electron-Deficient Alkanes

Qi-Chao Shan[a,#], You-Wei Wu[a,#], Mu-Xiang Chen[a], Xuefei Zhao[a], Teck-Peng Loh[b,c,*], and Xu-Hong Hu[a,*]

[a] Institute of Advanced Synthesis, School of Chemistry and Molecular Engineering, Nanjing Tech University, Nanjing 211816, China

[b] College of Advanced Interdisciplinary Science and Technology, Henan University of Technology, 100 Lianhua Street, Zhengzhou 450001, China

[c] Division of Chemistry and Biological Chemistry, School of Chemistry, Chemical Engineering and Biotechnology, Nanyang Technological University, 21 Nanyang Link, Singapore 637371, Singapore

[#] These authors contributed equally to this work.

* Email: teckpeng@ntu.edu.sg (T.-P. Loh), ias_xhhu@njtech.edu.cn (X.-H. Hu)

Table of Contents

1	General methods	S2
2	Optimization of the reaction conditions	S3
3	Preparation of the starting materials	S7
	3.1 Synthesis of ketone substrates **1**–**1aal**	S7
	3.2 Synthesis of tertiary acidic C(sp^3)–H bond substrates **2**–**2aw**	S10
	3.3 Synthesis of enolate derivatives **1a**–**1l**	S14
4	General Procedure 7 for the tertiary α-alkylation of ketones	S14
5	General Procedure 8 for the tertiary α-alkylation of enolate derivatives	S15
6	Derivatization of the products **3** and **4**	S15
	6.1 Synthesis of **68**	S15
	6.2 Synthesis of **69**	S16
	6.3 Synthesis of **70**	S16
	6.4 Synthesis of **71**	S16
7	Mechanistic studies	S17
	7.1 Radical trapping experiment	S17
	7.2 Evaluation of enamine catalysis	S17
8	Characterization date of the products	S18
9	References	S36
10	NMR spectra of the starting materials	S37
11	NMR spectra of the products	S48

1. General methods

Experiments involving moisture and/or air sensitive components were performed in oven-dried glassware under a nitrogen atmosphere. Commercial solvents and reagents were purchased from Energy-Chemical, Aladdin Bio-Chem Technology and J&K Scientific, and were used without further purification.

Analytical thin layer chromatography (TLC) was performed using Merck 60 F254 precoated silica gel plate (0.2 mm thickness). Subsequent to elution, plates were visualized using UV radiation (254 nm) on Spectroline Model ENF-24061/F 254 nm. Further visualization was possible by staining with basic solution of potassium permanganate or acidic solution of ceric molybdate, followed by heating on a hot plate. Flash chromatography was performed using Nuotai silica gel (200 – 300 mesh) with distilled solvents. Columns were typically packed as slurry and equilibrated with petroleum ether (PE) prior to use.

Proton nuclear magnetic resonance (^{1}H NMR) and carbon nuclear magnetic resonance (^{13}C NMR) spectroscopy were performed on a Bruker Advance 400 MHz and JEOL 400 MHz spectrometers. Chemical shifts for ^{1}H NMR spectra are reported as in units of parts per million (ppm) downfield from $SiMe_4$ (δ 0.0) and relative to the signal of $CDCl_3$ (J = 7.264, singlet). Multiplicities were given as: s (singlet); d (doublet); t (triplet); q (quartet); dd (doublet of doublets); ddd (doublet of doublets of doublets); dddd (doublet of doublets of doublets of doublets); dt (doublet of triplets); m (multiplet), etc. The number of protons (n) for a given resonance is indicated by nH. Coupling constants are reported as a J value in Hz. Carbon nuclear magnetic resonance spectra (^{13}C NMR) are reported as δ in units of parts per million (ppm) downfield from $SiMe_4$ (δ = 0.0) and relative to the signal of $CDCl_3$ (δ = 77, triplet). To clarify the complete signal assignments, "× number" indicates the multiple carbons due to the superposition of chemical shifts.

High resolution mass spectral (HRMS) analysis was performed on Water Q-TOF Premier mass spectrometer (Thermo Electron Corporation). Enantiomeric excess (*ee*) was determined on an Agilent 1260 HPLC system and isopropanol-hexanes as the eluent.

2. Optimization of the reaction conditions

Table S1. Effect of Lewis acid on the reaction.[a]

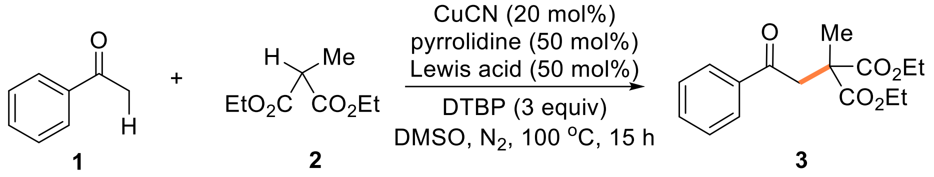

Entry	Lewis acid	Yield (%)[b]	Entry	Lewis acid	Yield (%)[b]
1	$BF_3 \cdot Et_2O$	trace	6	*p*TSA	0
2	$ZnCl_2$	43	7	$Zn(OAc)_2$	31
3	$Zn(OTf)_2$	22	8	$ZnBr_2$	28
4	$Fe(OTf)_2$	0	9	ZnF_2	29
5	$FeCl_3$	9	10	$AlCl_3$	7

[a] Reaction conditions: **1** (0.15 mmol, 1.0 equiv), **2** (0.3 mmol, 2.0 equiv), CuCN (0.03 mmol, 20 mol%), DTBP (0.45 mmol, 3.0 equiv), pyrrolidine (0.075 mmol, 50 mol%) and Lewis acid (0.075 mmol, 50 mol%) in DMSO (1.5 mL) at 100 °C for 15 h. [b] Yield of isolated product.

Table S2. Effect of copper salt on the reaction.[a]

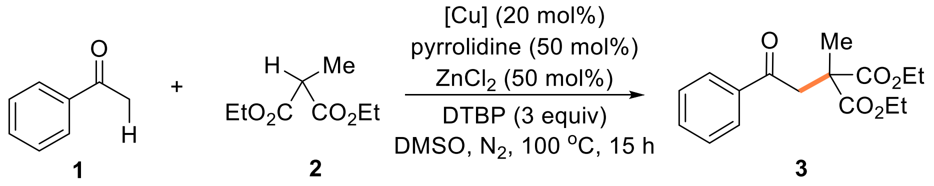

Entry	Copper salt	Yield (%)[b]	Entry	Copper salt	Yield (%)[b]
1	CuI	15	4	$CuCl_2$	16
2	CuCl	23	5	$CuBr_2$	12
3	CuCN	43	6	$Cu(OAc)_2$	22

[a] Reaction conditions: **1** (0.15 mmol, 1.0 equiv), **2** (0.3 mmol, 2.0 equiv), copper salt (0.03 mmol, 20 mol%), DTBP (0.45 mmol, 3.0 equiv), pyrrolidine (0.075 mmol, 50 mol%) and $ZnCl_2$ (0.075 mmol, 50 mol%) in DMSO (1.5 mL) at 100 °C for 15 h. [b] Yield of isolated product.

Table S3. Effect of solvent on the reaction.[a]

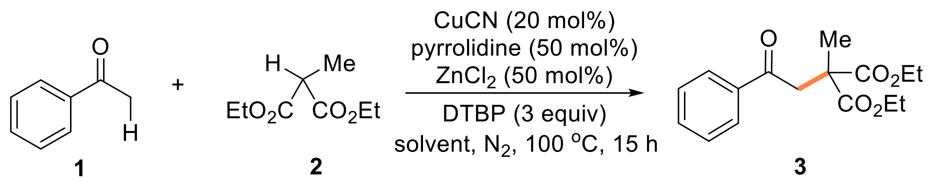

Entry	Solvent	Yield (%)[b]	Entry	Solvent	Yield (%)[b]
1	DMSO	43	5	MeCN	11
2	PhCl	19	6	DMF	29
3	THF	15	7	DMA	33

4	DCE	7			

[a] Reaction conditions: **1** (0.15 mmol, 1.0 equiv), **2** (0.3 mmol, 2.0 equiv), CuCN (0.03 mmol, 20 mol%), DTBP (0.45 mmol, 3.0 equiv), pyrrolidine (0.075 mmol, 50 mol%) and $ZnCl_2$ (0.075 mmol, 50 mol%) in Solvent (1.5 mL) at 100 °C for 15 h. [b] Yield of isolated product.

Table S4. Effect of aminocatalyst on the reaction.[a]

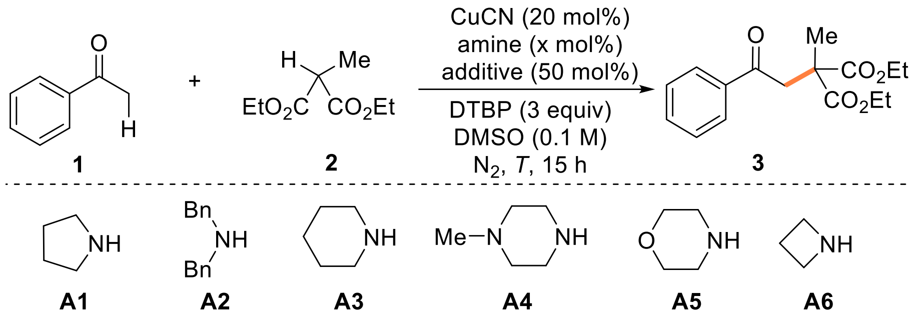

Entry	Amine	Yield (%)[b]	Entry	Amine	Yield (%)[b]
1	**A1**	43	4	**A4**	5
2	**A2**	16	5	**A5**	11
3	**A3**	14	6	**A6**	69

[a] Reaction conditions: **1** (0.15 mmol, 1.0 equiv), **2** (0.3 mmol, 2.0 equiv), CuCN (0.03 mmol, 20 mol%), DTBP (0.45 mmol, 3.0 equiv), amine catalyst (0.075 mmol, 50 mol%) and $ZnCl_2$ (0.075 mmol, 50 mol%) in DMSO (1.5 mL) at 100 °C for 15 h. [b] Yield of isolated product.

Table S5. Effect of amount of aminocatalyst on the reaction.[a]

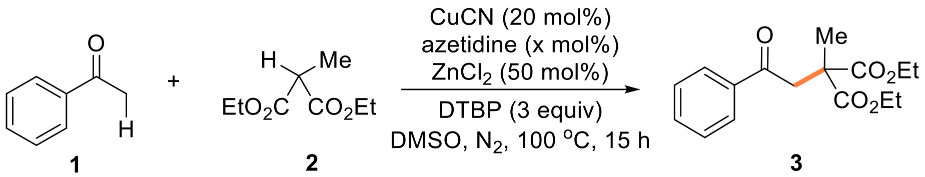

Entry	x (mol%)	Yield (%)[b]	Entry	x (mol%)	Yield (%)[b]
1	100	64	4	20	73
2	50	69	5	10	61
3	30	73			

[a] Reaction conditions: **1** (0.15 mmol, 1.0 equiv), **2** (0.3 mmol, 2.0 equiv), CuCN (0.03 mmol, 20 mol%), DTBP (0.45 mmol, 3.0 equiv), azetidine (X mol%) and $ZnCl_2$ (0.075mmol, 50 mol%) in DMSO (1.5 mL) at 100 °C for 15 h. [b] Yield of isolated product.

Table S6. Effect of temperature on the reaction.[a]

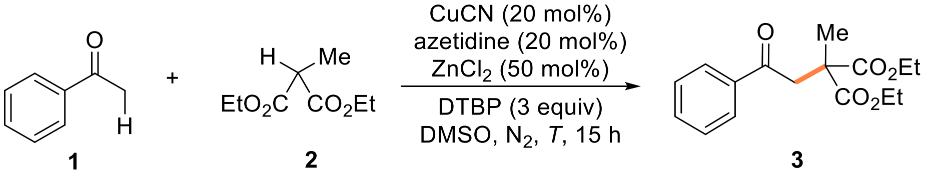

Entry	Temperature (°C)	Yield (%)[b]	Entry	Temperature (°C)	Yield (%)[b]
1	70	53	3	90	74

2	80	81	4	100	73

[a] Reaction conditions: **1** (0.15 mmol, 1.0 equiv), **2** (0.3 mmol, 2.0 equiv), CuCN (0.03mmol, 20 mol%), DTBP (0.45 mmol, 3.0 equiv), Azetidine (0.03 mmol, 20 mol%) and $ZnCl_2$ (0.075 mmol, 50 mol%) in DMSO (1.5 mL) at *T* (°C) for 15 h. [b] Yield of isolated product.

Table S7. Effect of time on the reaction.[a]

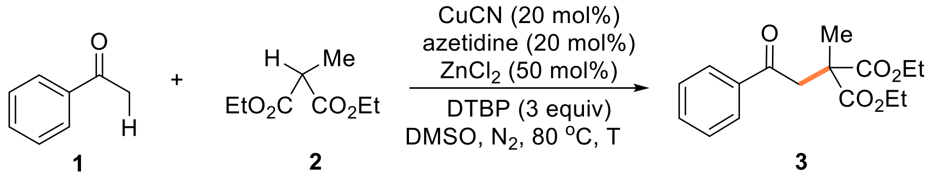

Entry	Time (h)	Yield (%)	Entry	Time (h)	Yield (%)[b]
1	8	67	3	15	81
2	12	84	4	24	83

[a] Reaction conditions: **1** (0.15 mmol), **2** (0.3 mmol), CuCN (20 mol%), DTBP (3.0 equiv), Azetidine (20 mol%) and $ZnCl_2$ (50 mol%) in DMSO (1.5 mL) at 80 °C for time. [b] Yield of isolated product.

Table S8. Effect of amount of Lewis acid on the reaction.[a]

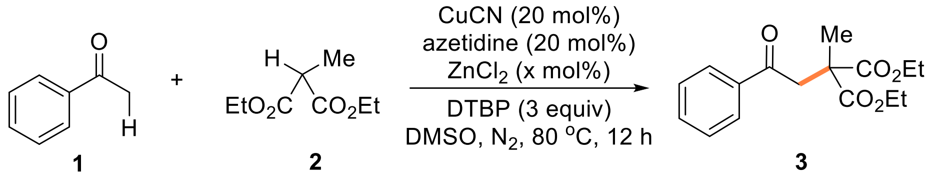

Entry	x (mol%)	Yield (%)	Entry	x (mol%)	Yield (%)[b]
1	50	84	3	10	68
2	30	74			

[a] Reaction conditions: **1** (0.15 mmol, 1.0 equiv), **2** (0.3 mmol, 2.0 equiv), CuCN (0.03 mmol, 20 mol%), DTBP (0.45 mmol, 3.0 equiv), Azetidine (0.03 mmol, 20 mol%) and $ZnCl_2$ (X mol%) in DMSO (1.5 mL) at 80 °C for 12 h. [b] Yield of isolated product.

Table S9. Effect of amount of copper catalyst on the reaction.[a]

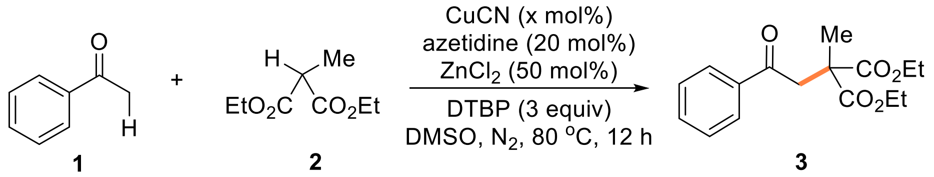

Entry	x (mol%)	Yield (%)	Entry	x (mol%)	Yield (%)[b]
1	20	84	2	13	62

[a] Reaction conditions: **1** (0.15 mmol, 1.0 equiv), **2** (0.3 mmol, 2.0 equiv), CuCN (x mmol%), DTBP (0.45 mmol, 3.0 equiv), azetidine (0.03 mmol, 20 mol%) and $ZnCl_2$ (0.075 mmol, 50 mol%) in DMSO (1.5 mL) at 80 °C for 12 h. [b] Yield of isolated product.

Table S10. Control experiments.[a]

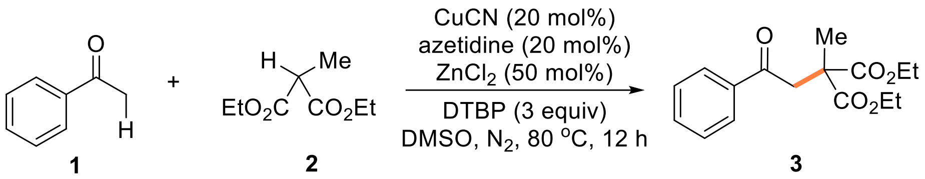

Entry	Change from the "standard conditions"	Yield (%)[b]
1	No change	84
2	w/o CuCN	0
3	w/o DTBP	0
4	w/o azetidine	trace
5	w/o $ZnCl_2$	68

[a] Reaction conditions: **1** (0.15 mmol, 1.0 equiv), **2** (0.3 mmol, 2.0 equiv), CuCN (0.03 mmol, 20 mol%), DTBP (0.45 mmol, 3.0 equiv), azetidine (0.03 mmol, 20 mol%) and $ZnCl_2$ (0.075 mmol, 50 mo%) in DMSO (1.5 mL) at 80 °C for 12 h. [b] Yield of isolated product.

3. Preparation of the starting materials

3.1 Synthesis of ketone substrates 1–1aal

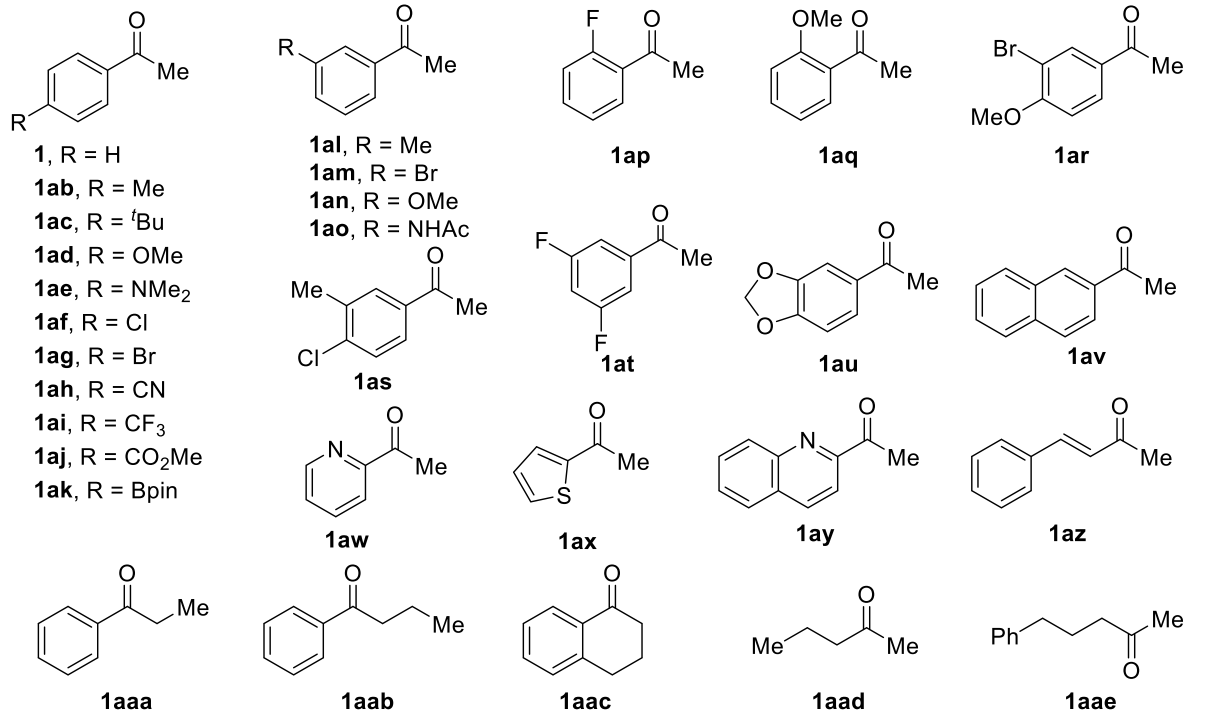

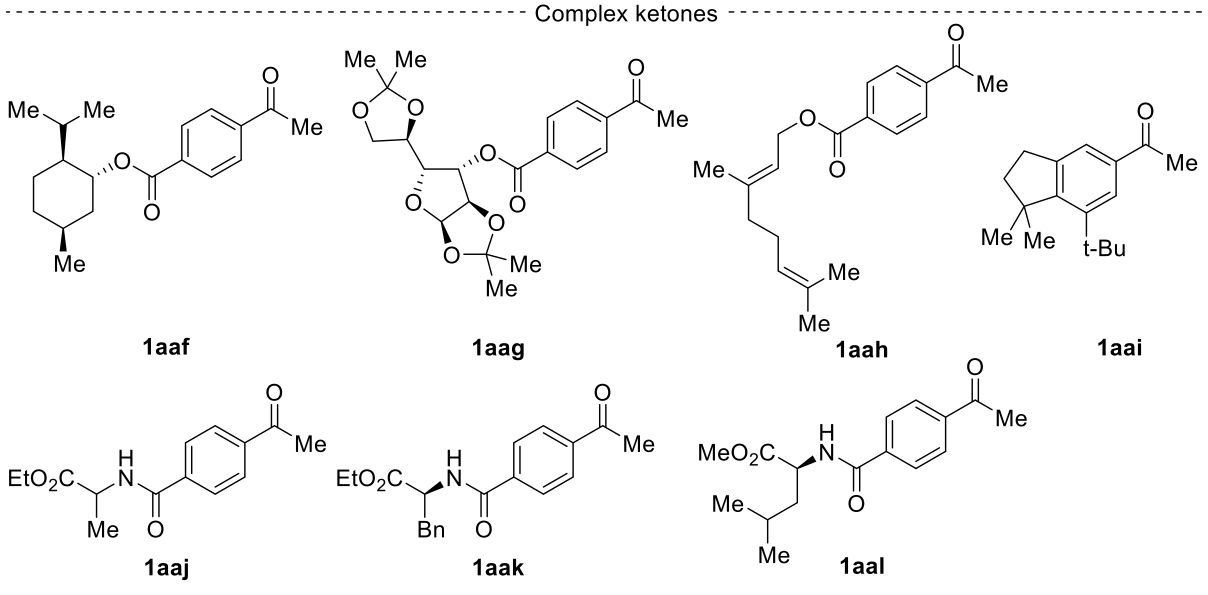

Substrates **1**–**1aad** and **1aai** are commercially available compounds. Substrate **1aae** is a known compound and was prepared according to the literature report.[1] Substrates **1aaf**–**1aah** were prepared according to the General Procedure **1**. Substrates **1aaj**–**1aal** were prepared according to General Procedure 2.

General Procedure 1 (GP1) for the synthesis of substrates 1aaf, 1aag and 1aah

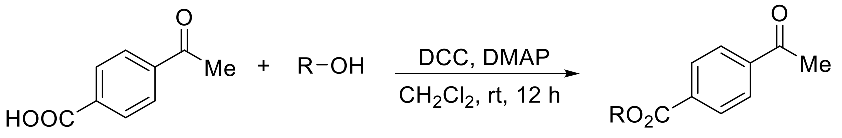

An over-dried 50 mL round bottom flask equipped with a stirring bar was charged with 4-acetylbenzoic acid (328.3 mg, 2 mmol, 1.0 equiv), alcohol (2 mmol, 1.0 equiv), N,N'-dicyclohexylcarbodiimide (DCC, 412.7 mg, 2 mmol, 1.0 equiv), 4-dimethylaminopyridine (DMAP, 244.3 mg, 2 mmol, 1.0 equiv) and CH_2Cl_2 (10 mL). The reaction was stirred at room temperature for overnight. After concentration, the resultant residue was purified by flash chromatography (PE/EtOAc) on silica gel to afford the corresponding ketones **1aaf**, **1aag** and **1aah**.

(1*R*,2*S*,5*S*)-2-Isopropyl-5-methylcyclohexyl 4-acetylbenzoate (1aaf)

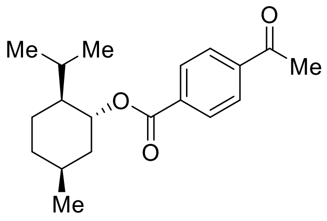

The title compound was prepared according to **GP1** and isolated as a colorless oil (459.7 mg, 1.52 mmol, 76%). ^{1}H NMR (400 MHz, $CDCl_3$) δ 8.08 – 8.02 (m, 2H), 7.97 – 7.87 (m, 2H), 4.88 (td, *J* = 10.9, 4.4 Hz, 1H), 2.56 (s, 3H), 2.09 – 2.00 (m, 1H), 1.87 (pd, *J* = 7.0, 2.7 Hz, 1H), 1.75 – 1.58 (m, 2H), 1.57 – 1.38 (m, 2H), 1.18 – 0.96 (m, 2H), 0.85 (dd, *J* = 6.8, 3.5 Hz, 7H), 0.72 (d, *J* = 7.0 Hz, 3H). ^{13}C NMR (101 MHz, $CDCl_3$) δ 197.1, 164.9, 139.8, 134.6, 129.5 × 2, 127.9× 2, 75.1, 47.0, 40.7, 34.0, 31.2, 26.6, 26.3, 23.4, 21.8, 20.5, 16.3. HRMS (ESI): m/z calculated for $C_{19}H_{27}O_3$ [M + H]$^+$: 303.1955, found: 303.1958.

(3a*R*,5*R*,6a*R*)-5-((*R*)-2,2-Dimethyl-1,3-dioxolan-4-yl)-2,2-dimethyltetrahydrofuro[2,3-*d*][1,3]dioxol-6-yl 4-acetyl benzoate (1aag)

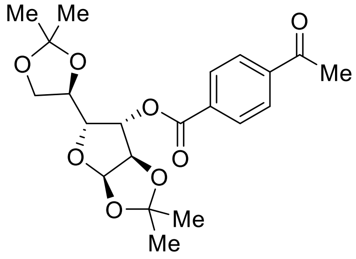

The title compound was prepared according to **GP1** and isolated as a colorless oil (658.3 mg, 1.62 mmol, 81%). ^{1}H NMR (400 MHz, $CDCl_3$) δ 8.10 (d, *J* = 8.4 Hz, 2H), 8.01 (d, *J* = 8.4 Hz, 2H), 5.96 (d, *J* = 3.7 Hz, 1H), 5.51 (d, *J* = 2.8 Hz, 1H), 4.64 (d, *J* = 3.7 Hz, 1H), 4.44 – 4.22 (m, 2H), 4.20 – 3.96 (m, 2H), 2.64 (s, 3H), 1.55 (s, 3H), 1.40 (s, 3H), 1.32 (s, 3H), 1.25 (s, 3H). ^{13}C NMR (101 MHz, $CDCl_3$) δ 197.2, 164.3, 140.5, 133.1, 129.9 × 2, 128.3 × 2, 112.3, 109.4, 105.1, 83.2, 79.8, 77.5, 72.5, 67.3, 26.8, 26.8, 26.6, 26.1, 25.1. HRMS (ESI): m/z calculated for $C_{21}H_{27}O_8$ [M + H]$^+$: 407.1700, found: 407.1702.

(*E*)-3,7-Dimethylocta-2,6-dien-1-yl 4-acetylbenzoate (1aah)

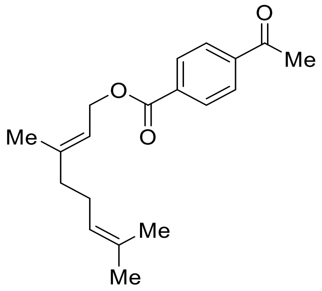

The title compound was prepared according to **GP1** and isolated as a colorless oil (438.5 mg, 1.46 mmol, 73%). ^{1}H NMR (400 MHz, $CDCl_3$) δ 8.16 – 8.11 (m, 2H), 8.03 – 7.94 (m, 2H), 5.49 (td, *J* = 7.2, 1.6 Hz, 1H), 5.17 – 5.01 (m, 1H), 4.84 (dd, *J* = 7.3, 1.0 Hz, 2H), 2.64 (s, 3H), 2.23 – 2.17 (m, 2H), 2.15 – 2.08 (m, 2H), 1.81 (d, *J* = 1.3 Hz, 3H), 1.67 (d, *J* = 1.3 Hz, 3H), 1.61 (d, *J* = 1.2 Hz, 3H). ^{13}C NMR (101 MHz, $CDCl_3$) δ 197.5, 165.7, 143.2, 140.0, 134.2, 132.2, 129.8 × 2, 128.1 × 2, 123.4, 118.8, 62.0, 32.2, 26.8, 26.6, 25.6, 23.5, 17.6. HRMS (ESI): m/z calculated for $C_{19}H_{25}O_3$ [M + H]$^+$: 301.1798, found: 301.1800.

General Procedure 2 (GP2) for the synthesis of substrates 1aaj, 1aak and 1aal

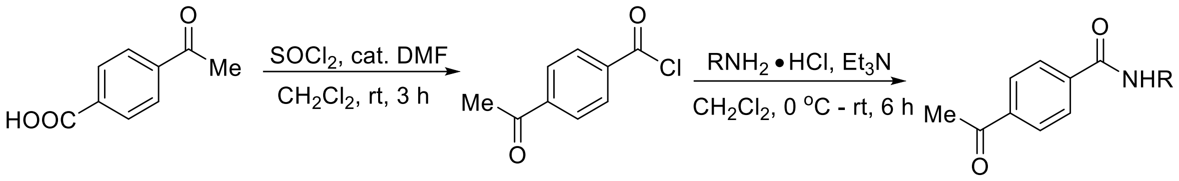

Step 1: An over-dried 50 mL round bottom flask equipped with a stirring bar was charged with 4-acetylbenzoic acid (328.3 mg, 2 mmol, 1.0 equiv) and CH_2Cl_2 (10 mL). Then sulfurous dichloride (285.5 mg, 2.4 mmol, 1.2 equiv) and DMF (7.3 mg, 0.1 mmol, 5 mol%) were added in the mixture in order. The reaction mixture was then stirred at room temperature for 3 h and monitored by TLC (PE/EtOAc). After the reaction was completed, the solvent was removed under reduced pressure to give 4-acetylbenzoyl chloride without further purification.

Step 2: An over-dried 50 mL round bottom flask equipped with a stirring bar was charged with amine (1.42 mmol, 1.0 equiv), Et_3N (172.0 mg, 1.70 mmol, 1.2 equiv) and CH_2Cl_2 (10 mL). Then, the obtained 4-acetylbenzoyl chloride was added dropwise at 0 °C. The resulting reaction mixture was allowed to warm to room temperature and stirred for 6 h. Saturated $NaHCO_3$ was then added, and the biphasic system was separated. The aqueous phase was extracted with CH_2Cl_2 (20 mL × 3) and the organic phases were combined and dried over anhydrous Na_2SO_4, filtered and concentrated under reduced pressure. The residue was purified by flash column chromatography on silica gel (PE/EtOAc) to afford the desired amide.

Ethyl (4-acetylbenzoyl)alaninate (1aaj)

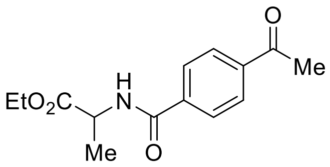

The title compound was prepared according to **GP2** and isolated as a colorless oil (273.8 mg, 1.04 mmol, 52% over two steps). ^1H NMR (400 MHz, $CDCl_3$) δ 8.07 – 7.97 (m, 2H), 7.90 – 7.84 (m, 2H), 6.83 (d, J = 7.1 Hz, 1H), 4.76 (p, J = 7.2 Hz, 1H), 4.24 (q, J = 7.1 Hz, 2H), 2.62 (s, 3H), 1.52 (d, J = 7.1 Hz, 3H), 1.30 (t, J = 7.1 Hz, 3H). ^{13}C NMR (101 MHz, $CDCl_3$) δ 197.4, 173.1, 165.8, 139.3, 137.8, 128.5 × 2, 127.4 × 2, 61.8, 48.7, 26.8, 18.6, 14.1. HRMS (ESI): m/z calculated for $C_{14}H_{18}NO_4$ [M + H]$^+$: 264.1230, found: 264.1235.

Ethyl (4-acetylbenzoyl)-*L*-phenylalaninate (1aak)

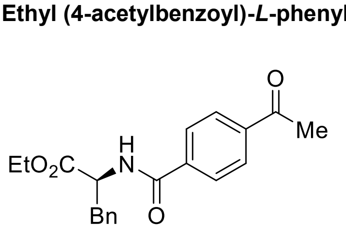

The title compound was prepared according to **GP2** and isolated as a colorless oil (325.7 mg, 0.96 mmol, 48% over two steps). ^1H NMR (400 MHz, $CDCl_3$) δ 7.98 (d, J = 8.3 Hz, 2H), 7.80 (d, J = 8.3 Hz, 2H), 7.33 – 7.24 (m, 2H), 7.18 – 7.06 (m, 2H), 6.73 (d, J = 7.6 Hz, 1H), 5.06 (q, J = 5.8 Hz, 1H), 4.23 (q, J = 7.1 Hz, 2H), 3.27 (qd, J = 13.8, 5.7 Hz, 2H), 2.62 (s, 3H), 1.29 (t, J = 7.2 Hz, 3H). ^{13}C NMR (101 MHz, $CDCl_3$) δ 197.4, 171.4, 165.8, 139.3, 137.7, 135.7, 129.3 × 2, 128.6 × 2, 128.5 × 2, 127.3 × 2, 127.2, 61.8, 53.6, 37.8, 26.8, 14.1. HRMS (ESI): m/z calculated for $C_{20}H_{22}NO_4$ [M + H]$^+$: 340.1543, found: 340.1545.

Methyl (4-acetylbenzoyl)-L-leucinate (1aal)

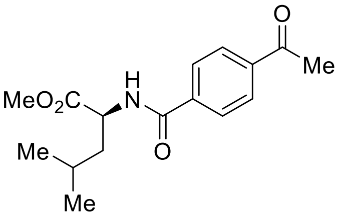

The title compound was prepared according to **GP2** and isolated as a colorless oil (224.5 mg, 0.77 mmol, 39% over two steps).^{1}H NMR (400 MHz, $CDCl_3$) *δ* 7.85 (dd, *J* = 8.6, 2.2 Hz, 2H), 7.81 – 7.76 (m, 2H), 7.11 (d, *J* = 8.2 Hz, 1H), 4.86 – 4.69 (m, 1H), 3.70 (s, 3H), 2.53 (s, 3H), 1.76 – 1.56 (m, 3H), 0.90 (dd, *J* = 6.0, 4.4 Hz, 6H). ^{13}C NMR (101 MHz, $CDCl_3$) *δ* 197.4, 173.6, 166.2, 139.0, 137.5, 128.2 × 2, 127.3 × 2, 52.3, 51.1, 41.2, 26.7, 24.8, 22.7, 21.7. HRMS (ESI): m/z calculated for $C_{16}H_{22}NO_4$ [M + H]$^+$: 292.1543, found: 292.1547.

3.2 Synthesis of tertiary acidic C(sp^3)–H bond substrates 2–2aw

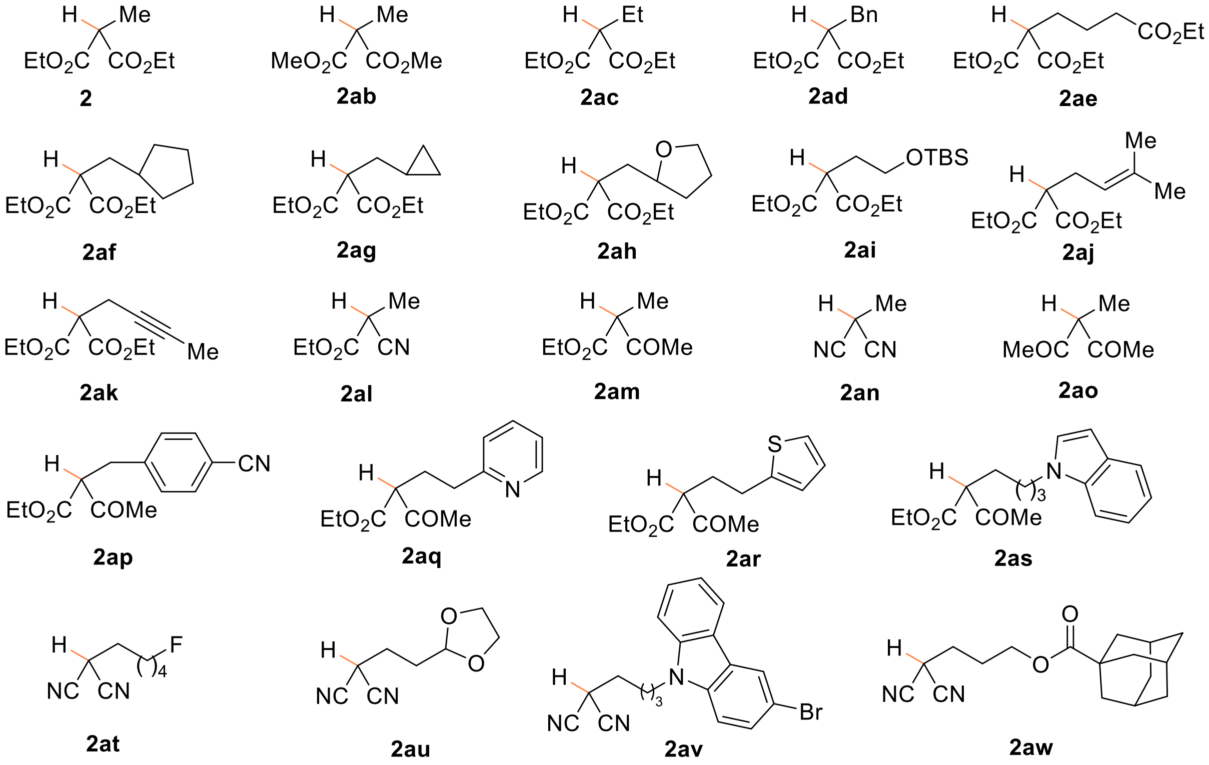

Substrates **2**–**2ad**, **2al**, **2am, 2ao** are commercially available compounds. Substrates **2ae**–**2aj**,[2] **2ak**[3], **2an,**[4] **2ap,**[5] **2at,**[4] **2au**[4] are known compounds and can be prepared according to the methods reported in the literatures. Substrates **2aq** and **2ar** were prepared according to the General Procedure **3**. Substrate **2as** was prepared according to the Typical Procedure **4**. Substrate **2av** was prepared according to the Typical Procedure **5**. Substrate **2aw** was prepared according to the Typical Procedure **6**.

General Procedure 3 (GP3) for the synthesis of substrates 2aq and 2ar

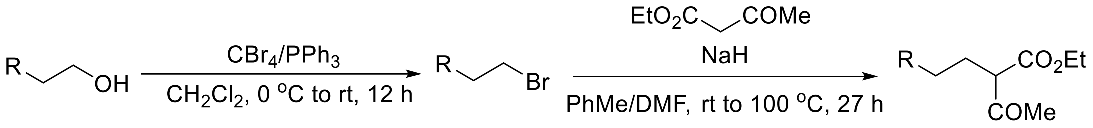

Step1: An over-dried 50 mL round bottom flask equipped with a stirring bar was charged with corresponding alcohol (5 mmol, 1.0 equiv) and CH_2Cl_2 (10 mL). Then, PPh_3 (1.44 g, 5.5 mmol, 1.1 equiv) and CBr_4 (1.82 g, 5.5 mmol, 1.1 equiv)

were added to the mixture at 0 °C. The reaction mixture was stirred at room temperature for overnight. The reaction was quenched by saturated NaCl (10 mL) and extracted with CH_2Cl_2 (20 mL × 3). The organic phases were combined, dried over anhydrous Na_2SO_4 and filtered. The filtrate was concentrated under reduced pressure to give the crude product and purified by flash column chromatography on silica gel (PE/EtOAc) to give the corresponding alkyl bromides.

Step 2: An over-dried 25 mL round bottom flask equipped with a stirring bar was charged with NaH (60 wt% in mineral oil, 96.0 mg, 2.4 mmol, 1.2 equiv) and toluene/DMF (0.6 mL / 0.5 mL) under N_2 atmosphere. The bottom flask was evacuated and backfilled with N_2 for 3 times. Then, was added. A solution of ethyl acetoacetate (260.3 mg, 2.0 mmol, 1.0 equiv) in toluene/DMF (0.6 mL / 0.5 mL) solution was slowly added dropwise to the reaction. The reaction mixture was stirred for 20 minutes at room temperature and alkyl bromide (2.4 mmol, 1.2 equiv) was added to the mixture which was stirred at 100 °C for 27 h. The reaction was quenched by saturated NH_4Cl (10 mL). The aqueous phase was extracted with Et_2O (10 mL × 3), washed with saturated NaCl, combined the organic phase, dried over anhydrous Na_2SO_4, filtered. The filtrate was concentrated under reduced pressure to give the crude product, which was purified by column chromatography on silica gel (PE/EtOAc) to give the desired products **2aq** and **2ar**.

Ethyl 2-acetyl-4-(pyridin-2-yl)butanoate (2aq)

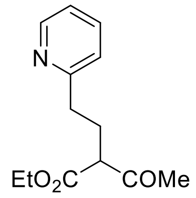

The title compound was prepared according to **GP3** and isolated as a colorless oil **2aq** (168.1 mg, 0.71 mmol, 36%). ^{1}H NMR (400 MHz, $CDCl_3$) δ 8.54 – 8.50 (m, 1H), 7.60 (td, J = 7.7, 1.8 Hz, 1H), 7.20 – 7.06 (m, 2H), 4.21 (qd, J = 7.2, 0.9 Hz, 2H), 3.50 (t, J = 7.3 Hz, 1H), 2.91 – 2.70 (m, 2H), 2.37 – 2.25 (m, 2H), 2.24 (s, 3H), 1.27 (t, J = 7.2 Hz, 3H). ^{13}C NMR (101 MHz, $CDCl_3$) δ 203.3, 169.7, 160.5, 149.3, 136.9, 123.3, 121.6, 61.6, 59.0, 35.6, 29.2, 27.9, 14.2. HRMS (ESI): m/z calculated for $C_{13}H_{18}NO_3$ [M + H]$^+$: 236.1281, found: 236.1282.

Ethyl 2-acetyl-4-(thiophen-2-yl)butanoate (2ar)

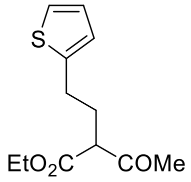

The title compound was prepared according to **GP3** and isolated as a colorless oil **2ar** (205.1 mg, 0.85 mmol, 43%). ^{1}H NMR (400 MHz, $CDCl_3$) δ 7.13 (dd, J = 5.2, 1.2 Hz, 1H), 6.94 – 6.89 (m, 1H), 6.79– 6.77 (m, 1H), 4.19 (q, J = 7.1 Hz, 2H), 3.47 (t, J = 7.1 Hz, 1H), 2.93 – 2.77 (m, 2H), 2.55 – 2.14 (m, 5H), 1.27 (t, J = 7.1 Hz, 3H). ^{13}C NMR (101 MHz, $CDCl_3$) δ 203.0, 169.6, 143.3, 127.0, 125.1, 123.7, 61.6, 58.5, 29.9, 29.3, 27.5, 14.2. HRMS (ESI): m/z calculated for $C_{12}H_{17}O_3S$ [M + H]$^+$: 241.0893, found: 241.0895.

Typical Procedure 4 for the synthesis of substrate 2as

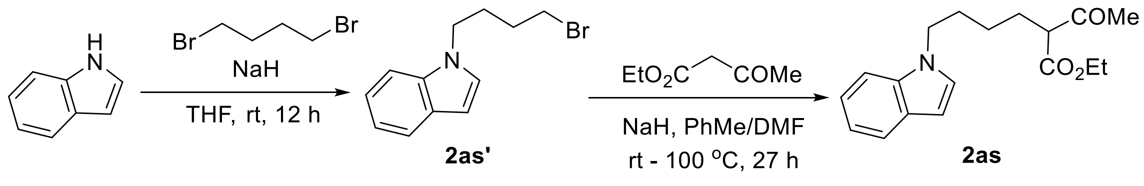

Step 1: An over-dried 50 mL round bottom flask equipped with a stirring bar was charged with NaH (60 wt% in mineral oil, 0.24 g, 6.0 mmol, 1.2 equiv) and THF (8 mL) under N_2 atmosphere. Then a THF solution (2 mL) of 1,4-dibromobutane (2.16 g, 10.0 mmol, 2.0 equiv) was added dropwise to the mixture. The reaction mixture was stirred for 20 min at room temperature and then indole (0.59 g, 5.0 mmol, 1.0 equiv) was added and stirred for 12 h at room temperature. Then, the reaction was quenched with saturated NH_4Cl (10 mL). The aqueous phase was extracted with EtOAc (10 mL × 3), washed with saturated NaCl. The organic phases were combined, dried over anhydrous Na_2SO_4, and filtered. The filtrate was concentrated under reduced pressure to give the crude product which was purified by column chromatography on silica gel (PE/EtOAc = 20:1) to give the corresponding bromide **2as'** (0.61 g, 2.4 mmol, 48%).

Step 2: An over-dried 25 mL round bottom flask equipped with a stirring bar was charged with NaH (60 wt% in mineral oil, 96.0 mg, 2.4 mmol, 1.2 equiv) and toluene/DMF (0.6 mL / 0.5 mL) under N_2 atmosphere. Subsequently, ethyl acetoacetate (260.3 mg, 2.0 mmol, 1.0 equiv) in toluene/DMF (0.6 mL / 0.5 mL) solution was added dropwise. The reaction mixture was stirred at room temperature for 20 min and **2as'** (0.68 g, 2.4 mmol, 1.2 equiv) was added and the reaction was stirred at 100 °C for 27 h. The reaction was quenched by saturated NH_4Cl (10 mL). The aqueous phase was extracted with EtOAc (10 mL × 3). The combined organic phases were washed with saturated NaCl, dried over anhydrous Na_2SO_4, and filtered. The filtrate was concentrated under reduced pressure and purified by flash column chromatography on silica gel (PE/EtOAc = 4:1) to give the desired product **2as** (0.42 g, 1.4 mmol, 70%). ^1H NMR (400 MHz, $CDCl_3$) δ 7.71 – 7.56 (m, 1H), 7.40 – 7.30 (m, 1H), 7.25 – 7.18 (m, 1H), 7.13 – 7.07 (m, 2H), 6.49 (dd, J = 3.2, 0.9 Hz, 1H), 4.24 – 4.04 (m, 4H), 3.50 (t, J = 7.3 Hz, 1H), 2.20 (s, 3H), 1.93 – 1.76 (m, 4H), 1.39 – 1.17 (m, 5H). ^{13}C NMR (101 MHz, $CDCl_3$) δ 203.1, 169.8, 135.9, 128.6, 127.9, 121.5, 121.1, 119.3, 109.4, 101.1, 61.5, 59.6, 46.0, 30.0, 29.1, 27.7, 24.9, 14.2. HRMS (ESI): m/z calculated for $C_{18}H_{24}NO_3$ $[M + H]^+$: 302.1751, found: 302.1754.

Typical Procedure 5 for the synthesis of substrate 2av

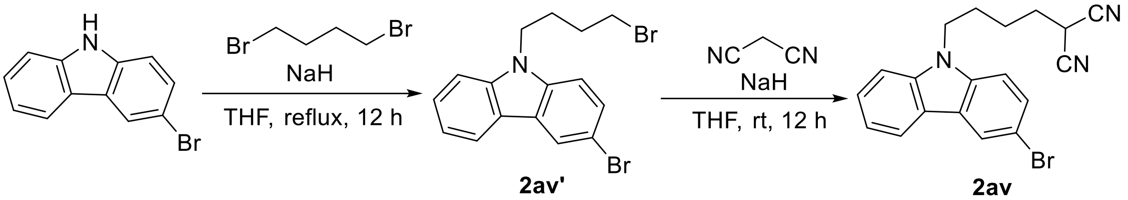

2av' 2av

Step 1: An over-dried 50 mL round bottom flask equipped with a stirring bar was charged with NaH (60 wt% in mineral oil, 0.50 g, 12.5 mmol, 2.5 equiv) and THF (8 mL) under N_2 atmosphere. A solution of 1,4-dibromobutane (1.60 g, 7.5 mmol, 1.5 equiv) in THF (2 mL) was added dropwise. The reaction mixture was stirred at room temperature for 20 min. Then 3-bromocarbazole (1.20 g, 5 mmol, 1.0 equiv) was added and the reaction was refluxed for overnight. The reaction was quenched by saturated NH_4Cl (10 mL). The aqueous phase was extracted with EtOAc (10 mL x 3). The combined organic phase were washed with saturated NaCl, dried over anhydrous Na_2SO_4 and filtered. The filtrate was concentrated under reduced pressure to give the crude product, which was purified by flash column chromatography on silica gel (PE/EtOAc = 20:1) to give the corresponding bromide **2av'** (1.03 g, 2.7 mmol, 54%).

Step 2: An over-dried 25 mL round bottom flask equipped with a stirring bar was charged with NaH (60 wt% in mineral

oil, 0.11 g, 2.8 mmol, 1.05 equiv) and THF (4 mL) under N_2 atmosphere. The reaction solution was cooled to 0 °C. A solution of malononitrile (0.18 g, 2.7 mmol, 1.0 equiv) in THF (1 mL) was added dropwise. The reaction mixture was stirred at 0 °C for 20 min. **2av'** (1.03 g, 2.7 mmol, 1.0 equiv) was added to the reaction system and stirred for 12 h at room temperature. After the completion (monitored by TLC), the reaction was quenched by saturated NH_4Cl (10 mL). The aqueous phase was extracted with EtOAc (10 mL × 3). The combined organic phases were washed with saturated NaCl, dried over anhydrous Na_2SO_4, and filtered. The filtrate was concentrated under reduced pressure to give the crude product which was purified by flash column chromatography on silica gel (PE/EtOAc = 3:1) to give the desired product **2av** (0.66 g, 1.8 mmol, 67%). ^1H NMR (400 MHz, $CDCl_3$) *δ* 8.19 (d, *J* = 1.9 Hz, 1H), 8.07 – 7.96 (m, 1H), 7.58 – 7.44 (m, 2H), 7.35 (d, *J* = 8.2 Hz, 1H), 7.27 – 7.19 (m, 2H), 4.29 (t, *J* = 7.0 Hz, 2H), 3.56 (t, *J* = 6.8 Hz, 1H), 2.03 – 1.83 (m, 4H), 1.73 – 1.56 (m, 2H). ^{13}C NMR (101 MHz, $CDCl_3$) *δ* 140.4, 138.7, 128.4, 126.6, 124.6, 123.2, 121.8, 120.7, 119.6, 112.2 × 2, 111.9, 109.8, 108.6, 42.4, 30.4, 27.8, 24.3, 22.4. HRMS (ESI): m/z calculated for $C_{19}H_{17}BrN_3$ $[M + H]^+$: 366.0600, found: 366.0598.

Typical Procedure 6 for the synthesis of substrate 2aw

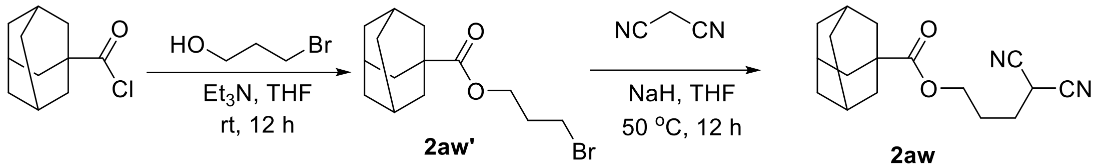

Step 1: An over-dried 25 mL round bottom flask equipped with a stirring bar was charged with 2-bromoethanol (0.55 g, 4.4 mmol, 1.1 equiv), triethylamine (0.61 g, 6.0 mmol, 1.5 equiv) and THF (10 mL). Then, adamantane chloride (0.79 g, 4.0 mmol, 1.0 equiv) was added dropwise. The reaction was stirred at room temperature for overnight. The reaction solution was concentrated under reduced pressure and the crude product was purified by flash column chromatography on silica gel (PE/EtOAc = 100:3) to give the ester **2aw'** (0.70 g, 2.3 mmol, 58%).

Step 2: An over-dried 25 mL round bottom flask equipped with a stirring bar was charged with NaH (60 wt% in mineral oil, 102.4 mg, 2.6 mmol, 1.05 equiv) and THF (4 mL) under N_2 atmosphere. The mixture was cooled to 0 °C. A solution of malononitrile (161.2 mg, 2.4 mmol, 1.0 equiv) in THF (1 mL) was added dropwise at 0 °C and stirred for 20 min. Then, alkyl bromides **2aw'** (0.70 g, 2.4 mmol, 1.0 equiv) was added to the mixture and stirred at 50 °C for 12 h. After the completion (monitored by TLC), the reaction was quenched with saturated NH_4Cl (10 mL) and extracted with EtOAc (10 mL × 3). The combined organic phases were washed with saturated NaCl, dried over anhydrous Na_2SO_4, and filtered. The filtrate was concentrated under reduced pressure to give the crude product which was purified by flash column chromatography on silica gel (PE/EtOAc = 6:1) to give **2aw** (359.5 mg, 1.3 mmol, 54%). ^1H NMR (400 MHz, $CDCl_3$) *δ* 4.14 (t, *J* = 5.8 Hz, 2H), 3.88 (t, *J* = 6.9 Hz, 1H), 2.17 – 2.09 (m, 2H), 2.07 – 1.94 (m, 5H), 1.90 – 1.82 (m, 6H), 1.78 – 1.65 (m, 6H). ^{13}C NMR (101 MHz, $CDCl_3$) δ 177.7, 112.5 × 2, 62.0, 40.9, 38.9 × 2, 38.7, 36.5 × 3, 28.1, 27.90 × 2, 27.88, 25.9, 22.4. HRMS (ESI): m/z calculated for $C_{17}H_{23}N_2O_2$ $[M + H]^+$: 287.1754, found: 287.1757.

3.3 Synthesis of enolate derivatives 1a–1m

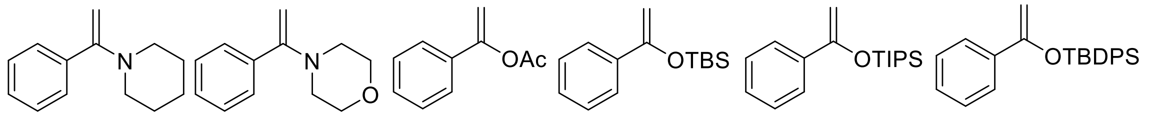

1a **1b** **1c** **1d** **1e** **1f**

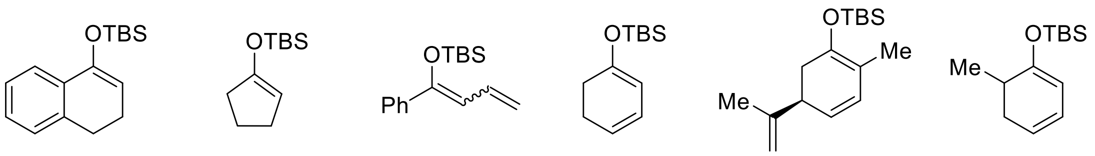

1h **1i** **1j** **1k** **1l** **1m**

Substrates **1a**,[6] **1b**,[6] **1c**,[7] **1d**,[6] **1e**,[6] **1f**,[6] ,**1h**,[8] **1i**,[9] **1j**,[9] **1k**,[10] **1l**,[10], **1m**[10] are known compounds and were prepared according to the existing methods reported in the literatures.

4. General Procedure 7 for the tertiary α-alkylation of ketones

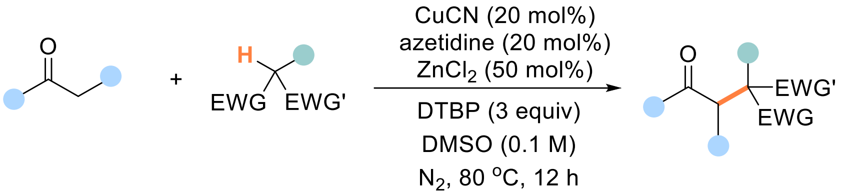

An oven-dried 10 mL Schlenk tube equipped with a stir bar was charged with CuCN (2.7 mg, 0.03 mmol, 20 mol%) and $ZnCl_2$ (10.2 mg, 0.075 mmol, 50 mol%). The tube was evacuated and backfilled with N_2 for 3 times. Azetidine (1.7 mg, 0.03 mmol, 20 mol%), DTBP (65.8 mg, 0.45 mmol, 3.0 equiv), ketone (0.15 mmol, 1.0 equiv), tertiary alkane (0.3 mmol, 1.5 equiv) and DMSO (1.5 mL) were successively added via syringe under N_2 atmosphere. The sealed tube was placed into a preheated oil bath at 80 °C with stirring for 12 h. After cooling to room temperature, the mixture was diluted with water (10 mL) and the aqueous layer was extracted with CH_2Cl_2 (10 mL × 3). The combined organic layers were rinsed with saturated NaCl (10 mL), dried over anhydrous Na_2SO_4, filtered and the filtrate was concentrated under reduced pressure. The resultant residue was purified by flash chromatography on silica gel (PE/EtOAc) to afford the sp^3-sp^3 coupling product.

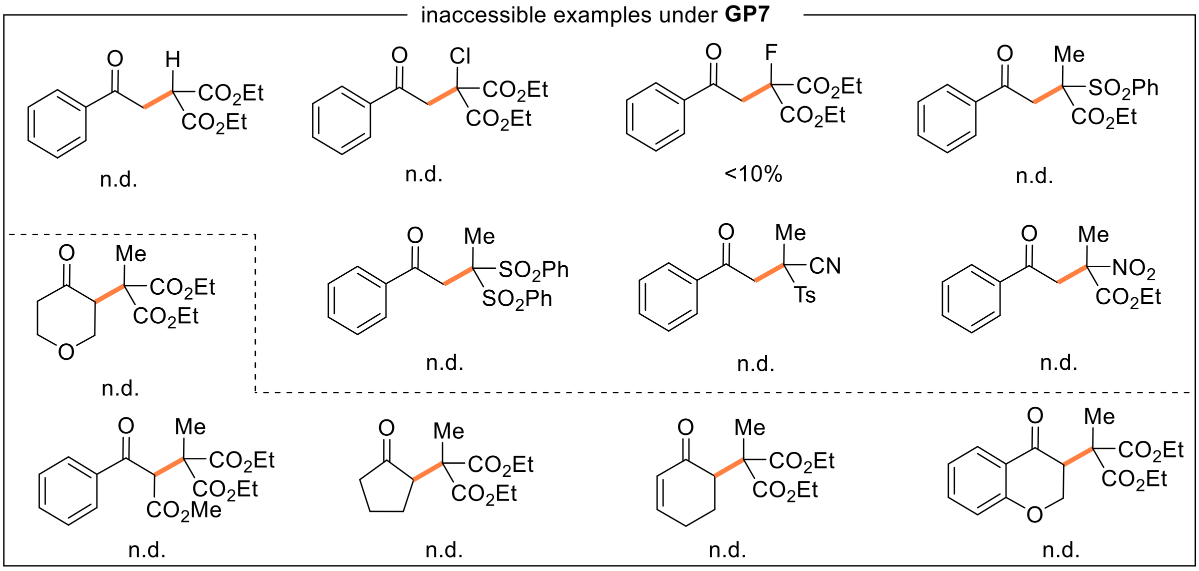

5. General Procedure 8 for the tertiary α-alkylation of enolate derivatives

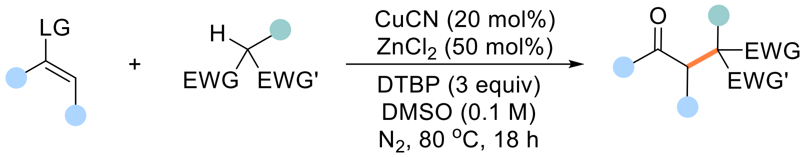

An oven-dried 10 mL Schlenk tube equipped with a stir bar was charged with CuCN (2.7 mg, 0.03 mmol, 20 mol%) and $ZnCl_2$ (10.2 mg, 0.075 mmol, 50 mol%). The tube was evacuated and backfilled with N_2 for 3 times. DTBP (65.8 mg, 0.45 mmol, 3.0 equiv), enolate (0.3 mmol, 2.0 equiv) and tertiary alkane (0.15 mmol, 1.0 equiv) and DMSO (1.5 mL) were successively added via syringe under N_2 atmosphere. The sealed tube was placed into a preheated oil bath at 80 °C with stirring for 18 h. After cooling to room temperature, the mixture was diluted with water (10 mL) and the aqueous layer was extracted with CH_2Cl_2 (10 mL × 3). The combined organic layers were rinsed with saturated NaCl (10 mL), dried over anhydrous Na_2SO_4, filtered and the filtrate was concentrated under reduced pressure. The resultant residue was purified by flash chromatography on silica gel (PE/EtOAc) to afford the sp^3-sp^3 coupling product.

6. Derivation of the products 3 and 4

6.1 Synthesis of compound 68

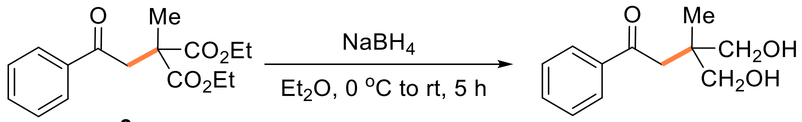

3 **68**, 90%

An oven-dried 10 mL Schlenk tube equipped with a stir bar was charged with **3** (29.2 mg, 0.10 mmol, 1 equiv) and Et_2O (2 mL), and $NaBH_4$ (22.7 mg, 0.60 mmol, 6 equiv) at 0 °C. The reaction was stirred for 5 h at room temperature. After completion, the reaction was quenched with water (10 mL), partitioned and the aqueous phase was extracted with Et_2O (10 mL × 3). The combined organic layers were rinsed with saturated NaCl (10 mL), dried over anhydrous Na_2SO_4 and filtered. The filtrate was concentrated under reduced pressure that was purified by flash chromatography on silica gel to afford **68** (19.0 mg, 0.090 mmol, 90%). R_f = 0.12 (PE/EtOAc = 1:1). ^{1}H NMR (400 MHz, $CDCl_3$) δ 7.39 – 7.27 (m, 5H), 4.93 (d, J = 10.5 Hz, 1H), 3.90 – 3.58 (m, 2H), 3.57 – 3.33 (m, 4H), 2.14 (dd, J = 15.2, 10.5 Hz, 1H), 1.79 (brs, 1H), 1.51 (d, J = 15.1 Hz, 1H), 0.87 (s, 3H). ^{13}C NMR (101 MHz, $CDCl_3$) δ 145.5, 128.6 × 2, 127.6, 125.5 × 2, 71.3, 71.0, 68.8, 45.1, 39.5, 20.1. HRMS (ESI): m/z calculated for $C_{12}H_{19}O_3$ $[M + H]^+$: 211.1329, found: 211.1336.

6.2 Synthesis of compound 69

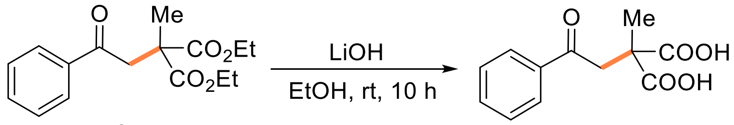

3 **69**, 95%

An oven-dried 10 mL Schlenk tube equipped with a stir bar was charged with **3** (29.2 mg, 0.10 mmol, 1.0 equiv), aqueous lithium hydroxide (2.5 M, 1.3 mL) and ethanol (1 mL) sequentially. The mixture was stirred for 10 h at room temperature. The reaction is completed, diluted with CH_2Cl_2 (5 mL) and extracted with water (5 mL × 3). The pH was then

adjusted to 2 with HCl solution (1.0 M). The aqueous phase was extracted with EtOAc (10 mL × 3). The combined organic layers were rinsed with saturated NaCl (10 mL), dried over anhydrous Na_2SO_4, filtered and the filtrate was concentrated under reduced pressure to afford **69** (22.4 mg, 0.095 mmol, 95%). R_f = 0.36 (CH_2Cl_2/MeOH = 6:1). ^1H NMR (400 MHz, DMSO-d_6) δ 12.87 (brs, 2H), 8.00 – 7.86 (m, 2H), 7.69 – 7.59 (m, 1H), 7.57 – 7.48 (m, 2H), 3.60 (s, 2H), 1.45 (s, 3H). ^{13}C NMR (101 MHz, DMSO-d_6) δ 197.6, 173.5 × 2, 137.0, 133.9, 129.3 × 2, 128.3 × 2, 51.3, 44.4, 20.8. HRMS (ESI): m/z calculated for $C_{12}H_{13}O_5$ [M + H]$^+$: 237.0757, found: 237.0759.

6.3 Synthesis of compound 70

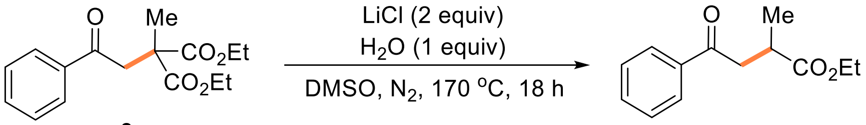

3 **70**, 71%

An oven-dried 10 mL Schlenk tube equipped with a stir bar was charged with **3** (29.2 mg, 0.10 mmol, 1.0 equiv), LiCl (8.5 mg, 0.20 mmol, 2.0 equiv), H_2O (1.8 mg, 0.10 mmol, 1.0 equiv) and DMSO (1 mL). The reaction was stirred at 170 °C for 18 hours. After cooling to room temperature, the mixture was diluted with water (10 mL) and the aqueous layer was extracted with CH_2Cl_2 (10 mL × 3). The combined organic layers were rinsed with saturated NaCl (10 mL), dried over anhydrous Na_2SO_4, filtered and the filtrate was concentrated under reduced pressure. The resultant residue was purified by flash chromatography on silica gel to afford **70** (15.6 mg, 0.071 mmol, 71%). R_f = 0.46 (PE/EtOAc = 8:1). ^1H NMR (400 MHz, $CDCl_3$) δ 8.00 – 7.93 (m, 2H), 7.60 – 7.53 (m, 1H), 7.50 – 7.41 (m, 2H), 4.15 (q, J = 7.1 Hz, 2H), 3.49 (dd, J = 17.5, 7.9 Hz, 1H), 3.19 – 3.06 (m, 1H), 3.02 (dd, J = 17.6, 5.5 Hz, 1H), 1.32 – 1.07 (m, 6H). ^{13}C NMR (101 MHz, $CDCl_3$) δ 198.3, 176.1, 136.8, 133.3, 128.7 × 2, 128.2 × 2, 60.7, 42.1, 35.1, 17.5, 14.3. HRMS (ESI): m/z calculated for $C_{13}H_{17}O_3$ [M + H]$^+$: 221.1172, found: 221.1174.

6.4 Synthesis of compound 71

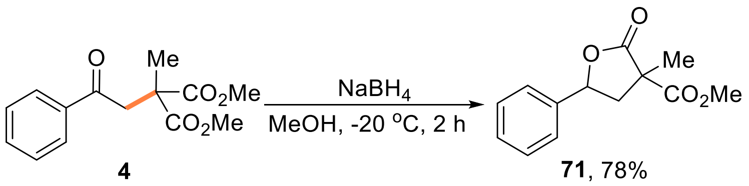

4 **71**, 78%

An oven-dried 10 mL Schlenk tube equipped with a stir bar was charged with **4** (26.4 mg, 0.10 mmol, 1 equiv), sodium borohydride (22.7 mg, 0.60 mmol, 6 equiv), and methanol (2 mL) at -20 °C. The reaction was stirred for 2 h at -20 °C. The reaction was quenched with water (10 mL), partitioned and the aqueous phase was extracted with Et_2O (10 mL × 3). The combined organic layers were rinsed with saturated NaCl (10 mL), dried over anhydrous Na_2SO_4, filtered and the filtrate was concentrated under reduced pressure. The resultant residue was purified by flash chromatography on silica gel to afford **71** (d.r. = 1.5:1, 18.2 mg, 0.078 mmol, 78%). R_f = 0.26 (PE/EtOAc = 10:1). ^1H NMR (400 MHz, $CDCl_3$) δ 7.49 – 7.31 (m, 5H), 5.58 (dd, J = 10.2, 6.1 Hz, 0.6H), 5.52 (dd, J = 9.2, 6.6 Hz, 0.4H), 3.84 (s, 1.8H), 3.74 (s, 1.2H), 3.11 (dd, J = 13.4, 6.1Hz, 0.6H), 2.85 (dd, J = 13.1, 9.1 Hz, 0.4H), 2.56 (dd, J = 13.1, 6.7 Hz, 0.4H), 2.11 (dd, J = 13.4, 10.3 Hz, 0.6H),

1.65 (s, 1.2H), 1.59 (s, 1.8H). ^{13}C NMR (101 MHz, $CDCl_3$) δ 175.5 & 175.0 (two isomers), 171.1 & 170.9 (two isomers), 138.6 & 138.4 (two isomers), 129.0 × 2 & 128.9 × 2 (two isomers), 128.88 & 128.85 (two isomers), 125.7 × 2 & 125.6 × 2 (two isomers), 79.1 & 78.9 (two isomers), 53.5 & 53.3 (two isomers), 52.2 & 51.6 (two isomers), 44.0 & 43.1 (two isomers), 21.1 & 19.8 (two isomers). HRMS (ESI): m/z calculated for $C_{13}H_{15}O_4$ $[M + H]^+$: 235.0965, found: 235.0964.

7. Mechanistic considerations.

7.1 Radical trapping experiments

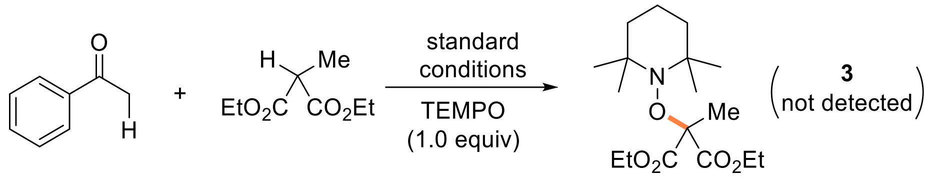

An oven-dried 10 mL Schlenk tube equipped with a stir bar was charged with CuCN (2.7 mg, 0.03 mmol, 20 mol%), TEMPO (23.5 mg, 0.15 mmol, 1.0 equiv)) and $ZnCl_2$ (10.2 mg, 0.075 mmol, 50 mol%). The tube was evacuated and backfilled with N_2 for 3 times. Azetidine (1.7 mg, 0.03 mmol, 20 mol%), DTBP (65.8 mg, 0.45 mmol, 3.0 equiv), **1** (18.0 mg, 0.15 mmol, 1.0 equiv), **2** (52.3 mg, 0.3 mmol, 2.0 equiv) and DMSO (1.5 mL) were successively added via syringe under N_2 atmosphere. The sealed tube was placed into a preheated oil bath at 80 °C with stirring for 12 h. After cooling to room temperature, the mixture was diluted with water (10 mL) and the aqueous layer was extracted with CH_2Cl_2 (10 mL × 3). The combined organic layers were rinsed with saturated NaCl (10 mL), dried over anhydrous Na_2SO_4, filtered and the filtrate was concentrated under reduced pressure. The resultant residue was purified by flash chromatography on silica gel (PE/EtOAc) to afford TEMPO-adduct **72** (6.4 mg, 0.019 mmol, 13%). ^1H NMR (400 MHz, $CDCl_3$) δ 4.35 – 4.13 (m, 4H), 1.74 (s, 3H), 1.57 – 1.38 (m, 6H), 1.28 (t, J = 7.1 Hz, 6H), 1.23 (s, 6H), 1.03 (s, 6H). This data is consistent with the one that reported in the literature.[4]

7.2 Evaluation of enamine catalysis

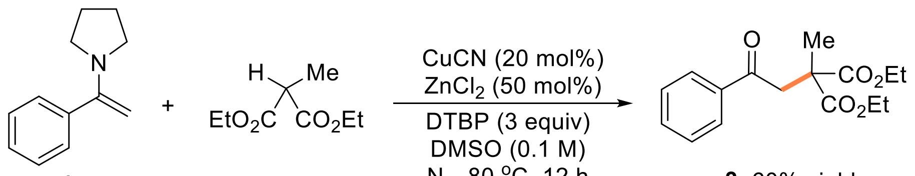

An oven-dried 10 mL Schlenk tube equipped with a stir bar was charged with CuCN (2.7 mg, 0.03 mmol, 20 mol%), $ZnCl_2$ (10.2 mg, 0.075 mmol, 0.5 equiv), evacuated and replaced 3 times with N_2. Under N_2 atmosphere, **1g** (26.0 mg, 0.15 mmol, 1.0 equiv), **2** (39.2 mg, 0.225 mmol, 1.5 equiv), DTBP (65.8 mg, 0.45 mmol, 3.0 equiv) and DMSO (1.5 mL) were added sequentially via syringe. The sealed tube was placed into a preheated oil bath at 80 °C with stirring for 12 h. After cooling to room temperature, the mixture was diluted with water (10 mL) and the aqueous layer was extracted with CH_2Cl_2 (10 mL × 3). The combined organic layers were rinsed with saturated NaCl (10 mL), dried over anhydrous Na_2SO_4, filtered and the filtrate was concentrated under reduced pressure. The resultant residue was purified by flash

chromatography on silica gel (PE/EtOAc = 10:1) to afford **3** (26.2 mg, 0.09 mmol, 60%).

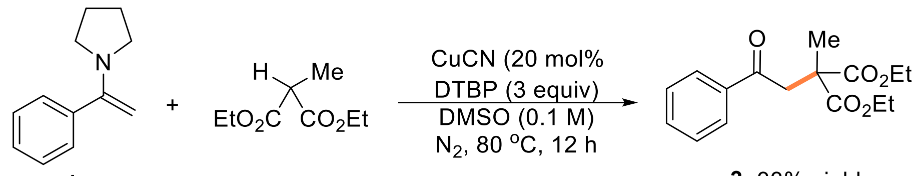

An oven-dried 10 mL Schlenk tube equipped with a stir bar was charged with CuCN (2.7 mg, 0.03 mmol, 20 mol%), evacuated and replaced 3 times with N_2. Under N_2 atmosphere, **1g** (26.0 mg, 0.15 mmol, 1.0 equiv), **2** (39.2 mg, 0.225 mmol, 1.5 equiv), DTBP (65.8 mg, 0.45 mmol, 3.0 equiv) and DMSO (1.5 mL) were added sequentially via syringe. The sealed tube was placed into a preheated oil bath at 80 °C with stirring for 12 h. After cooling to room temperature, the mixture was diluted with water (10 mL) and the aqueous layer was extracted with CH_2Cl_2 (10 mL × 3). The combined organic layers were rinsed with saturated NaCl (10 mL), dried over anhydrous Na_2SO_4, filtered and the filtrate was concentrated under reduced pressure. The resultant residue was purified by flash chromatography on silica gel (PE/EtOAc = 10:1) to afford **3** (14.5 mg, 0.050 mmol, 33%).

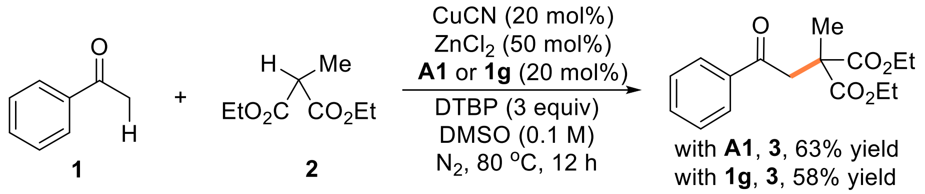

An oven-dried 10 mL Schlenk tube equipped with a stir bar was charged with CuCN (2.7 mg, 0.03 mmol, 20 mol%), $ZnCl_2$ (10.2 mg, 0.075 mmol, 0.5 equiv). The tube was evacuated and backfilled with N_2 for 3 times. Acetophenone **1** (18.0 mg, 0.15 mmol,1.0 equiv), **A1** (2.1 mg, 0.03 mmol, 20 mol%) or **1g** (5.2 mg, 0.03 mmol, 20 mol%), **2** (39.2 mg, 0.225 mmol, 1.5 equiv), DTBP (65.8 mg, 0.45 mmol, 3.0 equiv) and DMSO (1.5 mL). The sealed tube was placed into a preheated oil bath at 80 °C with stirring for 12 h. After cooling to room temperature, the mixture was diluted with water (10 mL) and the aqueous layer was extracted with CH_2Cl_2 (10 mL × 3). The combined organic layers were rinsed with saturated NaCl (10 mL), dried over anhydrous Na_2SO_4, filtered and the filtrate was concentrated under reduced pressure. The resultant residue was purified by flash chromatography on silica gel (PE /EtOAc = 10:1) to afford **3** (with **A1**, 27.6 mg, 0.094 mmol, 63%) or **3** (with **1g**, 25.4 mg, 0.087 mmol, 58%).

8. Characterization data of the products

Diethyl 2-methyl-2-(2-oxo-2-phenylethyl)malonate (3)

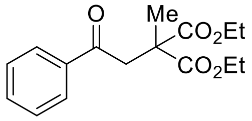

The title compound was prepared according to **GP7** and isolated as a pale yellow oil (36.8 mg, 0.126 mmol, 84%). R_f = 0.33 (PE/EtOAc = 10:1). ^{1}H NMR (400 MHz, $CDCl_3$) δ 8.15 – 7.84 (m, 2H), 7.63 – 7.55 (m, 1H), 7.51 – 7.36 (m, 2H), 4.21 (q, J = 7.2 Hz, 4H), 3.68 (s, 2H), 1.60 (s, 3H), 1.25 (t, J = 7.1 Hz, 6H). ^{13}C NMR (101 MHz, $CDCl_3$) δ 196.5, 171.6 × 2, 136.6, 133.3, 128.6 × 2, 128.0 × 2, 61.6 × 2, 51.5, 44.2, 20.5, 13.9 × 2. HRMS (ESI): m/z calculated for $C_{16}H_{21}O_5$ [M + H]$^+$: 293.1384, found: 293.1387.

Dimethyl 2-methyl-2-(2-oxo-2-phenylethyl)malonate (4)

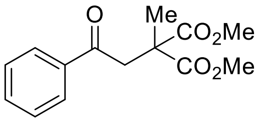

The title compound was prepared according to **GP7** and isolated as a pale yellow oil (32.3 mg, 0.122 mmol, 81%). R_f = 0.33 (PE/EtOAc = 10:1). ^{1}H NMR (400 MHz, $CDCl_3$) *δ* 7.99 – 7.93 (m, 2H), 7.62 – 7.55 (m, 1H), 7.50 – 7.43 (m, 2H), 3.76 (d, *J* = 0.7 Hz, 6H), 3.69 (s, 2H), 1.61 (s, 3H). ^{13}C NMR (101 MHz, $CDCl_3$) *δ* 196.5, 172.2 × 2, 136.5, 133.6, 128.8 × 2, 128.1 × 2, 53.0 × 2, 51.5, 44.6, 20.8. HRMS (ESI): m/z calculated for $C_{14}H_{17}O_5$ [M + H]$^+$: 265.1071, found: 265.1075.

Diethyl 2-ethyl-2-(2-oxo-2-phenylethyl)malonate (5)

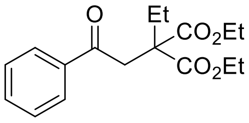

The title compound was prepared according to **GP7** and isolated as a colorless oil (36.7 mg, 0.120 mmol, 80%). R_f = 0.6 (PE/EtOAc = 10:1). ^{1}H NMR (400 MHz, $CDCl_3$) *δ* 8.01 – 7.95 (m, 2H), 7.60 – 7.53 (m, 1H), 7.50 – 7.44 (m, 2H), 4.21 (q, *J* = 7.1 Hz, 4H), 3.68 (s, 2H), 2.16 (q, *J* = 7.6 Hz, 2H), 1.24 (t, *J* = 7.1 Hz, 6H), 0.86 (t, *J* = 7.6 Hz, 3H). ^{13}C NMR (101 MHz, $CDCl_3$) *δ* 196.9, 171.1 × 2, 136.7, 133.4, 128.7 × 2, 128.1 × 2, 61.6 × 2, 55.9, 40.8, 26.2, 14.1 × 2, 9.2. HRMS (ESI): m/z calculated for $C_{17}H_{23}O_5$ [M + H]$^+$: 307.1540, found: 307.1543.

Diethyl 2-benzyl-2-(2-oxo-2-phenylethyl)malonate (6)

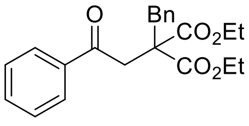

The title compound was prepared according to a modified **GP7** (at 90 °C) and isolated as a pale yellow solid (39.6 mg, 0.107 mmol, 72%). R_f = 0.57 (PE/EtOAc = 10:1). M.p.: 69.4 – 71.2 °C. ^{1}H NMR (400 MHz, $CDCl_3$) *δ* 7.95 – 7.89 (m, 2H), 7.61 – 7.53 (m, 1H), 7.49 – 7.41 (m, 2H), 7.22 – 7.14 (m, 3H), 7.01 – 6.89 (m, 2H), 4.24 (qd, *J* = 7.1, 1.1 Hz, 4H), 3.53 (s, 4H), 1.27 (t, *J* = 7.1 Hz, 6H). ^{13}C NMR (101 MHz, $CDCl_3$) *δ* 197.4, 170.5 × 2, 136.7, 136.5, 133.6, 130.1 × 2, 128.8 × 2, 128.6 × 2, 128.2 × 2, 127.2, 61.9 × 2, 56.6, 40.8, 38.4, 14.2 × 2. HRMS (ESI): m/z calculated for $C_{22}H_{25}O_5$ [M + H]$^+$: 369.1697, found: 369.1704.

Triethyl 6-oxo-6-phenylPE-1,4,4-tricarboxylate (7)

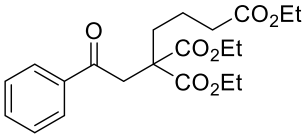

The title compound was prepared according to **GP7** and isolated as a pale yellow oil (41.6 mg, 0.106 mmol, 71%). R_f = 0.37 (PE/EtOAc = 10:1). ^{1}H NMR (400 MHz, $CDCl_3$) *δ* 8.07 – 7.87 (m, 2H), 7.63 – 7.51 (m, 1H), 7.46 (td, *J* = 7.8, 1.6 Hz, 2H), 4.20 (q, *J* = 7.1 Hz, 4H), 4.06 (q, *J* = 7.1 Hz, 2H), 3.71 (s, 2H), 2.27 (t, *J* = 7.5 Hz, 2H), 2.18 – 2.07 (m, 2H), 1.60 – 1.48 (m, 2H), 1.26 – 1.17 (m, 9H). ^{13}C NMR (101 MHz, $CDCl_3$) *δ* 196.7, 173.1, 170.9 × 2, 136.6, 133.5, 128.8 × 2, 128.2 × 2, 61.8 × 2, 60.5, 55.3, 41.3, 34.3, 32.5, 20.4, 14.3, 14.1 × 2. HRMS (ESI): m/z calculated for $C_{21}H_{29}O_7$ [M + H]$^+$: 393.1908, found: 393.1909.

Diethyl 2-(cyclopentylmethyl)-2-(2-oxo-2-phenylethyl)malonate (8)

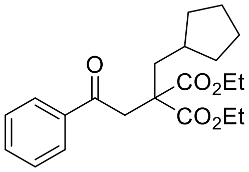

The title compound was prepared according to **GP7** and isolated as a pale yellow oil (35.4 mg, 0.098 mmol, 65%). R_f = 0.63 (PE/EtOAc = 10:1). ^{1}H NMR (400 MHz, $CDCl_3$) *δ* 8.03 – 7.94 (m, 2H), 7.62 – 7.54 (m, 1H), 7.51 – 7.43 (m, 2H), 4.26 – 4.12 (m, 4H), 3.73 (s, 2H), 2.08 (d, *J* =

5.9 Hz, 2H), 1.66 – 1.47 (m, 4H) 1.23 (t, *J* = 7.1 Hz, 6H), 1.19 – 1.05 (m, 3H), 0.98 – 0.86 (m, 2H). ^{13}C NMR (101 MHz, $CDCl_3$) *δ* 196.9, 171.5 × 2, 136.8, 133.4, 128.8 × 2, 128.1 × 2, 61.6 × 2, 54.9, 41.6, 34.3 × 2, 34.2, 26.3, 26.4 × 2, 14.1 × 2. HRMS (ESI): m/z calculated for $C_{21}H_{29}O_5$ [M + H]$^+$: 361.2010, found: 361.2012.

Diethyl 2-(cyclopropylmethyl)-2-(2-oxo-2-phenylethyl)malonate (9)

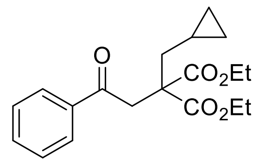

The title compound was prepared according to **GP7** and isolated as a pale yellow oil (36.8 mg, 0.111 mmol, 74%). R_f = 0.60 (PE/EtOAc = 10:1). ^{1}H NMR (400 MHz, $CDCl_3$) *δ* 8.03 – 7.97 (m, 2H), 7.62 – 7.55 (m, 1H), 7.50 – 7.44 (m, 2H), 4.21 (qd, *J* = 7.1, 2.1 Hz, 4H), 3.87 (s, 2H), 2.08 (d, *J* = 6.9 Hz, 2H), 1.24 (t, *J* = 7.1 Hz, 6H), 0.71 – 0.52 (m, 1H), 0.44 – 0.34 (m, 2H), 0.01 – -0.08 (m, 2H). ^{13}C NMR (101 MHz, $CDCl_3$) *δ* 197.1, 171.1 × 2, 136.7, 133.4, 128.8 × 2, 128.1 × 2, 61.6 × 2, 55.8, 41.4, 37.4, 14.1 × 2, 6.7, 4.4 × 2. HRMS (ESI): m/z calculated for $C_{19}H_{25}O_5$ [M + H]$^+$: 333.1697, found: 333.1700.

Diethyl 2-(2-oxo-2-phenylethyl)-2-((tetrahydrofuran-2-yl)methyl)malonate (10)

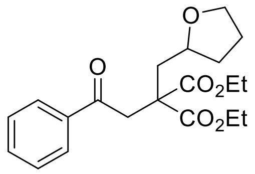

The title compound was prepared according to **GP7** and isolated as a pale yellow oil (38.3 mg, 0.106 mmol, 70%). R_f = 0.37 (PE/EtOAc = 10:1). ^{1}H NMR (400 MHz, $CDCl_3$) *δ* 8.03 – 7.95 (m, 2H), 7.61 – 7.50 (m, 1H), 7.49 – 7.41 (m, 2H), 4.20 (qd, *J* = 7.1, 0.7 Hz, 4H), 4.06 (d, *J* = 18.4 Hz, 1H), 3.92 – 3.82 (m, 1H), 3.78 (d, *J* = 18.4 Hz, 1H), 3.64 – 3.50 (m, 2H), 2.41 (dd, *J* = 14.8, 2.5 Hz, 1H), 2.31 (dd, *J* = 14.8, 10.4 Hz, 1H), 2.07 – 1.92 (m, 1H), 1.88 – 1.70 (m, 2H), 1.55 – 1.43 (m, 1H), 1.29 – 1.20 (m, 6H). ^{13}C NMR (101 MHz, $CDCl_3$) *δ* 197.4, 171.2, 171.0, 137.0, 133.2, 128.6 × 2, 128.2 × 2, 75.5, 67.7, 61.7, 61.6, 54.6, 41.5, 38.2, 32.5, 25.4, 14.1 × 2. HRMS (ESI): m/z calculated for $C_{20}H_{27}O_6$ [M + H]$^+$: 363.1802, found: 363.1803.

Diethyl 2-(2-((*tert*-butyldimethylsilyl)oxy)ethyl)-2-(2-oxo-2-phenylethyl)malonate (11)

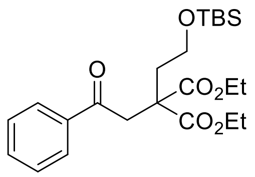

The title compound was prepared according to **GP7** and isolated as a pale yellow oil (46.5 mg, 0.106 mmol, 71%). R_f = 0.67 (PE/EtOAc = 10:1). ^{1}H NMR (400 MHz, $CDCl_3$) *δ* 8.01 – 7.94 (m, 2H), 7.60 – 7.50 (m, 1H), 7.49 – 7.41 (m, 2H), 4.24 – 4.15 (m, 4H), 3.85 (s, 2H), 3.65 (t, *J* = 6.1 Hz, 2H), 2.41 (t, *J* = 6.1 Hz, 2H), 1.23 (t, *J* = 7.1 Hz, 6H), 0.78 (s, 9H), -0.08 (s, 6H). ^{13}C NMR (101 MHz, $CDCl_3$) *δ* 196.9, 171.0 × 2, 136.7, 133.3, 128.6 × 2, 128.2 × 2, 61.6 × 2, 59.9, 54.3, 41.7, 35.2, 25.9 × 3, 18.4, 14.1 × 2, 5.5 × 2. HRMS (ESI): m/z calculated for $C_{23}H_{37}SiO_6$ [M + H]$^+$: 437.2354, found: 437.2354.

Diethyl 2-(3-methylbut-2-en-1-yl)-2-(2-oxo-2-phenylethyl)malonate (12)

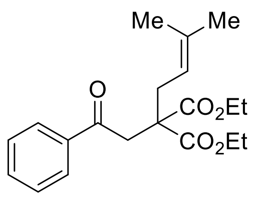

The title compound was prepared according to **GP7** and isolated as a pale yellow oil (36.3 mg, 0.105 mmol, 70%). R_f = 0.57 (PE/EtOAc = 10:1). ^{1}H NMR (400 MHz, $CDCl_3$) *δ* 8.00 – 7.92 (m, 2H), 7.60 – 7.53 (m, 1H), 7.45 (dd, *J* = 8.4, 7.0 Hz, 2H), 4.98 – 4.91 (m, 1H) , 4.21 (q, *J* = 7.1 Hz, 4H), 3.65 (s, 2H), 2.84 (d, *J* = 7.7 Hz, 2H), 1.61 (d, *J* = 1.4 Hz, 3H), 1.42 (d, *J* = 1.3 Hz, 3H), 1.23 (t, *J* = 7.1 Hz, 6H). ^{13}C NMR (101 MHz, $CDCl_3$) *δ* 197.2, 170.9 × 2, 136.8, 136.5,

133.4, 128.7 × 2, 128.1 × 2, 118.3, 61.7 × 2, 55.6, 41.0, 31.7, 26.1, 17.9, 14.1 × 2. HRMS (ESI): m/z calculated for $C_{20}H_{27}O_5$ [M + H]$^+$: 347.1853, found: 347.1856.

Diethyl 2-(but-2-yn-1-yl)-2-(2-oxo-2-phenylethyl)malonate (13)

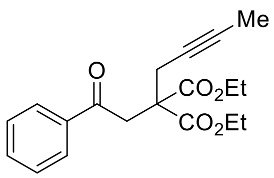

The title compound was prepared according to **GP7** and isolated as a colorless solid (35.6 mg, 0.108 mmol, 72%). R_f = 0.53 (PE/EtOAc = 10:1). M.p.: 66.2 – 67.9 °C. ^{1}H NMR (400 MHz, $CDCl_3$) *δ* 8.06 – 7.96 (m, 2H), 7.64 – 7.54 (m, 1H), 7.53 – 7.42 (m, 2H), 4.22 (qd, *J* = 7.1, 1.7 Hz, 4H), 3.88 (s, 2H), 3.04 (q, *J* = 2.6 Hz, 2H), 1.70 (t, *J* = 2.6 Hz, 3H), 1.24 (t, *J* = 7.1 Hz, 6H). ^{13}C NMR (101 MHz, $CDCl_3$) *δ* 197.1, 169.8 × 2, 136.7, 133.5, 128.8 × 2, 128.3 × 2, 79.3, 74.1, 62.0 × 2, 55.1, 41.1, 23.8, 14.1 × 2, 3.7. HRMS (ESI): m/z calculated for $C_{19}H_{23}O_5$ [M + H]$^+$: 331.1540, found: 331.1543.

Ethyl 2-acetyl-2-methyl-4-oxo-4-phenylbutanoate (14)

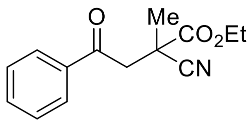

The title compound was prepared according to **GP7** and isolated as a white solid (27.7 mg, 0.113 mmol, 75%). R_f = 0.33 (PE/EtOAc = 4:1). M.p.: 67.5 – 69.4 °C. ^{1}H NMR (400 MHz, $CDCl_3$) *δ* 7.98 – 7.91 (m, 2H), 7.64 – 7.58 (m, 1H), 7.51 – 7.46 (m, 2H), 4.32 (qd, *J* = 7.1, 3.3 Hz, 2H), 3.74 (d, *J* = 18.0 Hz, 1H), 3.56 (d, *J* = 17.9 Hz, 1H), 1.74 (s, 3H), 1.36 (t, *J* = 7.1 Hz, 3H). ^{13}C NMR (101 MHz, $CDCl_3$) *δ* 194.5, 169.3, 135.4, 134.1, 128.9 × 2, 128.2 × 2, 120.0, 63.2, 46.5, 40.0, 23.9, 14.1. HRMS (ESI): m/z calculated for $C_{14}H_{16}NO_3$ [M + H]$^+$: 246.1125, found: 246.1130.

Ethyl 2-acetyl-2-methyl-4-oxo-4-phenylbutanoate (15)

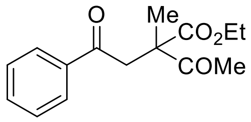

The title compound was prepared according to **GP7** and isolated as a pale yellow oil (36.3 mg, 0.138 mmol, 92%). R_f = 0.3 (PE/EtOAc = 10:1). ^{1}H NMR (400 MHz, $CDCl_3$) *δ* 7.99 – 7.91 (m, 2H), 7.60 – 7.52 (m, 1H), 7.51 – 7.39 (m, 2H), 4.20 (q, *J* = 7.1 Hz, 2H), 3.72 – 3.55 (m, 2H), 2.33 (s, 3H), 1.56 (s, 3H), 1.23 (t, *J* = 7.1 Hz, 3H). ^{13}C NMR (101 MHz, $CDCl_3$) *δ* 205.8, 197.2, 172.4, 136.6, 133.5, 128.7 × 2, 128.2 × 2, 61.7, 57.4, 44.7, 26.6, 20.8, 14.1. HRMS (ESI): m/z calculated for $C_{15}H_{19}O_4$ [M + H]$^+$: 263.1278, found: 263.1278.

2-Methyl-2-(2-oxo-2-phenylethyl)malononitrile (16)

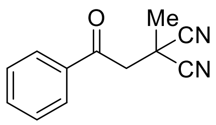

The title compound was prepared according to **GP7** and isolated as a pale yellow oil (20.5 mg, 0.103 mmol, 69%). R_f = 0.25 (PE/EtOAc = 4:1). ^{1}H NMR (400 MHz, $CDCl_3$) *δ* 8.02 – 7.86 (m, 2H), 7.74 – 7.61 (m, 1H), 7.59 – 7.48 (m, 2H), 3.70 (s, 2H), 1.99 (s, 3H). ^{13}C NMR (101 MHz, $CDCl_3$) *δ* 191.9, 134.9, 134.8, 129.2 × 2, 128.3 × 2, 116.1 × 2, 46.4, 27.7, 24.8. HRMS (ESI): m/z calculated for $C_{12}H_{11}N_2O$ [M + H]$^+$: 199.0866, found: 199.0872.

3-Acetyl-3-methyl-1-phenylpentane-1,4-dione (17)

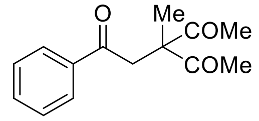

The title compound was prepared according to **GP7** and isolated as a colorless oil (8.7 mg, 0.037 mmol, 25%). R_f = 0.31 (PE/EtOAc = 6:1). ^{1}H NMR (400 MHz, $CDCl_3$) δ 8.00 – 7.93 (m, 2H), 7.62 – 7.56 (m, 1H), 7.52 – 7.44 (m, 2H), 3.71 (s, 2H), 2.23 (s, 6H), 1.56 (s, 3H) ^{13}C NMR (101 MHz, $CDCl_3$) δ 206.2 × 2, 197.3, 136.3, 133.6, 128.7 × 2, 128.1 × 2, 64.5, 44.8, 26.5 × 2, 19.5. This data is consistent with the one that reported in the literature.[11]

Ethyl 2-acetyl-2-(4-cyanobenzyl)-4-oxo-4-phenylbutanoate (18)

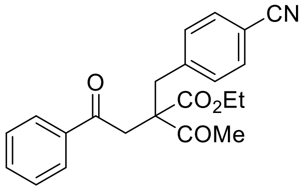

The title compound was prepared according to **GP7** and isolated as a colorless oil (42.4 mg, 0.117 mmol, 78%). R_f = 0.15 (PE/EtOAc = 10:1). ^{1}H NMR (400 MHz, $CDCl_3$) δ 7.98 – 7.79 (m, 2H), 7.65 – 7.55 (m, 1H), 7.53 – 7.41 (m, 4H), 7.12 – 7.00 (m, 2H), 4.22 (q, *J* = 7.1 Hz, 2H), 3.67 – 3.36 (m, 4H), 2.42 (s, 3H), 1.24 (t, *J* = 7.1 Hz, 3H). ^{13}C NMR (101 MHz, $CDCl_3$) δ 204.1, 197.4, 171.0, 142.1, 136.2, 133.9, 132.4 × 2, 130.8 × 2, 128.9 × 2, 128.2 × 2, 118.7, 111.4, 62.17, 62.15, 41.7, 39.0, 27.4, 14.1. HRMS (ESI): m/z calculated for $C_{22}H_{22}NO_4$ [M + H]$^+$: 364.1543, found: 364.1554.

Ethyl 2-acetyl-4-oxo-4-phenyl-2-(2-(pyridin-2-yl)ethyl)butanoate (19)

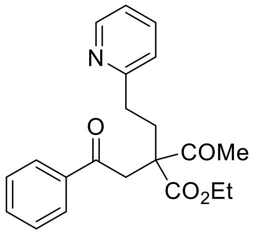

The title compound was prepared according to **GP7** and isolated as a pale yellow oil (38.3 mg, 0.108 mmol, 72%). R_f = 0.18 (PE/EtOAc = 4:1). ^{1}H NMR (400 MHz, $CDCl_3$) δ 8.47 (d, *J* = 4.9 Hz, 1H), 8.05 – 7.79 (m, 2H), 7.64 – 7.54 (m, 2H), 7.51 – 7.40 (m, 2H), 7.14 – 7.05 (m, 2H), 4.21 (q, *J* = 7.1 Hz, 2H), 3.76 (d, *J* = 3.2 Hz, 2H), 2.74 – 2.66 (m, 2H), 2.62 – 2.45 (m, 2H), 2.43 (s, 3H), 1.23 (t, *J* = 7.1 Hz, 3H). ^{13}C NMR (101 MHz, $CDCl_3$) δ 205.4, 197.3, 171.1, 143.5, 136.4, 133.6 × 2, 128.8 × 2, 128.2 × 2, 127.0, 124.6, 123.6, 61.8, 61.2, 42.0, 35.7, 27.6, 25.4, 14.2. HRMS (ESI): m/z calculated for $C_{21}H_{24}NO_4$ [M + H]$^+$: 354.1700, found: 354.1707.

Ethyl 2-acetyl-4-oxo-4-phenyl-2-(2-(thiophen-2-yl)ethyl)butanoate (20)

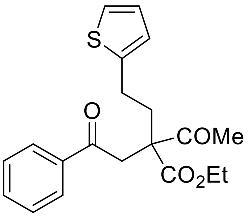

The title compound was prepared according to **GP7** and isolated as a yellow oil (40.3 mg, 0.112 mmol, 75%). R_f = 0.33 (PE/EtOAc = 10:1). ^{1}H NMR (400 MHz, $CDCl_3$) δ 7.98 – 7.94 (m, 2H), 7.63 – 7.54 (m, 1H), 7.47 (t, *J* = 7.7 Hz, 2H), 7.10 – 7.09 (m, 1H), 6.88 (dd, *J* = 5.2, 3.4 Hz, 1H), 6.77 (d, *J* = 3.4 Hz, 1H), 4.23 (qd, *J* = 7.1, 1.2 Hz, 2H), 3.80 – 3.66 (m, 2H), 2.77 – 2.71 (m, 2H), 2.63 – 2.49 (m, 1H), 2.47 – 2.36 (m, 4H), 1.25 (t, *J* = 7.2 Hz, 3H). ^{13}C NMR (101 MHz, $CDCl_3$) δ 205,4, 197.3, 171.6, 143.4, 136.4, 133.6, 128.7 × 2, 128.2 × 2, 126.9, 124.6, 123.6, 61.8, 61.1, 42.0, 35.7, 27.6, 25.4, 14.1. HRMS (ESI): m/z calculated for $C_{20}H_{23}SO_4$ [M + H]$^+$: 359.1312, found: 359.1315.

Ethyl 2-acetyl-6-(1*H*-indol-1-yl)-2-(2-oxo-2-phenylethyl)hexanoate (21)

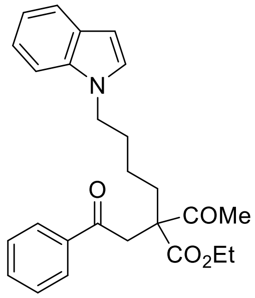

The title compound was prepared according to **GP7** and isolated as a yellow oil (43.8 mg, 0.104 mmol, 70%). R_f = 0.37 (PE/EtOAc = 10:1). ^{1}H NMR (400 MHz, $CDCl_3$) *δ* 7.95 – 7.91 (m, 2H), 7.63 – 7.56 (m, 2H), 7.50 – 7.44 (m, 2H), 7.30 (dd, *J* = 8.2, 1.0 Hz, 1H), 7.23 – 7.15 (m, 1H), 7.14 – 7.03 (m, 1H), 7.02 (d, *J* = 3.1 Hz, 1H), 6.42 (dd, *J* = 3.0, 0.8 Hz, 1H), 4.16 (q, *J* = 7.1 Hz, 2H), 4.10 (q, *J* = 7.0 Hz, 2H), 3.61 (d, *J* = 3.9 Hz, 2H), 2.33 (s, 3H), 2.21 – 2.10 (m, 1H), 2.09 – 1.97 (m, 1H), 1.84 (p, *J* = 7.2 Hz, 2H), 1.21 – 1.13 (m, 5H). ^{13}C NMR (101 MHz, $CDCl_3$) *δ* 205.5, 197.5, 171.8, 136.5, 135.9, 133.5, 128.8 × 2, 128.7, 128.2 × 2, 127.8, 121.5, 121.1, 119.4, 109.4, 101.2, 61.7, 61.2, 45.8, 41.8, 33.4, 30.3, 27.4, 22.0, 14.0. HRMS (ESI): m/z calculated for $C_{20}H_{23}SO_4$ [M + H]$^+$: 420.2169, found: 420.2172.

2-(5-Fluoropentyl)-2-(2-oxo-2-phenylethyl)malononitrile (22)

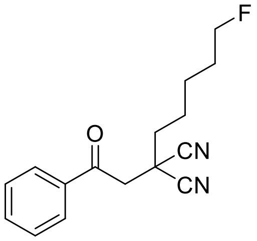

The title compound was prepared according to a modified **GP7** (for 18 h) and isolated as a pale yellow solid (26.9 mg, 0.099 mmol, 66%). R_f = 0.30 (PE/EtOAc = 4:1). M.p.: 87.3 – 90.4 °C. ^{1}H NMR (400 MHz, $CDCl_3$) *δ* 8.06 – 7.87 (m, 2H), 7.72 – 7.63 (m, 1H), 7.57 – 7.50 (m, 2H), 4.54 (t, *J* = 5.8 Hz, 1H), 4.43 (t, *J* = 5.8 Hz, 1H), 3.69 (s, 2H), 2.18 – 2.11 (m, 2H), 1.88 – 1.71 (m, 4H), 1.61 – 1.53 (m, 2H). ^{19}F NMR (376 MHz, CDCl3) *δ* -218.90. ^{13}C NMR (101 MHz, $CDCl_3$) *δ* 191.8, 134.9, 134.7, 129.7 × 2, 128.2 × 2, 115.3 × 2, 83.7 (d, *J* = 165.2 Hz), 45.3, 37.3, 33.3, 30.1 (d, *J* = 19.8 Hz), 25.3, 24.8 (d, *J* = 4.8 Hz). HRMS (ESI): m/z calculated for $C_{16}H_{18}FN_2O$ [M + H]$^+$: 273.1398, found: 273.1400.

2-(2-(1,3-Dioxolan-2-yl)ethyl)-2-(2-oxo-2-phenylethyl)malononitrile (23)

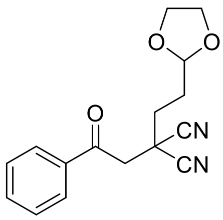

The title compound was prepared according to a modified **GP7** (for 18 h) and isolated as a pale yellow solid (26.8 mg, 0.094 mmol, 63%). R_f = 0.74 (PE/EtOAc = 1:1). M.p.: 132.4 – 134.4 °C. ^{1}H NMR (400 MHz, $CDCl_3$) *δ* 7.99 – 7.90 (m, 2H), 7.71 – 7.60 (m, 1H), 7.52 (t, *J* = 7.8 Hz, 2H), 5.01 (t, *J* = 4.0 Hz, 1H), 4.02 – 3.96 (m, 2H), 3.92 – 3.86 (m, 2H), 3.70 (s, 2H), 2.35 – 2.24 (m, 2H), 2.18 – 2.11 (m, 2H). ^{13}C NMR (101 MHz, $CDCl_3$) *δ* 191.8, 134.9, 134.7, 129.2 × 2, 128.2 × 2, 115.2 × 2, 102.3, 65.3 × 2, 45.3, 33.0, 31.4, 29.6. HRMS (ESI): m/z calculated for $C_{16}H_{17}N_2O_3$ [M + H]$^+$: 285.1234, found: 285.1237.

2-(4-(3-Bromo-9*H*-carbazol-9-yl)butyl)-2-(2-oxo-2-phenylethyl)malononitrile (24)

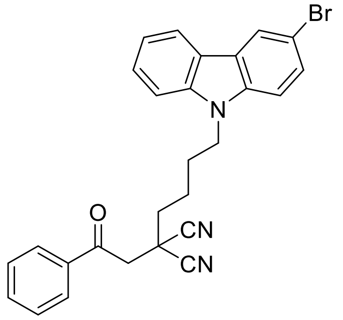

The title compound was prepared according to a modified **GP7** (at 90 ºC for 18 h) and isolated as a pale yellow solid (47.9 mg, 0.099 mmol, 66%). R_f = 0.24 (PE/EtOAc = 4:1). M.p.: 185.4 – 187.2 ºC. ^{1}H NMR (400 MHz, $CDCl_3$) *δ* 8.21 (d, *J* = 2.0 Hz, 1H), 8.05 (d, *J* = 7.6 Hz, 1H), 7.90 – 7.86 (m, 2H), 7.72 – 7.61 (m, 1H), 7.56 (dd, *J* = 8.7, 2.0 Hz, 1H), 7.54 – 7.48 (m, 3H), 7.41 (d, *J* = 2.0 Hz, 1H), 7.31 – 7.22 (m, 2H), 4,37 (t, *J* = 7.1 Hz, 2H), 3.56 (s, 2H), 2.15 – 2.07 (m, 2H), 2.01 (p, *J* = 7.2 Hz, 2H), 1.92 – 1.80 (m, 2H). ^{13}C NMR (101 MHz, $CDCl_3$) *δ* 194.6, 140.6, 138.9, 134.8, 134.7, 129.2 × 2, 128.6, 128.2 × 2, 126.8, 124.8, 123.4, 122.0, 120.9, 119.7, 115.1 × 2, 112.0, 110.0, 108.9, 45.0, 42.7, 37.0, 33.2, 28.2, 23.5. HRMS (ESI): m/z calculated for $C_{27}H_{23}BrN_3O$ [M + H]$^+$: 484.1019, found: 484.1028.

4,4-Dicyano-6-oxo-6-phenylhexyl adamantane-1-carboxylate (25)

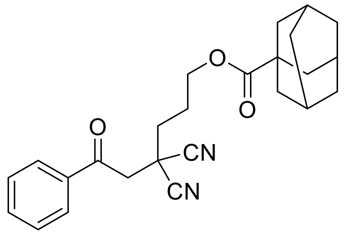

The title compound was prepared according to **GP7** and isolated as a pale yellow solid (39.2 mg, 0.097 mmol, 65%). R_f = 0.24 (PE/EtOAc = 4:1). M.p.: 116.9 – 117.4 ºC. ^{1}H NMR (400 MHz, $CDCl_3$) *δ* 8.02 – 7.85 (m, 2H), 7.70 – 7.63 (m, 1H), 7.58 – 7.47 (m, 2H), 4.18 (t, *J* = 5.8 Hz, 2H), 3.72 (s, 2H), 2.26 – 2.17 (m, 2H), 2.16 – 2.07 (m, 2H), 2.04 – 1.98 (m, 3H), 1.90 – 1.86 (m, 6H), 1.74 – 1.69 (m, 6H). ^{13}C NMR (101 MHz, $CDCl_3$) *δ* 191.6, 177.5, 134.6, 129.0 × 2, 128.1 × 2, 114.9 × 2, 62.0, 45.1, 40.7, 38.7 × 3, 36.32 × 2, 36.30, 34.2, 33.0, 27.8 × 3, 27.7, 25.0. HRMS (ESI): m/z calculated for $C_{25}H_{29}N_2O_3$ [M + H]$^+$: 405.2173, found: 405.2182.

Diethyl 2-methyl-2-(2-oxo-2-(*p*-tolyl)ethyl)malonate (26)

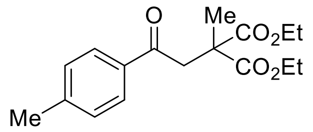

The title compound was prepared according to **GP7** and isolated as a pale yellow oil (40.3 mg, 0.132 mmol, 88%). R_f = 0.29 (PE/EtOAc = 10:1). ^{1}H NMR (400 MHz, $CDCl_3$) *δ* 7.85 (d, *J* = 8.2 Hz, 2H), 7.24 (d, *J* = 8.2 Hz, 2H), 4.19 (q, *J* = 7.1 Hz, 4H), 3.63 (s, 2H), 2.39 (s, 3H), 1.57 (s, 3H), 1.23 (t, *J* = 7.1 Hz, 6H). ^{13}C NMR (101 MHz, $CDCl_3$) *δ* 196.2, 171.8 × 2, 144.3, 134.3, 129.4 × 2, 128.2 × 2, 61.7 × 2, 51.6, 44.2, 21.8, 20.6, 14.1 × 2. HRMS (ESI): m/z calculated for $C_{17}H_{23}O_5$ [M + H]$^+$: 307.1540, found: 307.1541.

Diethyl 2-(2-(4-(*tert*-butyl)phenyl)-2-oxoethyl)-2-methylmalonate (27)

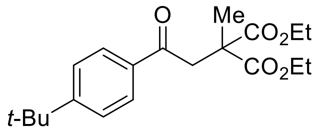

The title compound was prepared according to **GP7** and isolated as a pale yellow oil (43.0 mg, 0.123 mmol, 82%). R_f = 0.56 (PE/EtOAc = 10:1). ^{1}H NMR (400 MHz, $CDCl_3$) *δ* 7.90 (d, *J* = 8.8 Hz, 2H), 7.46 (d, *J* = 8.8 Hz, 2H), 4.19 (q, *J* = 7.1 Hz, 4H), 3.64 (s, 2H), 1.57 (s, 3H), 1.32 (s, 9H), 1.23 (t, *J* = 7.1 Hz, 6H). ^{13}C NMR (101 MHz, $CDCl_3$) *δ* 196.0, 171.6 × 2, 157.1, 134.0, 127.9 × 2, 125.5 × 2, 61.5 × 2, 51.4, 44.1, 35.1, 31.0 × 3, 20.5, 13.9 × 2. HRMS (ESI): m/z calculated for $C_{20}H_{29}O_5$ [M + H]$^+$: 349.2010, found: 349.2017.

Diethyl 2-(2-(4-methoxyphenyl)-2-oxoethyl)-2-methylmalonate (28)

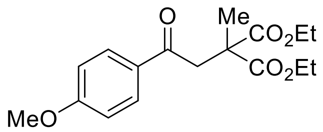

The title compound was prepared according to **GP7** and isolated as a pale yellow oil (42.9 mg, 0.133 mmol, 89%). R_f = 0.44 (PE/EtOAc = 10:1). ^{1}H NMR (400 MHz, $CDCl_3$) *δ* 8.02 – 7.85 (m, 2H), 7.01 – 6.86 (m, 2H), 4.20 (q, *J* = 7.1 Hz, 4H), 3.86 (s, 3H), 3.62 (s, 2H), 1.58 (s, 3H), 1.24 (t, *J* = 7.1 Hz, 6H). ^{13}C NMR (101 MHz, $CDCl_3$) *δ* 194.9, 171.7 × 2, 163.6, 130.2 × 2, 129.7 × 2, 113.7, 61.5 × 2, 55.4, 51.5, 43.8, 20.5, 13.9 × 2. HRMS (ESI): m/z calculated for $C_{17}H_{23}O_6$ [M + H]$^+$: 323.1489, found: 323.1490.

Diethyl 2-(2-(4-(dimethylamino)phenyl)-2-oxoethyl)-2-methylmalonate (29)

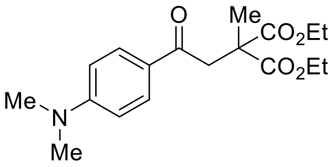

The title compound was prepared according to **GP7** and isolated as a yellow solid (25.0 mg, 0.075 mmol, 50%). R_f = 0.21 (PE/EtOAc = 10:1). M.p.: 53.4 – 55.1 ºC. ^{1}H NMR (400 MHz, $CDCl_3$) *δ* 7.87 (d, *J* = 9.1 Hz, 2H), 6.64 (d, *J* = 9.1 Hz, 2H), 4.20 (q, *J* = 7.2 Hz, 4H), 3.59 (s, 2H), 3.05 (s, 6H), 1.57 (s, 3H), 1.24 (t, *J* = 7.2 Hz, 6H). ^{13}C NMR (101 MHz, $CDCl_3$) *δ* 194.2, 171.9 × 2, 153.4, 130.2 × 2, 124.6, 110.5 × 2, 61.4 × 2, 51.5, 43.4, 40.0 × 2, 20.5, 13.9 × 2. HRMS (ESI): m/z calculated for $C_{18}H_{26}NO_5$ [M + H]$^+$: 336.1805, found: 336.1812.

Diethyl 2-(2-(4-chlorophenyl)-2-oxoethyl)-2-methylmalonate (30)

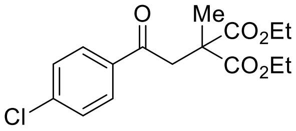

The title compound was prepared according to **GP7** and isolated as a pale yellow oil (38.3 mg, 0.117 mmol, 78%). R_f = 0.47 (PE/EtOAc = 10:1). ^{1}H NMR (400 MHz, $CDCl_3$) *δ* 7.90 (d, *J* = 8.5 Hz, 2H), 7.43 (d, *J* = 8.4 Hz, 2H), 4.20 (q, *J* = 7.2 Hz, 4H), 3.62 (s, 2H), 1.59 (s, 3H), 1.24 (t, *J* = 7.2 Hz, 6H). ^{13}C NMR (101 MHz, $CDCl_3$) *δ* 195.3, 171.5 × 2, 139.8, 134.9, 129.4 × 2, 128.9 × 2, 61.6 × 2, 51.5, 44.2, 20.5, 13.9 × 2. HRMS (ESI): m/z calculated for $C_{16}H_{20}ClO_5$ [M + H]$^+$: 327.0994, found: 327.0997.

Diethyl 2-(2-(4-bromophenyl)-2-oxoethyl)-2-methylmalonate (31)

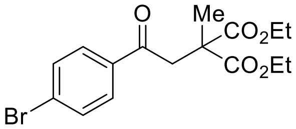

The title compound was prepared according to **GP7** and isolated as a pale yellow solid (40.2 mg, 0.108 mmol, 72%). R_f = 0.48 (PE/EtOAc = 10:1). M.p.: 43.4 – 45.6 ºC. ^{1}H NMR (400 MHz, $CDCl_3$) *δ* 7.82 (d, *J* = 8.5 Hz, 2H), 7.60 (d, *J* = 8.6 Hz, 2H), 4.20 (q, *J* = 7.1 Hz, 4H), 3.61 (s, 2H), 1.59 (s, 3H), 1.24 (t, *J* = 7.1 Hz, 6H). ^{13}C NMR (101 MHz, $CDCl_3$) *δ* 195.5, 171.5 × 2, 135.3, 131.9, 129.5 × 2, 128.5 × 2, 61.6 × 2, 51.5, 44.2, 20.5, 13.9 × 2. HRMS (ESI): m/z calculated for $C_{16}H_{20}BrO_5$ [M + H]$^+$: 371.0489, found: 371.0490.

Diethyl 2-(2-(4-cyanophenyl)-2-oxoethyl)-2-methylmalonate (32)

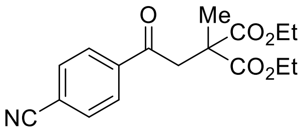

The title compound was prepared according to **GP7** and isolated as a pale yellow solid (35.0 mg, 0.110 mmol, 74%). R_f = 0.24 (PE/EtOAc = 10:1). M.p.: 93.1 – 94.2 ºC. ^{1}H NMR (400 MHz, $CDCl_3$) *δ* 8.10 – 8.02 (m, 2H), 7.82 – 7.74 (m, 2H), 4.21 (q, *J* = 7.1 Hz, 4H), 3.64 (s, 2H), 1.60 (s, 3H), 1.24 (t, *J* = 7.1 Hz, 6H). ^{13}C NMR (101 MHz, $CDCl_3$) *δ* 195.3, 171.3 × 2, 139.5, 132.5 × 2, 128.4

× 2, 117.8, 116.5, 61.8 × 2, 51.6, 44.5, 20.5, 13.9 × 2. HRMS (ESI): m/z calculated for $C_{17}H_{20}NO_5$ $[M + H]^+$: 318.1336, found: 318.1346.

Diethyl 2-methyl-2-(2-oxo-2-(4-(trifluoromethyl)phenyl)ethyl)malonate (33)

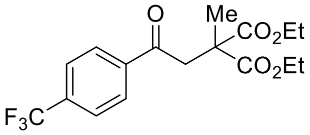

The title compound was prepared according to a modified **GP7** (at 75 °C) and isolated as a pale yellow oil (37.8 mg, 0.105 mmol, 70%). R_f = 0.47 (PE/EtOAc = 10:1). ^{1}H NMR (400 MHz, $CDCl_3$) δ 8.07 (d, J = 8.5 Hz, 2H), 7.73 (d, J = 8.5 Hz, 2H), 4.22 (q, J = 7.1 Hz, 4H), 3.67 (s, 2H), 1.61 (s, 3H), 1.25 (t, J = 7.1 Hz, 6H). ^{19}F NMR (376 MHz, CDCl3) δ -63.04. ^{13}C NMR (101 MHz, $CDCl_3$) δ 195.6, 171.4 × 2, 139.2 (q, J = 1.3 Hz), 134.6 (q, J = 32.8 Hz), 128.3 × 2, 125.7 × 2 (q, J = 4.1 Hz), 123.5 (q, J = 272.6 Hz), 61.7 × 2, 51.6, 44.5, 20.6, 13.9 × 2. HRMS (ESI): m/z calculated for $C_{17}H_{20}F_3O_5$ $[M + H]^+$: 361.1257, found: 361.1261.

Diethyl 2-(2-(4-(methoxycarbonyl)phenyl)-2-oxoethyl)-2-methylmalonate (34)

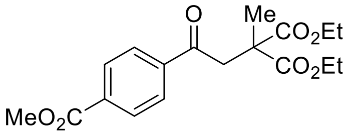

The title compound was prepared according to a modified **GP7** (at 75 °C) and isolated as a pale yellow oil (37.5 mg, 0.107 mmol, 71%). R_f = 0.25 (PE/EtOAc = 10:1). ^{1}H NMR (400 MHz, $CDCl_3$) δ 8.12 (d, J = 8.4 Hz, 2H), 8.01 (d, J = 8.4 Hz, 2H), 4.21 (q, J = 7.1 Hz, 4H), 3.94 (s, 3H), 3.67 (s, 2H), 1.60 (s, 3H), 1.24 (t, J = 7.1 Hz, 6H). ^{13}C NMR (101 MHz, $CDCl_3$) δ 196.1, 171.4 × 2, 166.1, 139.7, 134.0, 129.8 × 2, 127.9 × 2, 61.7 × 2, 52.5, 51.5, 44.6, 20.5, 13.9 × 2. HRMS (ESI): m/z calculated for $C_{18}H_{23}O_7$ $[M + H]^+$: 351.1438, found: 351.1444.

Diethyl 2-methyl-2-(2-oxo-2-(4-(4,4,5,5-tetramethyl-1,3,2-dioxaborolan-2-yl)phenyl)ethyl)malonate (35)

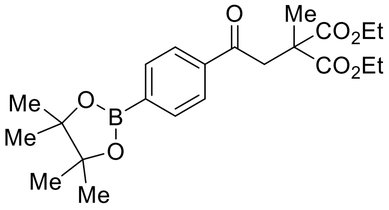

The title compound was prepared according to a modified **GP7** (at 90 °C) and isolated as a pale yellow oil (47.0 mg, 0.112 mmol, 75%). R_f = 0.38 (PE/EtOAc = 10:1). ^{1}H NMR (400 MHz, $CDCl_3$) δ 7.93 – 7.86 (m, 4H), 4.21 (q, J = 7.1 Hz, 4H), 3.67 (s, 2H), 1.60 (s, 3H), 1.35 (s, 12H), 1.23 (d, J = 7.1 Hz, 6H). ^{13}C NMR (101 MHz, $CDCl_3$) δ 196.8, 171.6 × 2, 138.4, 134.9 × 2, 127.3, 126.9 × 2, 84.2 × 2, 61.6 × 2, 51.5, 44.4, 24.8 × 4, 20.5, 13.9 × 2. HRMS (ESI): m/z calculated for $C_{22}H_{32}BO_7$ $[M + H]^+$: 419.2236, found: 419.2239.

Diethyl 2-methyl-2-(2-oxo-2-(*m*-tolyl)ethyl)malonate (36)

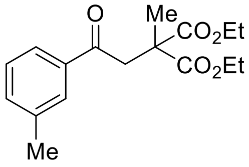

The title compound was prepared according to **GP7** and isolated as a pale yellow oil (37.0 mg, 0.121 mmol, 81%). R_f = 0.47 (PE/EtOAc = 10:1). ^{1}H NMR (400 MHz, $CDCl_3$) δ 7.80 – 7.74 (m, 2H), 7.42 – 7.30 (m, 2H), 4.21 (q, J = 7.1 Hz, 4H), 3.66 (s, 2H), 2.40 (s, 3H), 1.59 (s, 3H), 1.24 (t, J = 7.1 Hz, 6H). ^{13}C NMR (101 MHz, $CDCl_3$) δ 196.6, 171.6 × 2, 138.4, 136.6, 134.1, 128.5, 128.5, 125.2, 61.5 × 2, 51.4, 44.3, 21.3, 20.5, 13.9 × 2. HRMS (ESI): m/z calculated for $C_{17}H_{23}O_5$ $[M + H]^+$: 307.1540,

found: 307.1545.

Diethyl 2-(2-(3-bromophenyl)-2-oxoethyl)-2-methylmalonate (37)

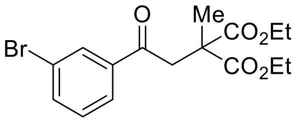

The title compound was prepared according to **GP7** and isolated as a pale yellow oil (41.7 mg, 0.112 mmol, 75%). R_f = 0.49 (PE/EtOAc = 10:1). ^{1}H NMR (400 MHz, $CDCl_3$) δ 8.08 (t, J = 1.8 Hz, 1H), 7.88 (dt, J = 7.8, 1.4 Hz, 1H), 7.71 – 7.66 (m, 1H), 7.34 (t, J = 7.8 Hz, 1H), 4.21 (q, J = 7.1 Hz, 4H), 3.61 (s, 2H), 1.59 (s, 3H), 1.24 (t, J = 7.1 Hz, 6H). ^{13}C NMR (101 MHz, $CDCl_3$) δ 195.2, 171.4 × 2, 138.3, 136.1, 131.1, 130.2, 126.5, 122.9, 61.6 × 2, 51.5, 44.3, 20.5, 13.9 × 2. HRMS (ESI): m/z calculated for $C_{16}H_{20}BrO_5$ [M + H]$^+$: 371.0489, found: 371.0490.

Diethyl 2-methyl-2-(2-oxo-2-(*m*-tolyl)ethyl)malonate (38)

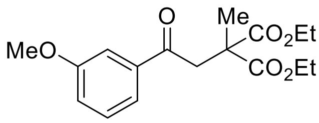

The title compound was prepared according to a modified **GP7** (at 90 °C) and isolated as a pale yellow oil (34.4 mg, 0.107 mmol, 71%). R_f = 0.47 (PE/EtOAc = 10:1). ^{1}H NMR (400 MHz, $CDCl_3$) δ 7.59 – 7.50 (m, 1H), 7.50 – 7.44 (m, 1H), 7.37 (t, J = 7.9 Hz, 1H), 7.15 – 7.04 (m, 1H), 4.21 (q, J = 7.1 Hz, 4H), 3.84 (s, 3H), 3.65 (s, 2H), 1.59 (s, 3H), 1.25 (t, J = 7.1 Hz, 6H). ^{13}C NMR (101 MHz, $CDCl_3$) δ 196.3, 171.6 × 2, 159.8, 138.0, 129.6, 120.7, 120.0, 112.0, 61.6 × 2, 55.4, 51.5, 44.4, 20.5, 13.9 × 2. HRMS (ESI): m/z calculated for $C_{17}H_{23}O_6$ [M + H]$^+$: 323.1489, found: 323.1494.

Diethyl 2-(2-(3-acetamidophenyl)-2-oxoethyl)-2-methylmalonate (39)

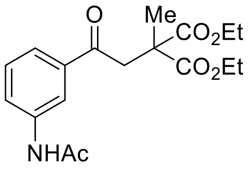

The title compound was prepared according to **GP7** and isolated as a pale yellow oil (41.3 mg, 0.118 mmol, 79%). R_f = 0.59 (PE/EtOAc = 1:1). ^{1}H NMR (400 MHz, $CDCl_3$) δ 8.06 (s, 1H), 7.94 – 7.87 (m, 2H), 7.68 – 7.62 (m, 1H), 7.41 – 7.35 (m, 1H), 4.19 (q, J = 7.1 Hz, 4H), 3.63 (s, 2H), 2.19 (s, 3H), 1.57 (s, 3H), 1.23 (t, J = 7.1 Hz, 6H). ^{13}C NMR (101 MHz, $CDCl_3$) δ 196.1, 171.6 × 2, 169.0, 138.6, 137.1, 129.3, 124.8, 123.6, 118.9, 61.6 × 2, 51.5, 44.3, 24.4, 20.5, 13.9 × 2. HRMS (ESI): m/z calculated for $C_{18}H_{24}NO_6$ [M + H]$^+$: 350.1598, found: 350.1599.

Diethyl 2-(2-(2-fluorophenyl)-2-oxoethyl)-2-methylmalonate (40)

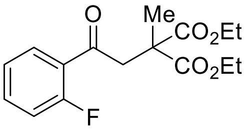

The title compound was prepared according to **GP7** and isolated as a pale yellow solid (32.0 mg, 0.103 mmol, 69%). R_f = 0.49 (PE/EtOAc = 10:1). M.p.: 63.1 – 65.2 °C. ^{1}H NMR (400 MHz, $CDCl_3$) δ 7.85 (td, J = 7.6, 1.9 Hz, 1H), 7.57 – 7.48 (m, 1H), 7.22 (td, J = 7.6, 1.1 Hz, 1H), 7.18 – 7.10 (m, 1H), 4.21 (q, J = 7.1 Hz, 4H), 3.65 (d, J = 3.0 Hz, 2H), 1.60 (s, 3H), 1.25 (t, J = 7.1 Hz, 6H). ^{19}F NMR (377 MHz, $CDCl_3$) δ -108.24. ^{13}C NMR (101 MHz, $CDCl_3$) δ 194.8 (d, J = 4.0 Hz), 171.5 × 2, 161.9 (d, J = 254.9 Hz), 134.8 (d, J = 9.2 Hz), 130.5 (d, J = 2.5 Hz), 125.3 (d, J = 13.2 Hz), 124.5 (d, J = 3.4 Hz), 116.7 (d, J = 23.7 Hz), 61.5 × 2, 51.7 (d, J = 2.4 Hz), 49.0 (d, J = 8.7 Hz), 20.6, 13.9 × 2. HRMS (ESI): m/z calculated for $C_{16}H_{20}FO_5$ [M + H]$^+$: 311.1289, found: 311.1296.

Diethyl 2-(2-(2-methoxyphenyl)-2-oxoethyl)-2-methylmalonate (41)

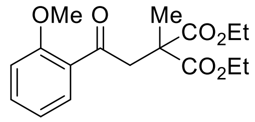

The title compound was prepared according to a modified **GP7** (at 100 °C) and isolated as a pale yellow solid (26.1 mg, 0.081 mmol, 54%). R_f = 0.43 (PE/EtOAc = 10:1). M.p.: 87.6 – 89.0 °C. ^{1}H NMR (400 MHz, $CDCl_3$) *δ* 7.72 (dd, *J* = 7.8, 1.8 Hz, 1H), 7.50 – 7.39 (m, 1H), 7.04 – 6.92 (m, 2H), 4.20 (q, *J* = 7.1 Hz, 4H), 3.93 (s, 3H), 3.68 (s, 2H), 1.57 (s, 3H), 1.24 (t, *J* = 7.1 Hz, 6H). ^{13}C NMR (101 MHz, $CDCl_3$) *δ* 198.2, 171.9 × 2, 158.7, 133.8, 130.4, 127.8, 120.6, 111.5, 61.4 × 2, 55.5, 51.7, 49.5, 20.5, 14.0 × 2. HRMS (ESI): m/z calculated for $C_{17}H_{23}O_6$ [M + H]$^+$: 323.1489, found: 323.1494.

Diethyl 2-(2-(3-bromo-4-methoxyphenyl)-2-oxoethyl)-2-methylmalonate (42)

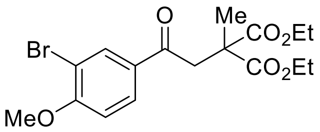

The title compound was prepared according to a modified **GP7** (at 100 °C) and isolated as a pale yellow solid (45.1 mg, 0.112 mmol, 75%). R_f = 0.29 (PE/EtOAc = 10:1). M.p.: 59.1 – 60.6 °C. ^{1}H NMR (400 MHz, $CDCl_3$) *δ* 8.16 (d, *J* = 2.2 Hz, 1H), 7.92 (dd, *J* = 8.7, 2.2 Hz, 1H), 6.93 (d, *J* = 8.7 Hz, 1H), 4.20 (q, *J* = 7.1 Hz, 4H), 3.96 (s, 3H), 3.58 (s, 2H), 1.58 (s, 3H), 1.24 (t, *J* = 7.1 Hz, 6H). ^{13}C NMR (101 MHz, $CDCl_3$) *δ* 194.0, 171.5 × 2, 159.7, 133.5, 130.6, 129.2, 111.8, 111.0, 61.8 × 2, 56.5, 51.5, 43.8, 20.5, 13.9 × 2. HRMS (ESI): m/z calculated for $C_{17}H_{22}BrO_6$ [M + H]$^+$: 401.0594, found: 401.0598.

Diethyl 2-(2-(4-chloro-3-methylphenyl)-2-oxoethyl)-2-methylmalonat (43)

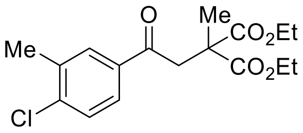

The title compound was prepared according to a modified **GP7** (at 100 °C) and isolated as a pale yellow solid (36.2 mg, 0.106 mmol, 71%). R_f = 0.44 (PE/EtOAc = 10:1). M.p.: 59.2 – 61.2 °C. ^{1}H NMR (400 MHz, $CDCl_3$) *δ* 7.82 (d, *J* = 2.2 Hz, 1H), 7.72 (dd, *J* = 8.4, 2.2 Hz, 1H), 7.42 (d, *J* = 8.3 Hz, 1H), 4.21 (q, *J* = 7.1 Hz, 4H), 3.62 (s, 2H), 2.42 (s, 3H), 1.59 (s, 3H), 1.24 (t, *J* = 7.1 Hz, 6H). ^{13}C NMR (101 MHz, $CDCl_3$) *δ* 195.6, 171.5 × 2, 139.9, 136.6, 135.0, 130.4, 129.3, 126.7, 61.6 × 2, 51.5, 44.2, 20.5, 20.1, 13.9 × 2. HRMS (ESI): m/z calculated for $C_{17}H_{22}ClO_5$ [M + H]$^+$: 341.1150, found: 341.1158.

Diethyl 2-(2-(3,5-difluorophenyl)-2-oxoethyl)-2-methylmalonate (44)

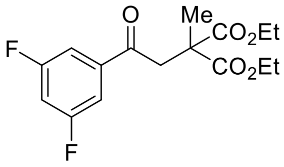

The title compound was prepared according to a modified **GP7** (at 100 °C) and isolated as a pale yellow oil (32.7 mg, 0.100 mmol, 66%). R_f = 0.44 (PE/EtOAc = 10:1). ^{1}H NMR (400 MHz, CDCl3) *δ* 7.52 – 7.40 (m, 2H), 7.06 – 6.97 (m, 1H), 4.21 (q, *J* = 7.1 Hz, 4H), 3.58 (s, 2H), 1.59 (s, 3H), 1.24 (t, *J* = 7.1 Hz, 6H). ^{19}F NMR (376 MHz, $CDCl_3$) *δ* -107.71 (t, *J* = 7.5 Hz). ^{13}C NMR (101 MHz, $CDCl_3$) *δ* 194.1, 171.3 × 2, 164.2 (d, *J* = 11.9 Hz), 161.7 (d, *J* = 11.6 Hz), 139.4 (t, *J* = 7.7 Hz), 111.0 (d, *J* = 26.4 Hz), 111.0 (d, *J* = 11.3 Hz), 108.6 (t, *J* = 25.5 Hz), 61.7 × 2, 51.5, 44.4, 20.5, 13.9 × 2. HRMS (ESI): m/z calculated for $C_{16}H_{19}F_2O_5$ [M + H]$^+$: 329.1195, found: 329.1196.

Diethyl 2-(2-(benzo[*d*][1,3]dioxol-5-yl)-2-oxoethyl)-2-methylmalonate (45)

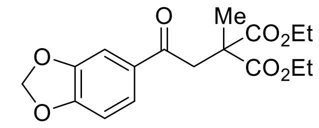

The title compound was prepared according to **GP7** and isolated as a pale yellow oil (35.7 mg, 0.106 mmol, 71%). R_f = 0.29 (PE/EtOAc = 10:1). ^{1}H NMR (400 MHz, $CDCl_3$) δ 7.57 (dd, J = 8.2, 1.7 Hz, 1H), 7.42 (d, J = 1.7 Hz, 1H), 6.84 (d, J = 8.2 Hz, 1H), 6.04 (s, 2H), 4.20 (d, J = 7.1 Hz, 4H), 3.58 (s, 2H), 1.57 (s, 3H), 1.24 (t, J = 7.1 Hz, 6H). ^{13}C NMR (101 MHz, $CDCl_3$) δ 194.5, 171.6 × 2, 151.9, 148.1, 131.5, 124.3, 107.8, 107.7, 101.9, 61.5 × 2, 51.5, 44.0, 20.5, 13.9 × 2. HRMS (ESI): m/z calculated for $C_{17}H_{21}O_7$ [M + H]$^+$: 337.1282, found: 337.1283.

Diethyl 2-methyl-2-(2-(naphthalen-2-yl)-2-oxoethyl)malonate (46)

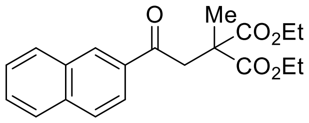

The title compound was prepared according to a modified **GP7** (at 90 °C) and isolated as a pale yellow solid (39.6 mg, 0.116 mmol, 77%). R_f = 0.44 (PE/EtOAc = 10:1). M.p.: 52.4 – 53.7 °C. ^{1}H NMR (400 MHz, $CDCl_3$) δ 8.50 (d, J = 1.2 Hz, 1H), 8.02 (dd, J = 8.7, 1.8 Hz, 1H), 7.97 (d, J = 7.4 Hz, 1H), 7.92 – 7.84 (m, 2H), 7.65 – 7.48 (m, 2H), 4.24 (q, J = 7.1 Hz, 4H), 3.83 (s, 2H), 1.65 (s, 3H), 1.26 (t, J = 7.1 Hz, 6H). ^{13}C NMR (101 MHz, $CDCl_3$) δ 196.4, 171.7 × 2, 135.6, 133.9, 132.4, 129.8, 129.5, 128.6, 128.4, 127.7, 126.8, 123.5, 61.6 × 2, 51.5, 44.3, 20.6, 13.9 × 2. HRMS (ESI): m/z calculated for $C_{20}H_{23}O_5$ [M + H]$^+$: 343.1540, found: 343.1542.

Diethyl 2-methyl-2-(2-oxo-2-(pyridin-3-yl)ethyl)malonate (47)

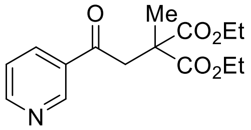

The title compound was prepared according to **GP7** and isolated as a yellow oil (33.5 mg, 0.114 mmol, 76%). R_f = 0.31 (PE/EtOAc = 4:1). ^{1}H NMR (400 MHz, $CDCl_3$) δ 9.19 (s, 1H), 8.80 (s, 1H), 8.22 (d, J = 7.9 Hz, 1H), 7.42 (dd, J = 8.1, 4.5 Hz, 1H), 4.20 (q, J = 7.1 Hz, 4H), 3.64 (s, 2H), 1.60 (s, 3H), 1.24 (t, J = 7.1 Hz, 6H). ^{13}C NMR (101 MHz, $CDCl_3$) δ 195.5, 171.3 × 2, 153.7, 149.4, 135.3 × 3, 61.7 × 2, 51.5, 44.4, 20.6, 13.9 × 2. HRMS (ESI): m/z calculated for $C_{15}H_{20}NO_5$ [M + H]$^+$: 294.1336, found: 294.1337.

Diethyl 2-methyl-2-(2-oxo-2-(thiophen-2-yl)ethyl)malonate (48)

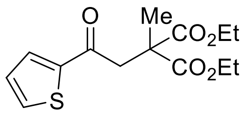

The title compound was prepared according to a modified **GP7** (at 100 °C) and isolated as a pale yellow oil (33.7 mg, 0.111 mmol, 75%). R_f = 0.38 (PE/EtOAc = 10:1). ^{1}H NMR (400 MHz, $CDCl_3$) δ 7.75 (dd, J = 3.7, 1.1 Hz, 1H), 7.65 (dd, J = 4.9, 1.2 Hz, 1H), 7.13 (dd, J = 4.9, 3.7 Hz, 1H), 4.21 (q, J = 7.1 Hz, 4H), 3.59 (s, 2H), 1.59 (s, 3H), 1.25 (t, J = 7.1 Hz, 6H). ^{13}C NMR (101 MHz, $CDCl_3$) δ 189.4, 171.4 × 2, 143.9, 133.9, 132.1, 128.1, 61.6 × 2, 51.6, 44.5, 20.5, 13.9 × 2. HRMS (ESI): m/z calculated for $C_{14}H_{19}SO_5$ [M + H]$^+$: 299.0948, found: 299.0950.

Diethyl 2-methyl-2-(2-oxo-2-(quinolin-3-yl)ethyl)malonate (49)

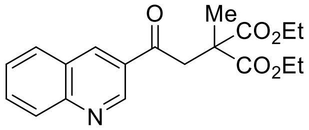

The title compound was prepared according to **GP7** and isolated as a yellow oil (38.2 mg, 0.111 mmol, 74%). R_f = 0.38 (PE/EtOAc = 4:1). ^{1}H NMR (400 MHz, $CDCl_3$) δ 9.42 (s, 1H), 8.74 (d, J = 2.0 Hz, 1H), 8.16 (d, J = 8.5 Hz, 1H), 7.95 (dd, J = 8.0, 1.3 Hz, 1H),

7.87 – 7.80 (m, 1H), 7.69 – 7.59 (m, 1H), 4.24 (q, *J* = 7.1 Hz, 4H), 3.78 (s, 2H), 1.66 (s, 3H), 1.26 (t, *J* = 7.1 Hz, 6H). ^{13}C NMR (101 MHz, $CDCl_3$) *δ* 195.5, 171.4 × 2, 149.8, 148.8, 137.1, 132.1, 129.5, 129.3, 128.8, 127.6, 126.7, 61.7 × 2, 51.6, 44.4, 20.6, 13.9 × 2. HRMS (ESI): m/z calculated for $C_{19}H_{22}NO_5$ [M + H]$^+$: 344.1492, found: 344.1496.

Ethyl (*E*)-2-cyano-2-methyl-4-oxo-6-phenylhex-5-enoate (50)

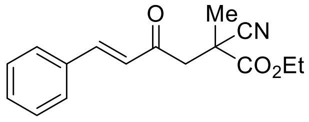

The title compound was prepared according to a modified **GP7** using tertiary alkane (0.15 mmol) and ketone (3.0 equiv) for 24 h and isolated as a pale yellow oil (28.0 mg, 0.103 mmol, 69%). R_f = 0.33 (PE/EtOAc = 4:1). ^{1}H NMR (400 MHz, $CDCl_3$) *δ* 7.58 (d, *J* = 16.3 Hz, 1H), 7.55 – 7.50 (m, 2H), 7.43 – 7.36 (m, 3H), 6.72 (d, *J* = 16.3 Hz, 1H), 4.36 – 4.23 (m, 2H), 3.44 (d, *J* = 17.9 Hz, 1H), 3.26 (d, *J* = 17.9 Hz, 1H), 1.69 (s, 3H), 1.34 (t, *J* = 7.1 Hz, 3H). ^{13}C NMR (101 MHz, $CDCl_3$) *δ* 194.3, 169.2, 144.4, 133.9, 131.1, 129.1 × 2, 128.6 × 2, 124.5, 119.9, 63.1, 48.0, 39.9, 23.8, 14.0. HRMS (ESI): m/z calculated for $C_{16}H_{18}NO_3$ [M + H]$^+$: 272.1281, found: 272.1285.

Ethyl 2-cyano-2,3-dimethyl-4-oxo-4-phenylbutanoate (51)

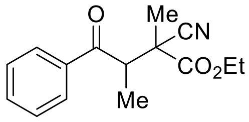

The title compound was prepared according to a modified **GP7** with 40 mol% **A6** at 90 °C for 18 h and isolated as a pale yellow solid (22.9 mg, 0.088 mmol, 59%). R_f = 0.35 (PE/EtOAc = 4:1). M.p.: 67.0 – 69.8 °C. One isomer **51A** (11.7 mg): ^{1}H NMR (400 MHz, $CDCl_3$) *δ* 7.97 – 7.91 (m, 2H), 7.66 – 7.59 (m, 1H), 7.52 – 7.46 (m, 2H), 4.23 (qd, *J* = 7.1, 1.1Hz, 2H), 4.10 (q, *J* = 7.5 Hz, 1H), 1.70 (s, 3H), 1.44 (d, *J* = 7.5 Hz, 3H), 1.31 (t, *J* = 7.1 Hz, 3H). ^{13}C NMR (101 MHz, $CDCl_3$) *δ* 199.2, 169.0, 135.7, 133.9, 129.1 × 2, 128.5 × 2, 119.1, 63.3, 46.3, 45.7, 20.9, 15.4, 14.1. HRMS (ESI): m/z calculated for $C_{15}H_{18}NO_3$ [M + H]$^+$: 260.1281, found: 260.1284. Another isomer **51B** (11.2 mg): ^{1}H NMR (400 MHz, $CDCl_3$) *δ* 7.92 (dd, *J* = 8.4, 1.3 Hz, 2H), 7.66 – 7.56 (m, 1H), 7.55 – 7.44 (m, 2H), 4.23 (qd, *J* = 7.2, 1.1 Hz, 2H), 3.92 (q, *J* = 7.6 Hz, 1H), 1.74 (s, 3H), 1.47 (d, *J* = 7.6 Hz, 3H), 1.28 (t, *J* = 7.2 Hz, 3H). ^{13}C NMR (101 MHz, $CDCl_3$) *δ* 200.5, 170.1, 134.9, 133.7, 129.0 × 2, 128.6 × 2, 118.9, 63.1, 47.4, 46.0, 23.0, 14.0, 13.9. HRMS (ESI): m/z calculated for $C_{15}H_{18}NO_3$ [M + H]$^+$: 260.1281, found: 260.1286.

Ethyl 3-benzoyl-2-cyano-2-methylpentanoate (52)

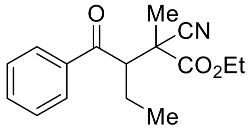

The title compound was prepared according to a modified **GP7** with 40 mol% **A6** and 7 equiv DTBP at 60 °C for 24 h and isolated as a pale yellow oil (18.4 mg, 0.067 mmol, 45%). One isomer **52A** (9.7 mg). R_f = 0.40 (PE/EtOAc = 10:1). ^{1}H NMR (400 MHz, $CDCl_3$) *δ* 8.08 – 7.93 (m, 2H), 7.66 – 7.59 (m, 1H), 7.55 – 7.46 (m, 2H), 4.19 (q, *J* = 7.1 Hz, 2H), 4.04 (dd, *J* = 9.9, 4.4 Hz, 1H), 2.14 – 2.01 (m, 1H), 1.84 – 1.72 (m, 1H), 1.59 (s, 3H), 1.28 (t, *J* = 7.1 Hz, 3H), 0.90 (t, *J* = 7.5 Hz, 3H). ^{13}C NMR (101 MHz, $CDCl_3$) *δ* 199.9, 168.8, 137.9, 133.8, 128.9 × 2, 128.3 × 2, 118.7, 63.2, 50.3, 46.6, 24.1, 21.2, 13.8, 12.2. HRMS (ESI): m/z calculated for $C_{16}H_{20}NO_3$ [M + H]$^+$: 274.1438, found: 274.1439. Another isomer **52B** (8.7 mg). R_f = 0.21 (PE/EtOAc = 10:1). ^{1}H NMR (400 MHz, $CDCl_3$) *δ* 8.03 – 7.86 (m, 2H), 7.68 – 7.53 (m, 1H), 7.46 (dd, *J* = 8.4, 7.0 Hz, 2H), 4.11 (qd, *J* = 7.2, 3.2 Hz, 2H), 3.78 (dd, *J* = 7.4, 6.0 Hz, 1H), 2.00 – 1.85 (m, 2H), 1.72 (s, 3H), 1.17 (t, *J* = 7.2 Hz, 3H), 0.92 (t, *J* = 7.5 Hz,

3H). ^{13}C NMR (101 MHz, $CDCl_3$) δ 201.3, 169.4, 137.1, 133.5, 128.7 × 2, 128.3 × 2, 118.9, 62.9, 52.0, 46.1, 23.2, 22.6, 13.7, 12.7. HRMS (ESI): m/z calculated for $C_{16}H_{20}NO_3$ [M + H]$^+$: 274.1438, found: 274.1446.

Ethyl 2-cyano-2-(1-oxo-1,2,3,4-tetrahydronaphthalen-2-yl)propanoate (53)

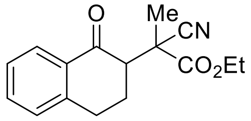

The title compound was prepared according to a modified **GP7** with 40 mol% **A6** at 90 °C for 24 h and isolated as a white oil (15.9 mg, 0.059 mmol, 39%). One isomer **53A** (4.2 mg). R_f = 0.32 (PE/EtOAc = 10:1). ^1H NMR (400 MHz, $CDCl_3$) δ 7.99 (dd, *J* = 7.9, 1.5 Hz, 1H), 7.52 (td, *J* = 7.5, 1.5 Hz, 1H), 7.36 – 7.27 (m, 2H), 4.43 – 4.32 (m, 2H), 3.52 (dd, *J* = 14.2, 4.1 Hz, 1H), 3.27 – 3.04 (m, 2H), 2.45 – 2.35 (m, 1H), 2.23 – 2.09 (m, 1H), 1.65 (s, 3H), 1.33 (t, *J* = 7.1 Hz, 3H). ^{13}C NMR (101 MHz, $CDCl_3$) δ 194.8, 168.0, 143.7, 134.3, 132.0, 128.9, 127.8, 127.1, 119.9, 63.1, 53.2, 44.2, 29.5, 25.9, 17.7, 14.0. HRMS (ESI): m/z calculated for $C_{16}H_{18}NO_3$ [M + H]$^+$: 272.1281, found: 272.1283. Another isomer **53B** (11.7 mg). R_f = 0.17 (PE/EtOAc = 10:1). ^1H NMR (400 MHz, $CDCl_3$) δ 8.02 (dd, *J* = 8.0, 1.4 Hz, 1H), 7.51 (td, *J* = 7.5, 1.5 Hz, 1H), 7.36 – 7.30 (m, 1H), 7.28 – 7.26 (m, 1H), 4.42 – 4.33 (m, 2H), 3.32 (dd, *J* = 14.4, 4.3 Hz, 1H), 3.21 – 3.11 (m, 2H), 2.43 – 2.37 (m, 1H), 2.22 – 2.11 (m, 1H), 1.67 (s, 3H), 1.41 (t, *J* = 7.1 Hz, 3H). ^{13}C NMR (101 MHz, $CDCl_3$) δ 195.4, 170.2, 143.4, 134.3, 131.7, 128.8, 128.0, 127.1, 118.8, 63.0, 54.1, 44.4, 29.2, 24.6, 22.2, 14.1. HRMS (ESI): m/z calculated for $C_{16}H_{18}NO_3$ [M + H]$^+$: 272.1281, found: 272.1287.

Ethyl 2-cyano-2-methyl-4-oxoheptanoate (54)

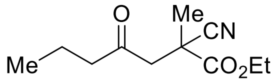

The title compound was prepared according to a modified **GP7** with 40 mol% **A6**, tertiary alkane (0.15 mmol) and ketone (2.0 equiv) at 70 ºC for 18 h and isolated as a pale yellow oil (10.1 mg, 0.048 mmol, 32%). R_f = 0.28 (PE/EtOAc = 10:1). ^1H NMR (400 MHz, $CDCl_3$) δ 4.27 (q, *J* = 7.1 Hz, 2H), 2.73 – 2.52 (m, 2H), 2.47 (q, *J* = 7.3 Hz, 2H), 2.31 – 2.18 (m, 1H), 2.14 – 2.01 (m, 1H), 1.62 (s, 3H), 1.13 (t, *J* = 7.1 Hz, 3H), 1.07 (t, *J* = 7.3 Hz, 3H). ^{13}C NMR (101 MHz, $CDCl_3$) δ 208.8, 169.1, 119.8, 63.1, 43.3, 38.0, 36.2, 31.8, 23.7, 14.2, 7.9. HRMS (ESI): m/z calculated for $C_{11}H_{18}NO_3$ [M + H]$^+$: 212.1281, found: 212.1285.

Ethyl 2-cyano-2-methyl-4-oxo-7-phenylheptanoate (55)

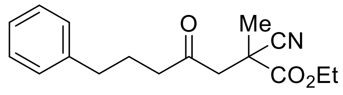

The title compound was prepared according to modified **GP7** with 40 mol% **A6**, tertiary alkane (0.15 mmol) and ketone (2.0 equiv) at 70 ºC for 18 h and isolated as a pale yellow oil (15.1 mg, 0.053 mmol, 35%). R_f = 0.17 (PE/EtOAc = 10:1). ^1H NMR (400 MHz, $CDCl_3$) δ 7.33 – 7.27 (m, 2H), 7.24 – 7.19 (m, 1H), 7.19 – 7.13 (m, 2H), 4.33 – 4.24 (m, 2H), 3.13 (d, *J* = 18.0 Hz, 1H), 2.92 (d, *J* = 18.0 Hz, 1H), 2.63 (t, *J* = 7.5 Hz, 2H), 2.43 (t, *J* = 7.4 Hz, 2H), 1.99 – 1.89 (m, 2H), 1.61 (s, 3H), 1.35 (t, *J* = 7.2 Hz, 3H). ^{13}C NMR (101 MHz, $CDCl_3$) δ 205.2, 169.2, 141.3, 128.61 × 2, 128.59 × 2, 126.2, 119.8, 63.2, 49.8, 41.3, 39.7, 34.9, 25.0, 23.8, 14.1. HRMS (ESI): m/z calculated for $C_{17}H_{22}NO_3$ [M + H]$^+$: 288.1594, found: 288.1595.

(1*R*,2*S*,5*S*)-2-Isopropyl-5-methylcyclohexyl 4-(3-(ethoxycarbonyl)-3-methyl-4-oxopentanoyl)benzoate (56)

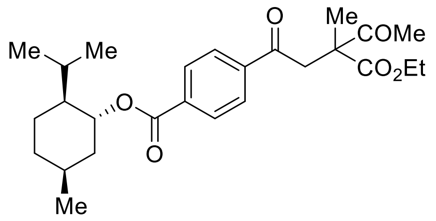

The title compound was prepared according to **GP7** and isolated as a pale yellow oil (57.9 mg, 0.130 mmol, 87%). R_f = 0.54 (PE/EtOAc = 4:1). ^{1}H NMR (400 MHz, $CDCl_3$) *δ* 8.11 (d, *J* = 8.4 Hz, 2H), 8.00 (d, *J* = 8.4 Hz, 2H), 4.94 (td, *J* = 10.9, 4.4 Hz, 1H), 4.20 (q, *J* = 7.1 Hz, 2H), 3.73 – 3.55 (m, 2H), 2.33 (s, 3H), 2.16 – 2.05 (m, 1H), 1.92 (td, *J* = 7.0, 2.7 Hz, 1H), 1.71 (t, *J* = 2.3 Hz, 2H), 1.57 – 1.49 (m, 5H), 1.23 (t, *J* = 7.1 Hz, 3H), 1.17 – 1.05 (m, 2H), 0.96 – 0.88 (m, 7H), 0.78 (d, *J* = 6.9 Hz, 3H). ^{13}C NMR (101 MHz, $CDCl_3$) *δ* 205.5, 196.7, 172.1, 165.1, 139.4, 134.7, 129.8 × 2, 127.9 × 2, 75.4, 61.6, 57.2, 47.1, 44.8, 40.8, 34.2, 31.4, 26.5 × 2, 23.5, 22.0, 20.8, 20.7, 16.4, 13.9. HRMS (ESI): m/z calculated for $C_{26}H_{37}O_6$ [M + H]$^+$: 445.2585, found: 445.2586.

(3a*R*,5*R*,6*S*,6a*R*)-5-((*R*)-2,2-Dimethyl-1,3-dioxolan-4-yl)-2,2-dimethyltetrahydrofuro[2,3-*d*][1,3]dioxol-6-yl 4-(3-(ethoxycarbonyl)-3-methyl-4-oxopentanoyl)benzoate (57)

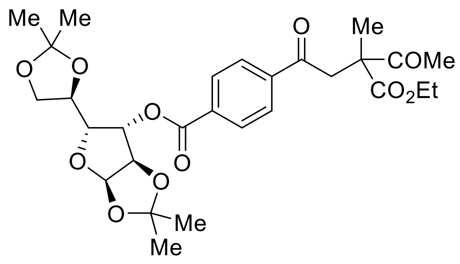

The title compound was prepared according to **GP7** and isolated as a pale yellow oil (74.1 mg, 0.135 mmol, 90%). R_f = 0.77 (PE/EtOAc = 1:1). ^{1}H NMR (400 MHz, $CDCl_3$) *δ* 8.09 (d, *J* = 8.5 Hz, 2H), 8.01 (d, *J* = 8.5 Hz, 2H), 5.95 (d, *J* = 3.7 Hz, 1H), 5.50 (d, *J* = 2.8 Hz, 1H), 4.64 (d, *J* = 3.8 Hz, 1H), 4.37 – 4.27 (m, 2H), 4.20 (q, *J* = 7.1 Hz, 2H), 4.15 – 4.03 (m, 2H), 3.70 – 3.54 (m, 2H), 2.33 (s, 3H), 1.57 (s, 3H), 1.55 (s, 3H), 1.40 (s, 3H), 1.31 (s, 3H), 1.27 – 1.19 (m, 6H). ^{13}C NMR (101 MHz, $CDCl_3$) *δ* 205.7, 196.7, 172.2, 164.4, 140.2, 133.5, 130.1 × 2, 128.2 × 2, 112.5, 109.6, 105.2, 83.4, 80.0, 77.4, 72.6, 67.5, 61.8, 57.4, 45.0, 27.0, 26.8, 26.7, 26.3, 25.3, 20.9, 14.1. HRMS (ESI): m/z calculated for $C_{28}H_{37}O_{11}$ [M + H]$^+$: 549.2330, found: 549.2336.

(*E*)-3,7-Dimethylocta-2,6-dien-1-yl-4-(3-(ethoxycarbonyl)-3-methyl-4-oxopentanoyl)benzoate (58)

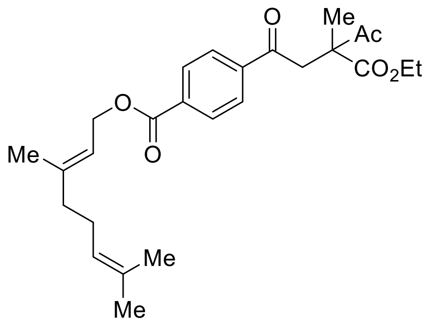

The title compound was prepared according to **GP7** and isolated as a pale yellow oil (54.8 mg, 0.124 mmol, 83%). R_f = 0.5 (PE/EtOAc = 4:1). ^{1}H NMR (400 MHz, $CDCl_3$) *δ* 8.11 (d, *J* = 8.4 Hz, 2H), 7.99 (d, *J* = 8.4 Hz, 2H), 5.48 (td, *J* = 7.3, 1.5 Hz, 1H), 5.12 – 5.08 (m, 1H), 4.82 (dd, *J* = 7.3, 1.1 Hz, 2H), 4.20 (q, *J* = 7.1 Hz, 2H), 3.73 – 3.54 (m, 2H), 2.33 (s, 3H), 2.22 – 2.14 (m, 2H), 2.14 – 2.06 (m, 2H), 1.79 (d, *J* = 1.3 Hz, 3H), 1.65 (d, *J* = 1.3 Hz, 3H), 1.58 (d, *J* = 8.9 Hz, 6H), 1.23 (t, *J* = 7.1 Hz, 3H). ^{13}C NMR (101 MHz, $CDCl_3$) *δ* 205.5, 196.7, 172.1, 165.6, 143.2, 139.5, 134.4, 132.2, 129.8 × 2, 127.89 × 2, 123.4, 118.8, 62.0, 61.6, 57.2, 44.8, 33.2, 26.6, 26.5, 25.7, 23.5, 20.7, 17.6, 13.9. HRMS (ESI): m/z calculated for $C_{26}H_{35}O_6$ [M + H]$^+$: 443.2428, found: 443.2431.

Ethyl 2-acetyl-4-(6-(*tert*-butyl)-1,1-dimethyl-2,3-dihydro-1*H*-inden-4-yl)-2-methyl-4-oxobutanoate (59)

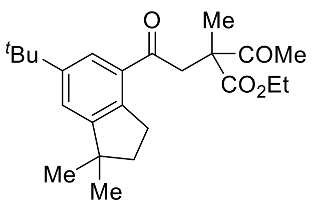

The title compound was prepared according to **GP7** and isolated as a colorless oil (40.6 mg, 0.105 mmol, 70%). R_f = 0.57 (PE/EtOAc = 10:1). ^1H NMR (400 MHz, $CDCl_3$) δ 7.71 (d, J = 1.8 Hz, 1H), 7.35 (d, J = 1.8 Hz, 1H), 4.21 (q, J = 7.1 Hz, 2H), 3.71 – 3.56 (m, 2H), 3.14 (t, J = 7.2 Hz, 2H), 2.34 (s, 3H), 1.92 (t, J = 7.2 Hz, 2H), 1.56 (s, 3H), 1.36 (s, 9H), 1.27 – 1.23 (m, 9H). ^{13}C NMR (101 MHz, $CDCl_3$) δ 205.8, 199.0, 172.5, 154.4, 150.0, 141.1, 133.0, 123.8, 123.5, 61.5, 57.3, 46.4, 43.4, 41.3, 34.7, 31.5 × 3, 30.9, 28.7 × 2, 26.5, 20.7, 13.9. HRMS (ESI): m/z calculated for $C_{24}H_{35}O_4$ $[M + H]^+$: 387.2530, found: 387.2534.

Diethyl (*S*)-2-(2-(4-((1-ethoxy-1-oxopropan-2-yl)carbamoyl)phenyl)-2-oxoethyl)-2-methylmalonate (60)

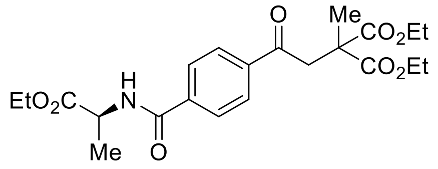

The title compound was prepared according to **GP7** and isolated as a pale yellow oil (54.4 mg, 0.125 mmol, 83%). R_f = 0.58 (PE/EtOAc = 1:1). ^1H NMR (400 MHz, $CDCl_3$) δ 8.00 (d, J = 8.4 Hz, 2H), 7.88 (d, J = 8.4 Hz, 2H), 6.91 (d, J = 7.2 Hz, 1H), 4.76 (p, J = 7.2 Hz, 1H), 4.28 – 4.13 (m, 6H), 3.65 (s, 2H), 1.59 (s, 3H), 1.52 (d, J = 7.1 Hz, 3H), 1.30 (t, J = 7.2 Hz, 3H), 1.23 (t, J = 7.1 Hz, 6H). ^{13}C NMR (101 MHz, $CDCl_3$) δ 195.9, 173.0, 171.4 × 2, 165.7, 138.8, 137.9, 128.2 × 2, 127.4 × 2, 61.7 × 2, 61.6, 51.5, 48.7, 44.4, 20.5, 18.5, 14.1, 13.9 × 2. HRMS (ESI): m/z calculated for $C_{22}H_{30}NO_8$ $[M + H]^+$: 436.1966, found: 436.1970.

Diethyl (*S*)-2-(2-(4-((1-ethoxy-1-oxo-3-phenylpropan-2-yl)carbamoyl)phenyl)-2-oxoethyl)-2-methylmalonate (61)

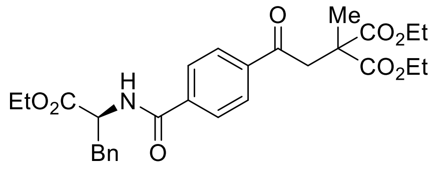

The title compound was prepared according to **GP7** and isolated as a pale yellow oil (63.4 mg, 0.124 mmol, 83%). R_f = 0.83 (PE/EtOAc = 1:1). ^1H NMR (400 MHz, $CDCl_3$) δ 7.99 (d, J = 8.4 Hz, 2H), 7.79 (d, J = 8.4 Hz, 2H), 7.33 – 7.22 (m, 3H), 7.13 (dd, J = 7.7, 1.7 Hz, 2H), 6.70 (d, J = 7.7 Hz, 1H), 5.05 (dt, J = 7.6, 5.7 Hz, 1H), 4.27 – 4.15 (m, 6H), 3.65 (s, 2H), 3.36 – 3.16 (m, 2H), 1.60 (s, 3H), 1.31 – 1.19 (m, 9H). ^{13}C NMR (101 MHz, $CDCl_3$) δ 195.9, 171.4 × 3, 165.7, 138.9, 138.0, 135.7, 129.3 × 2, 128.6 × 2, 128.2 × 2, 127.3 × 2, 127.2, 61.8, 61.6 × 2, 53.6, 51.5, 44.4, 37.7, 20.5, 14.1, 13.9 × 2. HRMS (ESI): m/z calculated for $C_{28}H_{34}NO_8$ $[M + H]^+$: 512.2279, found: 512.2288.

Diethyl (*S*)-2-(2-(4-((1-methoxy-4-methyl-1-oxopentan-2-yl)carbamoyl)phenyl)-2-oxoethyl)-2-methylmalonate (62)

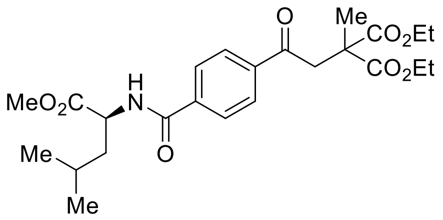

The title compound was prepared according to **GP7** and isolated as a pale yellow oil (58.5 mg, 0.126 mmol, 84%, *ee* > 99%). R_f = 0.43 (PE/EtOAc = 1:1). ^1H NMR (400 MHz, $CDCl_3$) δ 7.99 (d, J = 8.4 Hz, 2H), 7.86 (d, J = 8.4 Hz, 2H), 6.76 (d, J = 8.3 Hz, 1H), 4.84 (td, J = 8.5, 4.2 Hz, 1H), 4.20 (q, J = 7.1 Hz, 4H), 3.76 (s, 3H), 3.65 (s, 2H), 1.80 – 1.64 (m, 3H), 1.60 (s, 3H), 1.24 (t, J = 7.1 Hz, 6H), 0.97 (t, J = 6.1 Hz, 6H). ^{13}C NMR (101 MHz, $CDCl_3$) δ 195.9, 173.5, 171.4 × 2, 166.0, 138.8, 137.8, 128.2 × 2, 127.4 × 2, 61.6 × 2, 52.5, 51.5, 51.2, 44.4, 41.6, 24.9, 22.8, 21.9, 20.5, 13.9 × 2. HRMS (ESI): m/z

calculated for $C_{24}H_{34}NO_8$ [M + H]$^+$: 464.2279, found: 464.2280.

HPLC (Chiralcel AD-H): hexane/isopropanol = 80:20, 1.0 mL/minute, λ = 254 nm, t^1 = 22.460 min, t^2 = 35.576 min, *ee* >99%.

HPLC spectrum of racemic 62

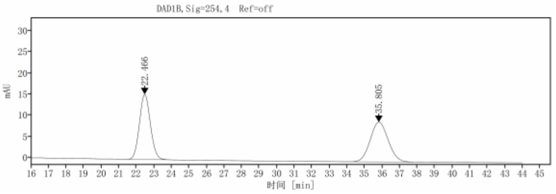

**Sorted By:**	Signal
**Dilution:**	1.0000
**Multiplier:**	1.0000
**Sample Amount:**	1.00000 [ng/ul] (not used in calc.)

Use Multiplier & Dilution Factor with ISTDs

Signal2: DAD1 B, Sig=254,4 Ref=off

Peak	RetTime [min]	Type	Width [min]	Area [mAU*s]	Height [mAU]	Area %
1	22.466	MM m	2.72	670.30	15.40	50.00
	35.805	MM m	3.68	670.29	9.41	50.00
			Totals :	1340.59		100.00

HPLC spectrum of enantio-enriched 62

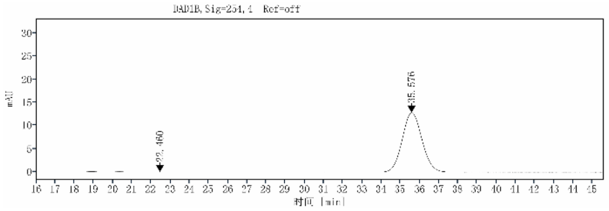

**Sorted By:**	Signal
**Dilution:**	1.0000
**Multiplier:**	1.0000
**Sample Amount:**	1.00000 [ng/ul] (not used in calc.)

Use Multiplier & Dilution Factor with ISTDs

Signal2: DAD1 B, Sig=254,4 Ref=off

Peak	RetTime [min]	Type	Width [min]	Area [mAU*s]	Height [mAU]	Area %
1	22.460	MM m	0.92	0.27	0.01	0.03
	35.576	BB	3.57	911.90	12.85	99.97
			Totals :	912.17		100.00

Diethyl (*R*)-2-methyl-2-(2-oxocyclopentyl)malonate (63)

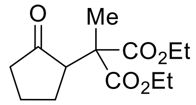

The title compound was prepared according to **GP8** and isolated as a pale yellow oil (10.6 mg, 0.041 mmol, 28%). R_f = 0.38 (PE/EtOAc = 10:1). ^{1}H NMR (400 MHz, $CDCl_3$) δ 4.28 – 4.14 (m, 4H), 2.77 – 2.68 (m, 1H), 2.33 – 2.23 (m, 2H), 2.24 – 2.14 (m, 1H), 2.11 – 2.00 (m, 1H), 1.87 – 1.69 (m, 2H), 1.51 (s, 3H), 1.27 (td, *J* = 7.2, 0.7 Hz, 6H). ^{13}C NMR (101 MHz, $CDCl_3$) δ 216.9, 171.8, 170.9, 61.73, 61.69, 55.6, 55.0, 38.3, 26.5, 20.5, 19.8, 14.13, 14.08. HRMS (ESI): m/z calculated for $C_{13}H_{21}O_5$ [M + H]$^+$: 257.1384, found: 257.1385.

Diethyl (*E*)-2-methyl-2-(4-oxo-4-phenylbut-2-en-1-yl)malonate (64)

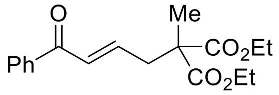

The title compound was prepared according to **GP8** and isolated as a pale yellow oil (28.6 mg, 0.090 mmol, 60%). R_f = 0.43 (PE/EtOAc = 10:1). ^{1}H NMR (400 MHz, $CDCl_3$) δ 7.94 – 7.86 (m, 2H), 7.59 – 7.53 (m, 1H), 7.50 – 7.43 (m, 2H), 6.95 – 6.87 (m, 2H), 4.20 (q, *J* = 7.1 Hz, 4H), 2.91 – 2.83 (m, 2H), 1.46 (s, 3H), 1.25 (t, *J* = 7.1 Hz, 6H). ^{13}C NMR (101 MHz, $CDCl_3$) δ 190.5, 171.5 × 2, 143.3, 137.7, 133.0, 129.6, 128.7 × 4, 61.7 × 2, 53.5, 39.1, 20.3, 14.2 × 2. HRMS (ESI): m/z calculated for $C_{18}H_{23}O_5$ [M + H]$^+$: 319.1540, found: 319.1547.

Diethyl 2-methyl-2-(4-oxocyclohex-2-en-1-yl)malonate (65)

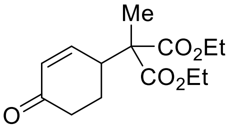

The title compound was prepared according to a modified **GP8** using 3.0 equiv enol and isolated as a pale yellow oil (21.6 mg, 0.081 mmol, 54%). R_f = 0.31 (PE/EtOAc = 10:1). ^{1}H NMR (400 MHz, $CDCl_3$) δ 6.91 (dt, *J* = 10.5, 2.0 Hz, 1H), 6.02 (ddd, *J* = 10.5, 2.9, 1.1 Hz, 1H), 4.28 – 4.14 (m, 4H), 3.26 (ddt, *J* = 11.7, 4.6, 2.5 Hz, 1H), 2.50 – 2.39 (m, 1H), 2.45 – 2.36 (m, 1H), 2.02 – 1.95 (m, 1H), 1.83 – 1.73 (m, 1H), 1.41 (s, 3H), 1.25 (q, *J* = 7.1 Hz, 6H). ^{1}H NMR (101 MHz, $CDCl_3$) δ 199.0, 171.0, 170.8,

151.4, 130.0, 61.9, 61.8, 56.5, 41.2, 37.6, 24.7, 17.5, 14.2, 14.2. HRMS (ESI): m/z calculated for $C_{14}H_{21}O_5$ $[M + H]^+$: 323.1853, found: 323.1860.

Diethyl 2-methyl-2-((6*S*)-3-methyl-4-oxo-6-(prop-1-en-2-yl)cyclohex-2-en-1-yl)malonate (66)

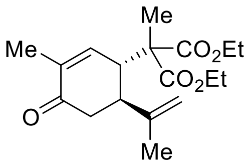

The title compound was prepared according to **GP8** and isolated as a pale yellow oil (31.2 mg, 0.097 mmol, 65%). The stereochemistry of compound **66** was established by NOESY and HMBC analyses. R_f = 0.53 (PE/EtOAc = 10:1). ^1H NMR (400 MHz, $CDCl_3$) *δ* 6.45 (d, *J* = 2.3 Hz, 1H), 4.73 (s, 1H), 4.70 (s, 1H), 4.17 (dtd, *J* = 13.7, 7.1, 3.3 Hz, 3H), 4.06 – 3.93 (m, 1H), 3.52 – 3.47 (m, 1H), 2.77 – 2.70 (m, 1H), 2.50 – 2.36 (m, 2H), 1.76 (s, 3H), 1.68 (s, 3H), 1.41 (s, 3H), 1.26 – 1.19 (m, 6H). ^{13}C NMR (101 MHz, $CDCl_3$) *δ* 198.7, 170.9, 170.4, 145.7, 144.4, 136.3, 113.3, 61.6, 61.4, 57.2, 43.4, 42.5, 42.0, 19.7, 17.0, 15.9, 13.9, 13.7. HRMS (ESI): m/z calculated for $C_{18}H_{27}O_5$ $[M + H]^+$: 323.1853, found: 323.1860.

Diethyl 2-methyl-2-(5-methyl-4-oxocyclohex-2-en-1-yl)malonate (67)

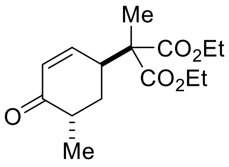

The title compound was prepared according to **GP8** and isolated as a pale yellow oil (17.9 mg, 0.063 mmol, 42%). R_f = 0.41 (PE/EtOAc = 10:1). ^1H NMR (400 MHz, $CDCl_3$) *δ* 6.87 (dd, *J* = 10.4, 2.3 Hz, 1H), 5.96 (dd, *J* = 10.4, 2.8 Hz, 1H), 4.64 – 3.97 (m, 4H), 3.38 – 3.33 (m, 1H), 2.60 – 2.53 (m, 1H), 1.97 – 1.89 (m, 1H), 1.80 – 1.70 (m, 1H), 1.42 (s, 3H), 1.26 (dt, *J* = 9.1, 6.9 Hz, 6H), 1.19 (d, *J* = 7.3 Hz, 3H). ^{13}C NMR (101 MHz, $CDCl_3$) *δ* 202.1, 170.8 × 2, 149.9, 128.6, 61.69, 61.66, 56.3, 39.7, 36.8, 30.6, 17.4, 15.8, 14.03, 13.99. HRMS (ESI): m/z calculated for $C_{15}H_{22}O_5$ $[M + H]^+$: 283.1540, found: 283.1535.

9. References

[1] S. Munnuri, J. R. Falck, *J. Am. Chem. Soc*. **2022**,*144*, 17989−17998.

[2] M.-Q. Tian, Z.-Y Shen, X. Zhao, P. J. Walsh, X.-H. Hu, *Angew. Chem. Int. Ed.* **2021**, *60*, 9706–9711.

[3] Y. Kamei, Y. Seino, Y. Yamaguchi, T. Yoshino, S. Maeda, M. Kojima, S. Matsunaga, *Nat. Commun.* **2021**, *12*, 966.

[3] M.-Q. Tian, C. Wang, X.-H. Hu, T.-P. Loh, *Org. Lett.* **2019**, *21*, 1607–1611.

[4] Q.-C. Shan, L.-M. Hu, W. Qin, X.-H. Hu, *Org. Lett*. **2021**, *23*, 6041−6045.

[5] X. Sheng, J. Zhang, H. Yang, G. Jiang, *Org. Lett*. **2017**, *19*, 2618−2621.

[6] V. Desrosiers, S. M. Knight, F.-G. Fontaine, *ACS Catal*. **2022**, *12*, 13609−13618.

[7] M. G. Siriboe, D. A. Vargas, R. Fasan, *J. Org. Chem*. **2023**,*88*, 7630−7640.

[8] J. Duran, J. Mateos, A. Moyano, X. Companyó, *Chem. Sci*. **2023**, *14*, 7147–7153.

[9] K. F. Szabo, K. Goliszewska, J. Szurmak, K. Rybicka-Jasinska, D. Gryko. *Org. Lett.* **2022**, *24*, 8120−8124.

[10] X. Zhao, F. Yang, S.-Y. Zou, Q.-Q. Zhou, Z.-S. Chen, K. Ji, *ACS. Catal*. **2022**, *12*, 1732−1741.

[11] M. Rössle, T. Werner, W. Frey, J. Christoffers, *Eur. J. Org. Chem.* **2005**, *23*, 5031–5038.

10. NMR spectra of the starting materials

^{1}H and ^{13}C NMR spectra for compound 1aaf

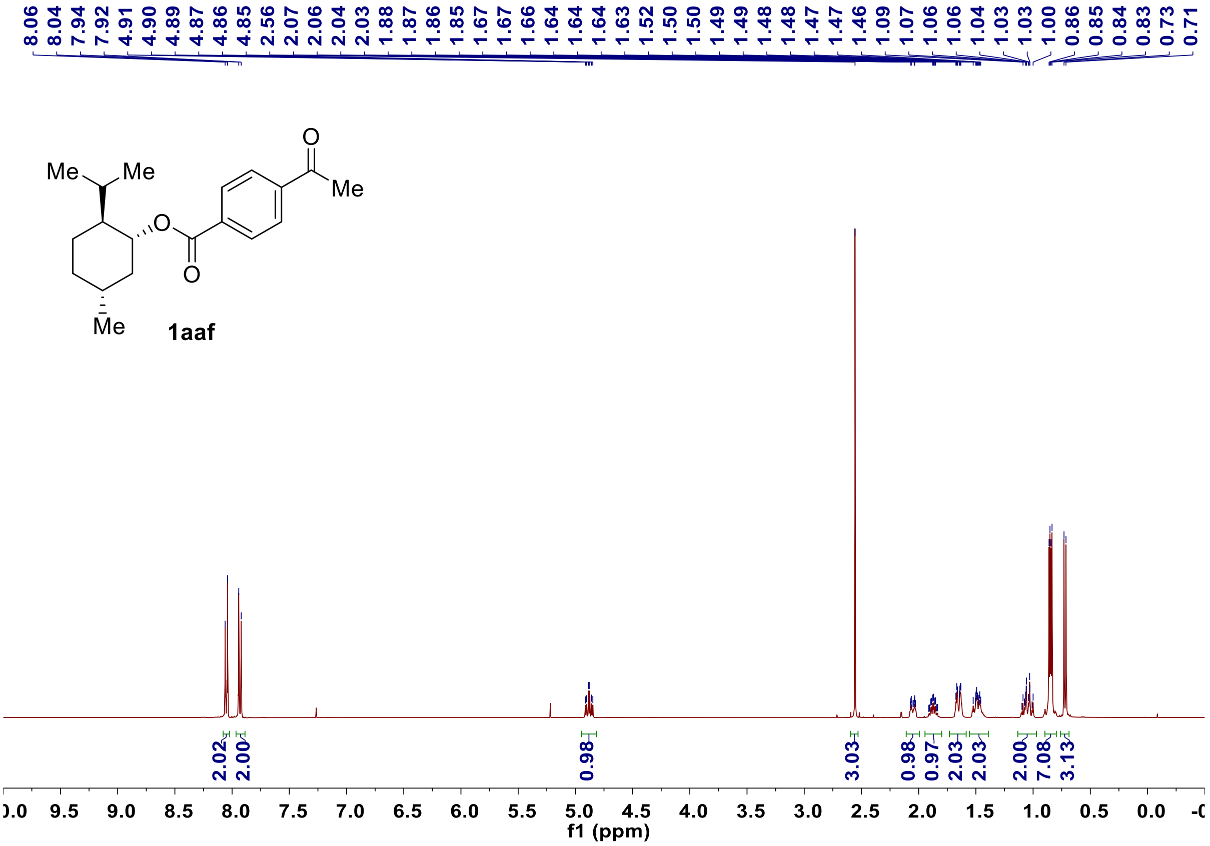

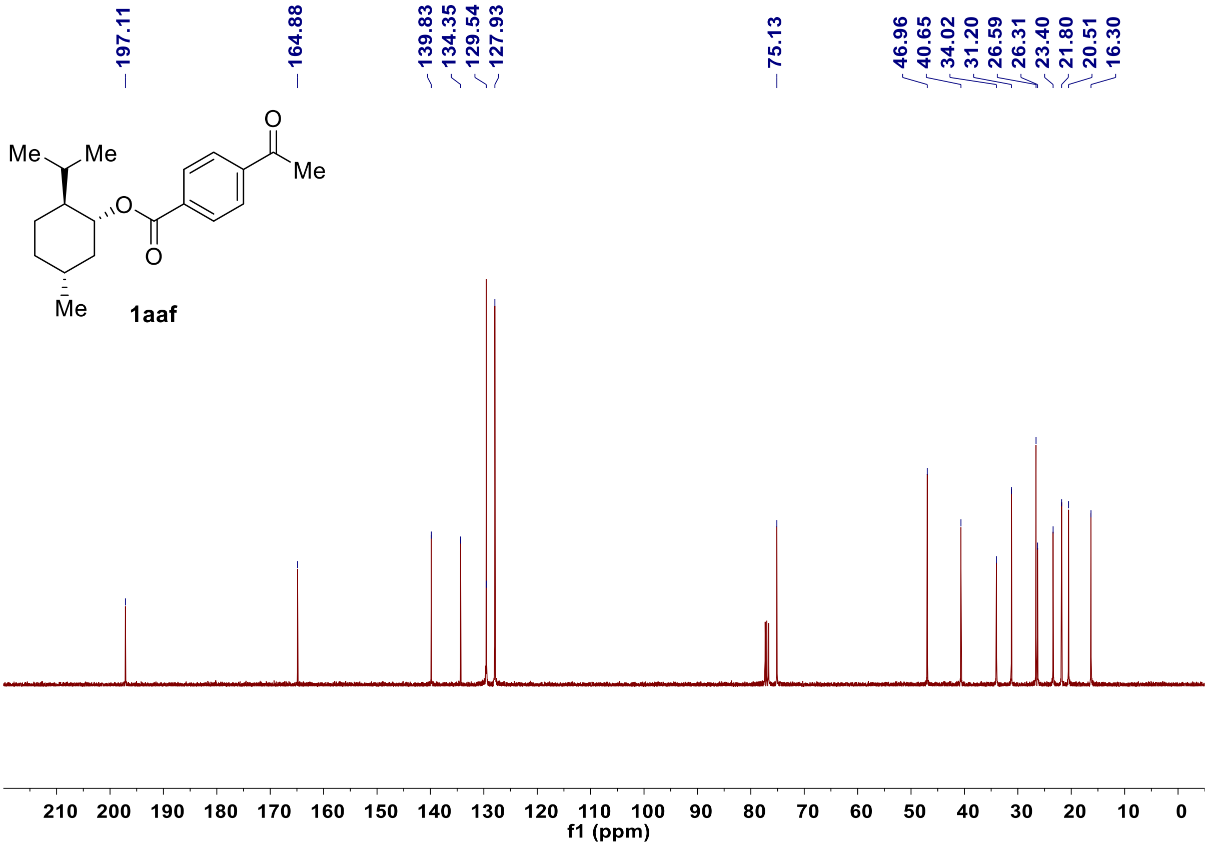

^{1}H and ^{13}C NMR spectra for compound 1aag

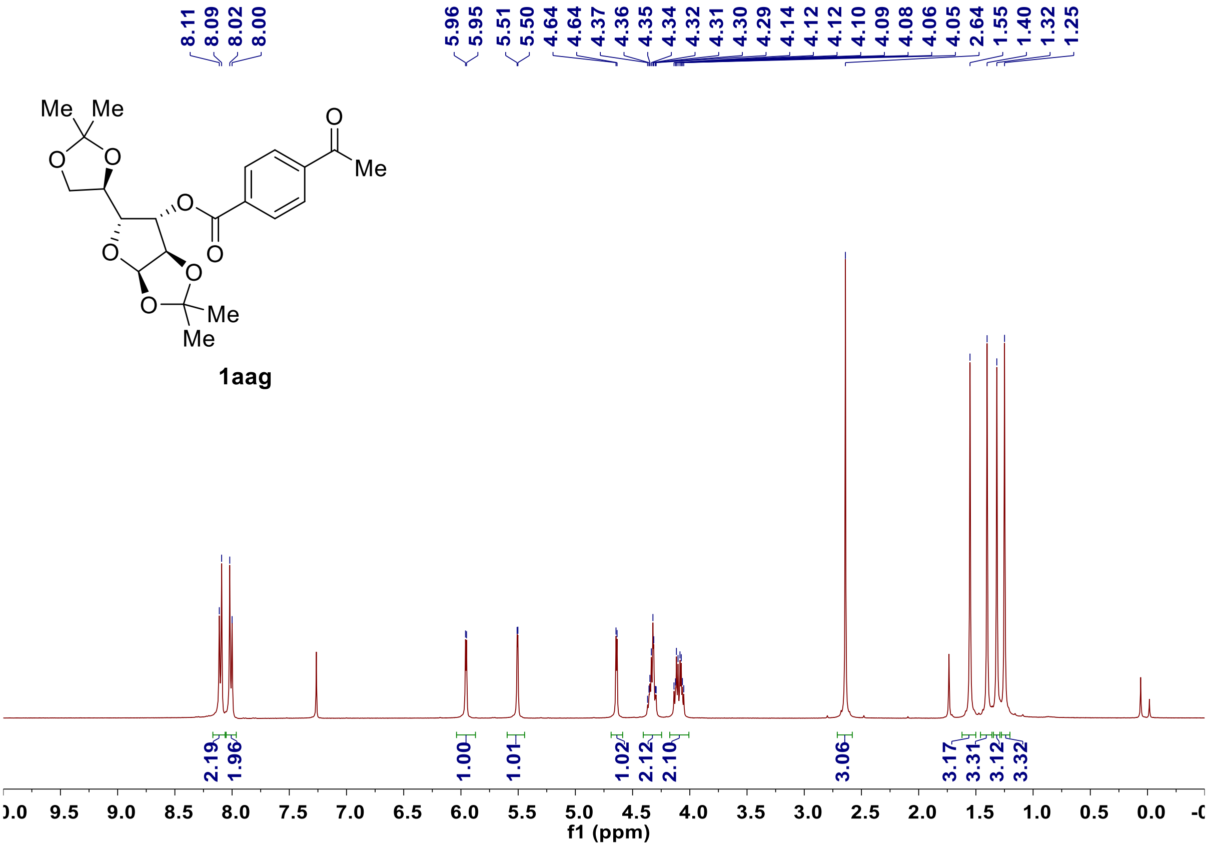

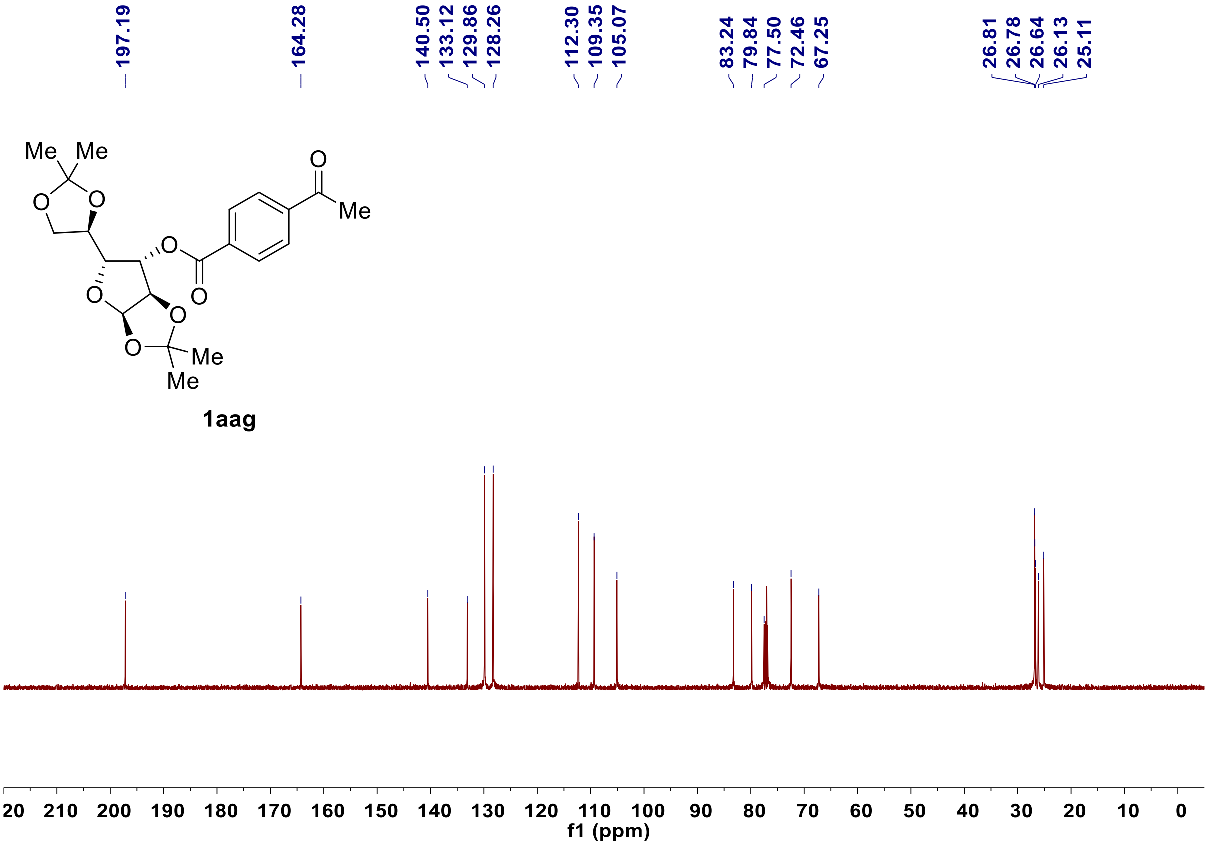

^1H and ^{13}C NMR spectra for compound 1aah

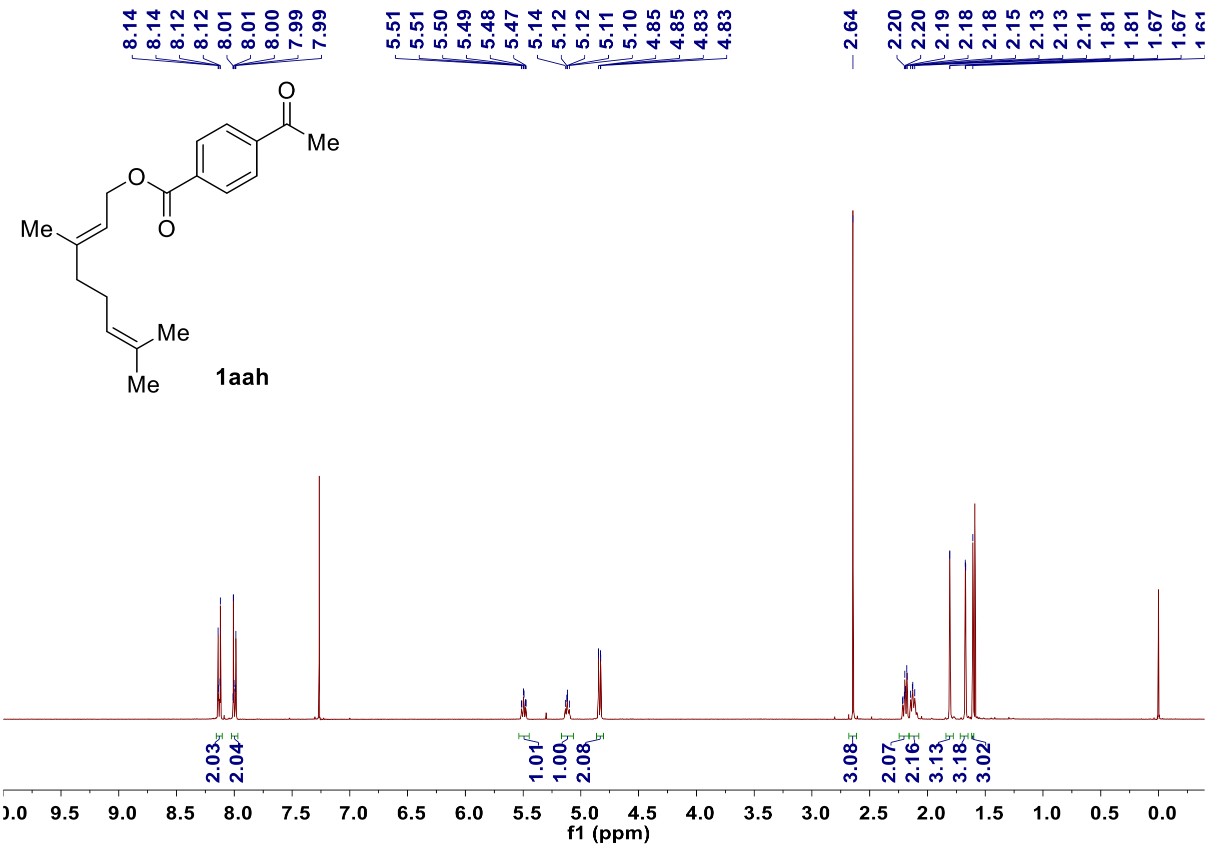

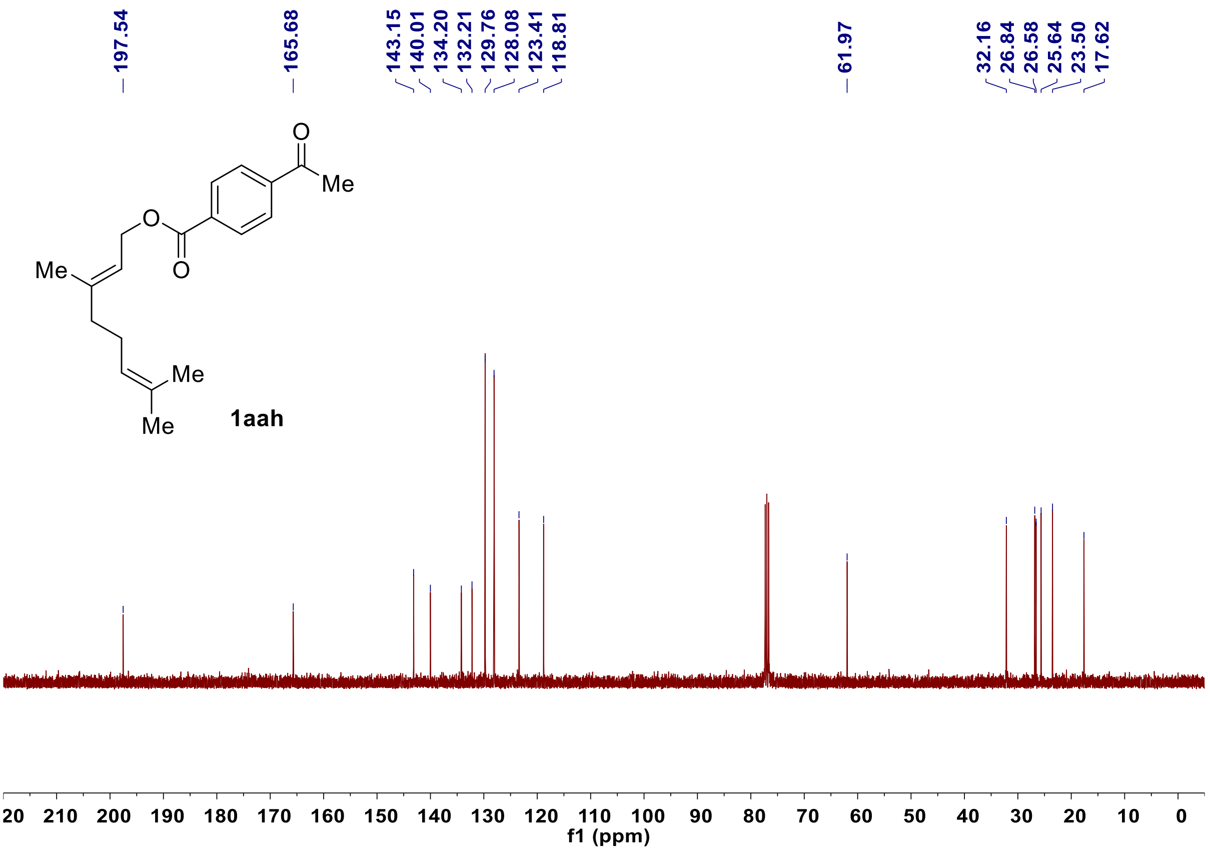

^{1}H and ^{13}C NMR spectra for compound 1aaj

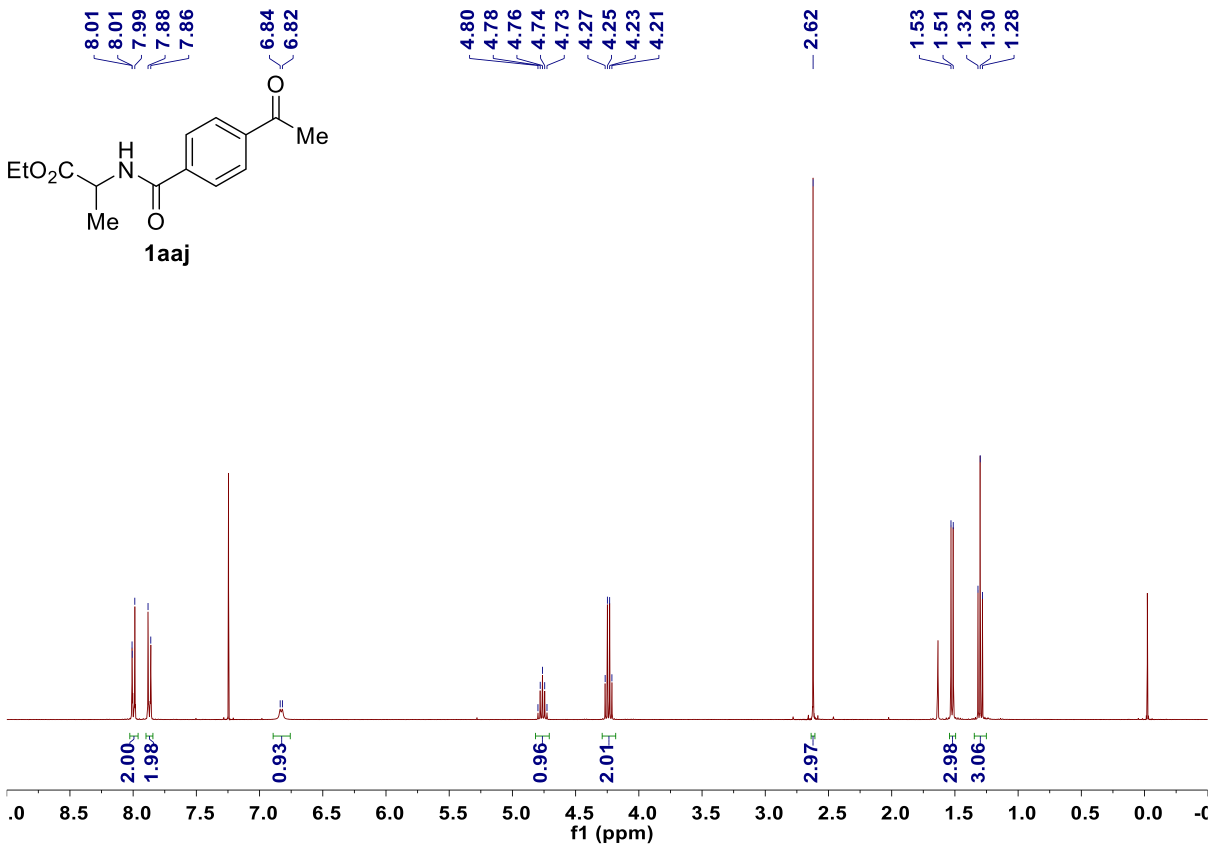

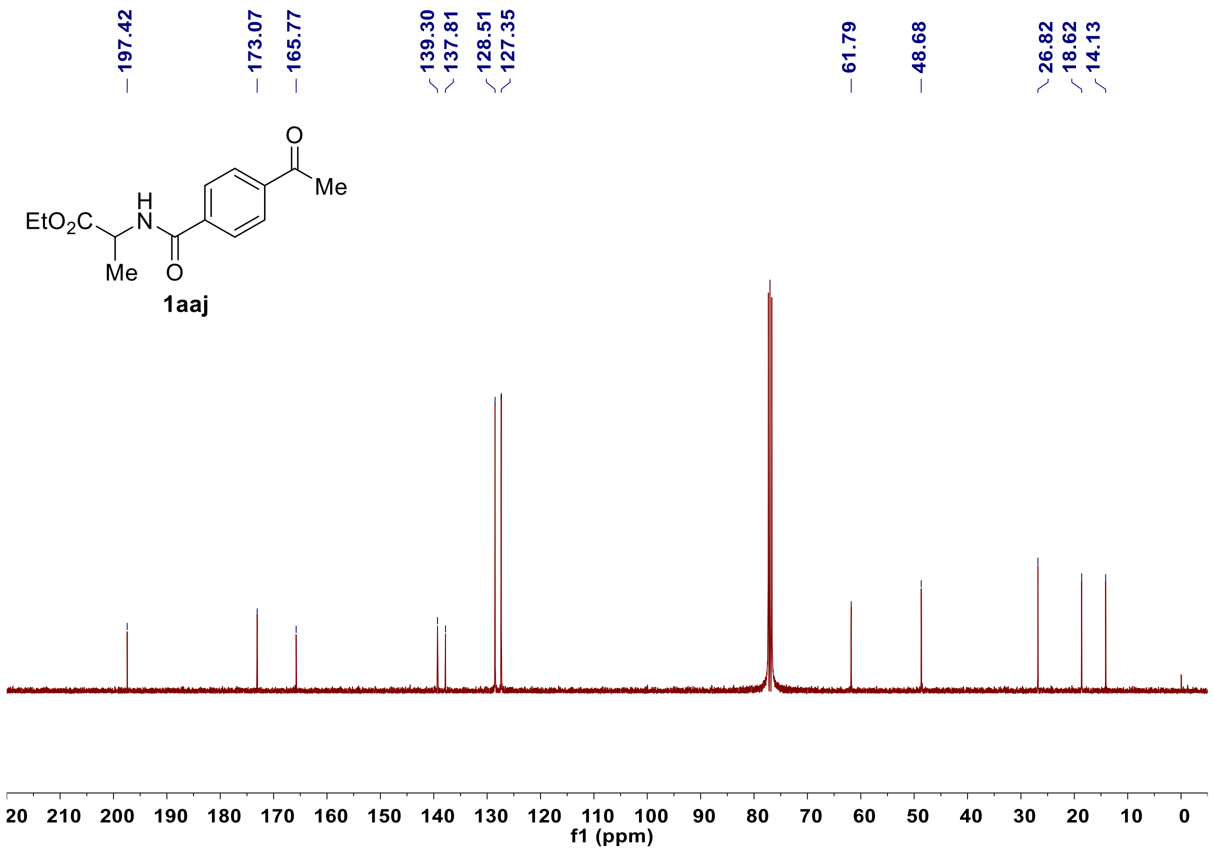

^{1}H and ^{13}C NMR spectra for compound 1aak

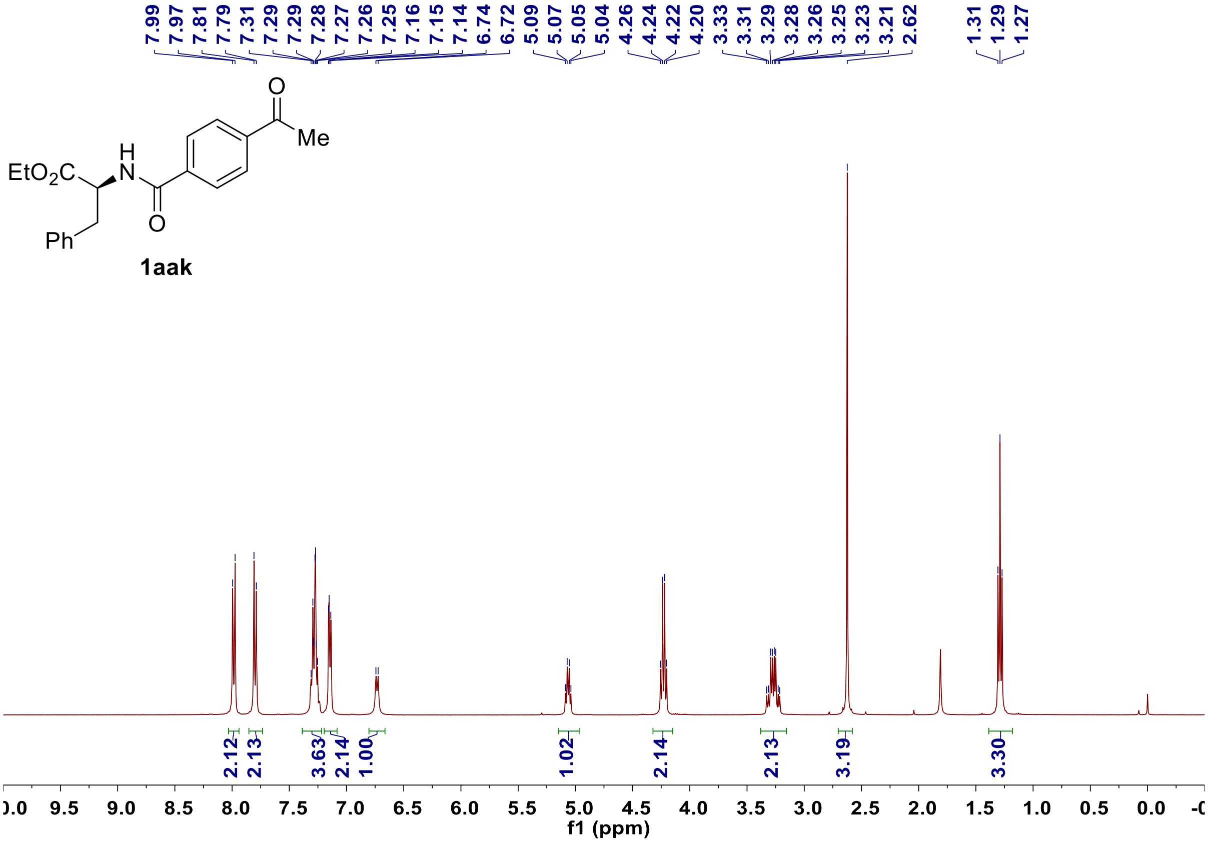

1aak

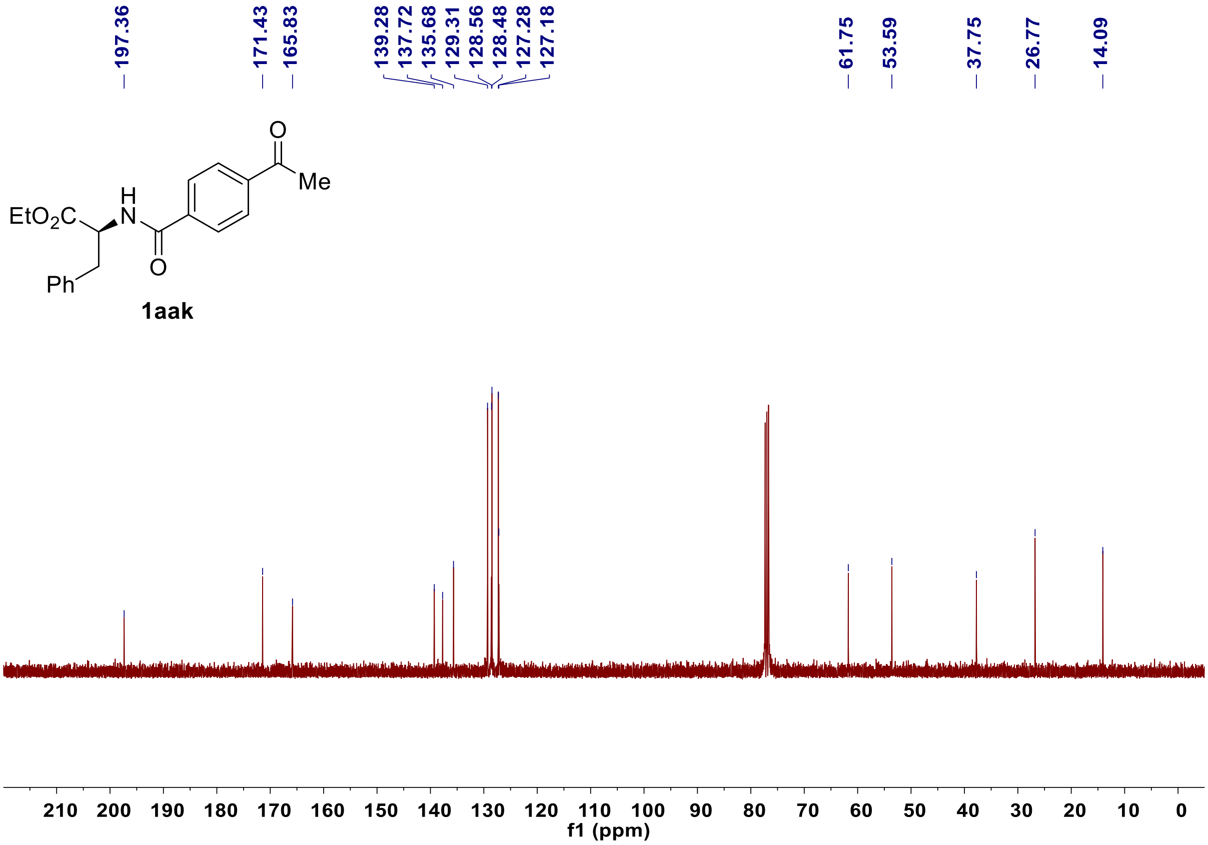

1aak

^{1}H and ^{13}C NMR spectra for compound 1aal

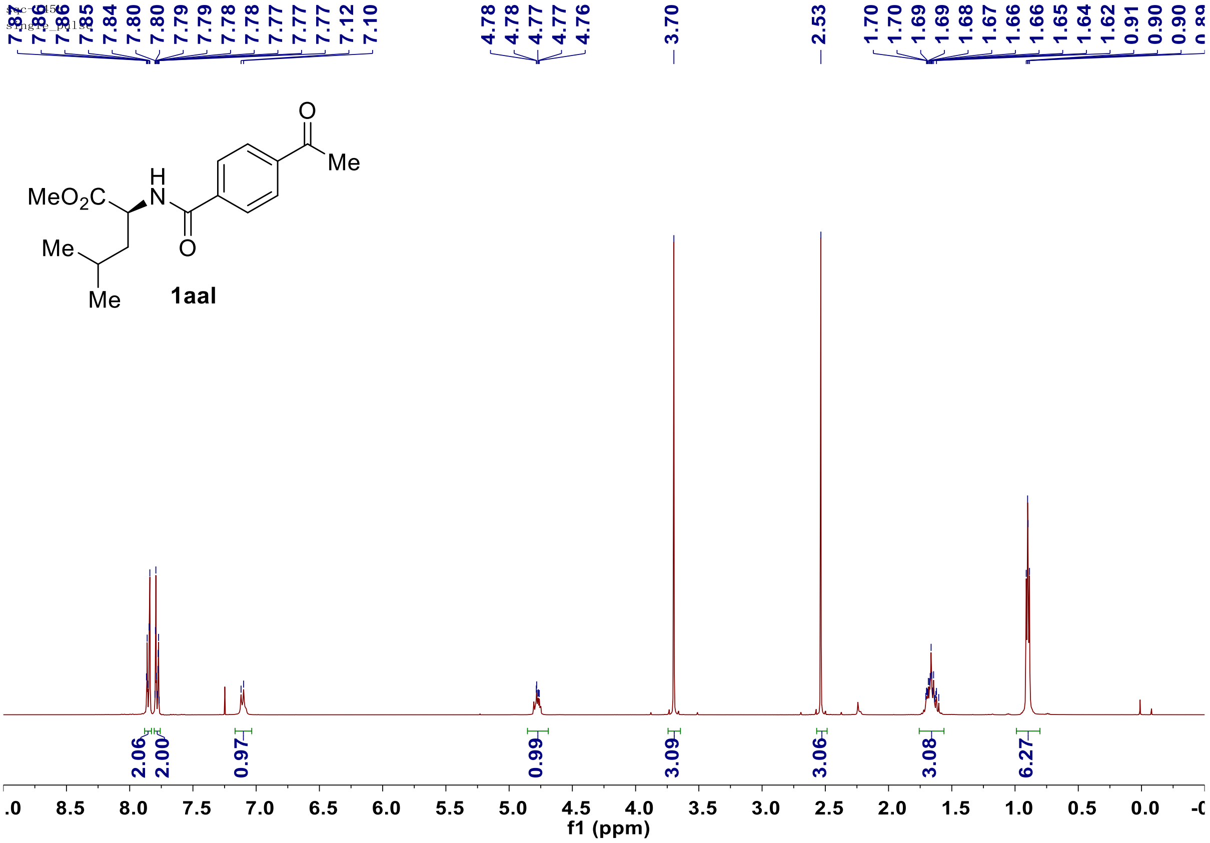

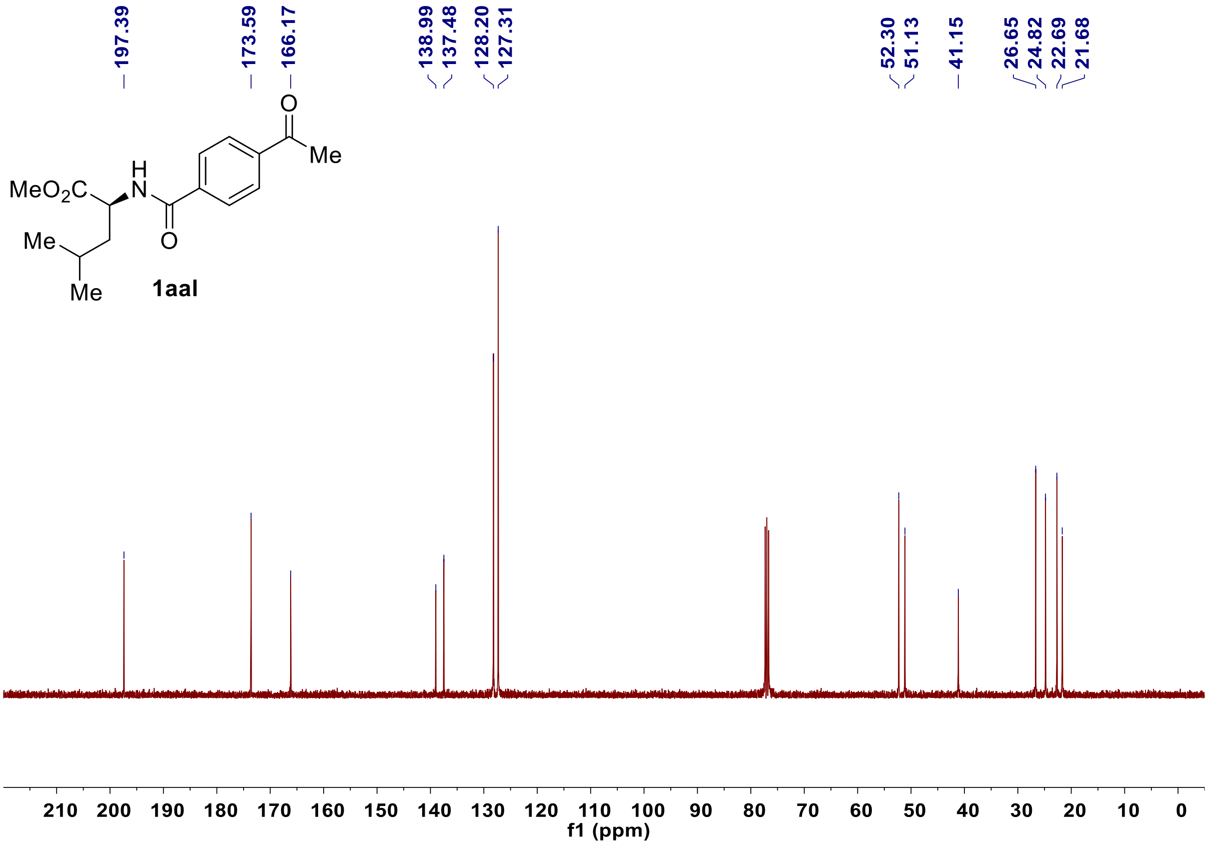

^{1}H and ^{13}C NMR spectra for compound 2aq

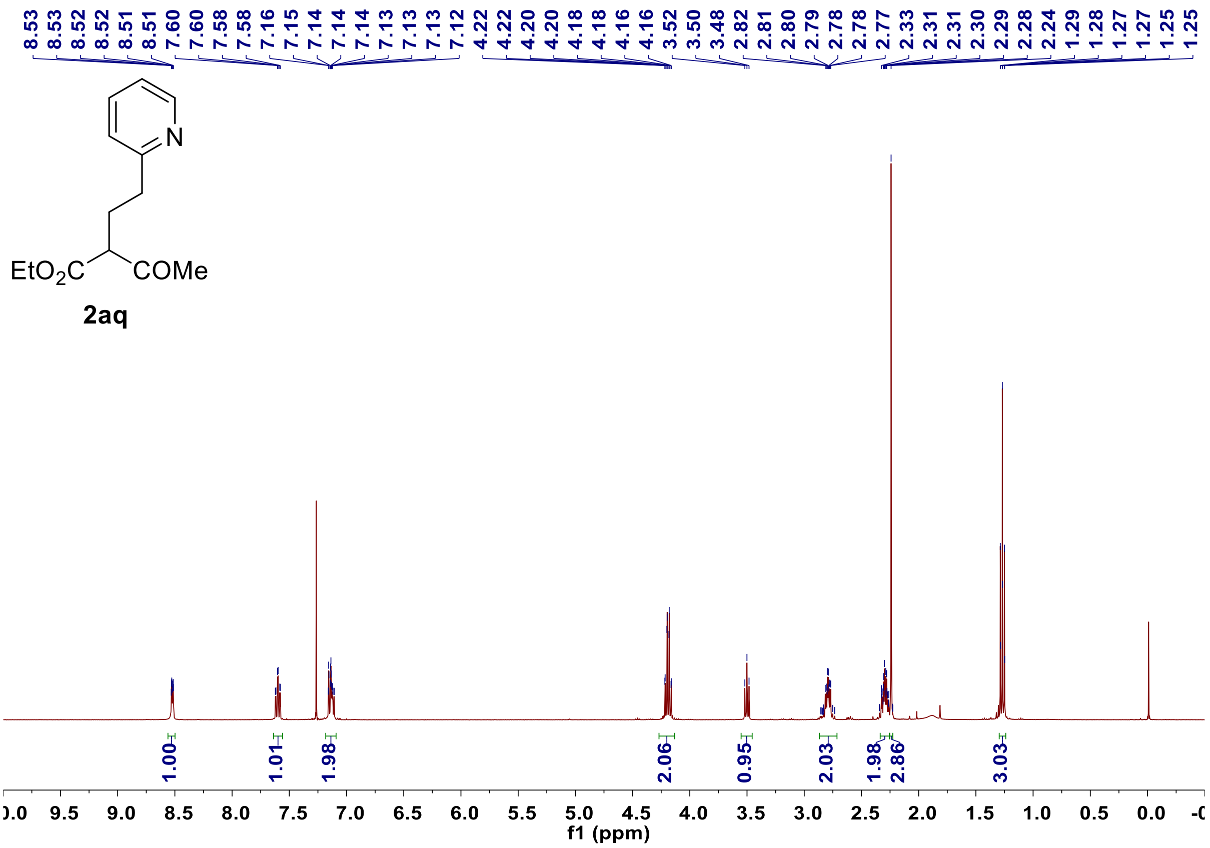

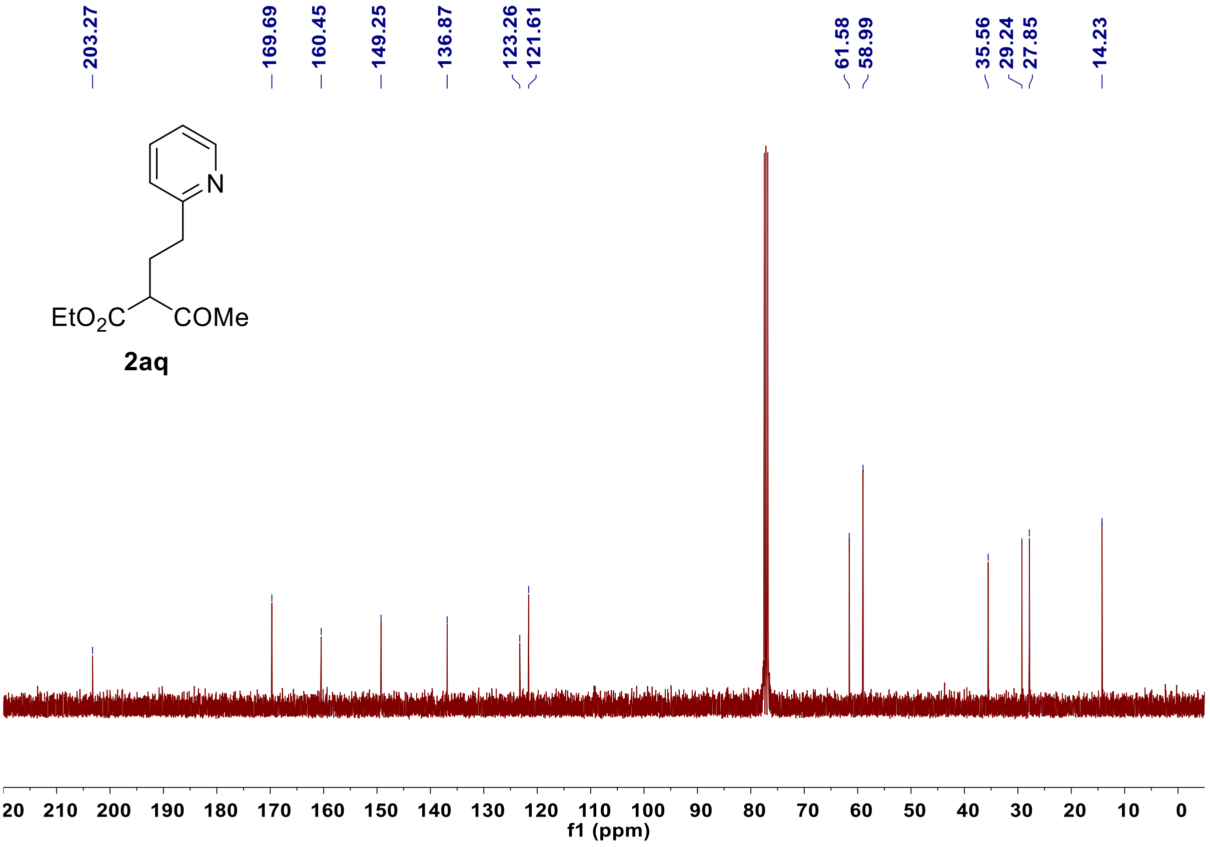

^{1}H and ^{13}C NMR spectra for compound 2ar

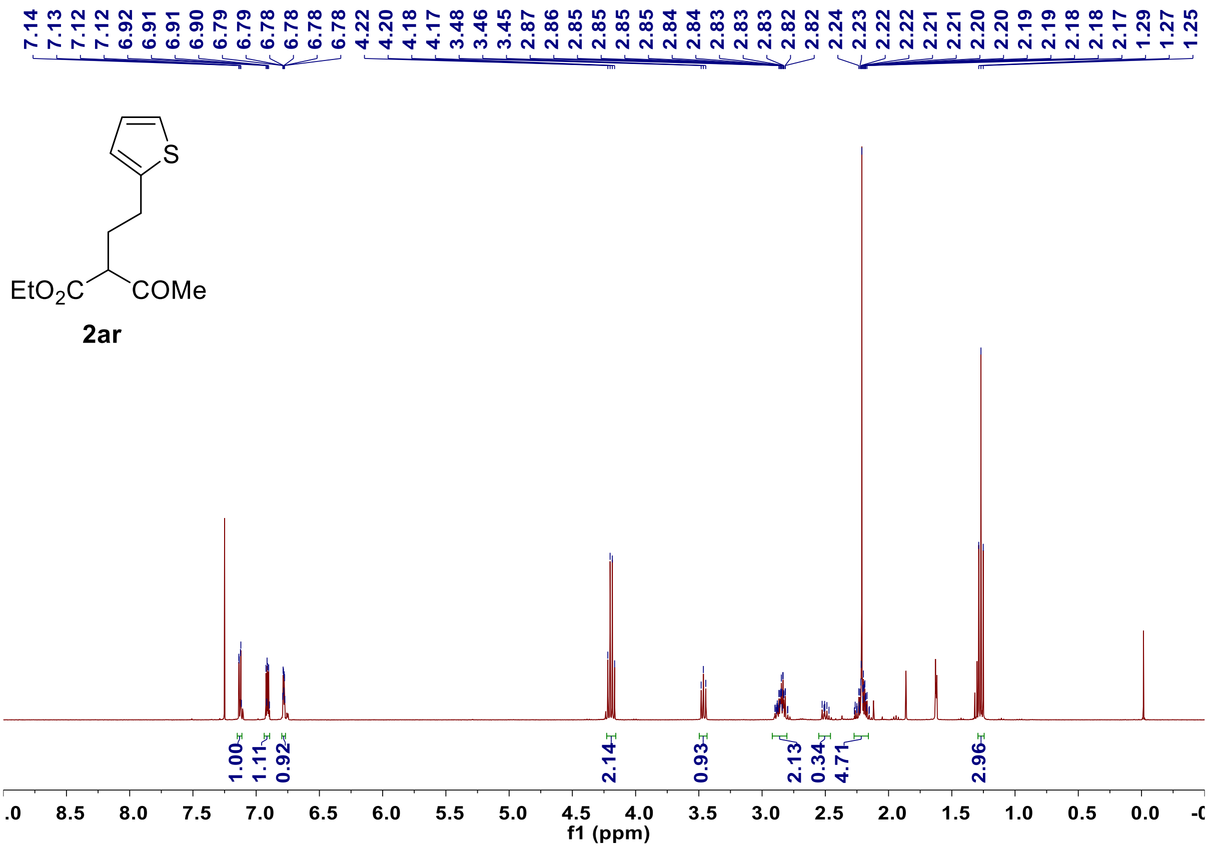

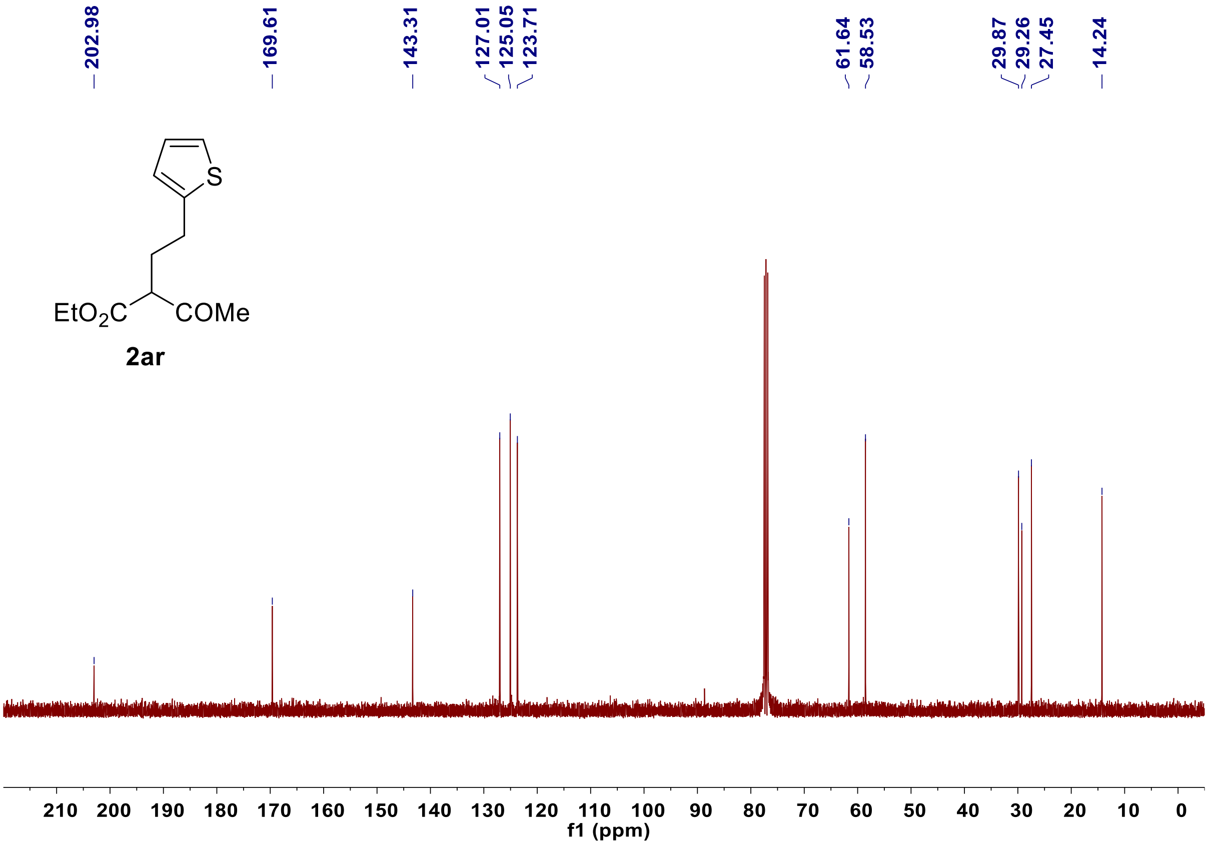

^{1}H and ^{13}C NMR spectra for compound 2as

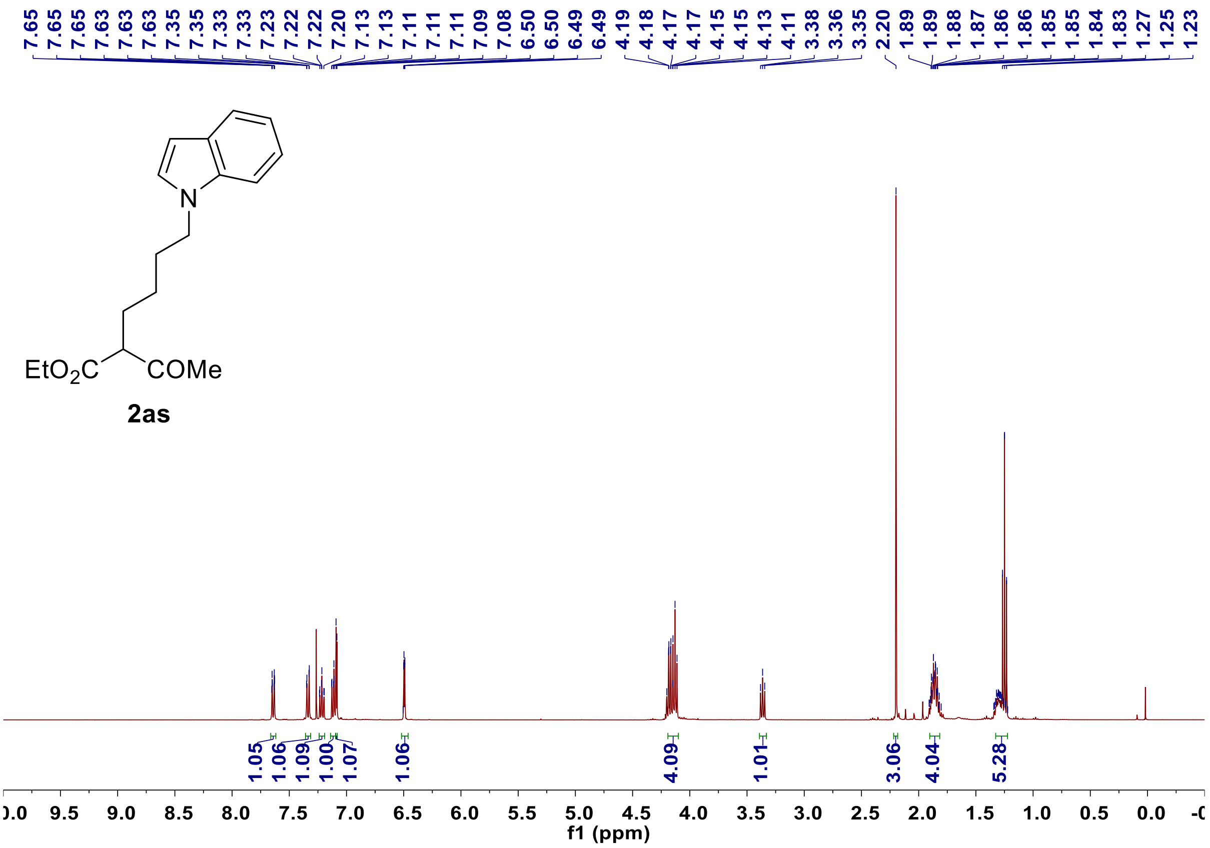

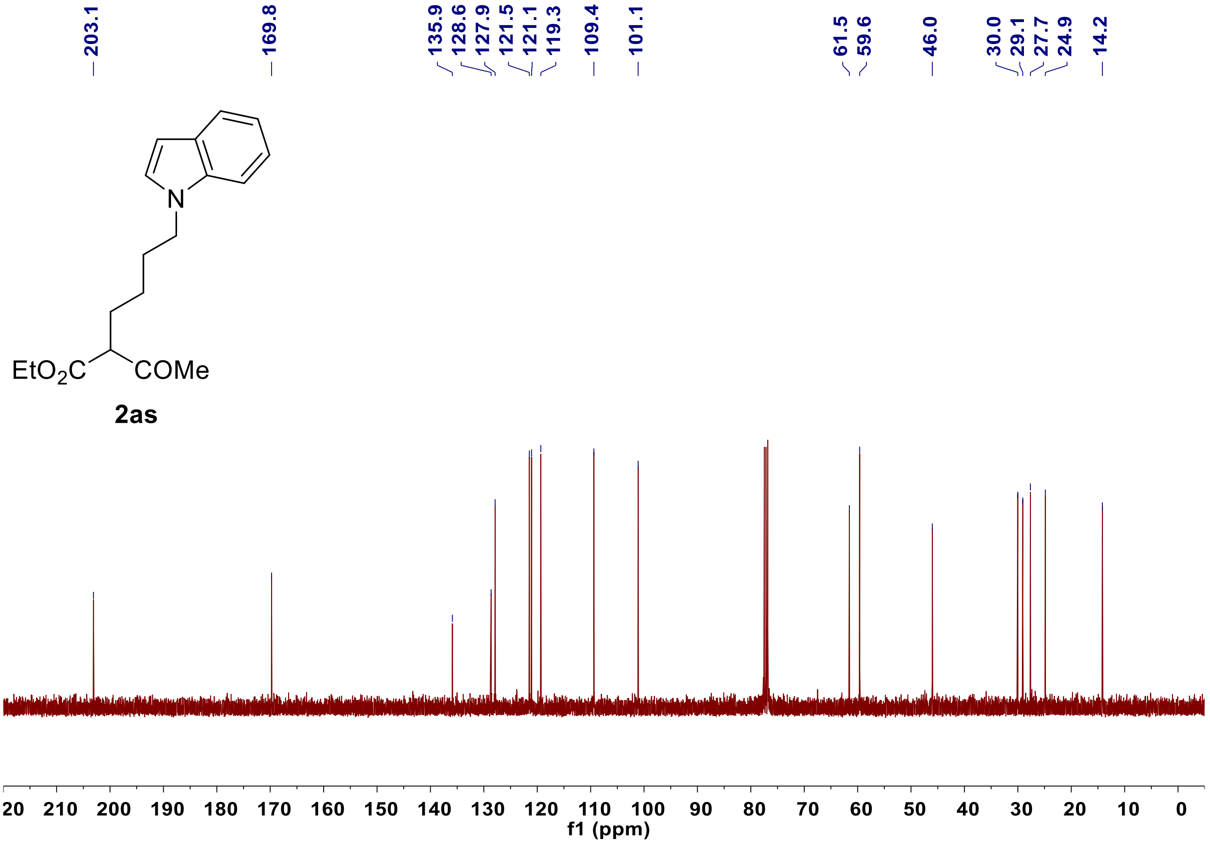

^{1}H and ^{13}C NMR spectra for compound 2av

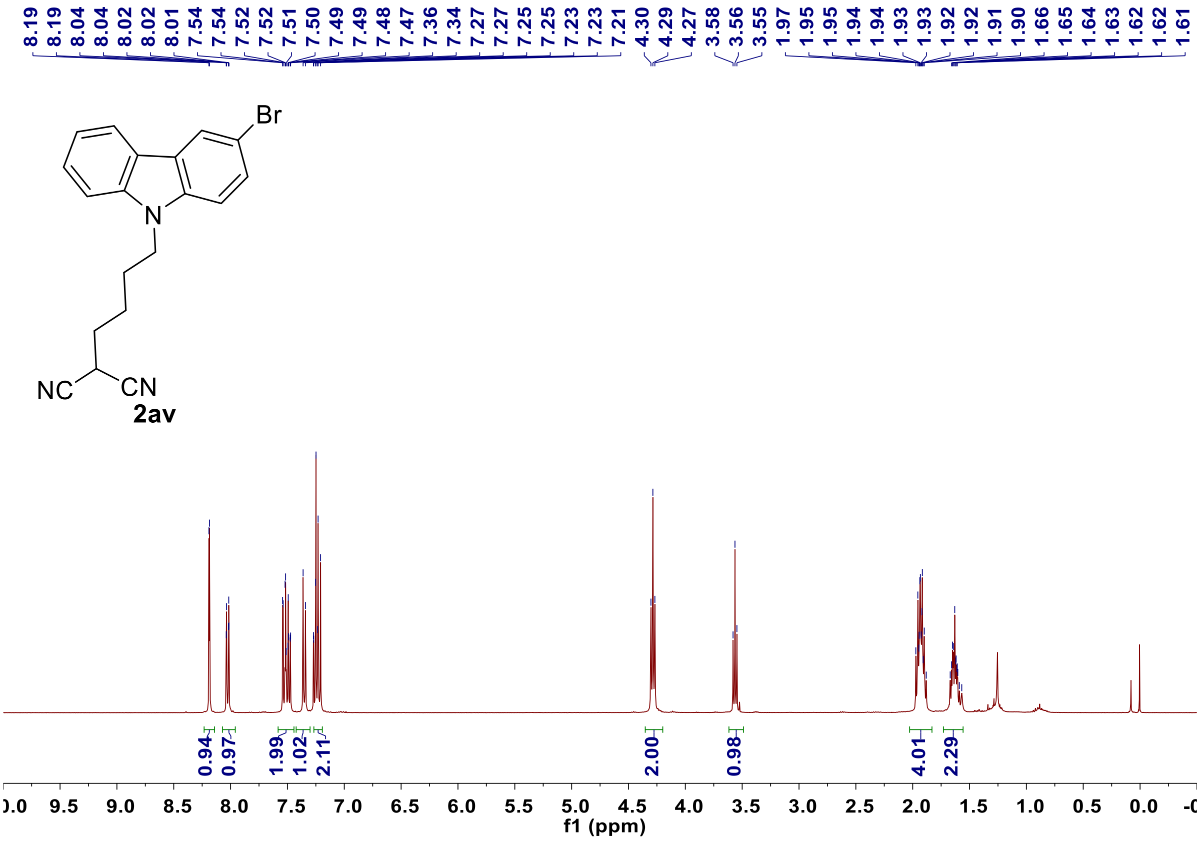

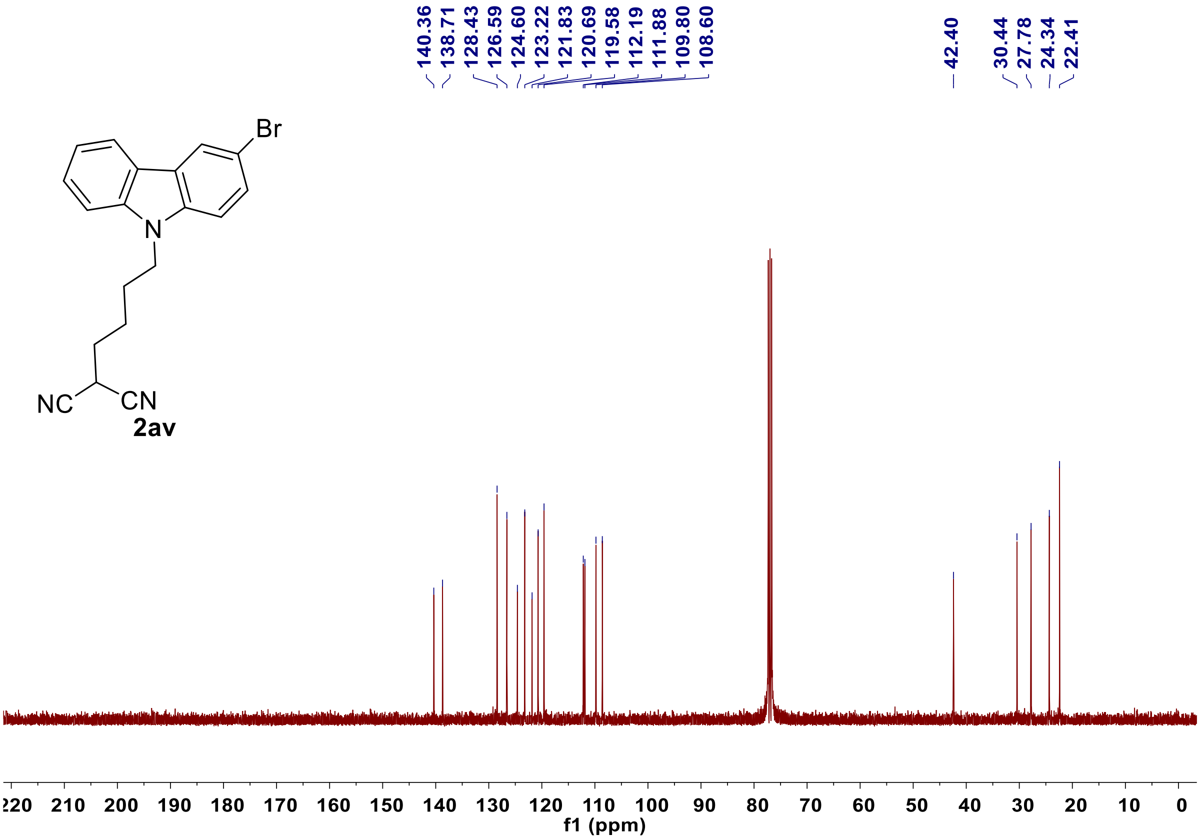

^{1}H and ^{13}C NMR spectra for compound 2aw

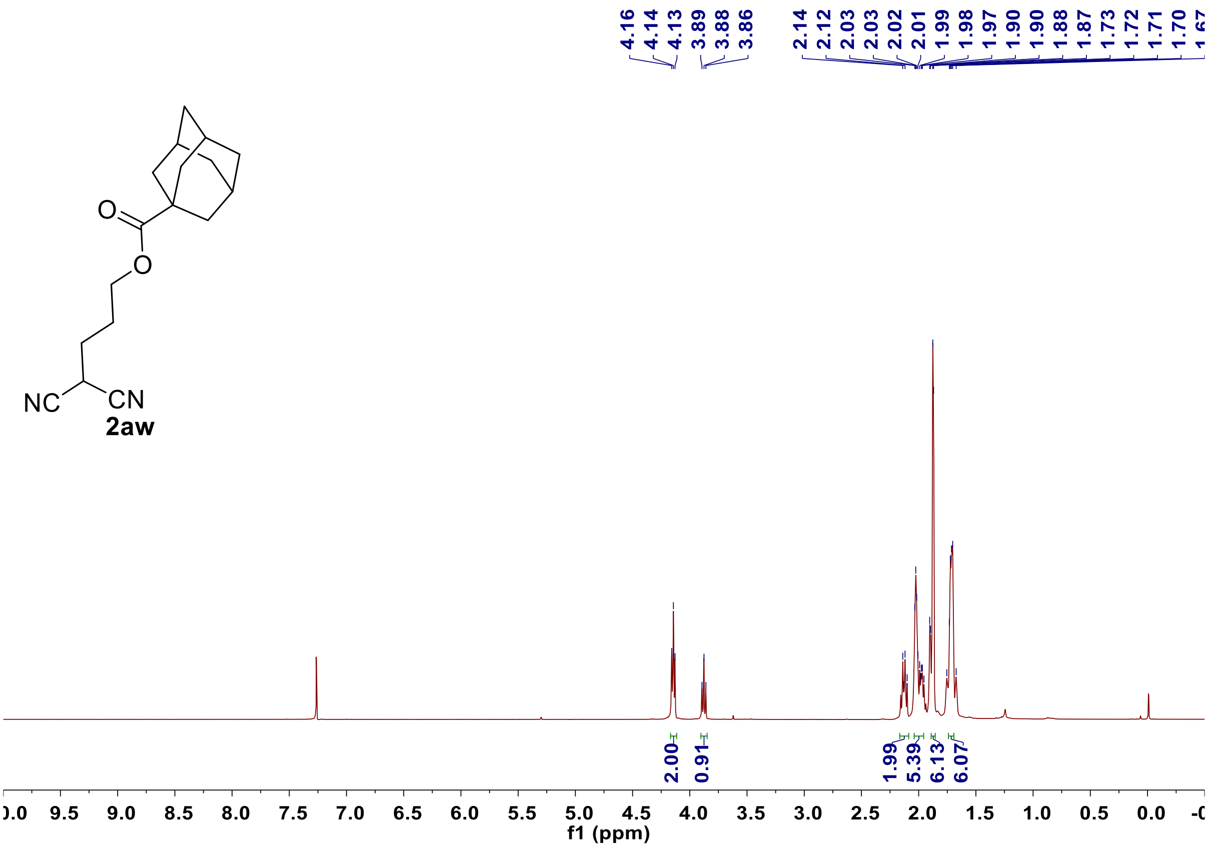

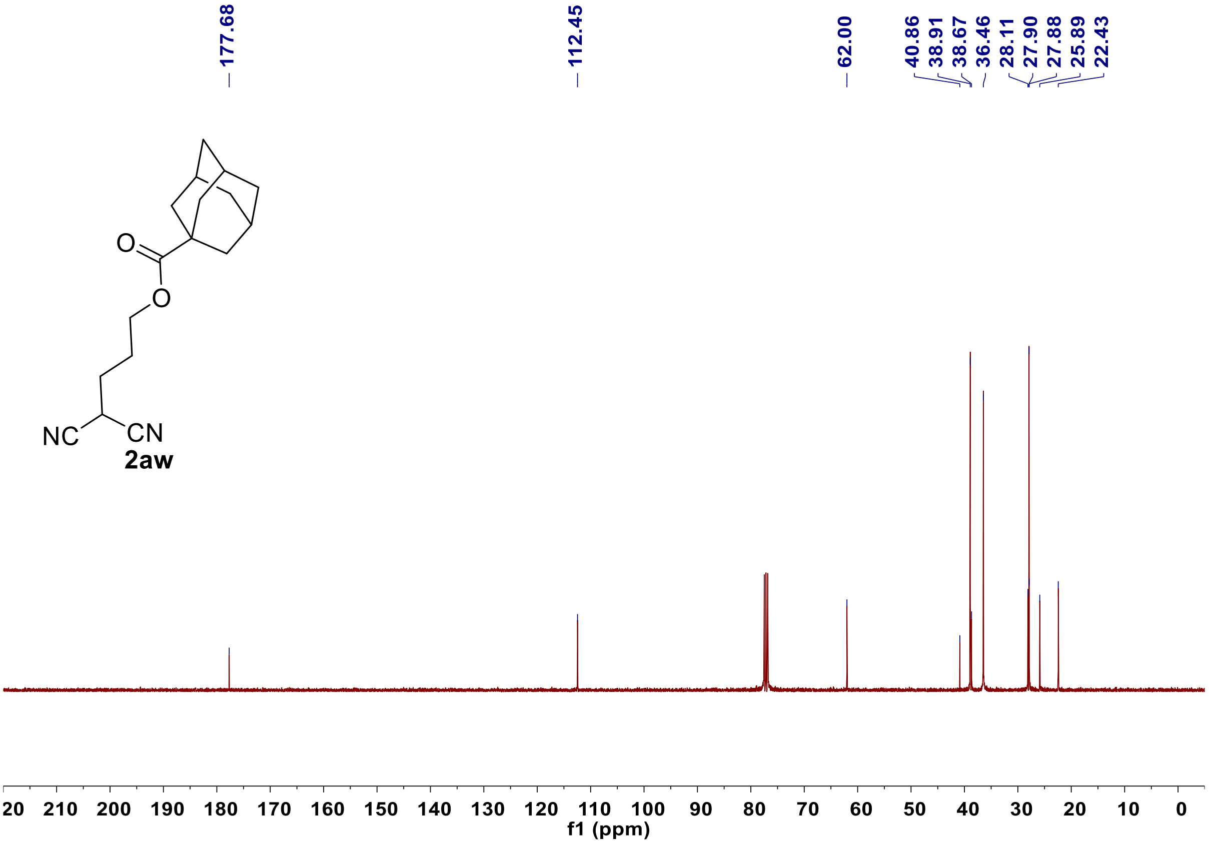

11. NMR spectra of the products

^{1}H and ^{13}C NMR spectra for compound 3

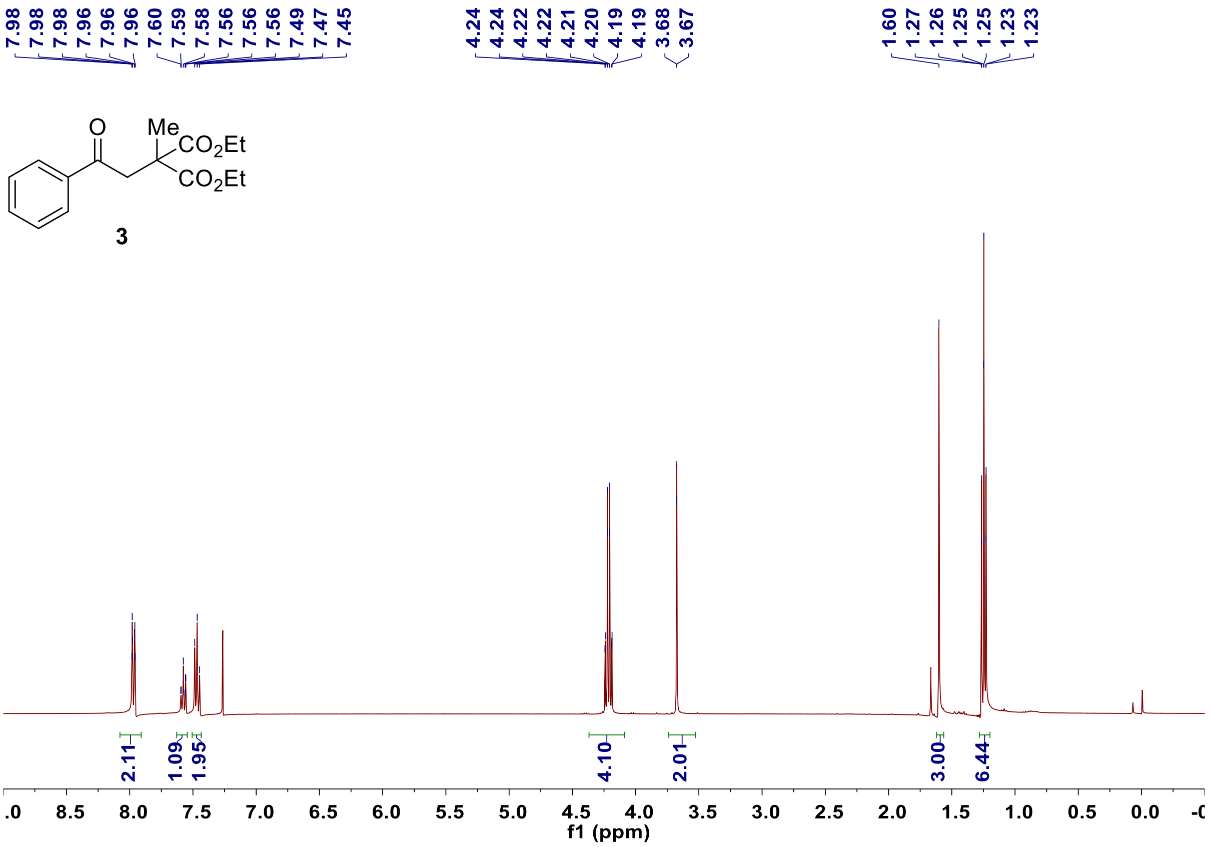

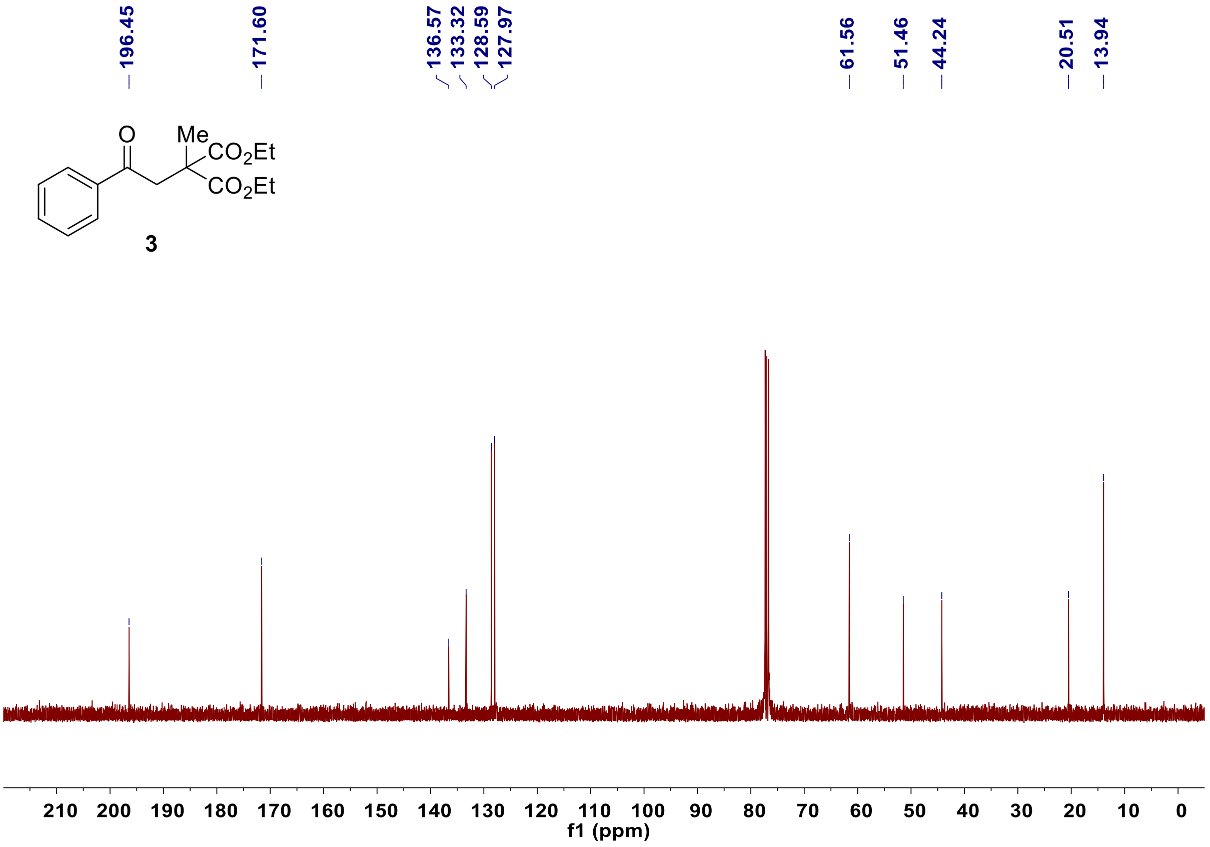

^1H and ^{13}C NMR spectra for compound 4

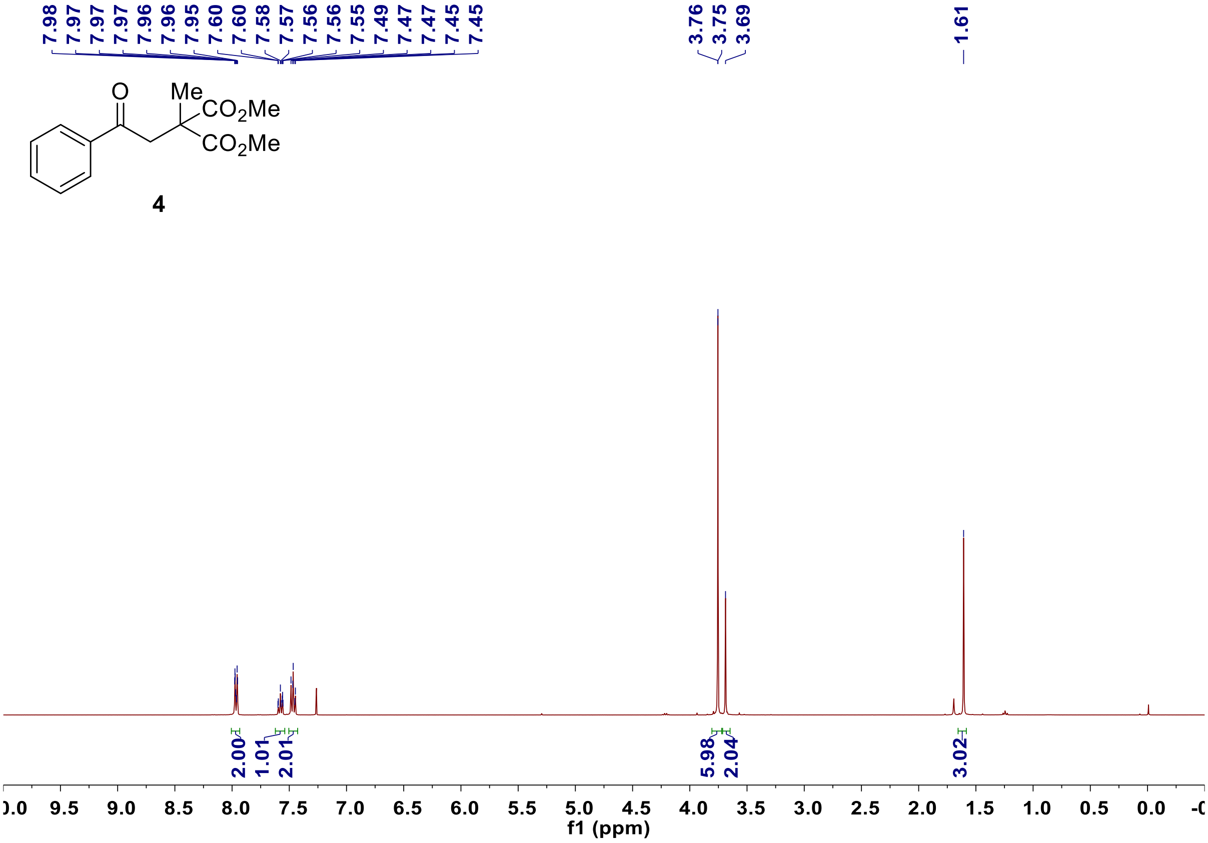

4

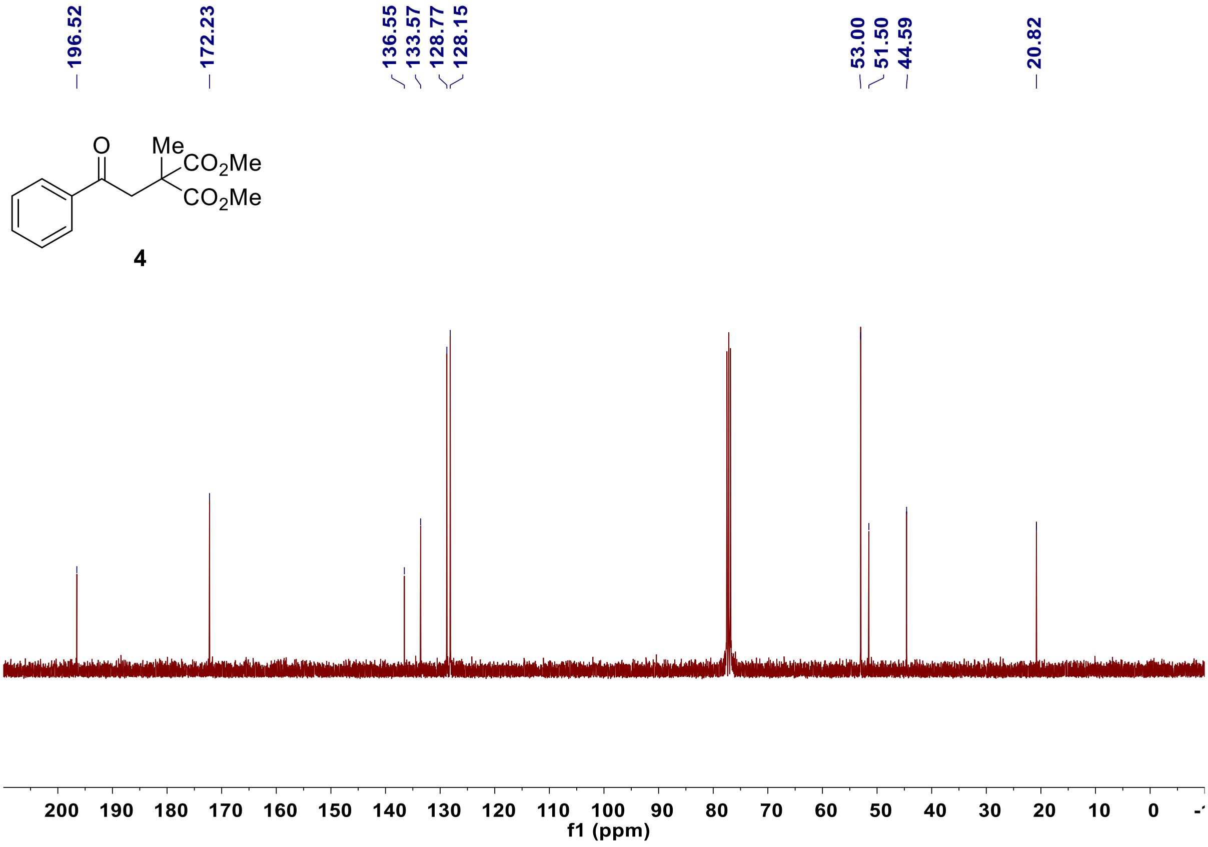

4

^{1}H and ^{13}C NMR spectra for compound 5

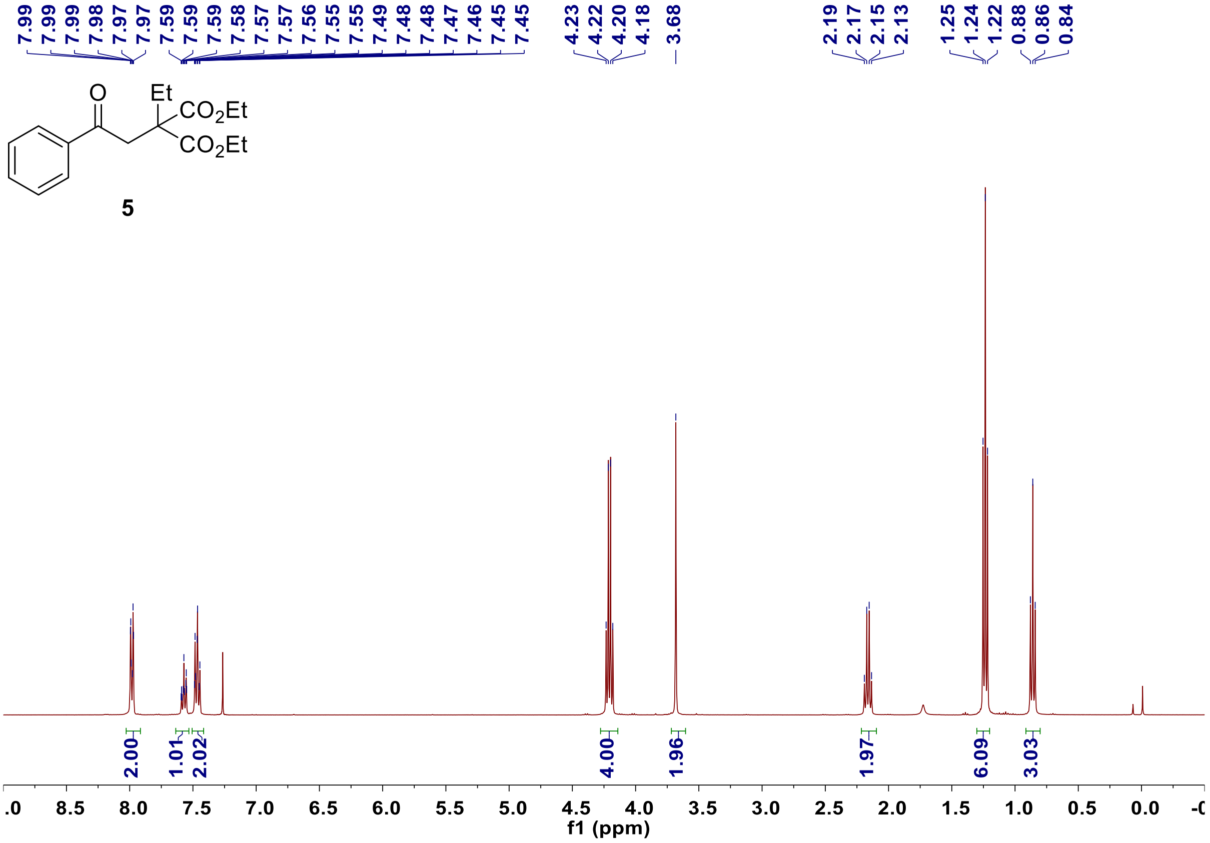

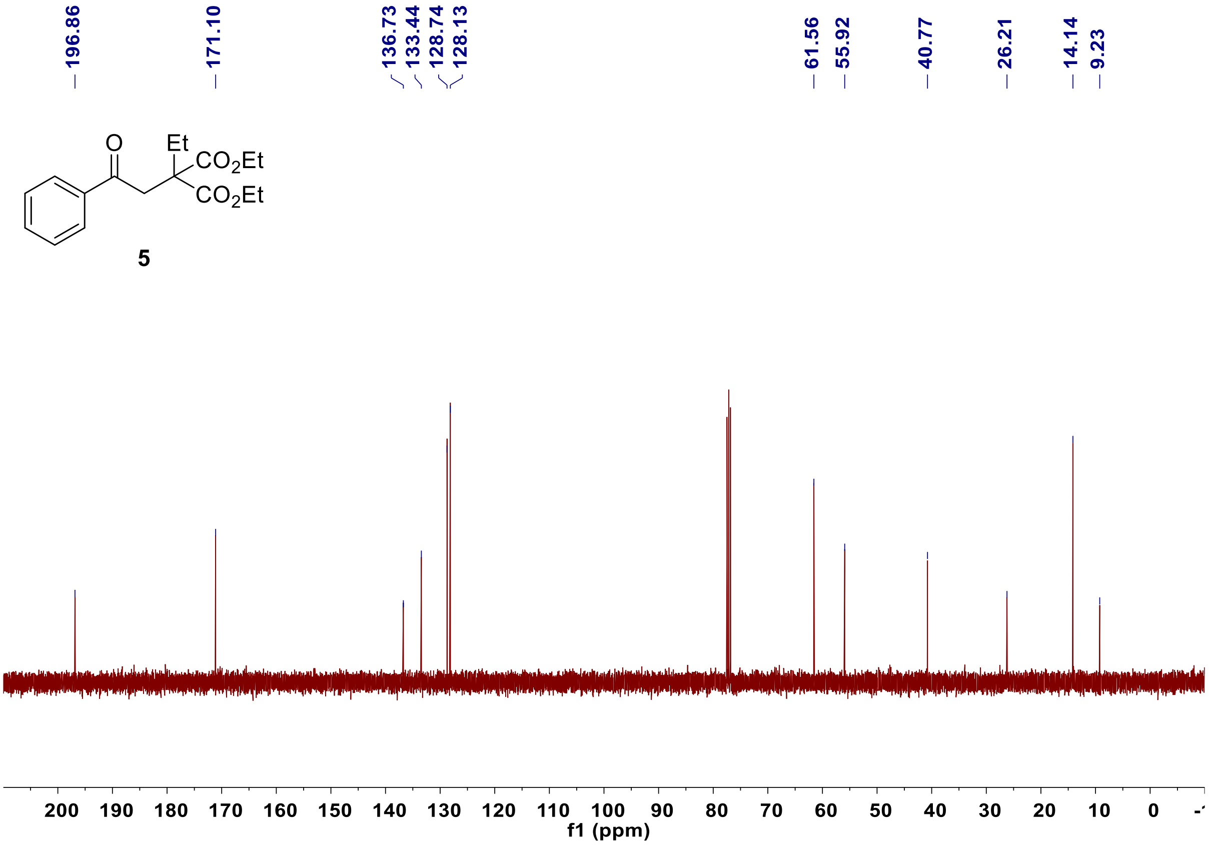

^1H and ^{13}C NMR spectra for compound 6

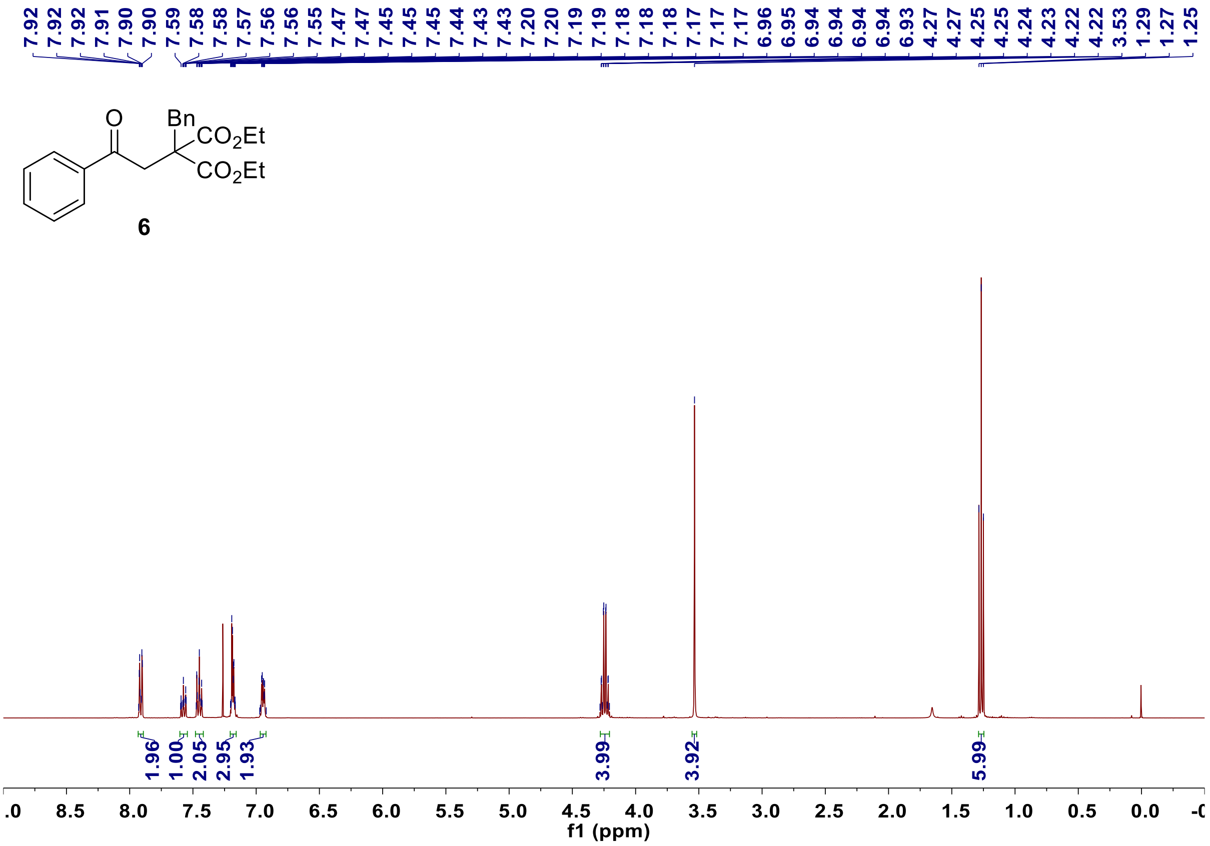

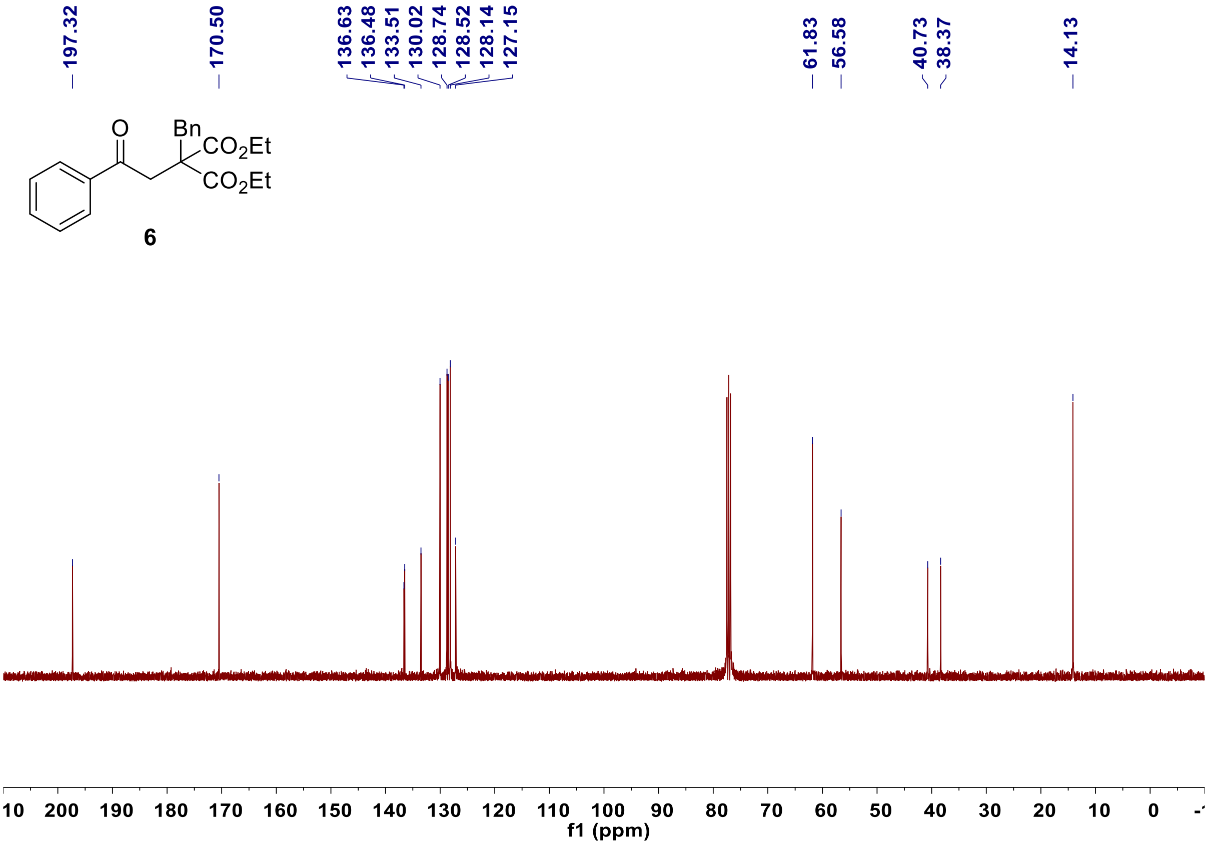

^{1}H and ^{13}C NMR spectra for compound 7

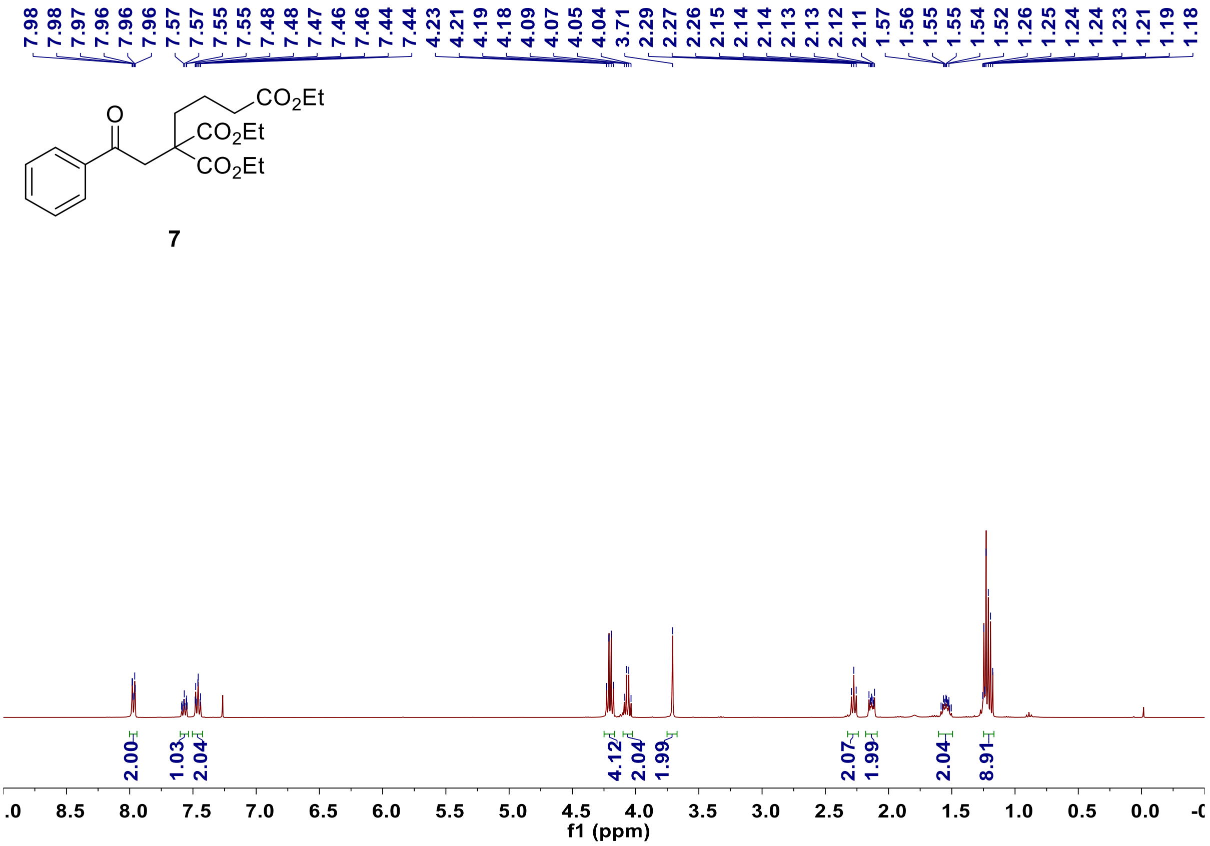

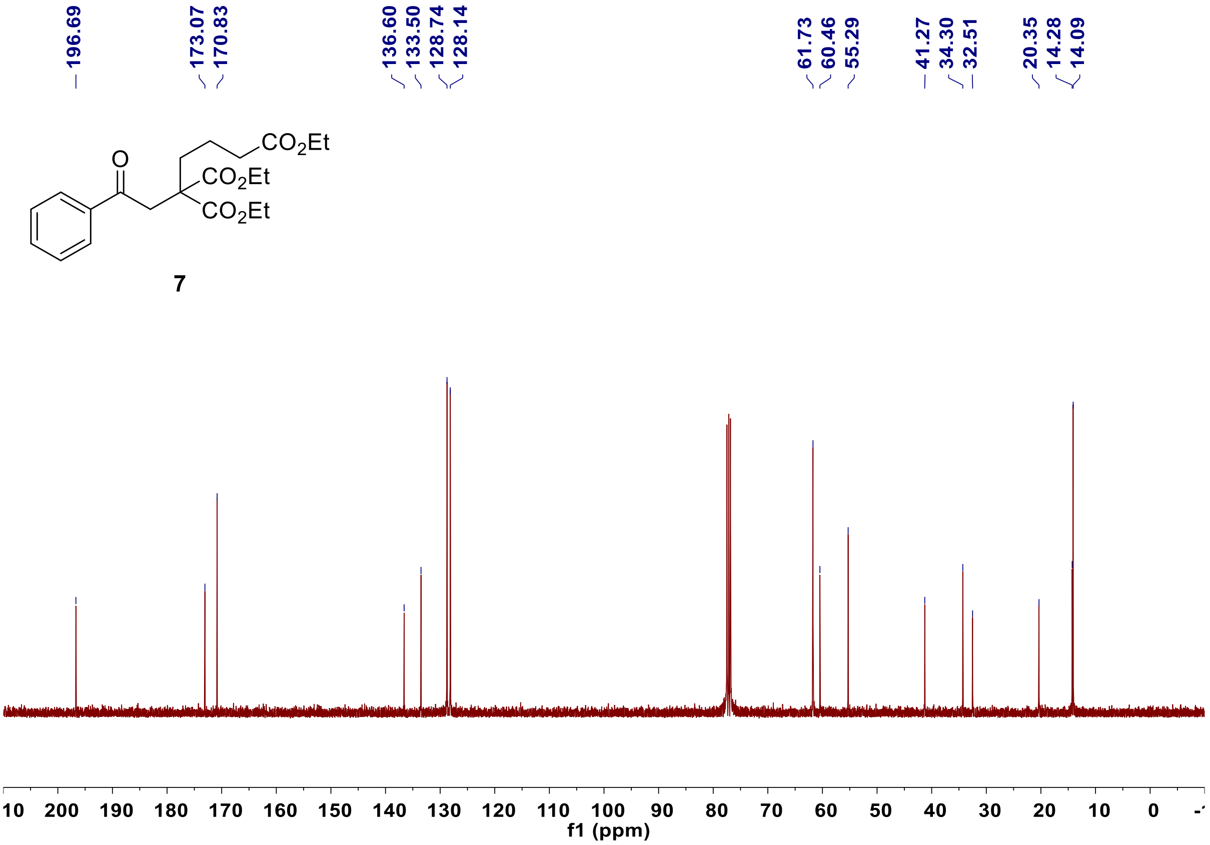

^{1}H and ^{13}C NMR spectra for compound 8

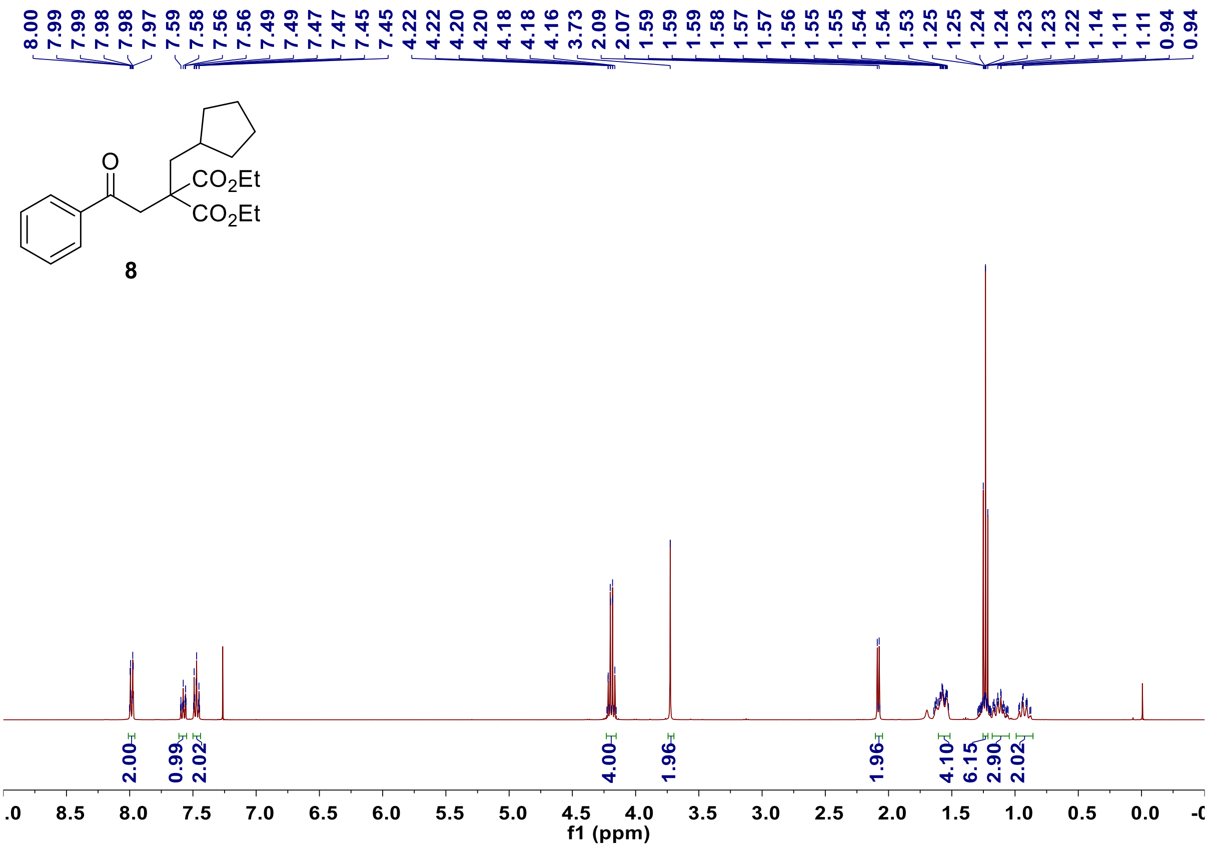

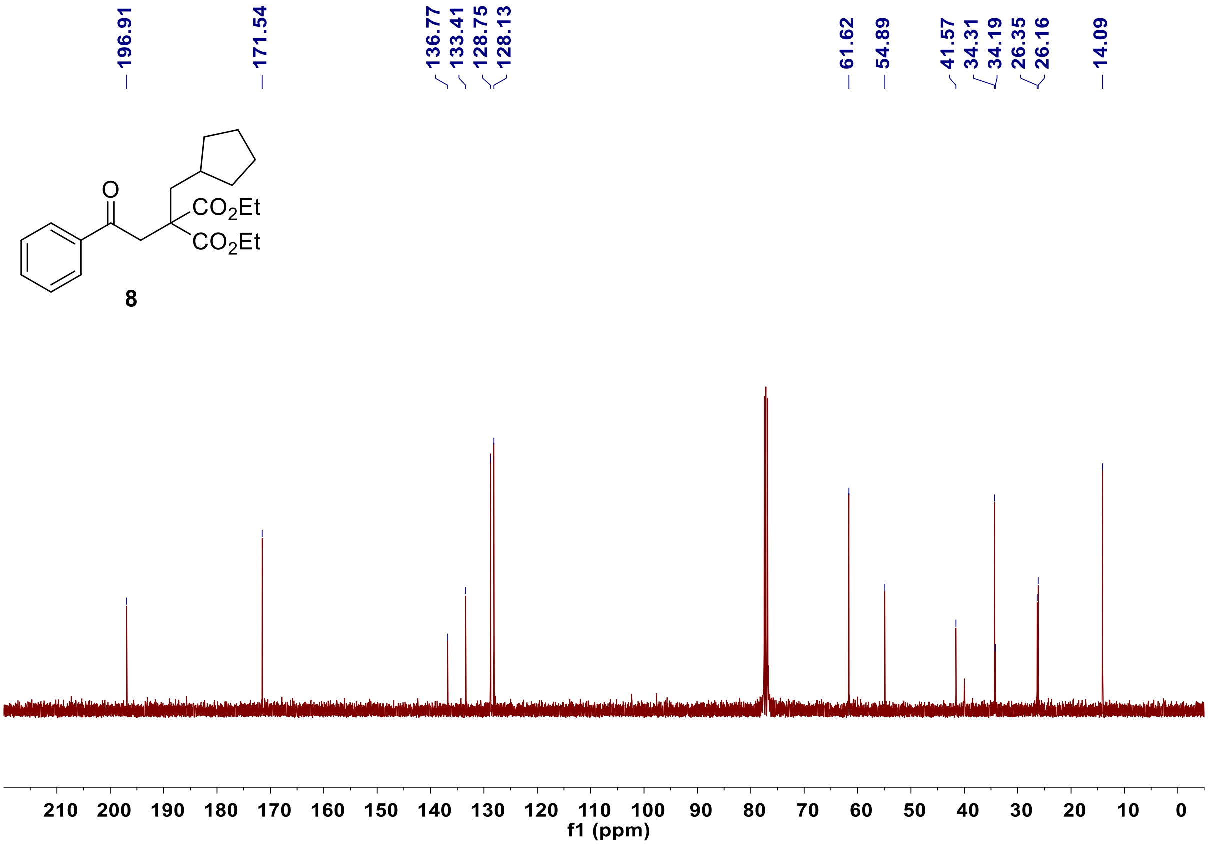

^{1}H and ^{13}C NMR spectra for compound 9

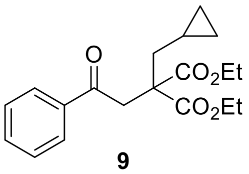

9

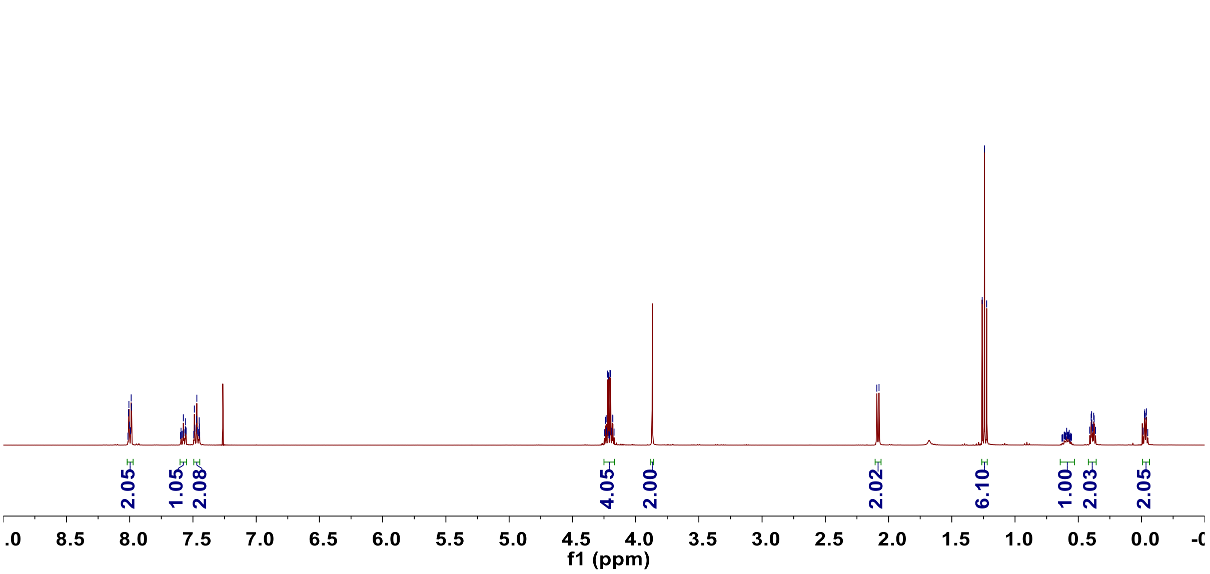

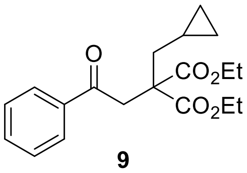

9

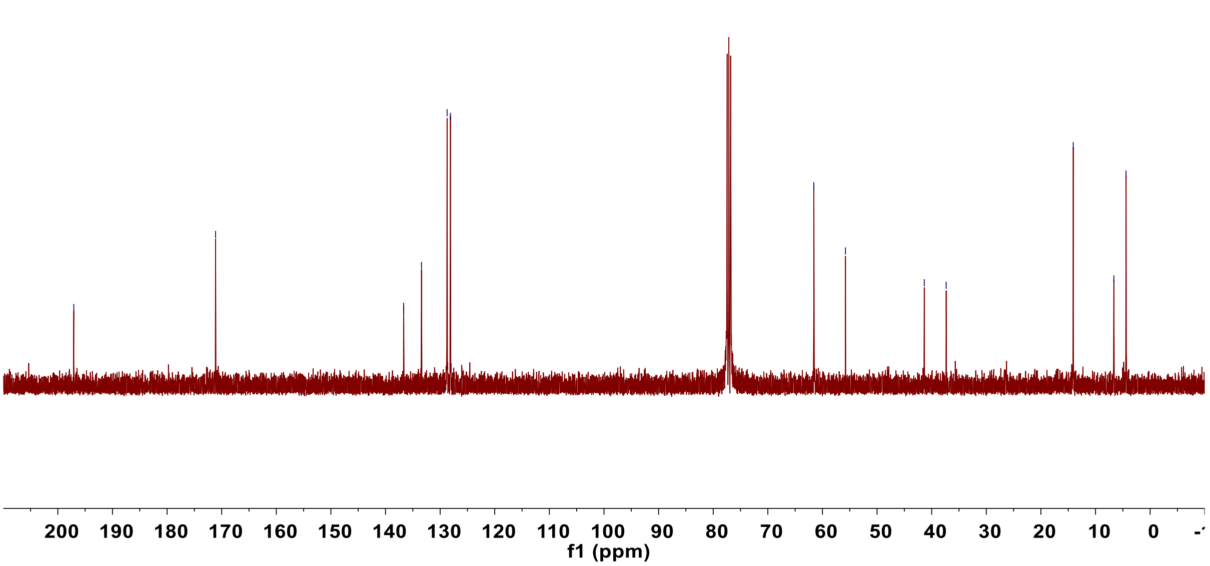

^1H and ^{13}C NMR spectra for compound 10

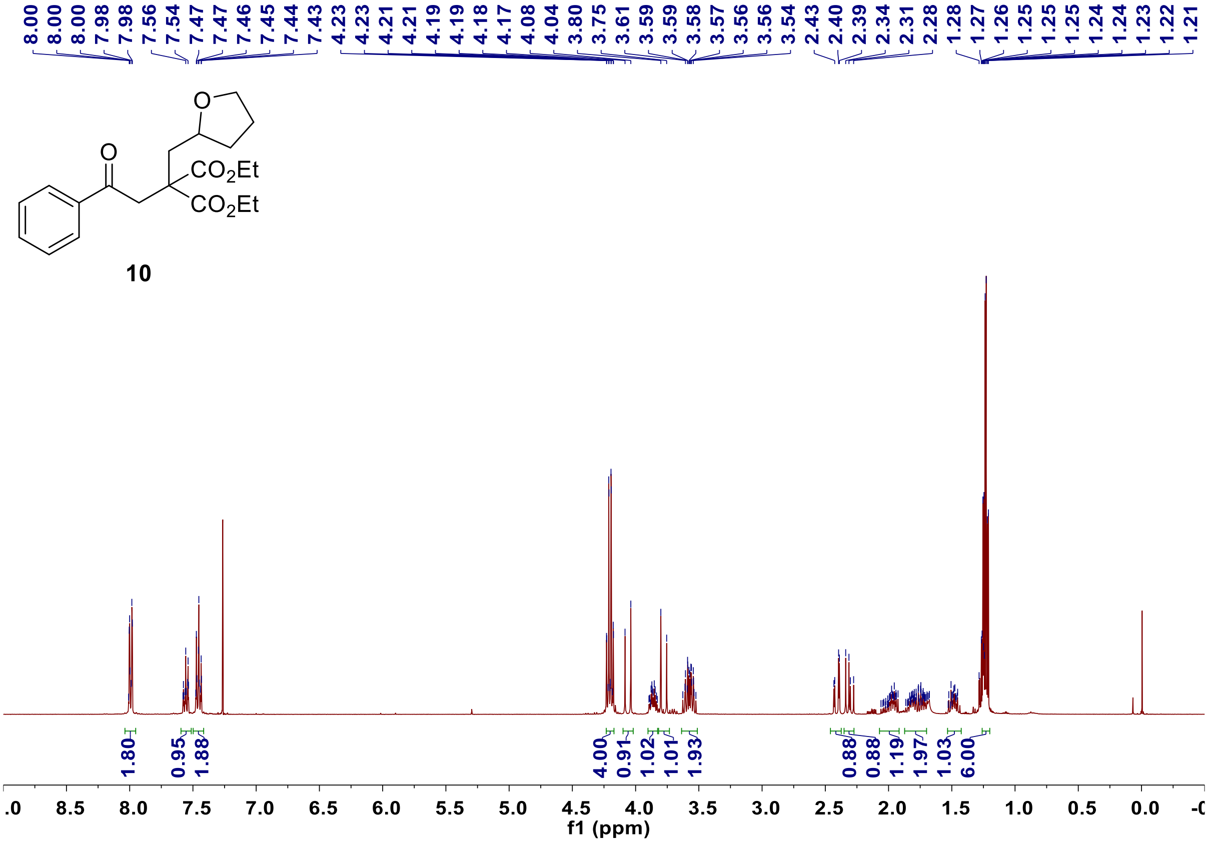

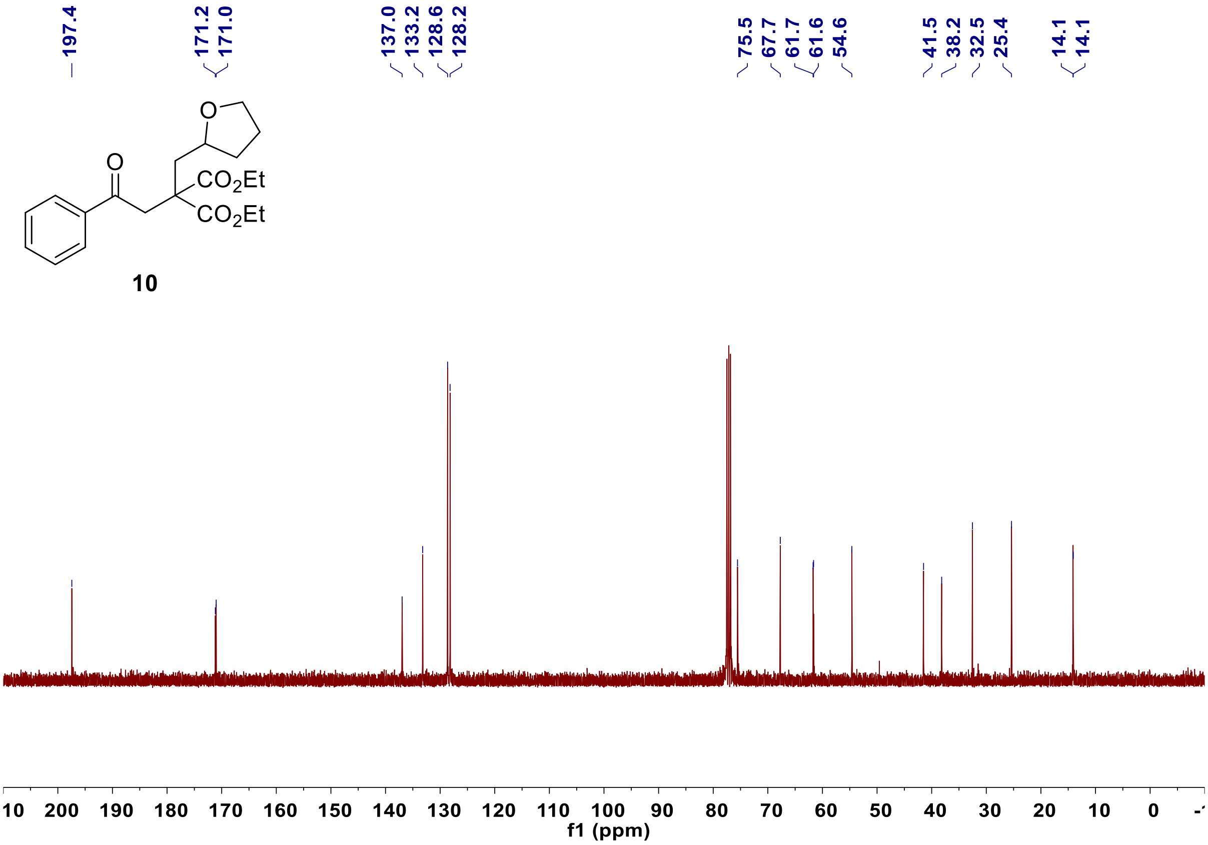

^{1}H and ^{13}C NMR spectra for compound 11

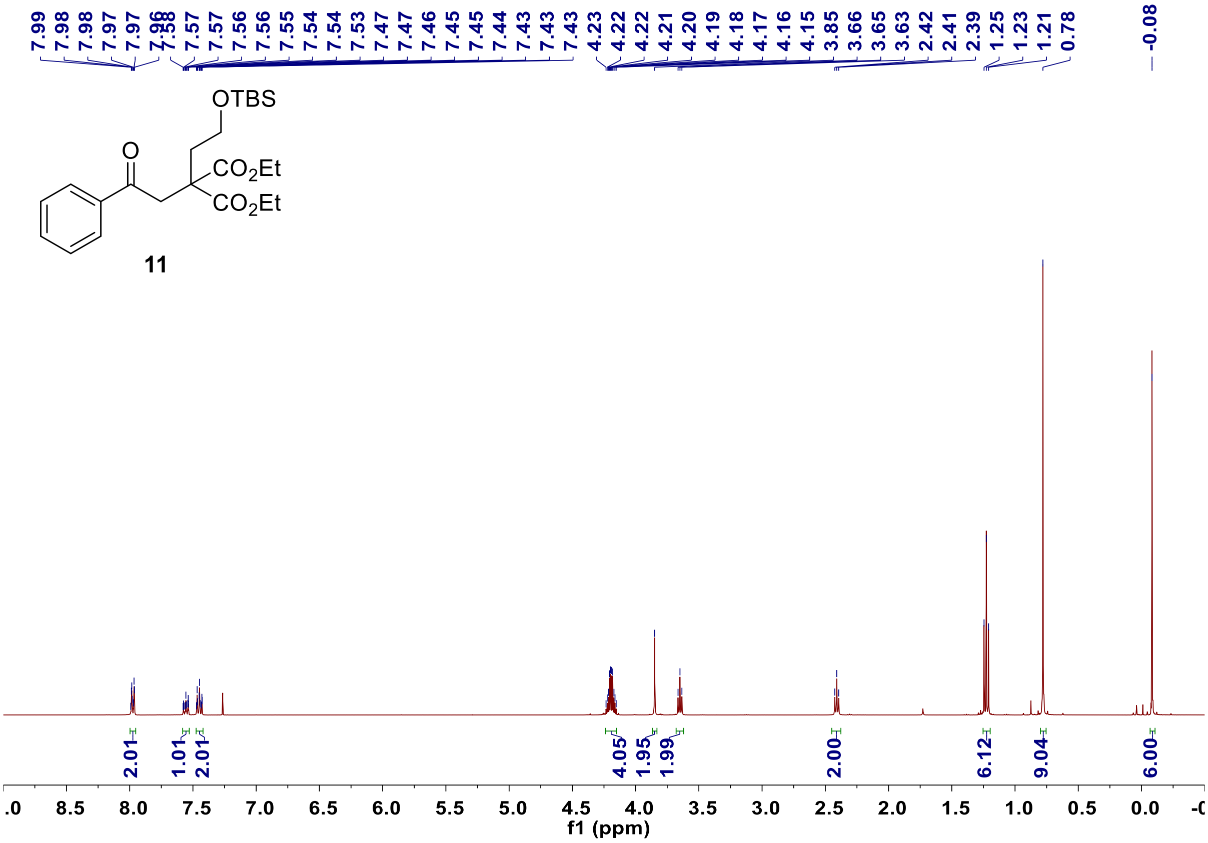

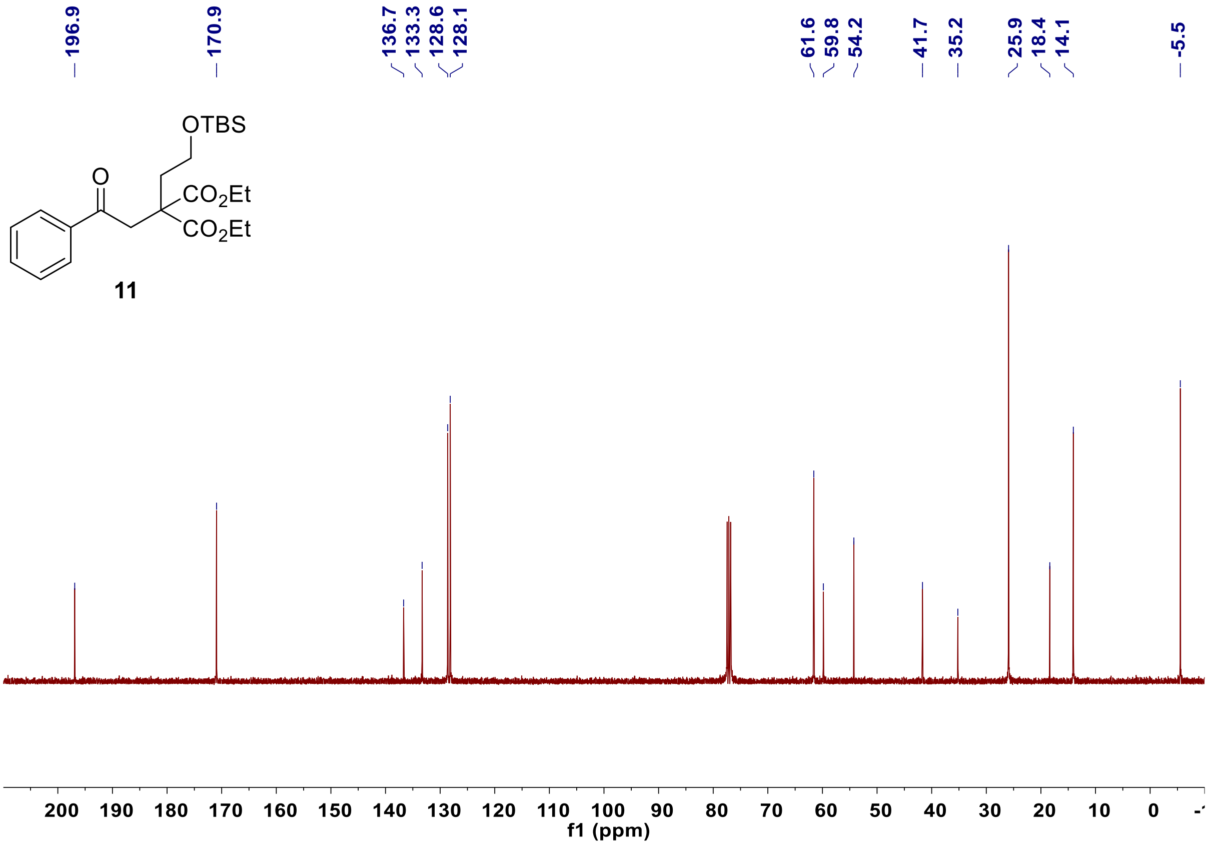

^{1}H and ^{13}C NMR spectra for compound 12

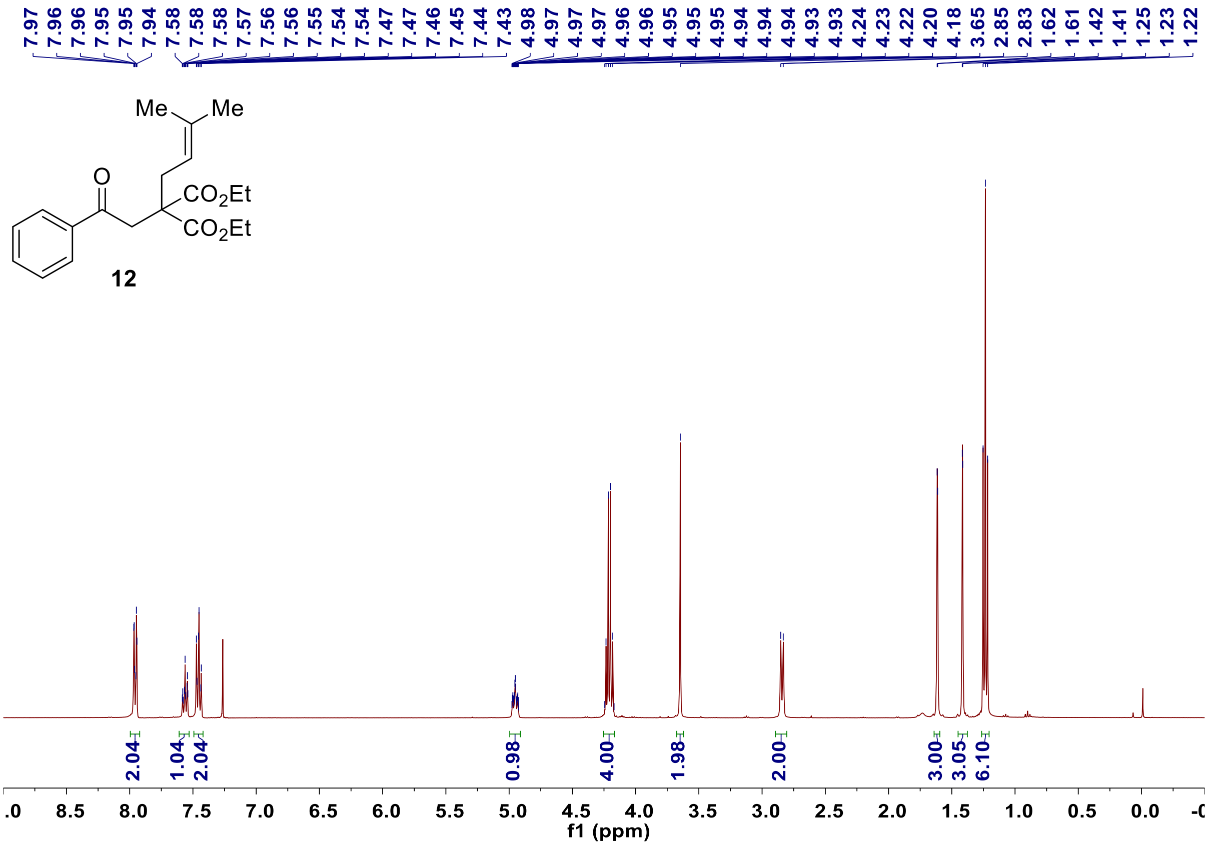

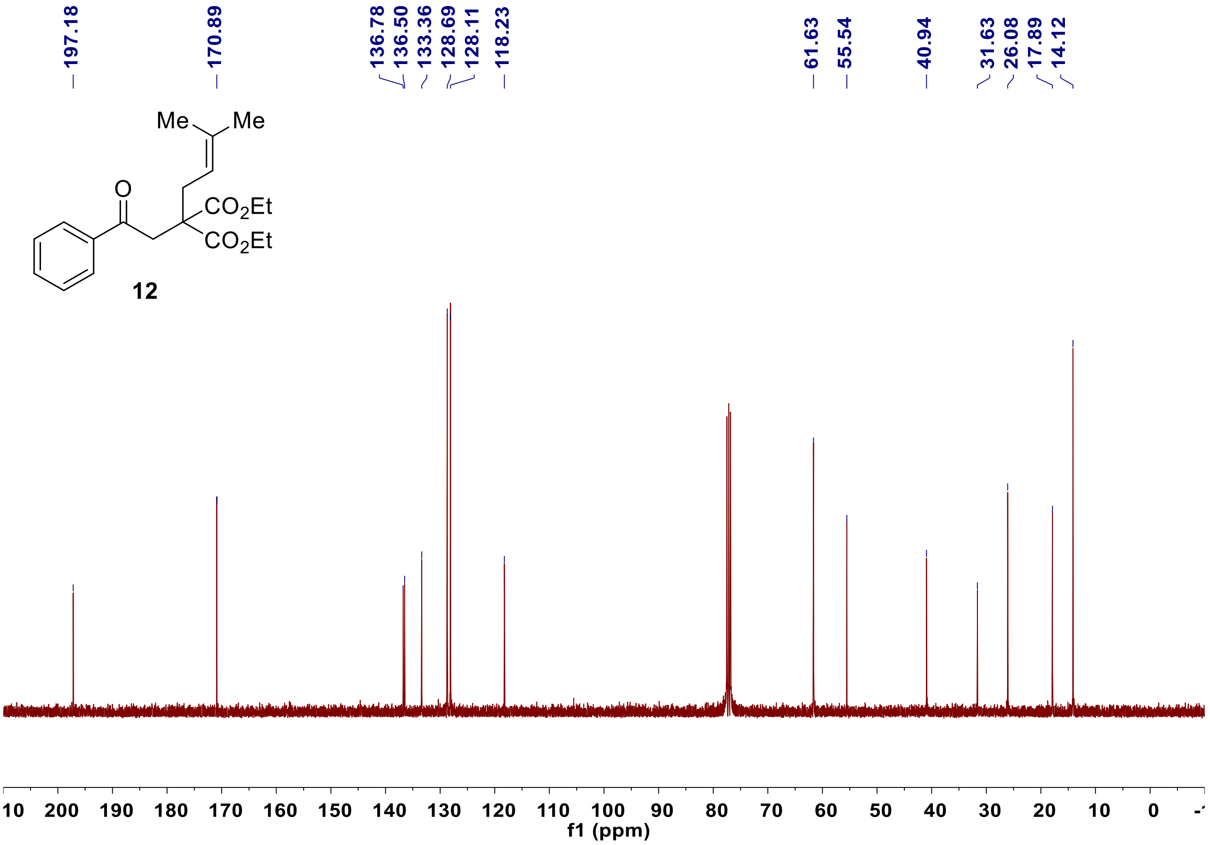

^{1}H and ^{13}C NMR spectra for compound 13

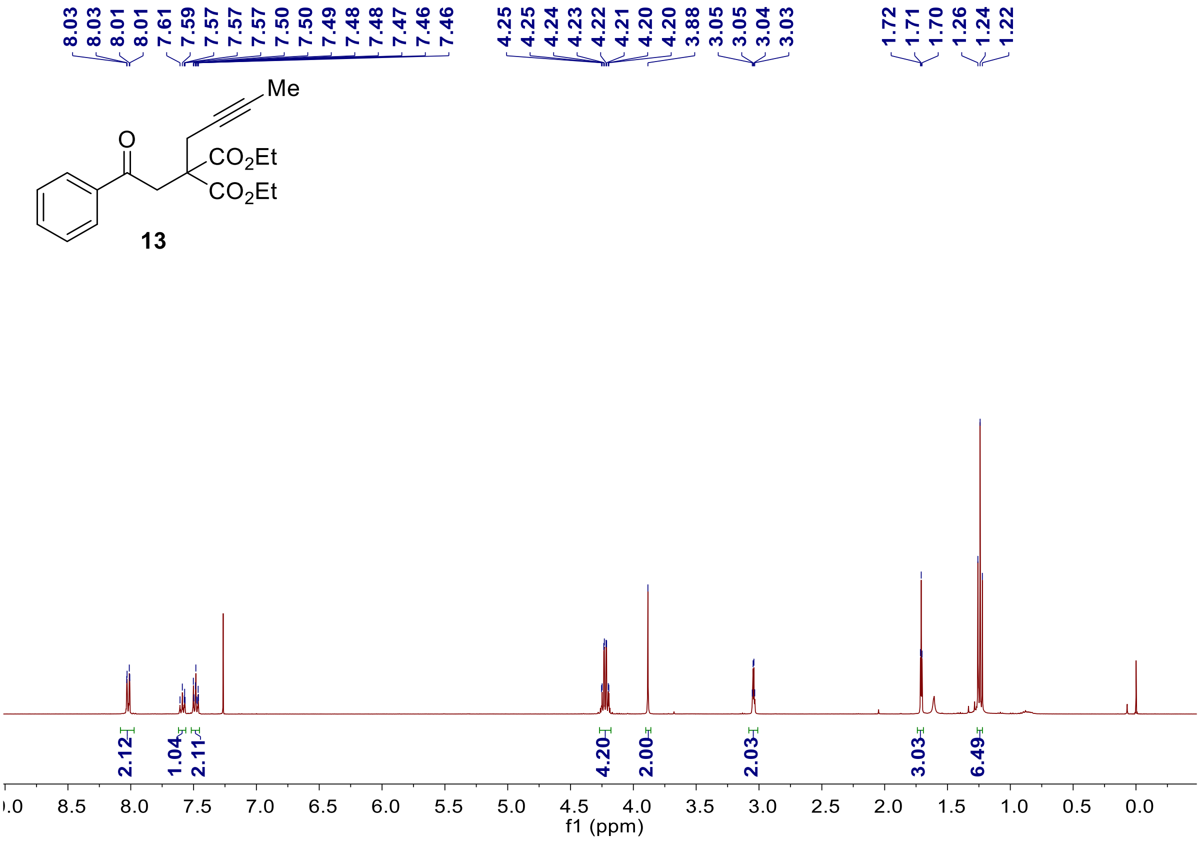

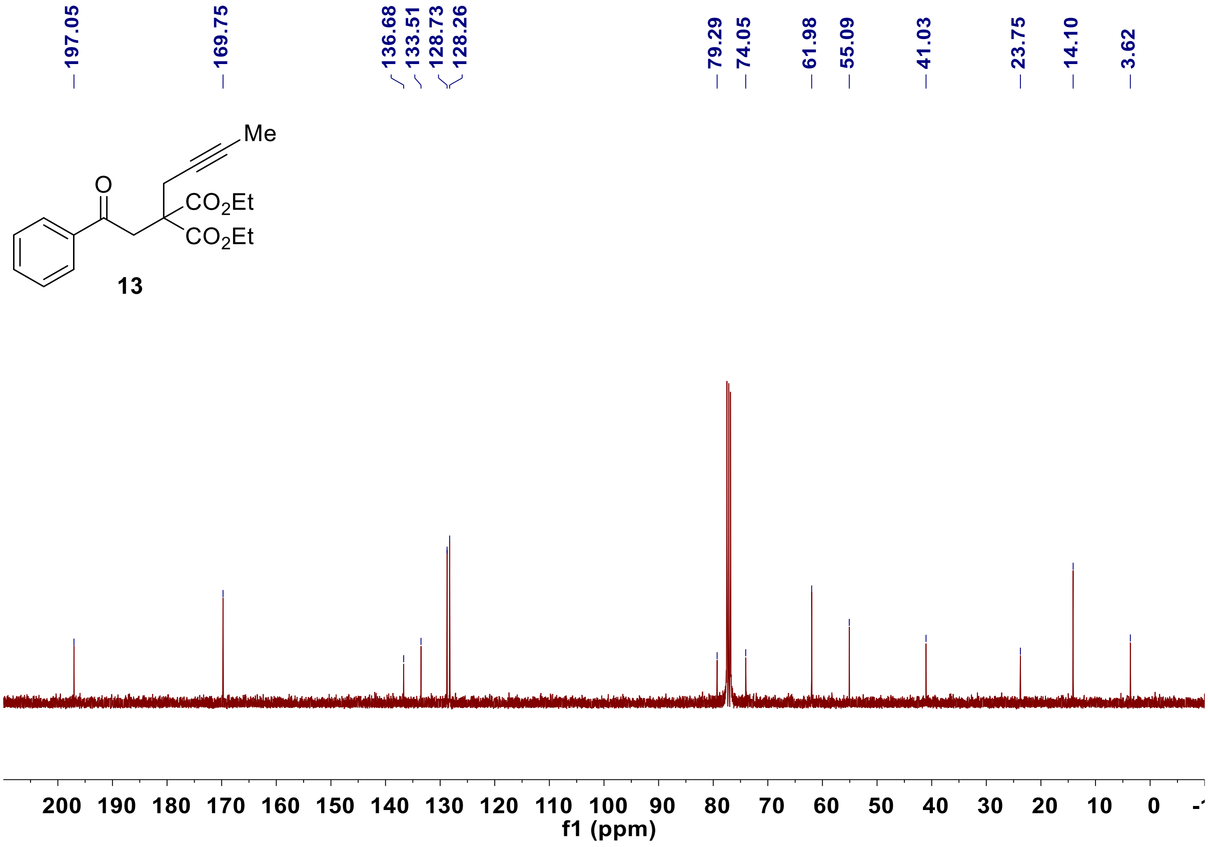

^{1}H and ^{13}C NMR spectra for compound 14

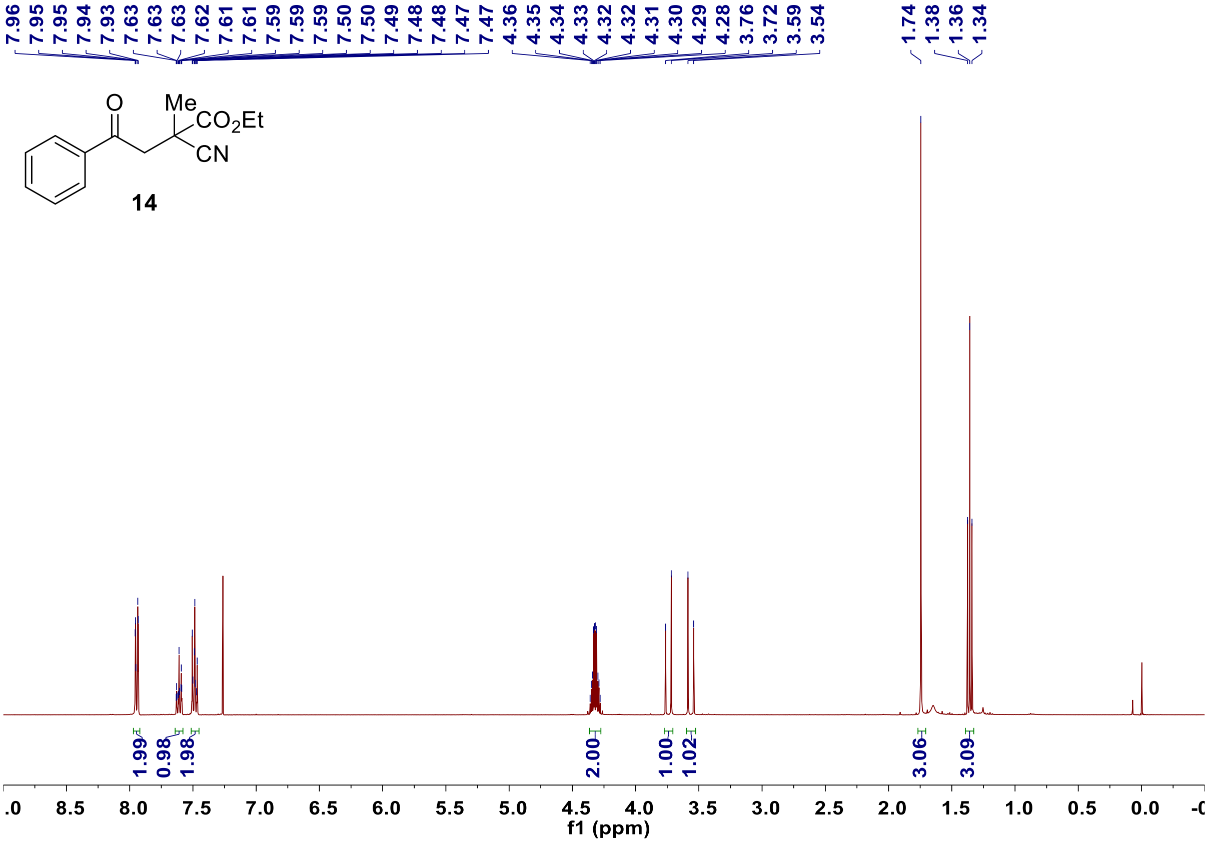

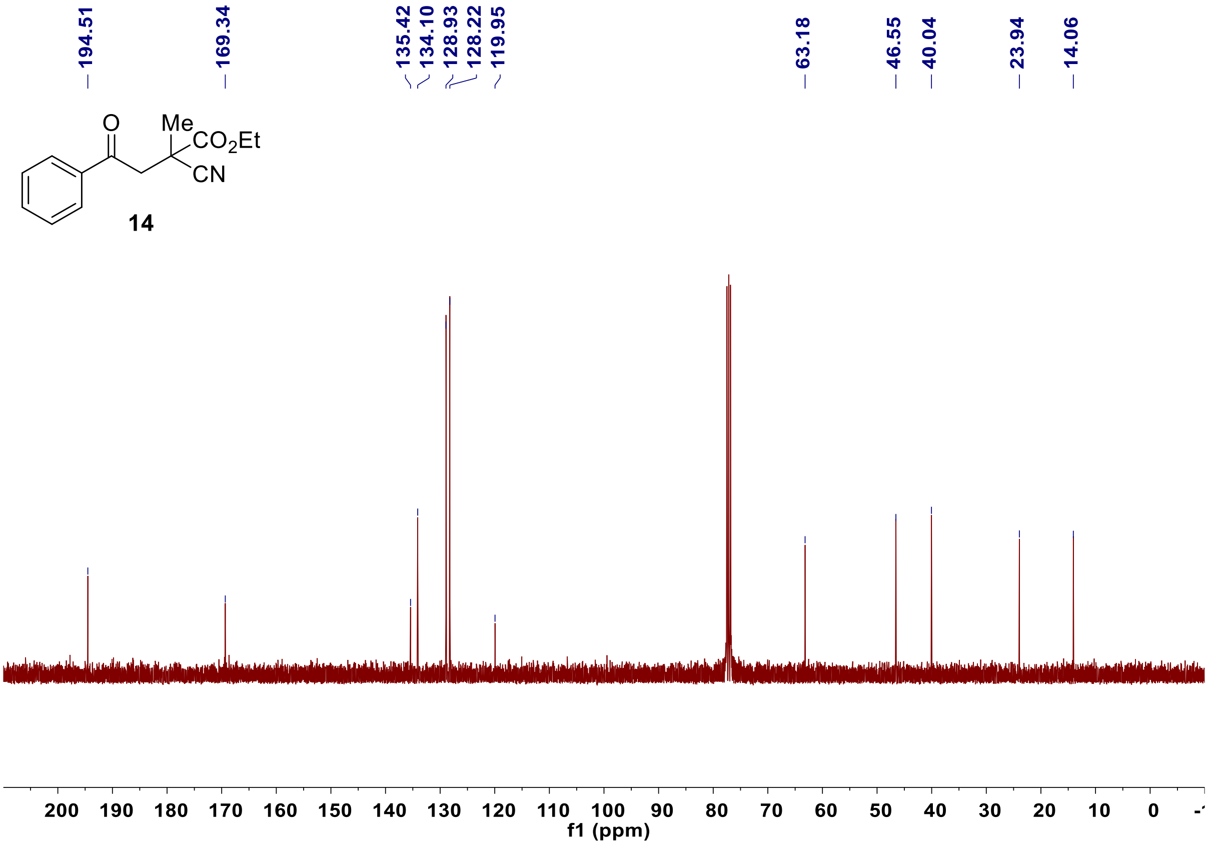

^{1}H and ^{13}C NMR spectra for compound 15

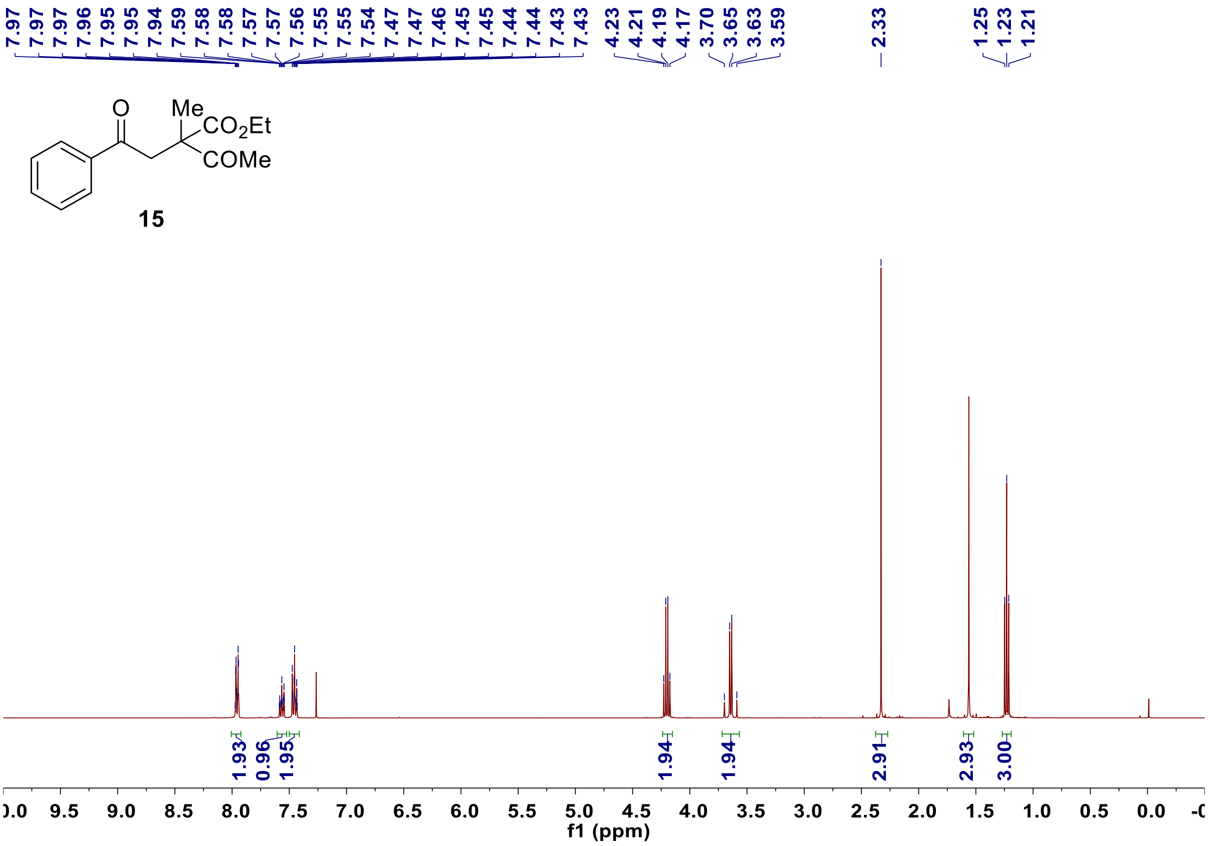

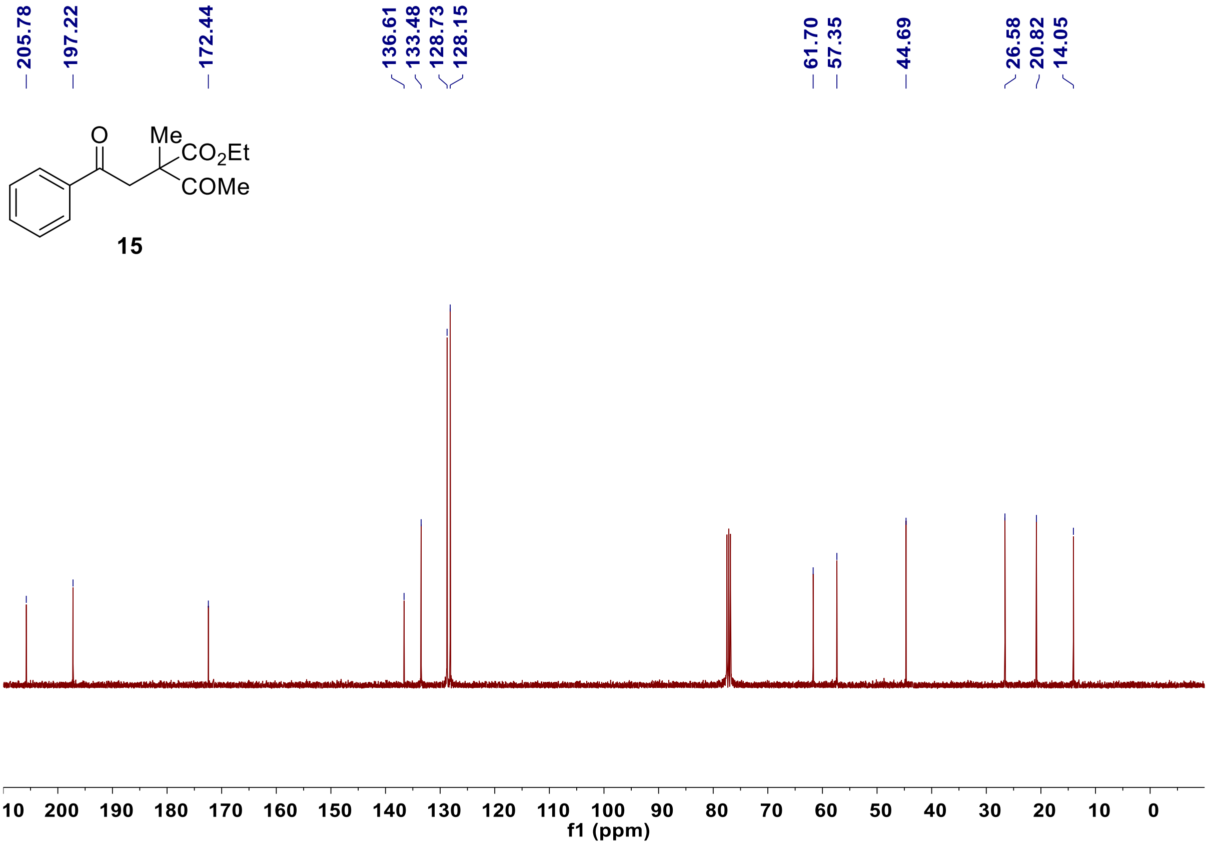

^{1}H and ^{13}C NMR spectra for compound 16

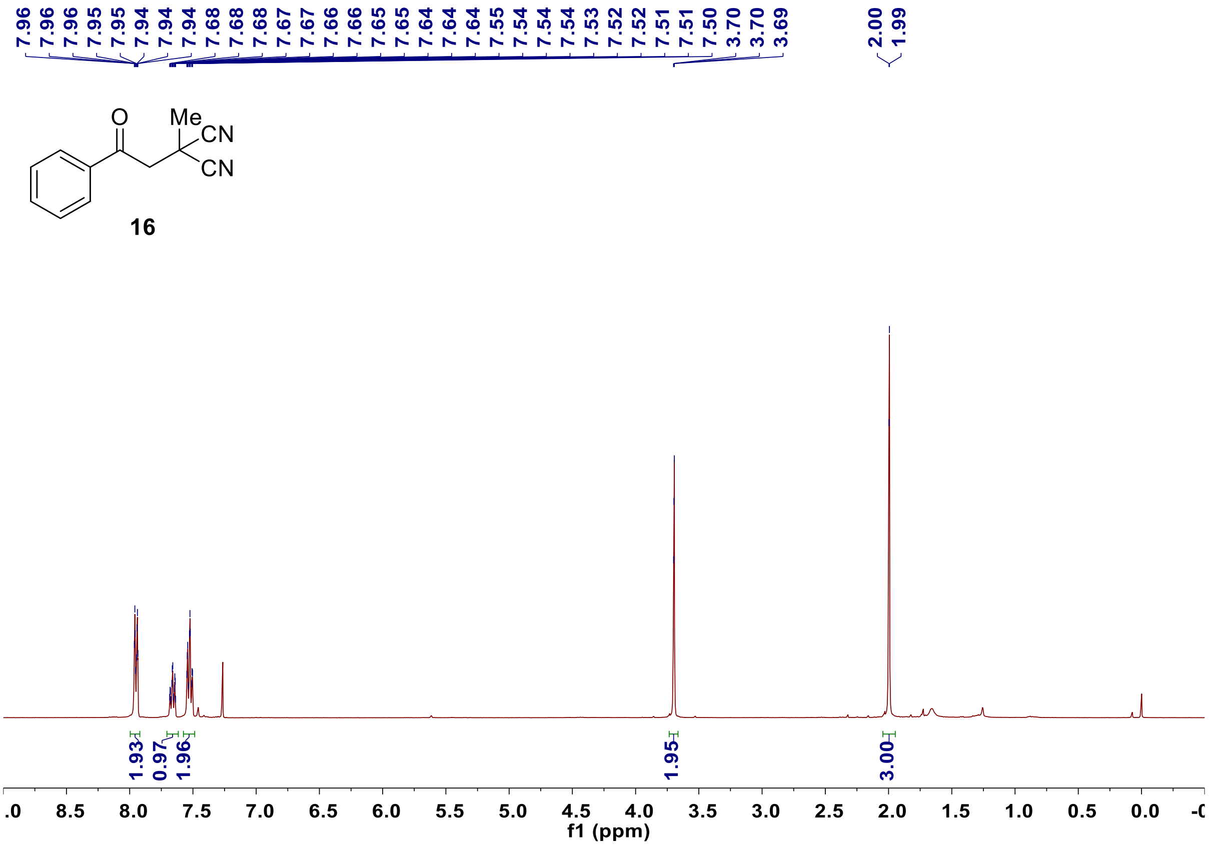

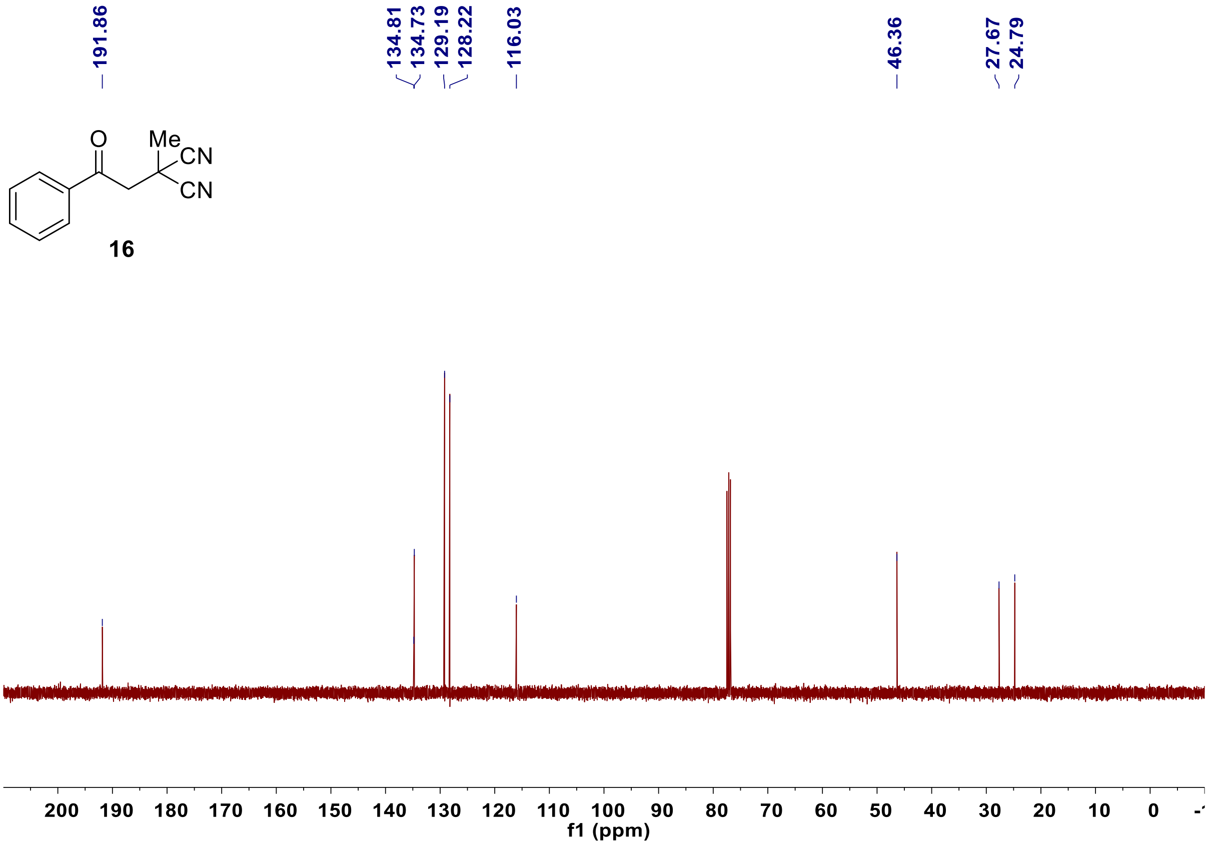

^{1}H and ^{13}C NMR spectra for compound 17

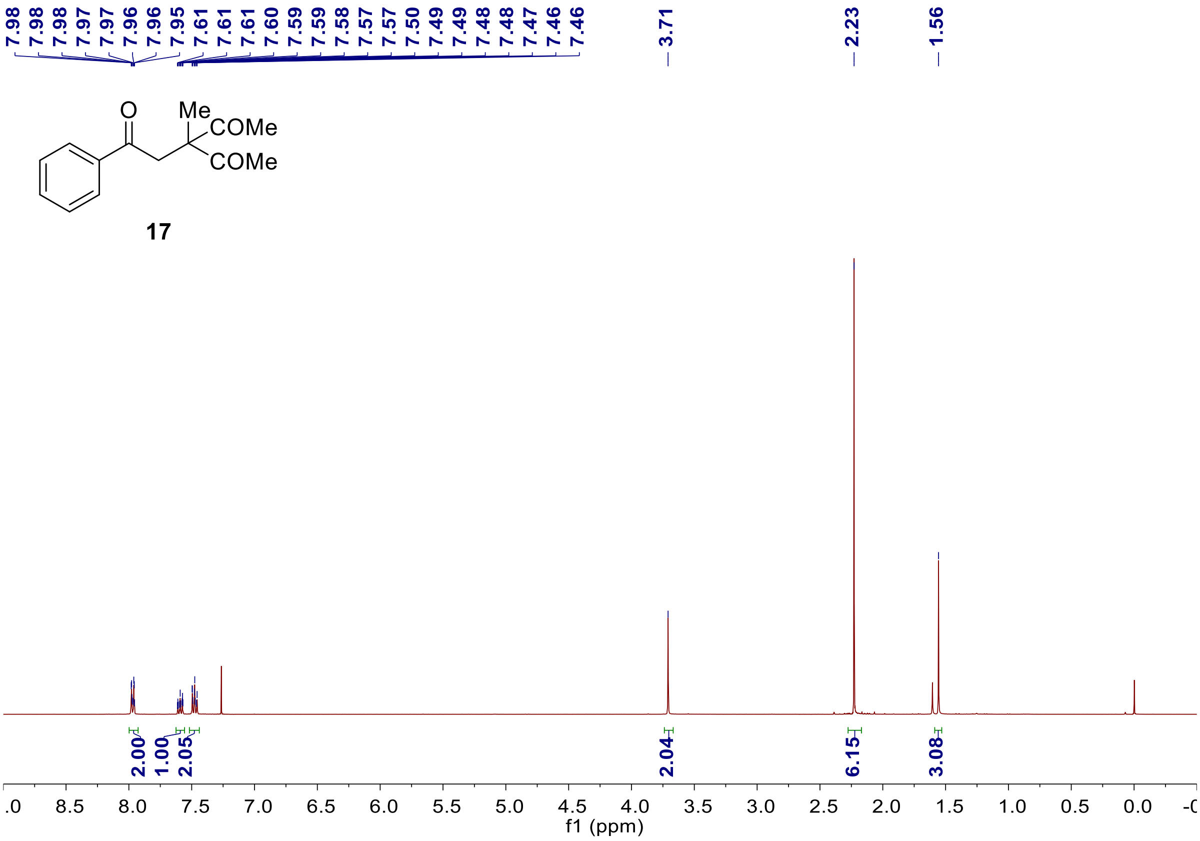

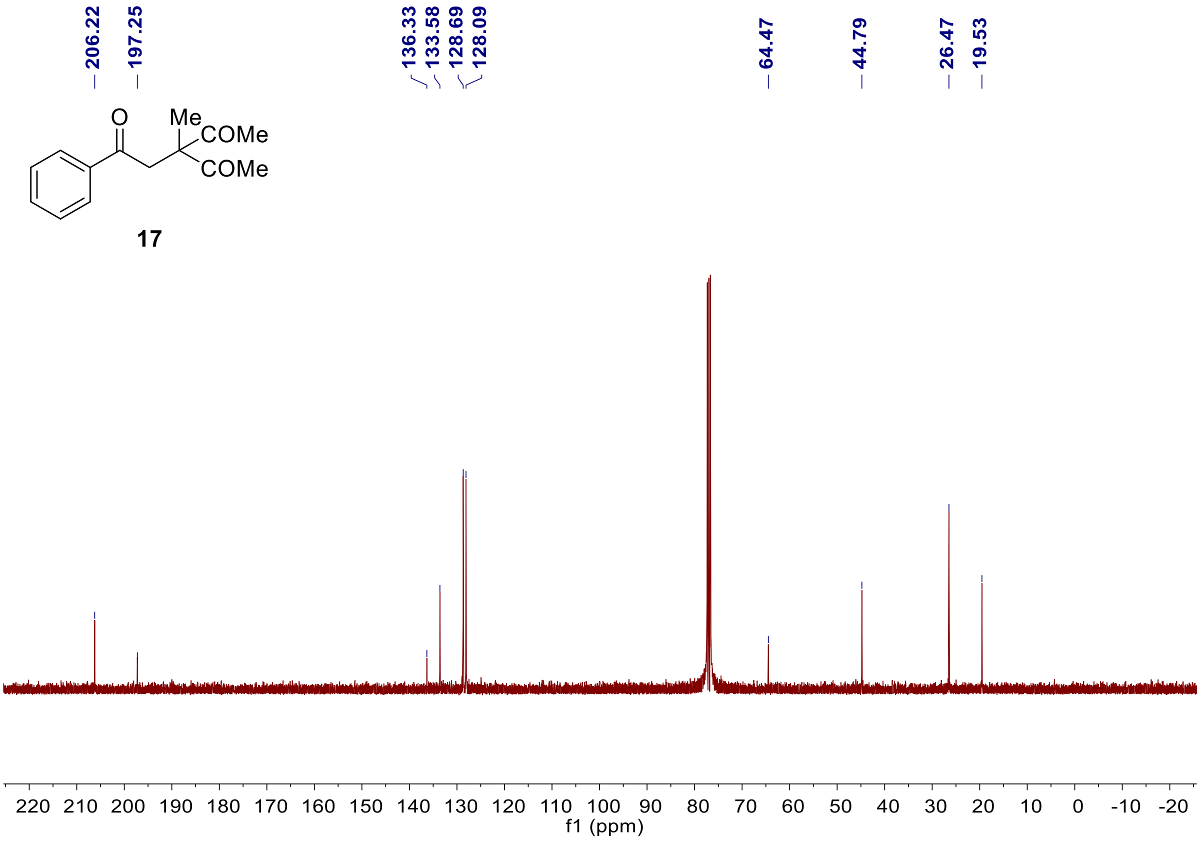

^{1}H and ^{13}C NMR spectra for compound 18

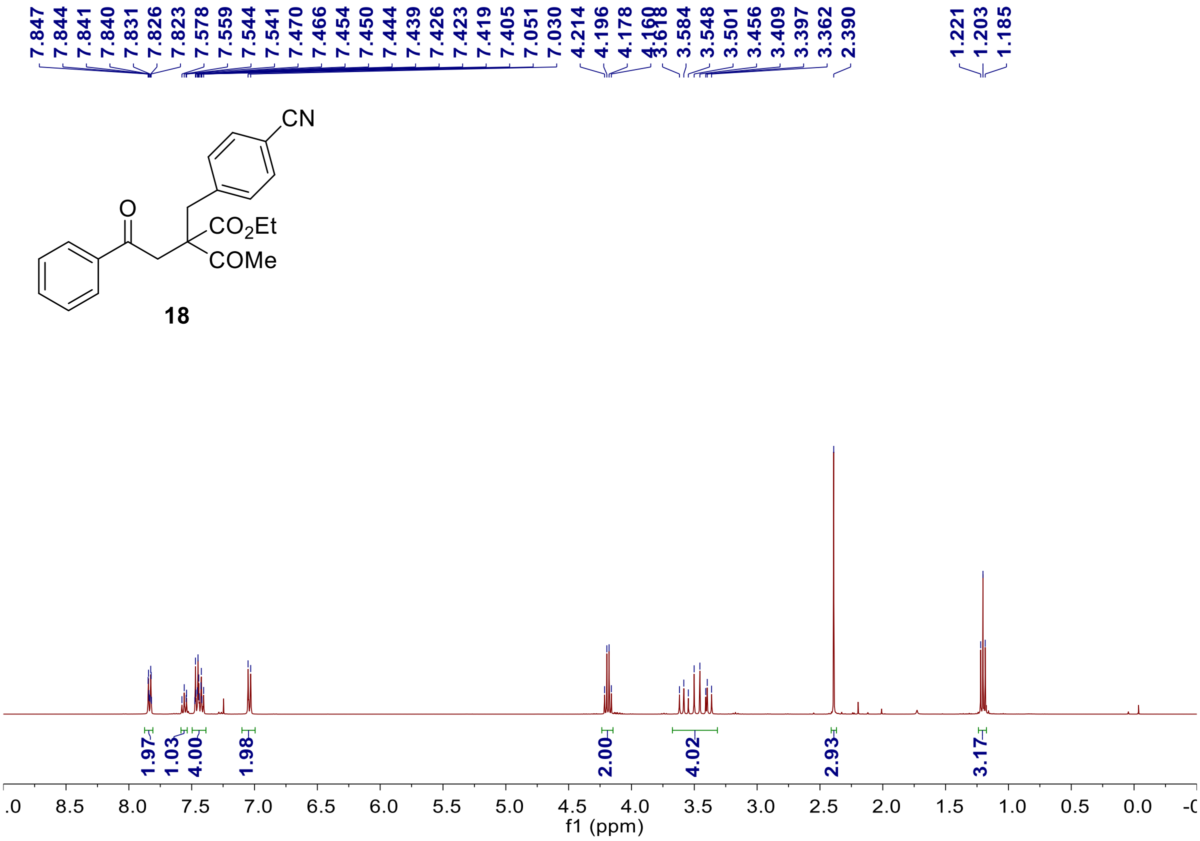

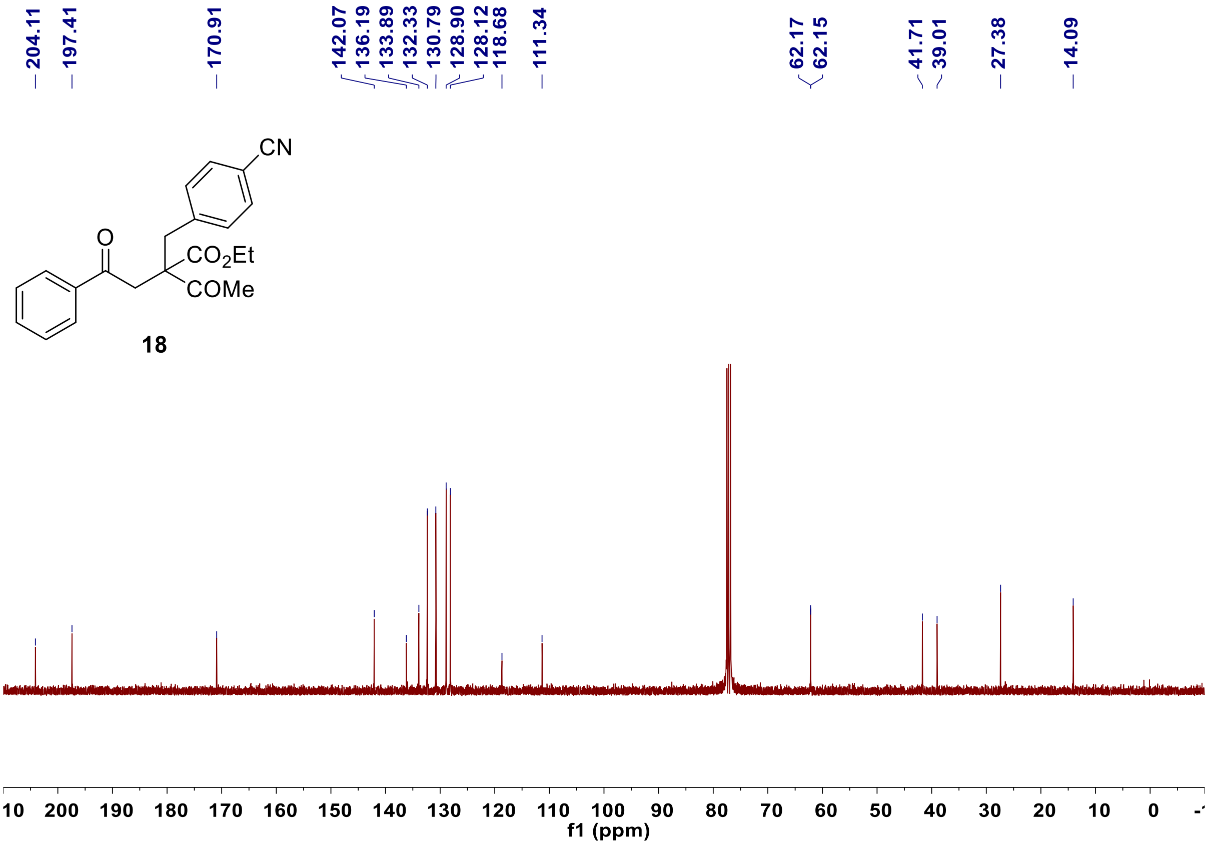

^{1}H and ^{13}C NMR spectra for compound 19

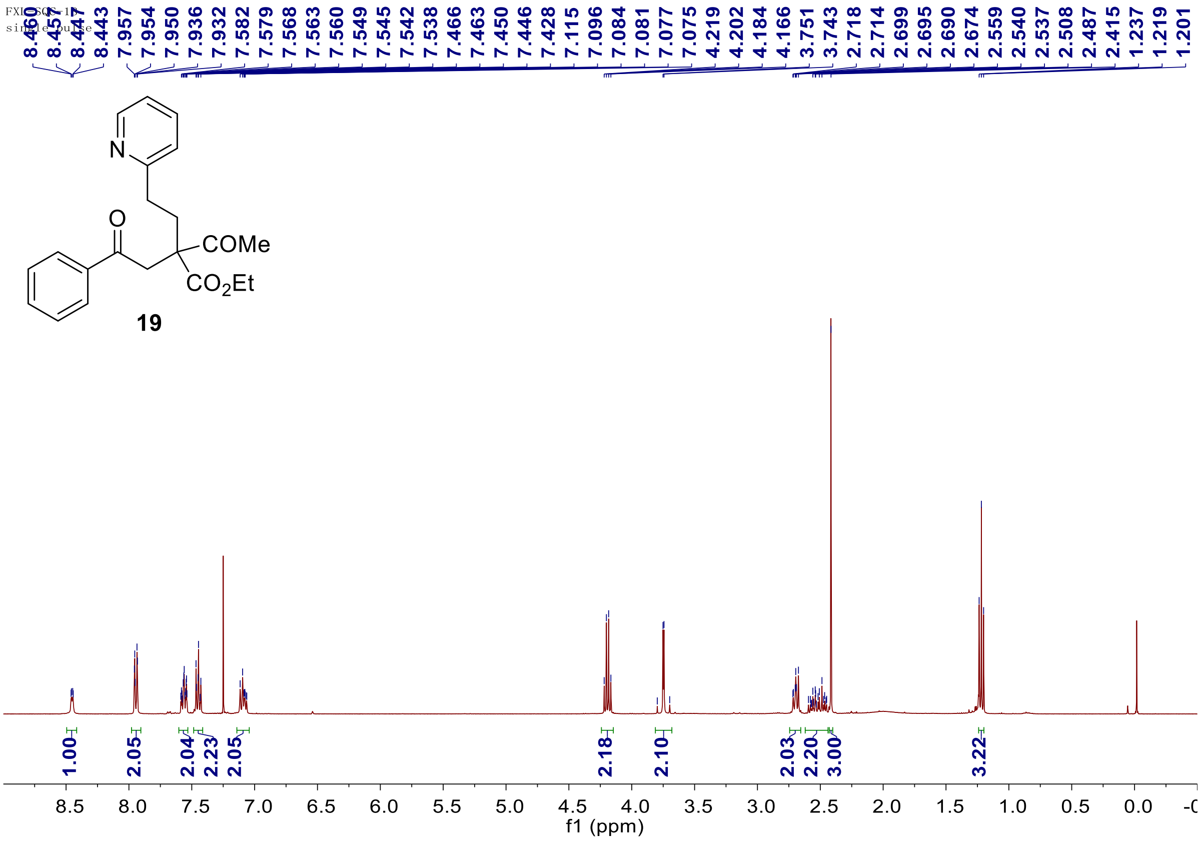

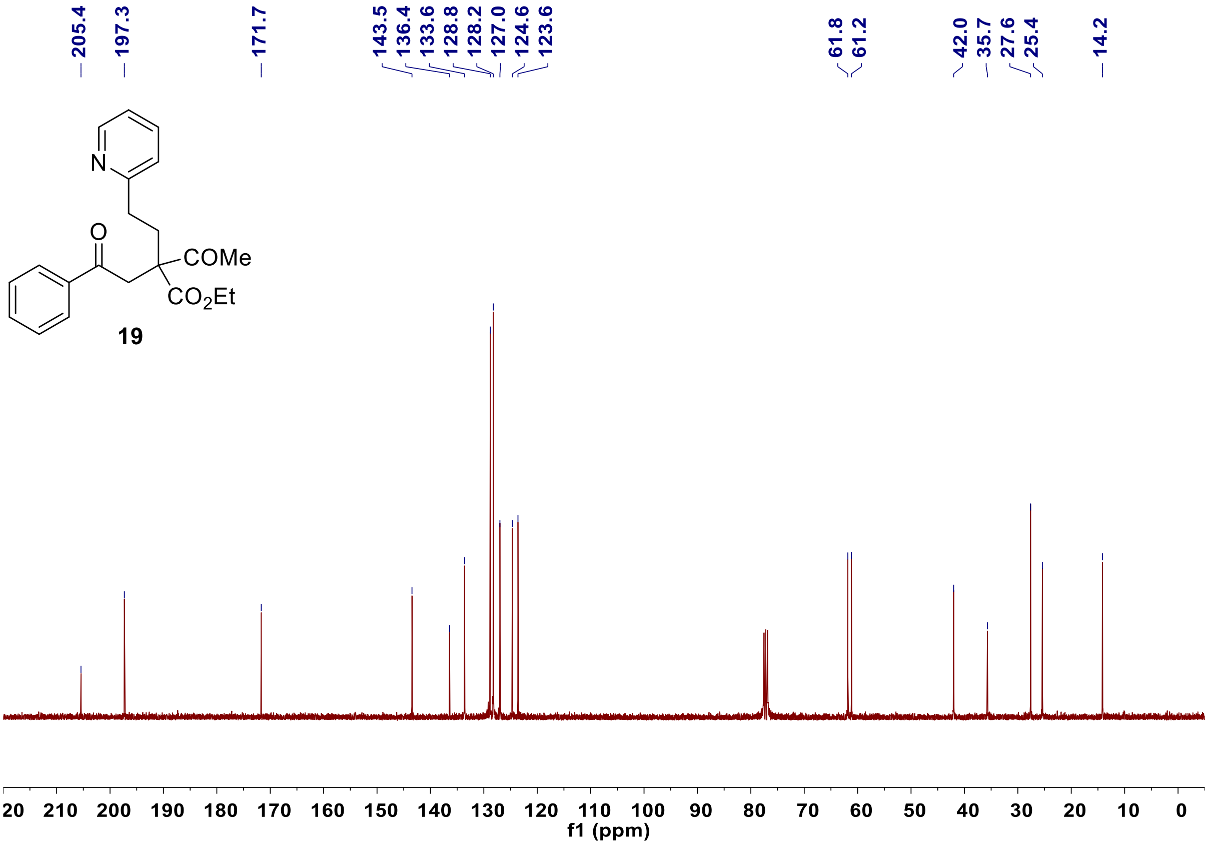

^{1}H and ^{13}C NMR spectra for compound 20

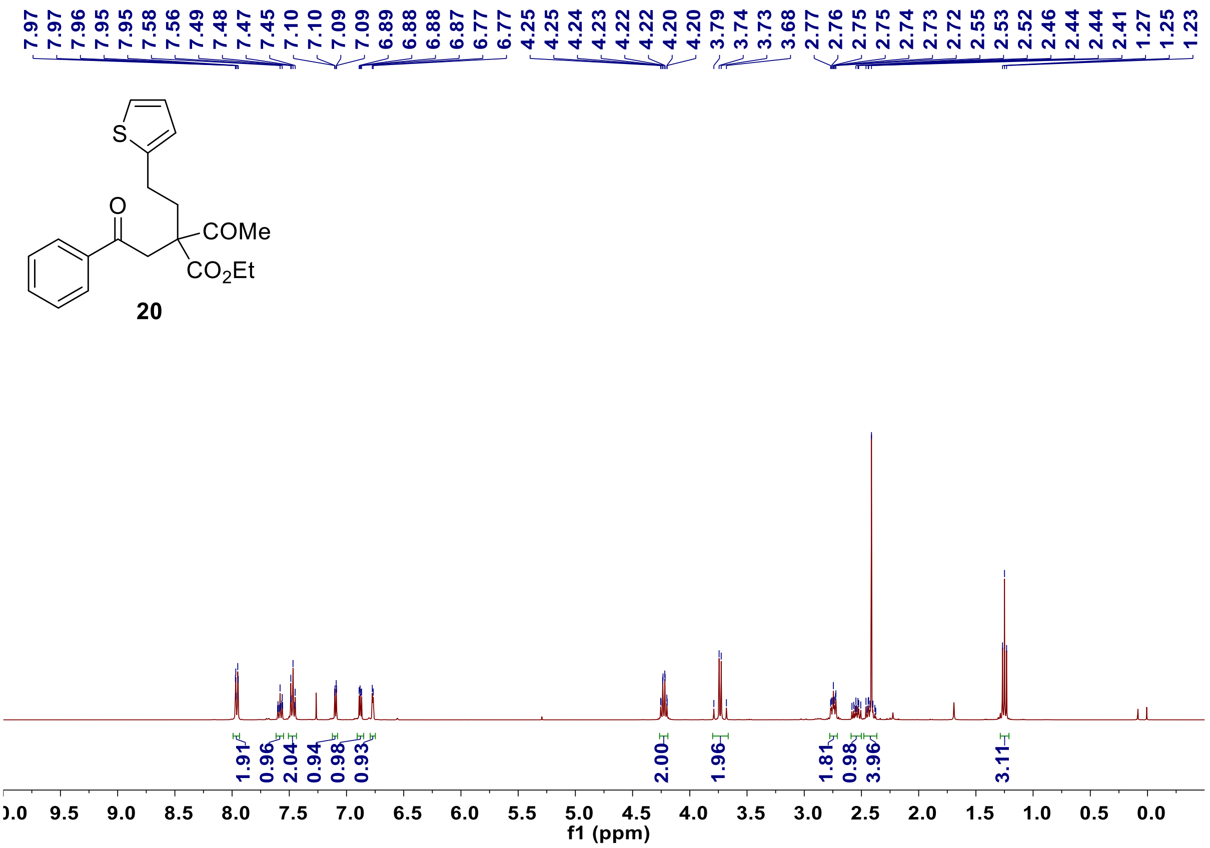

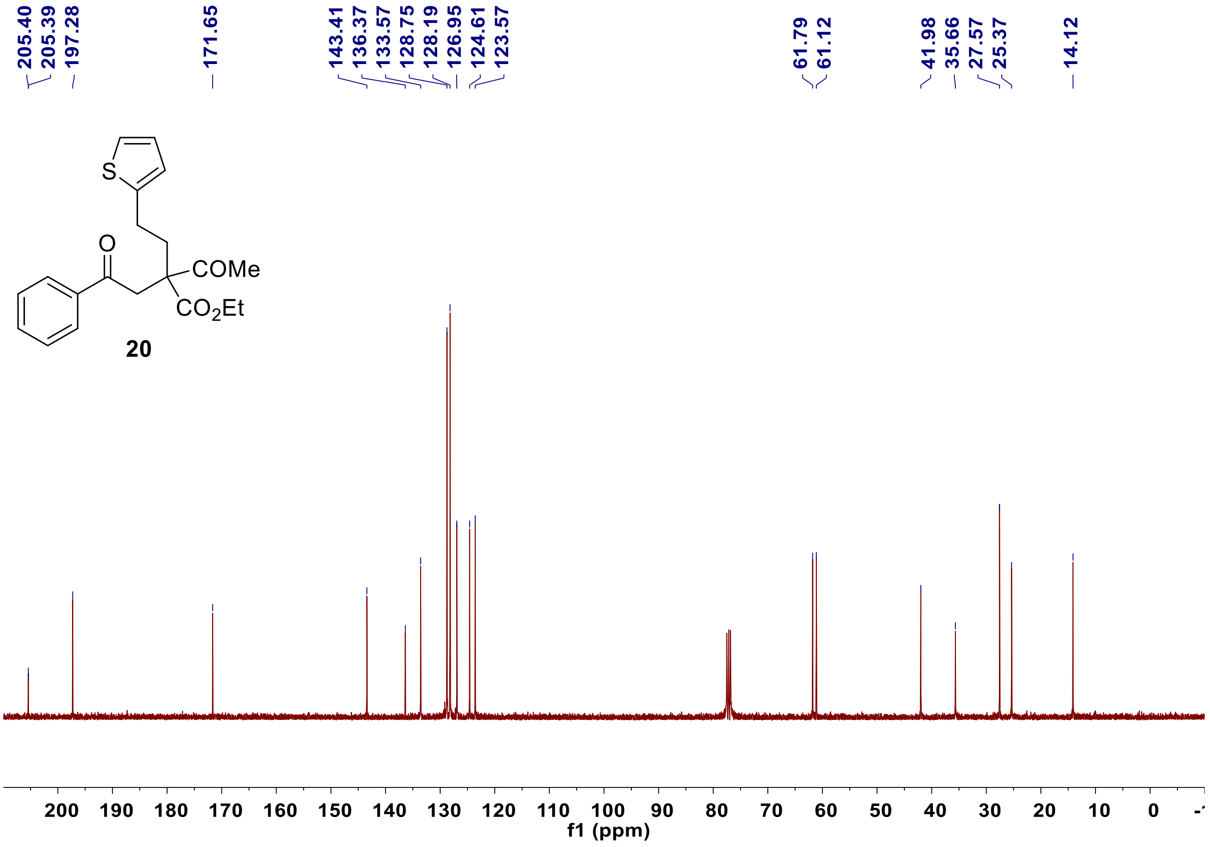

^{1}H and ^{13}C NMR spectra for compound 21

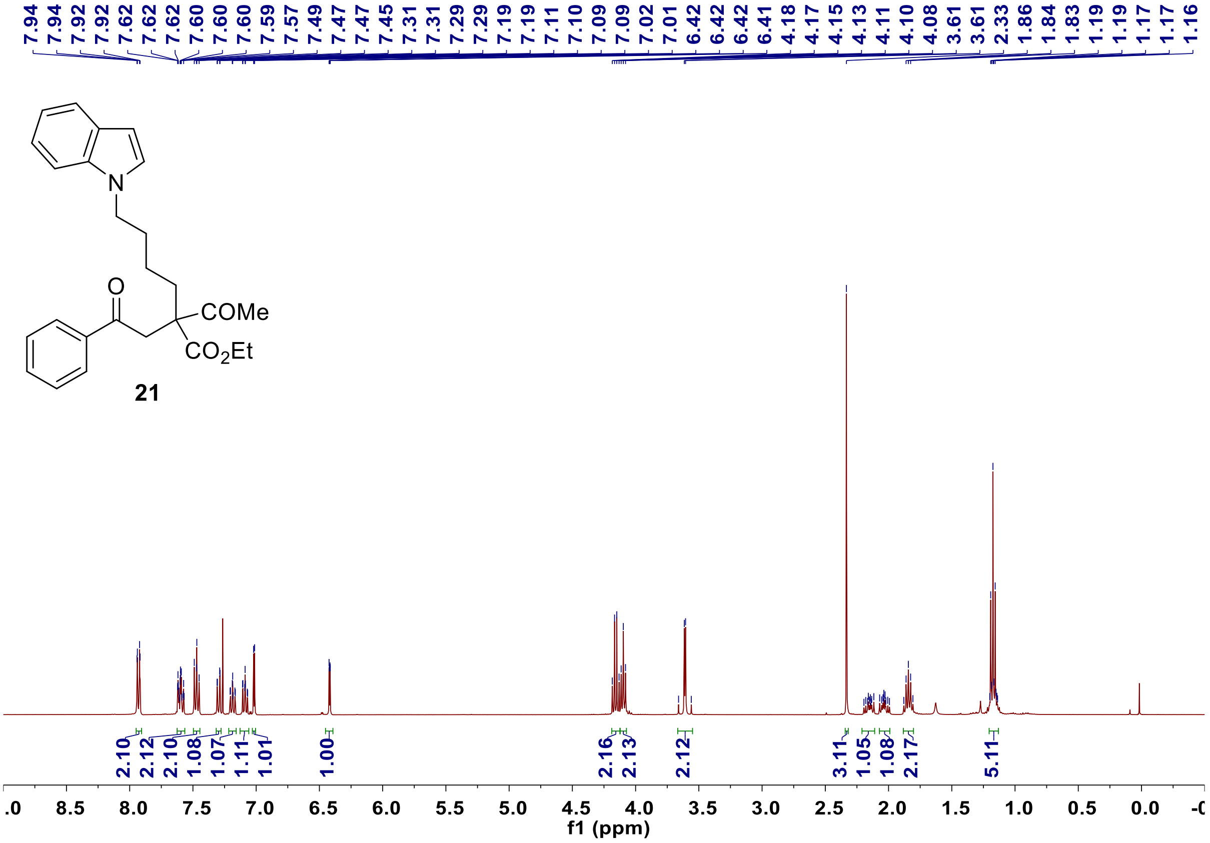

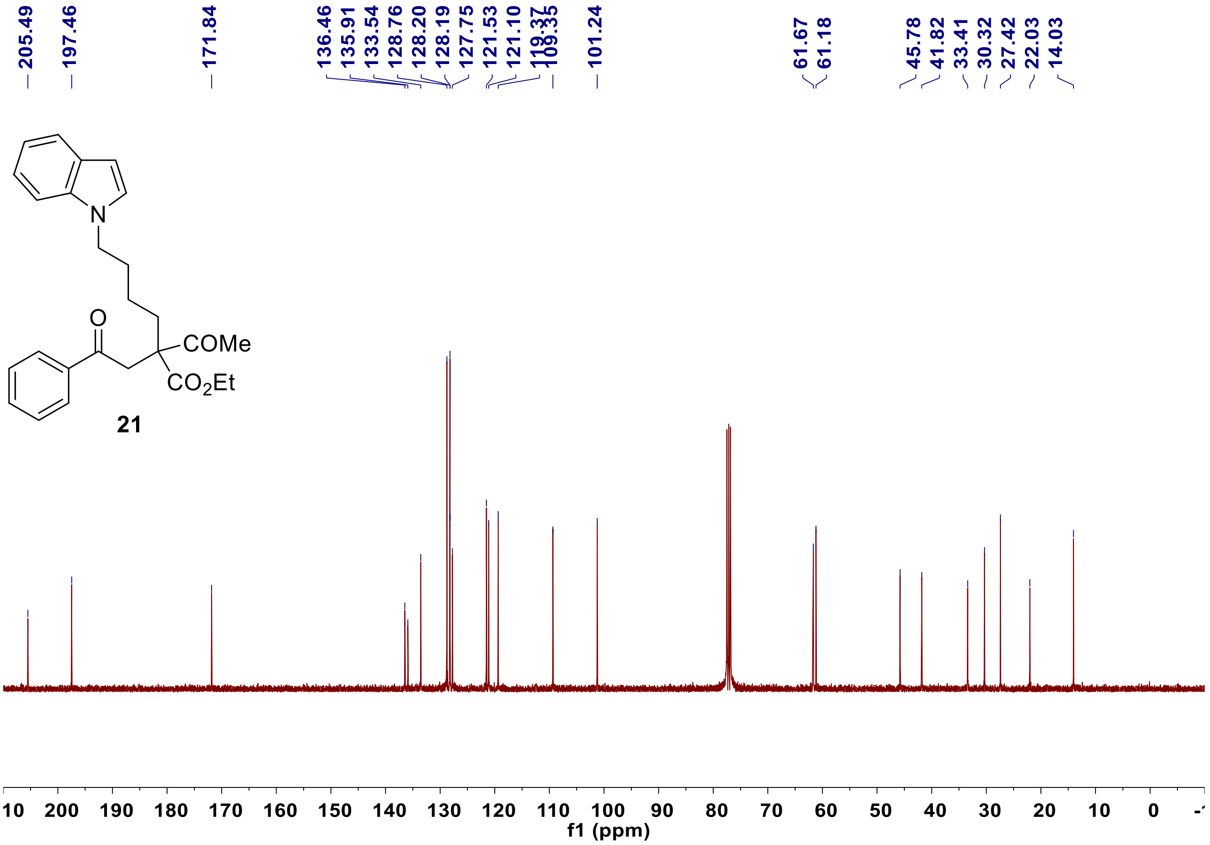

^{1}H, ^{19}F and ^{13}C NMR spectra for compound 22

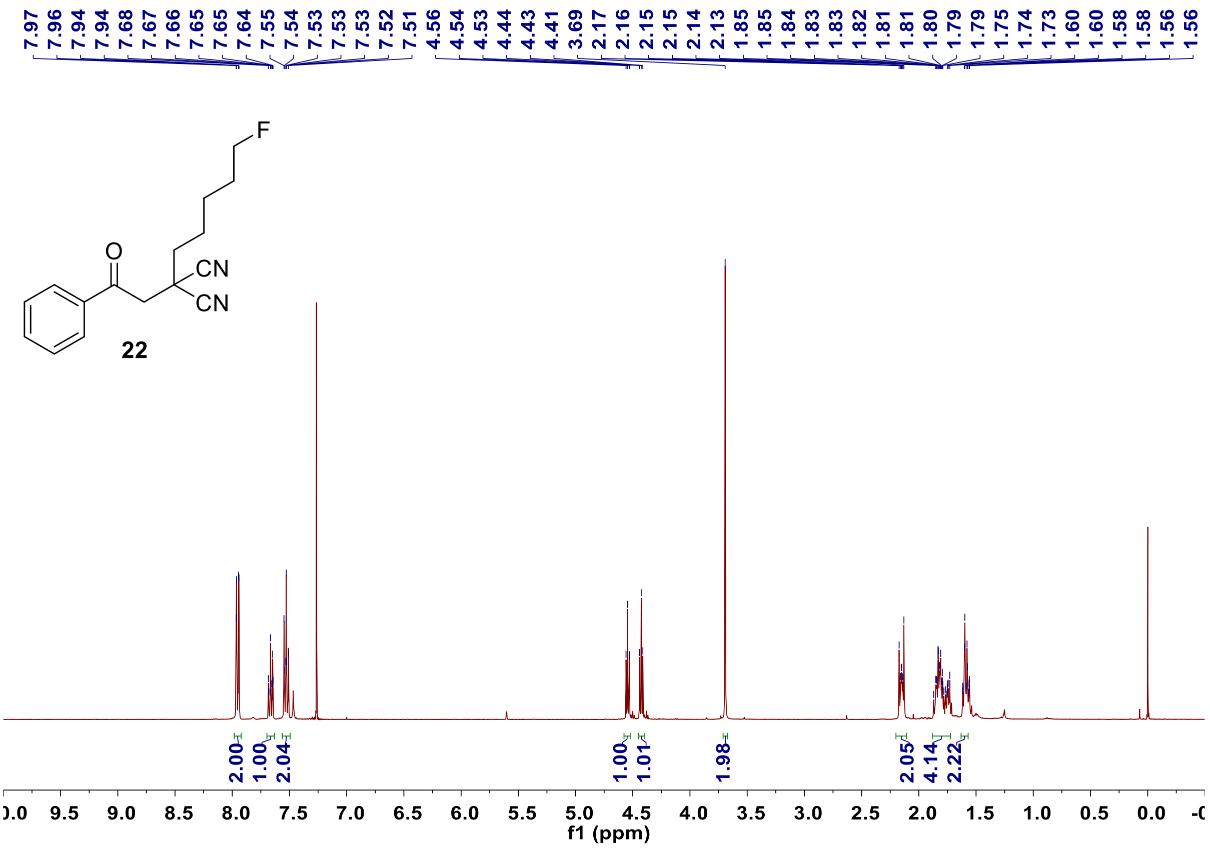

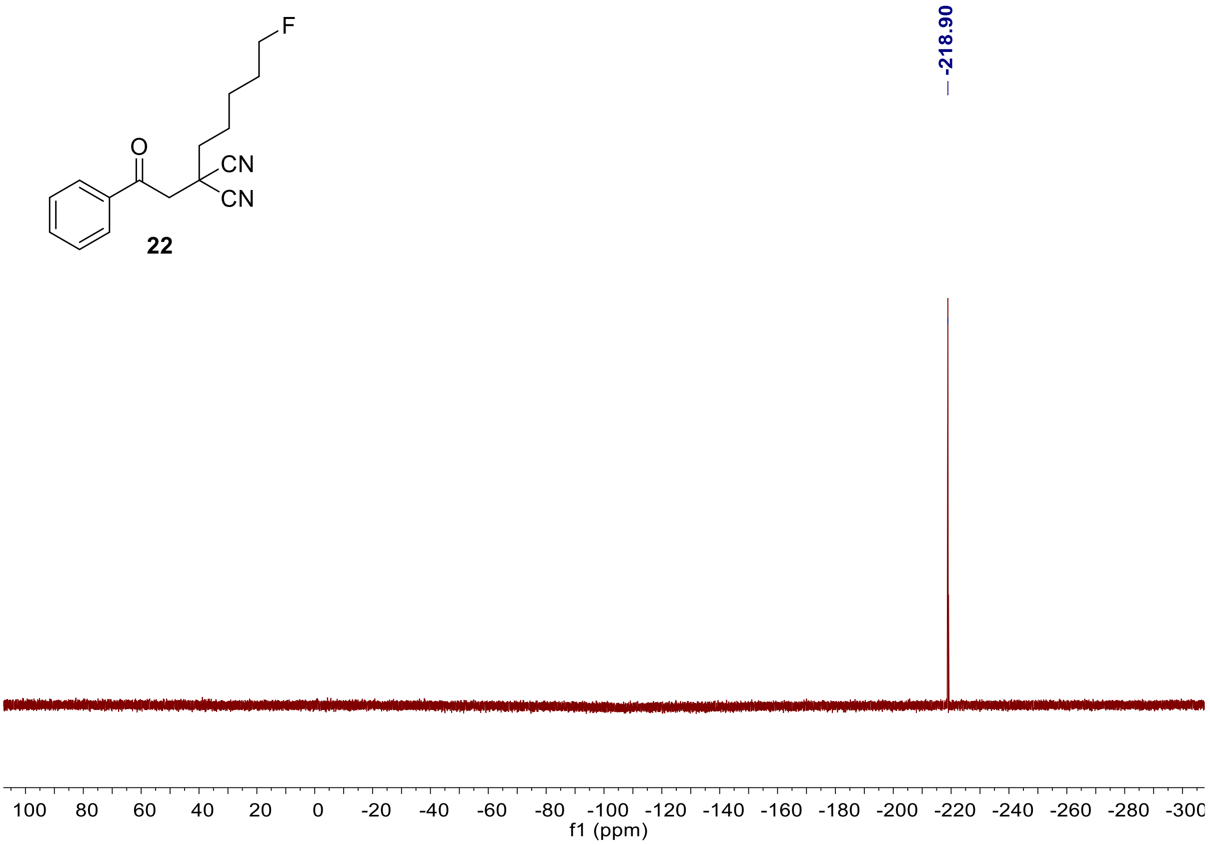

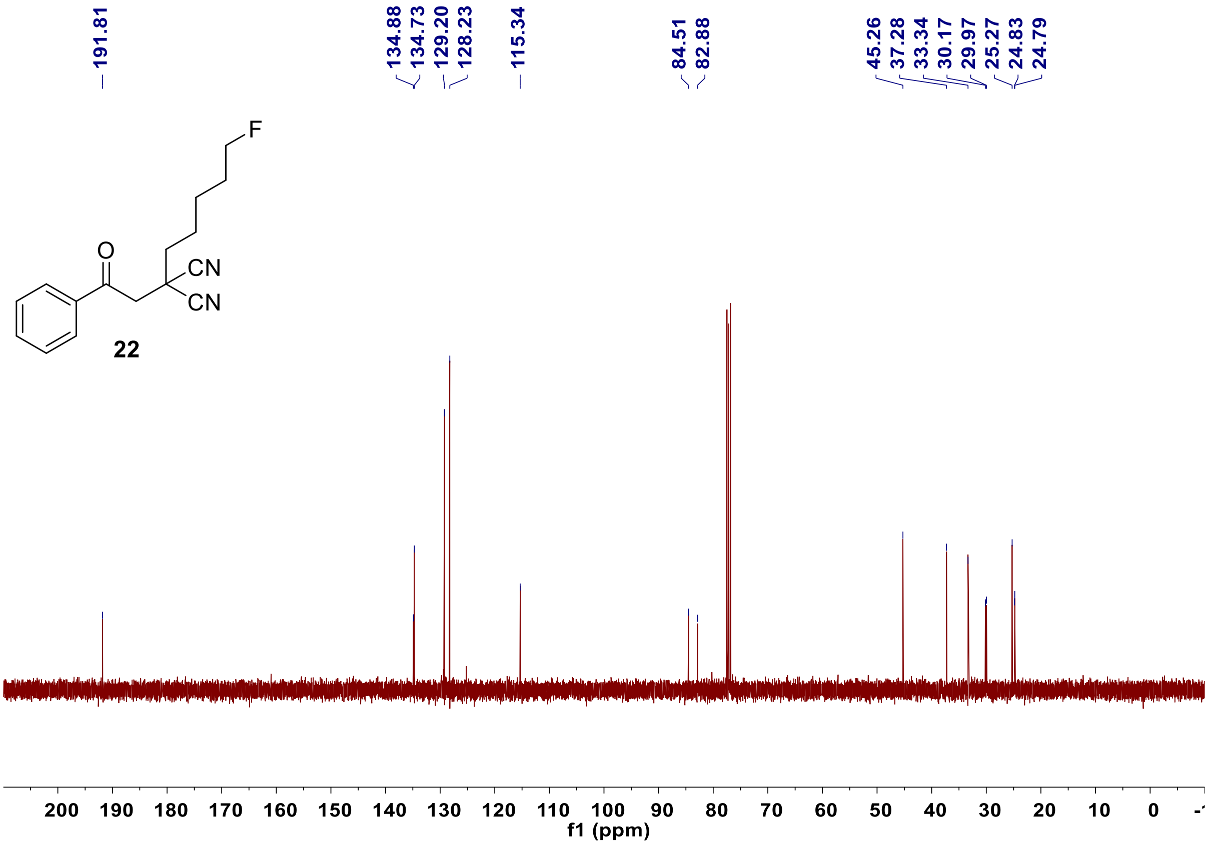

^{1}H and ^{13}C NMR spectra for compound 23

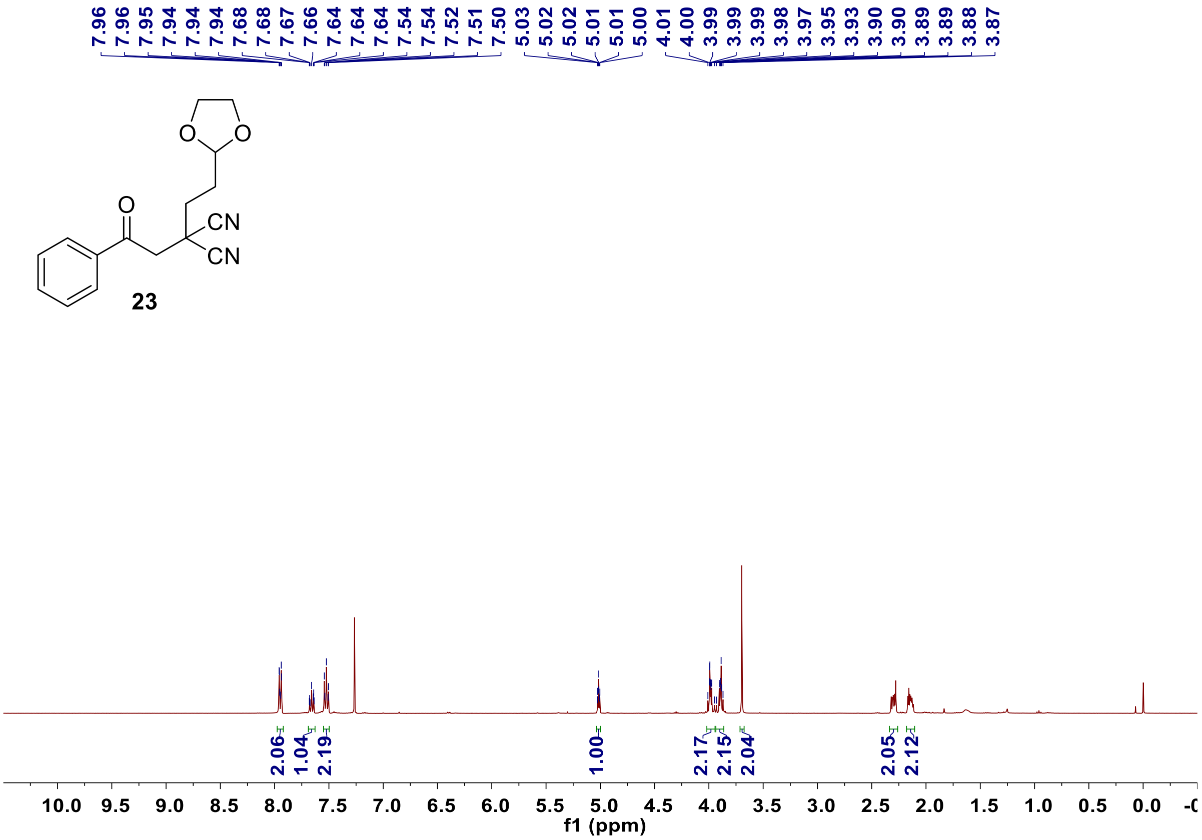

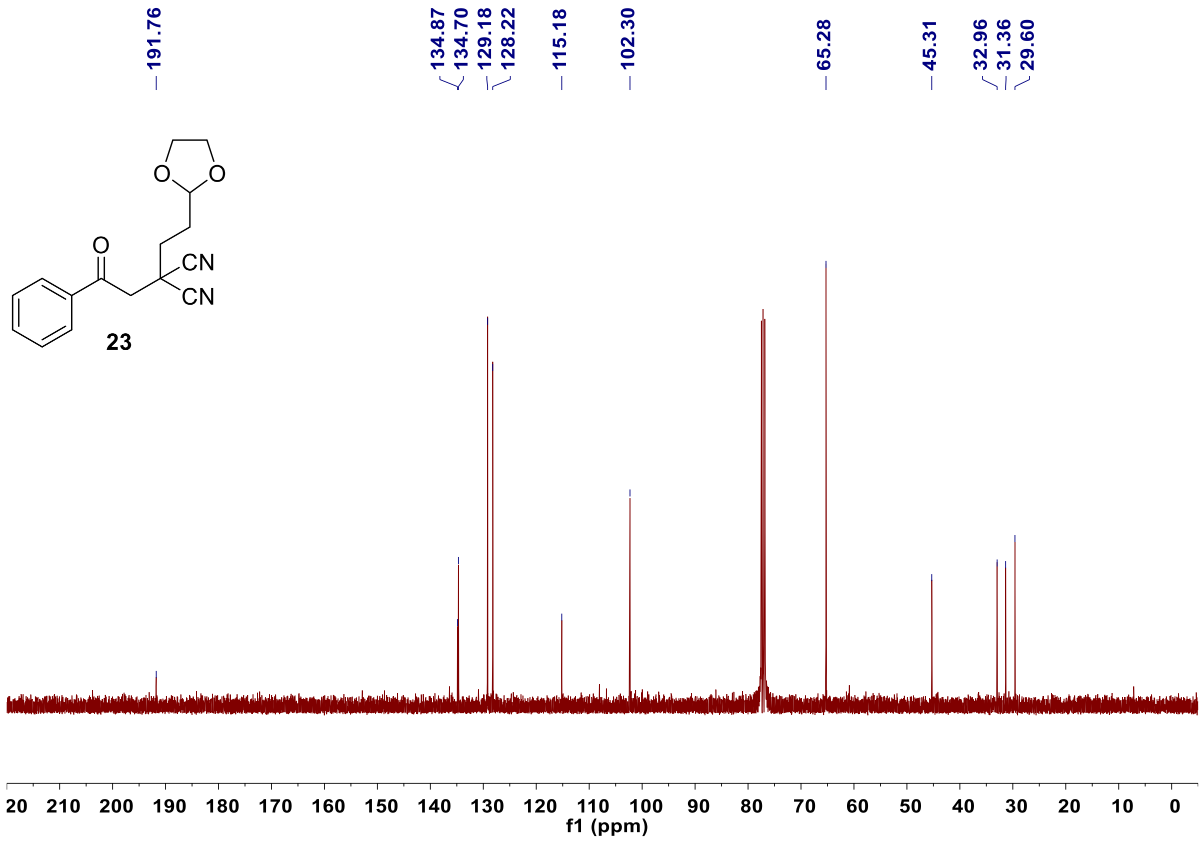

^1H and ^{13}C NMR spectra for compound 24

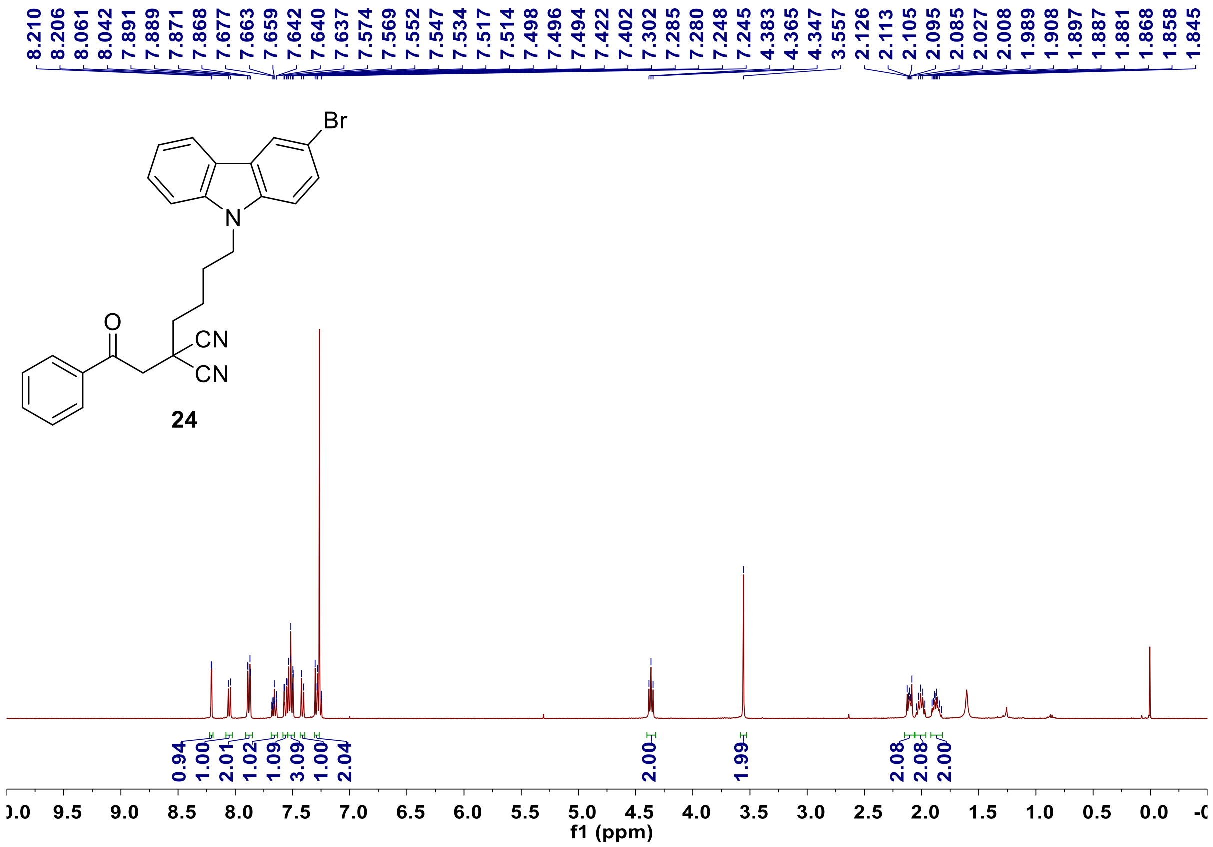

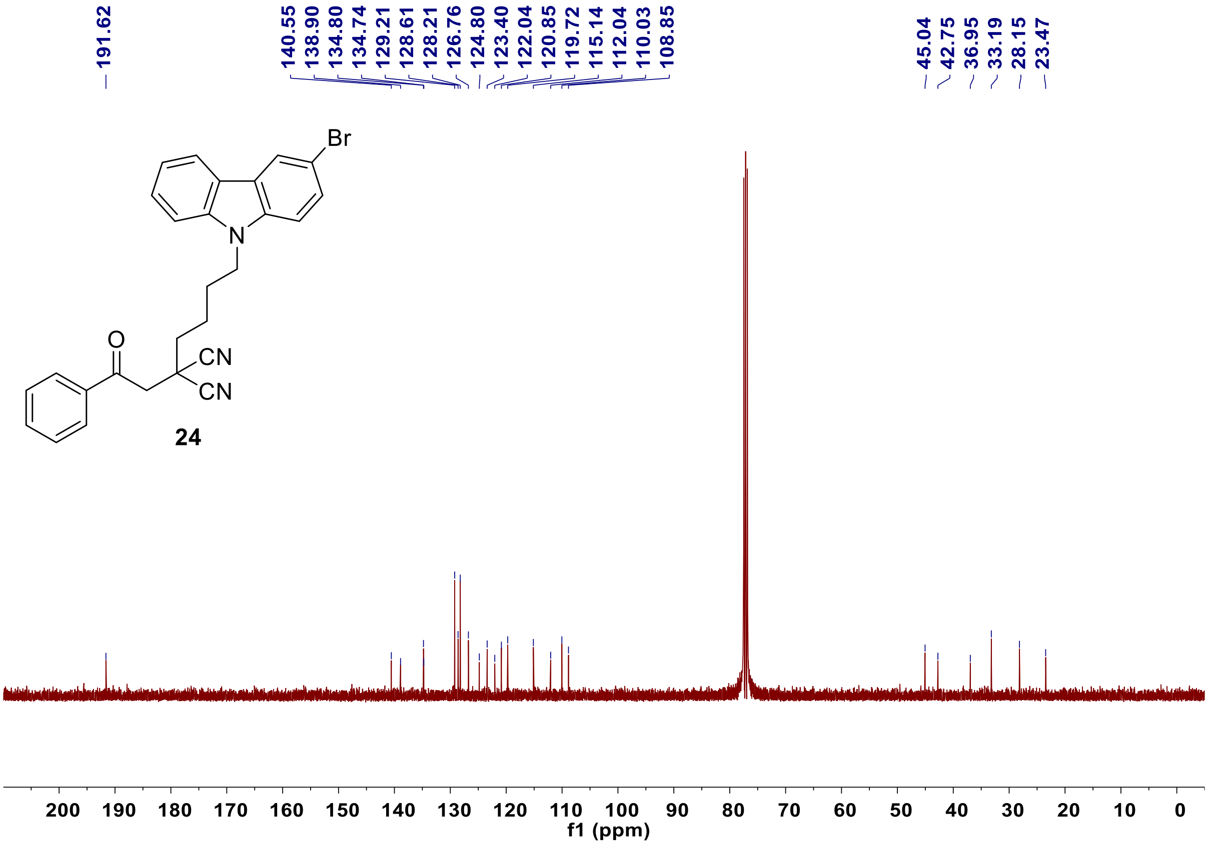

^1H and ^{13}C NMR spectra for compound 25

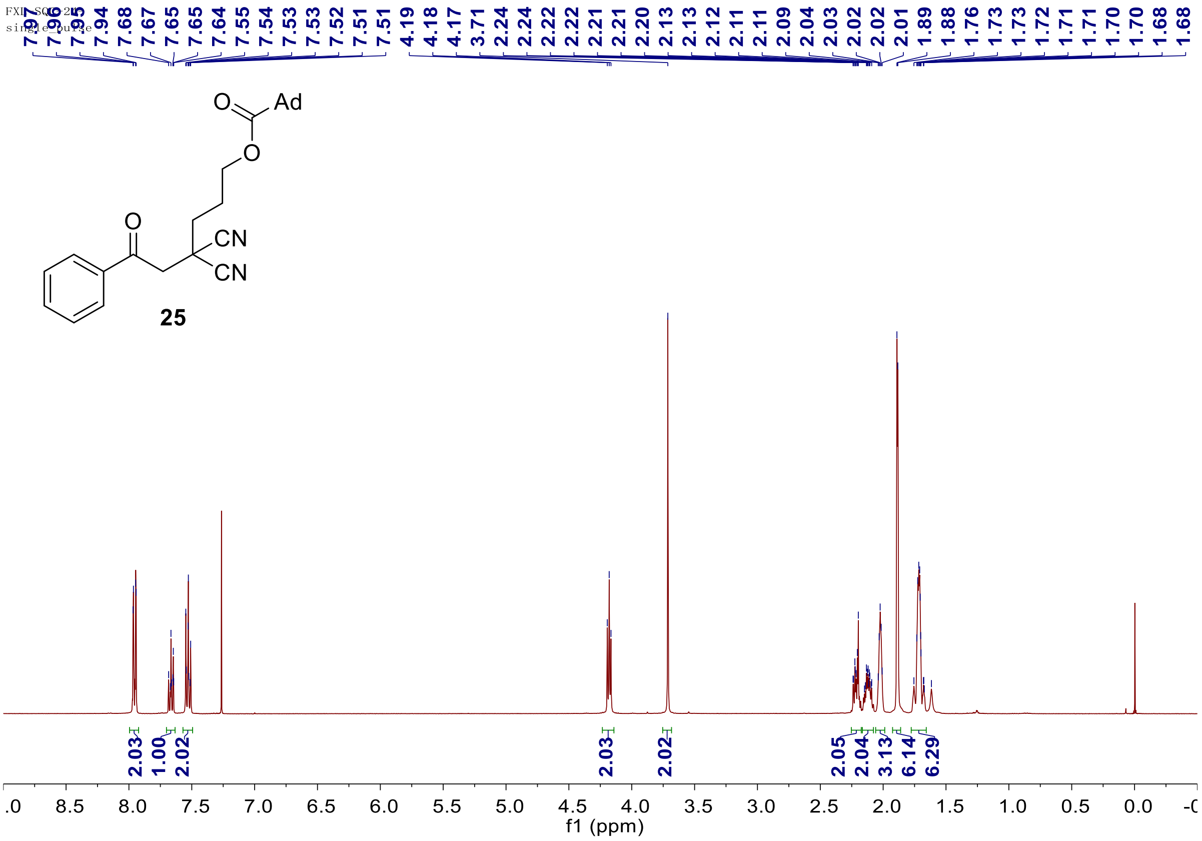

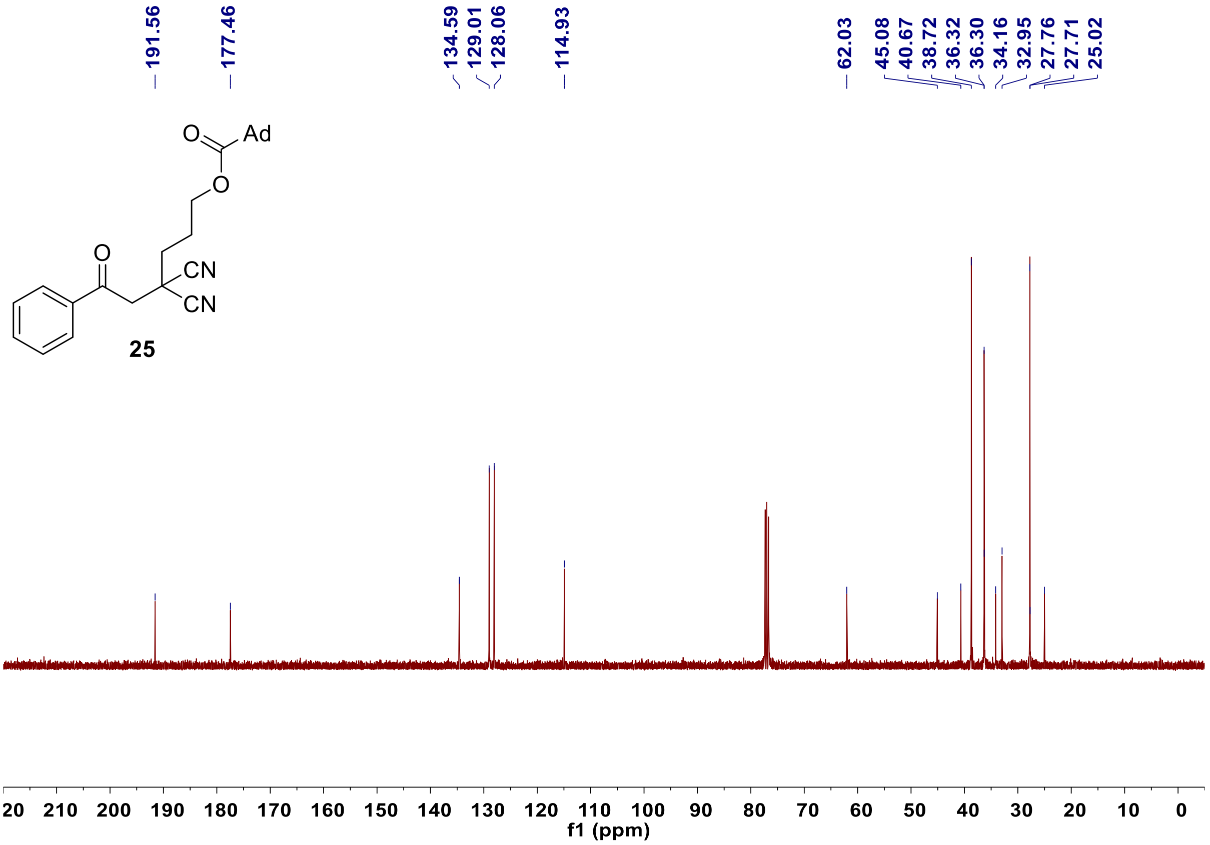

^{1}H and ^{13}C NMR spectra for compound 26

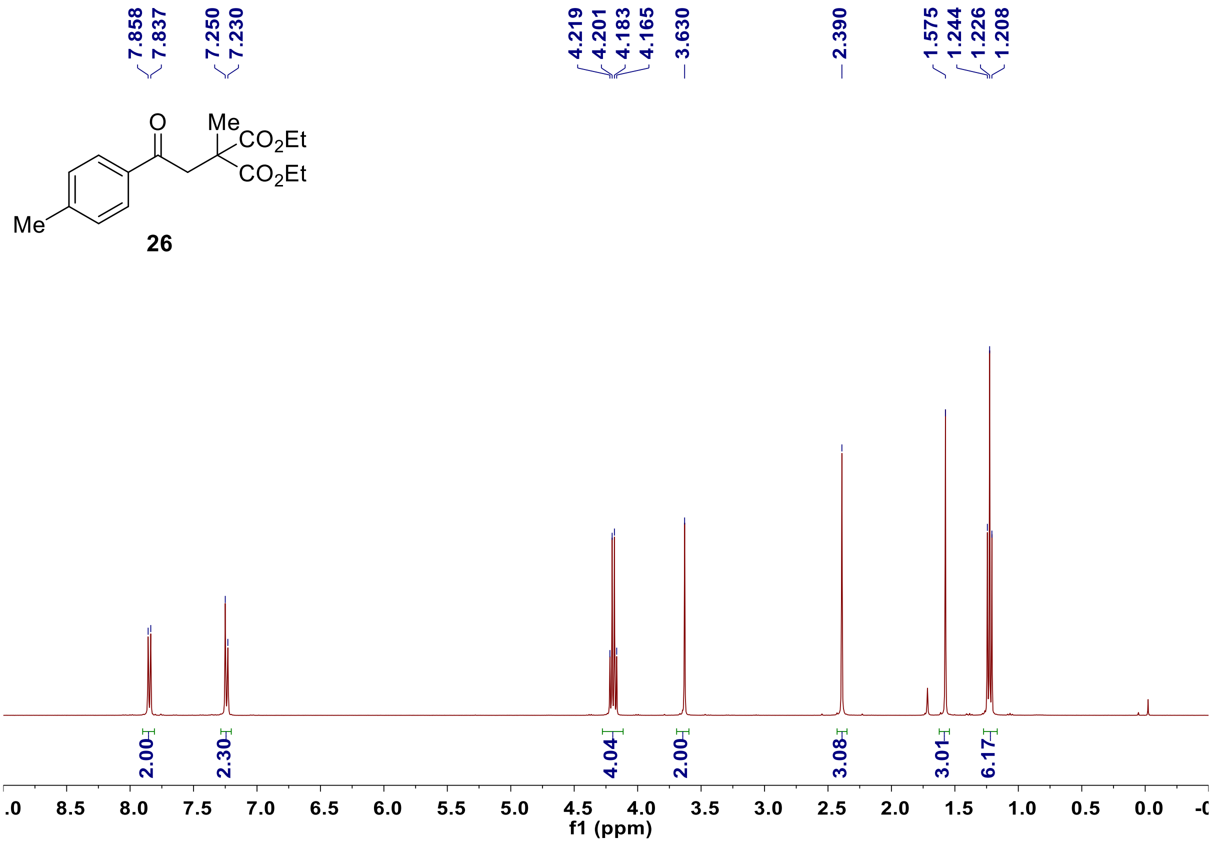

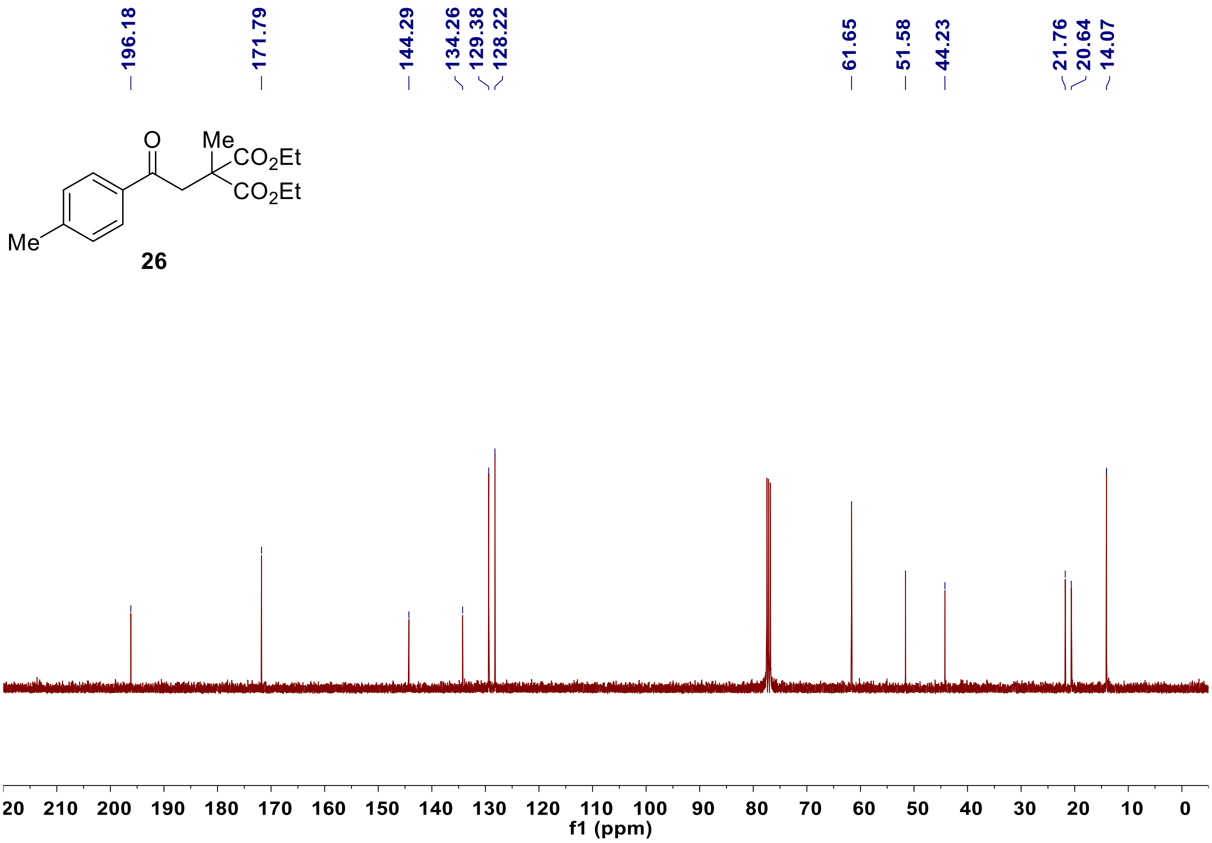

^{1}H and ^{13}C NMR spectra for compound 27

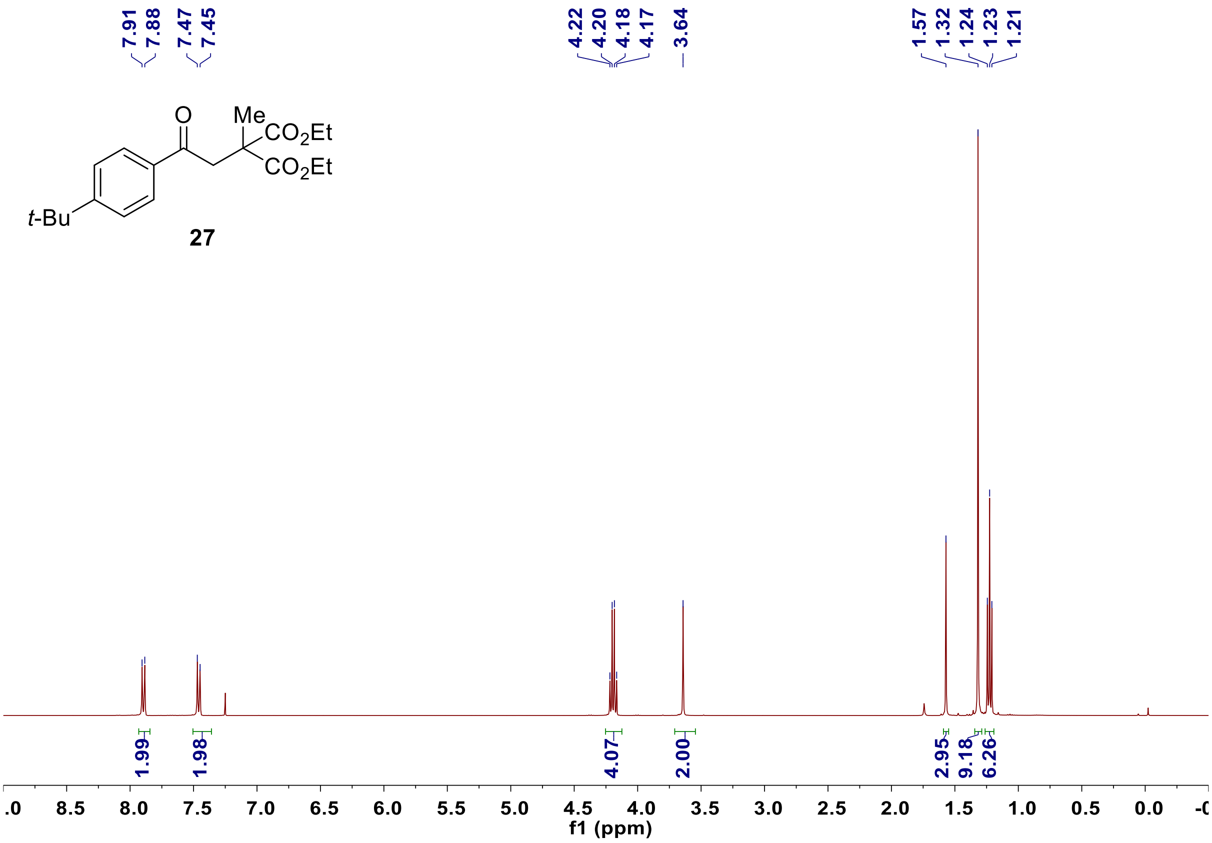

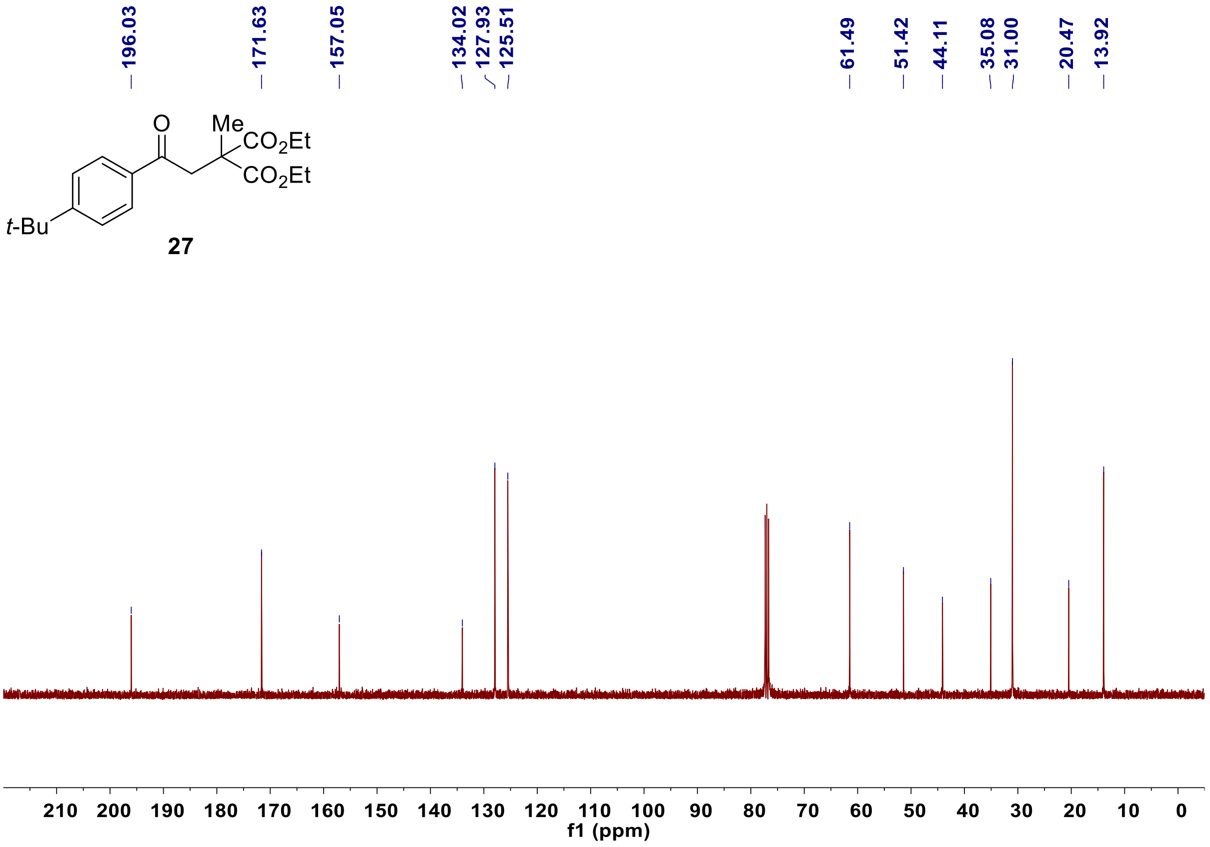

^1H and ^{13}C NMR spectra for compound 28

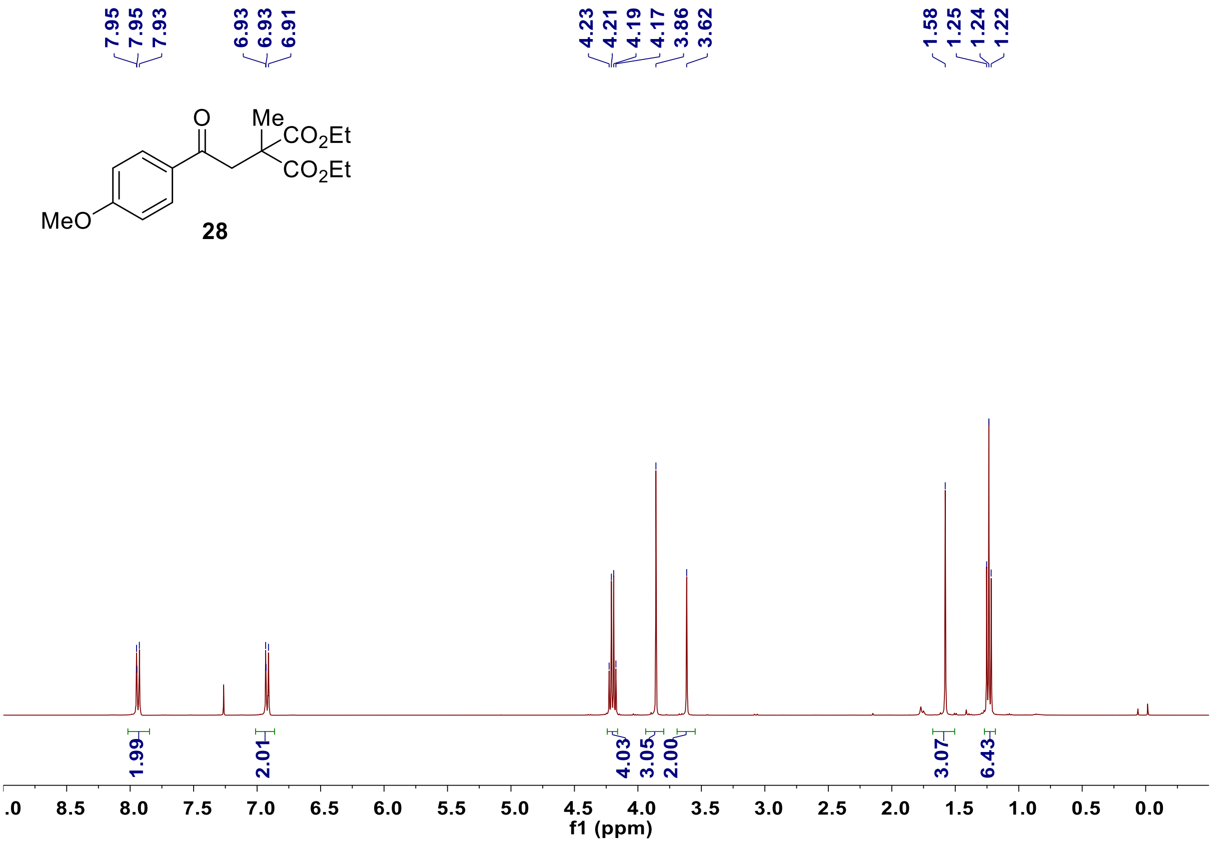

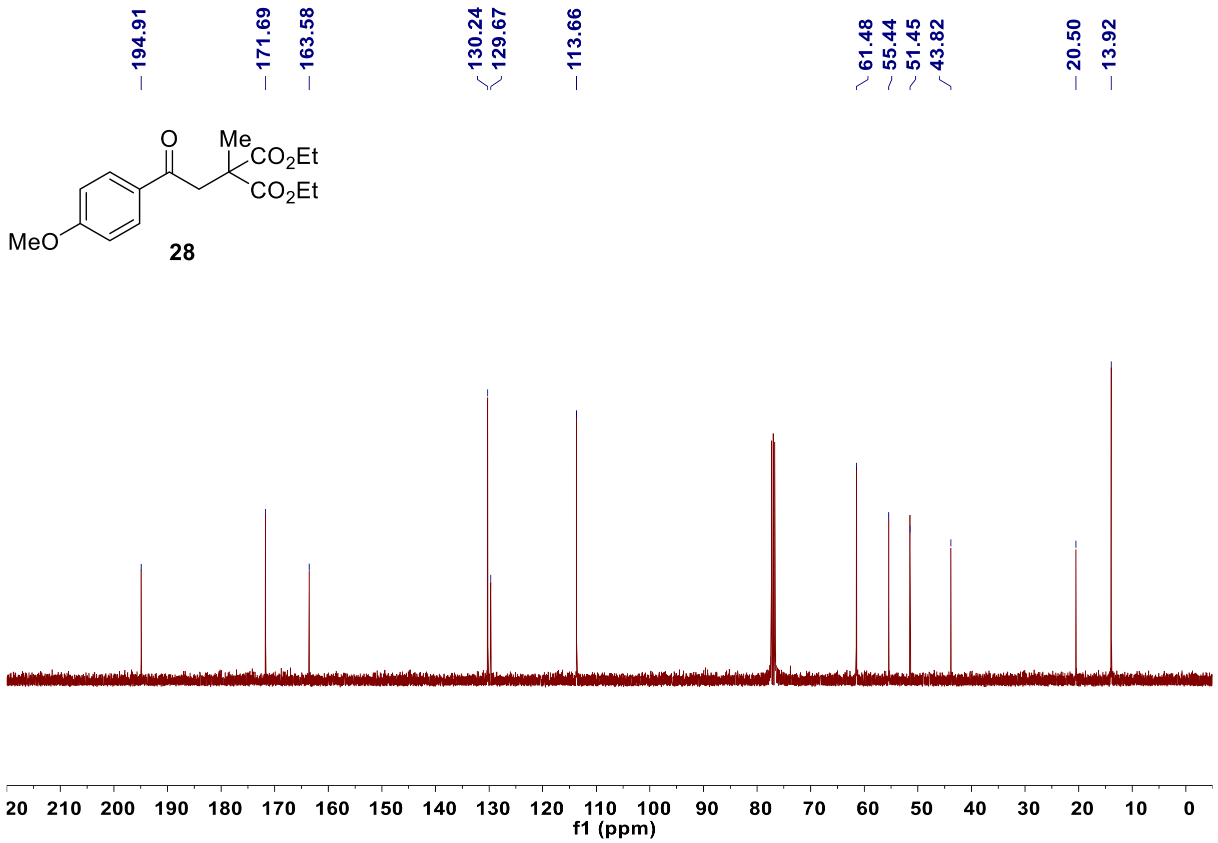

^{1}H and ^{13}C NMR spectra for compound 29

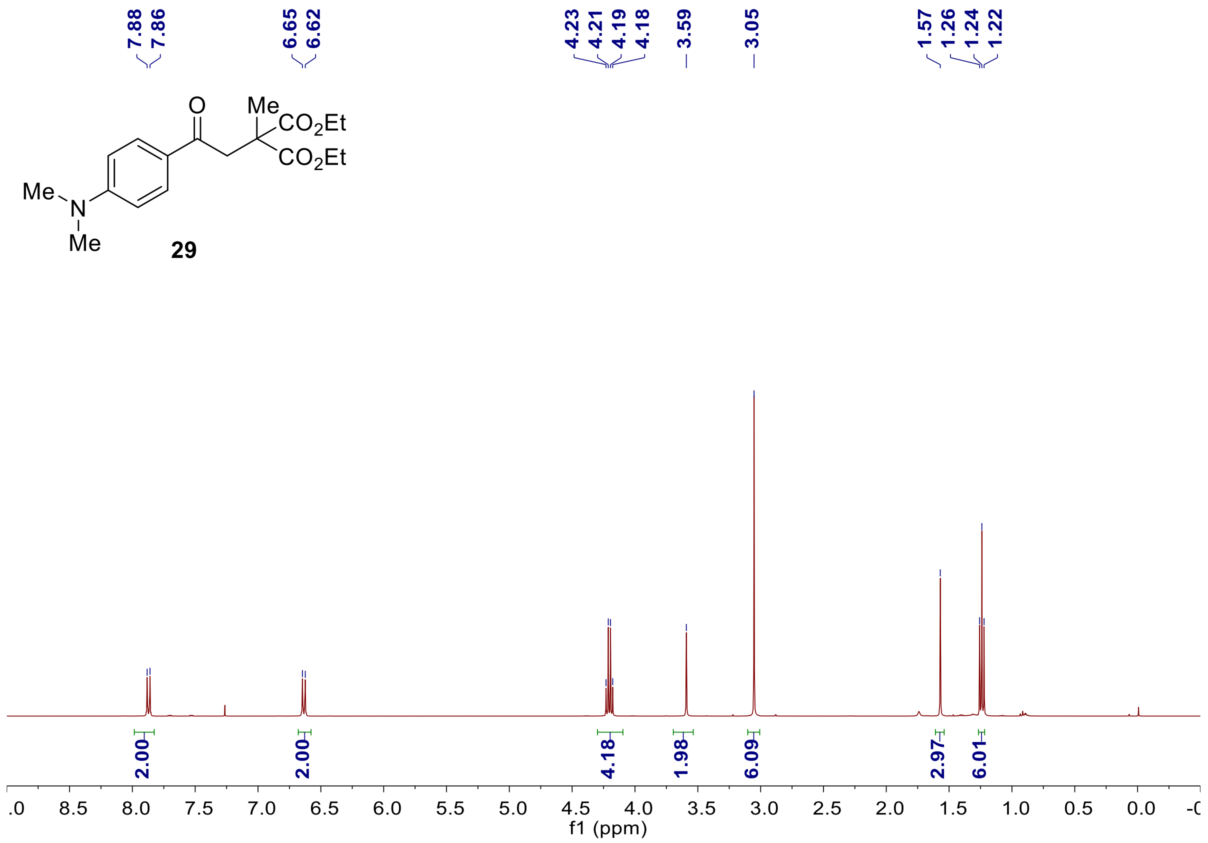

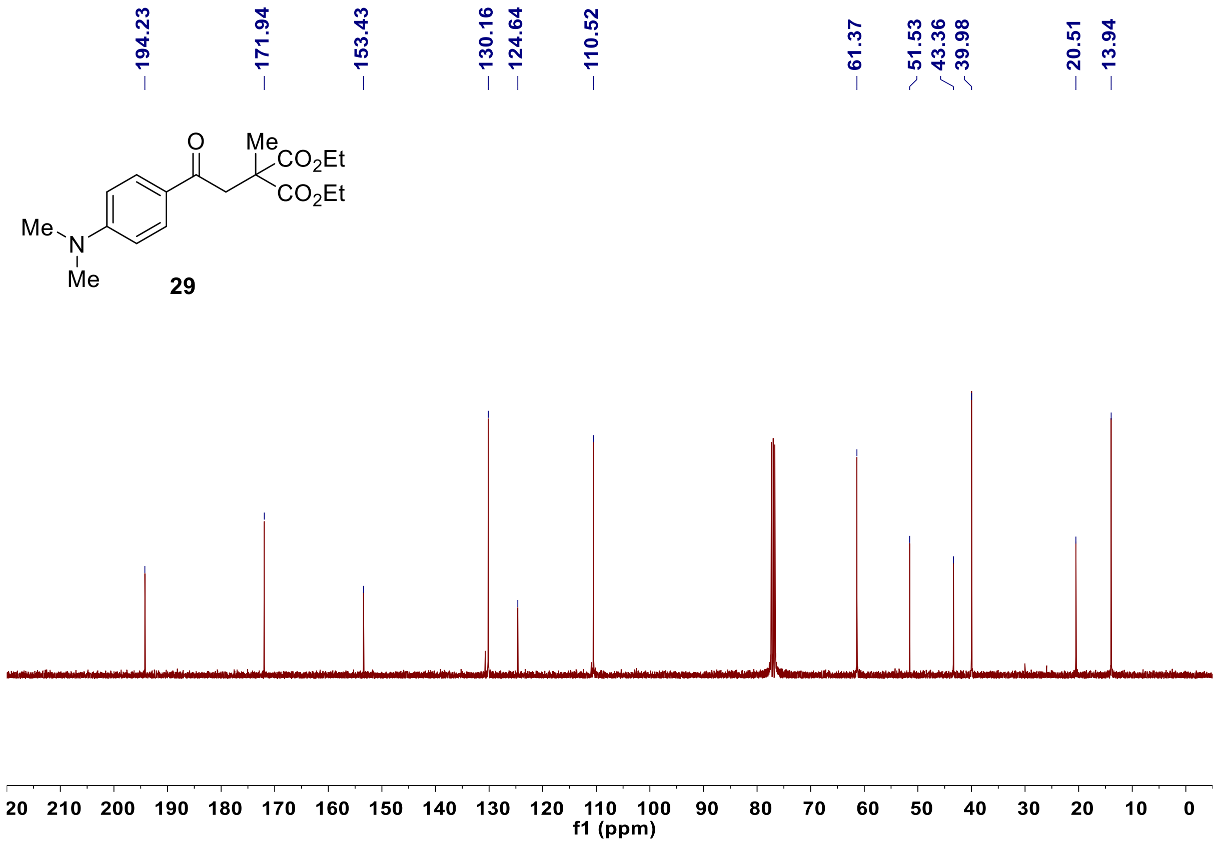

^1H and ^{13}C NMR spectra for compound 30

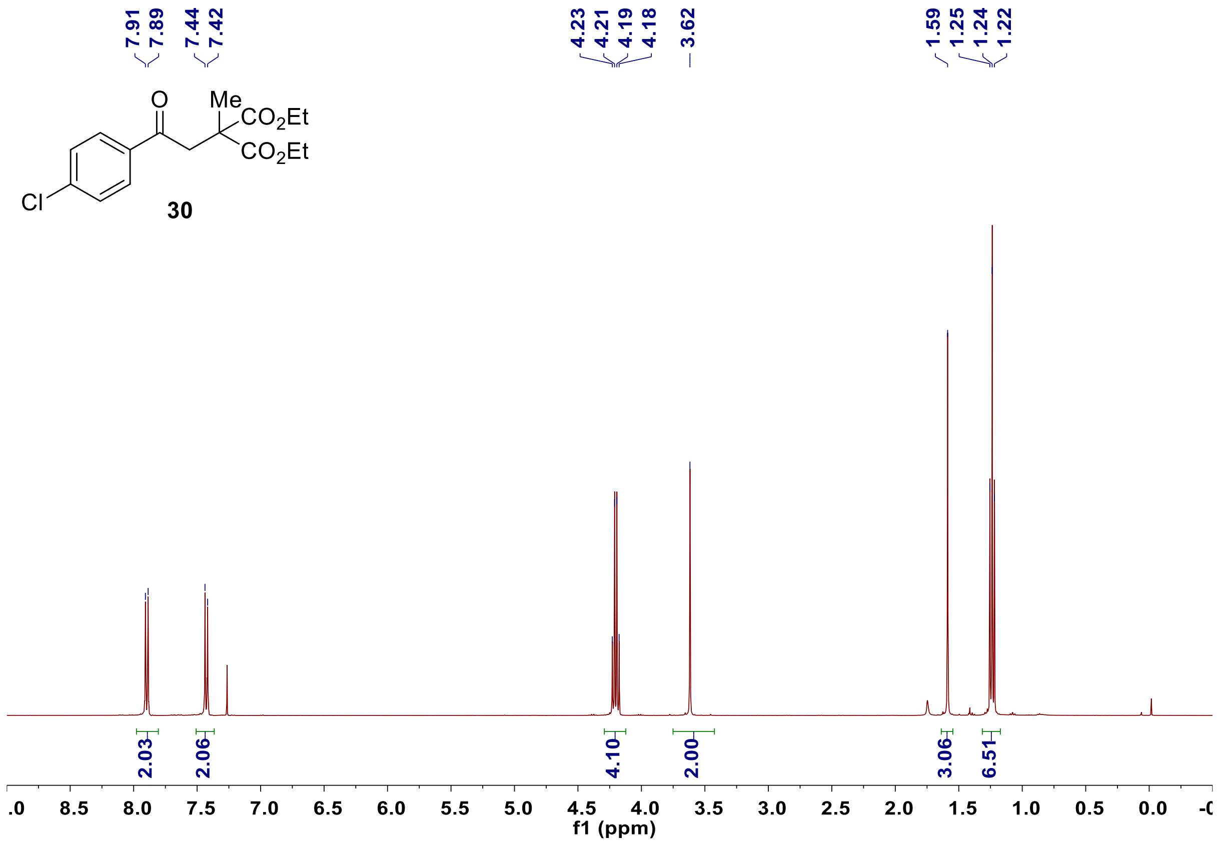

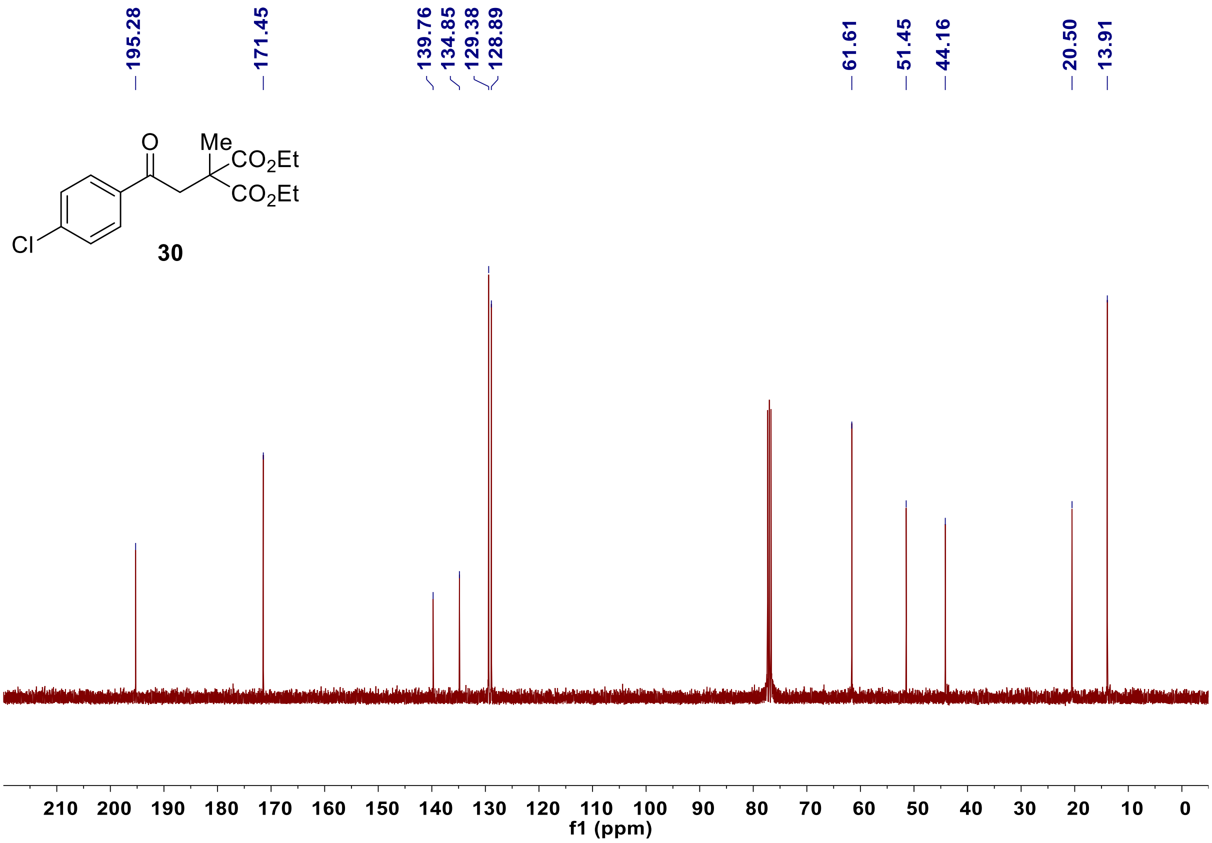

^{1}H and ^{13}C NMR spectra for compound 31

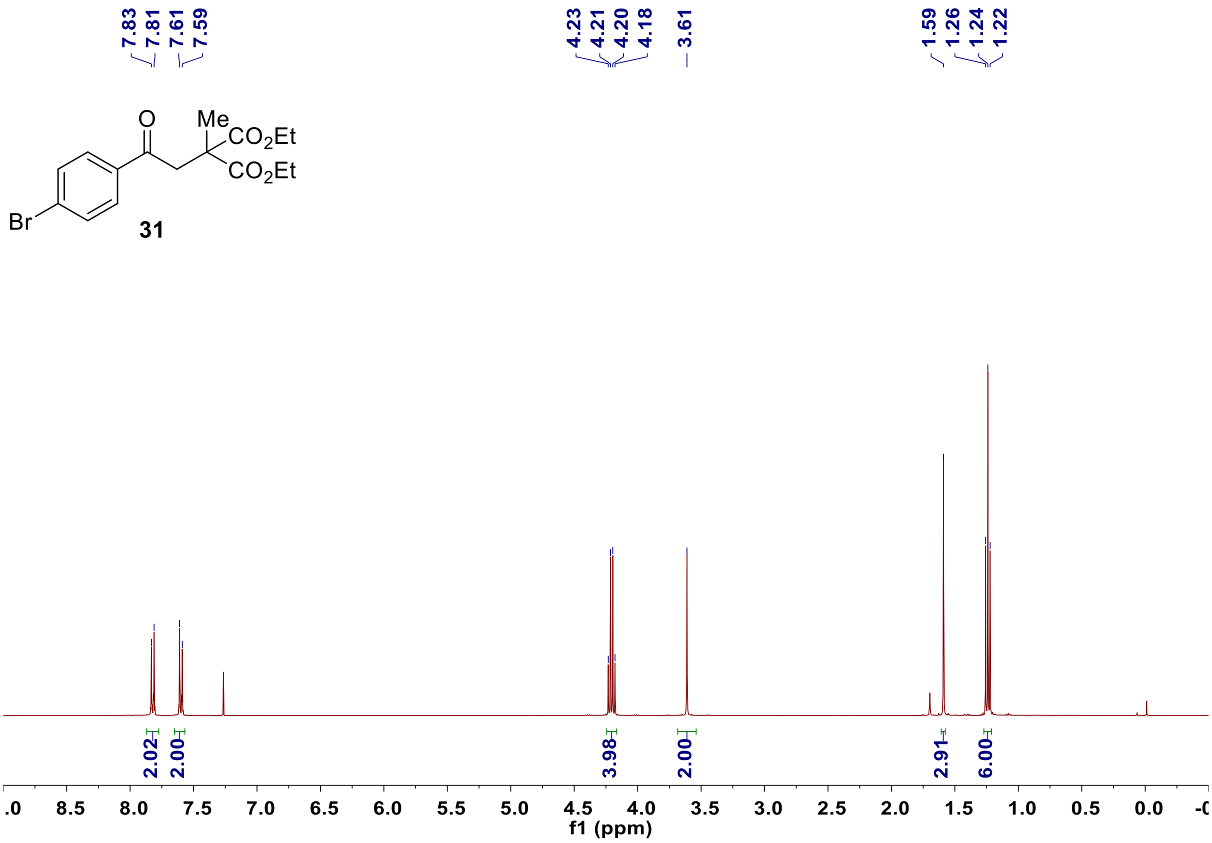

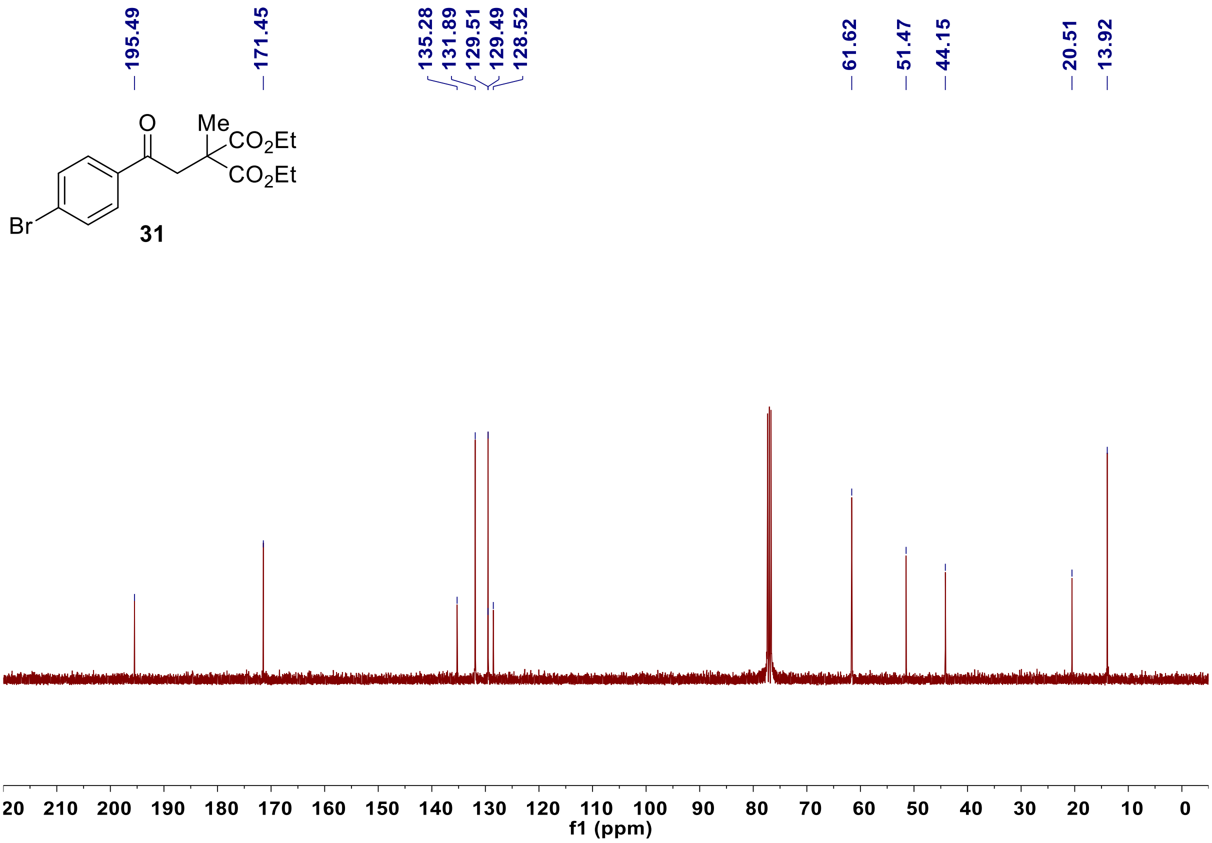

^1H and ^{13}C NMR spectra for compound 32

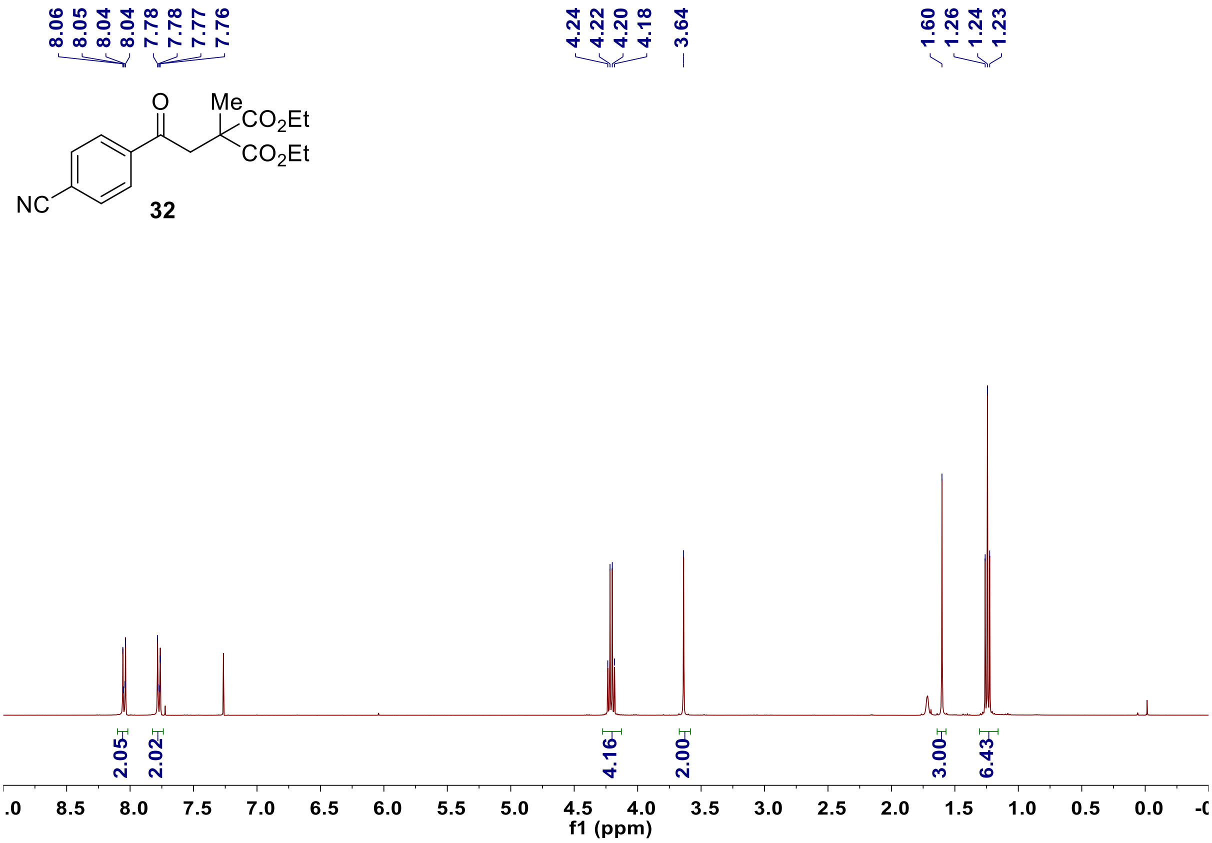

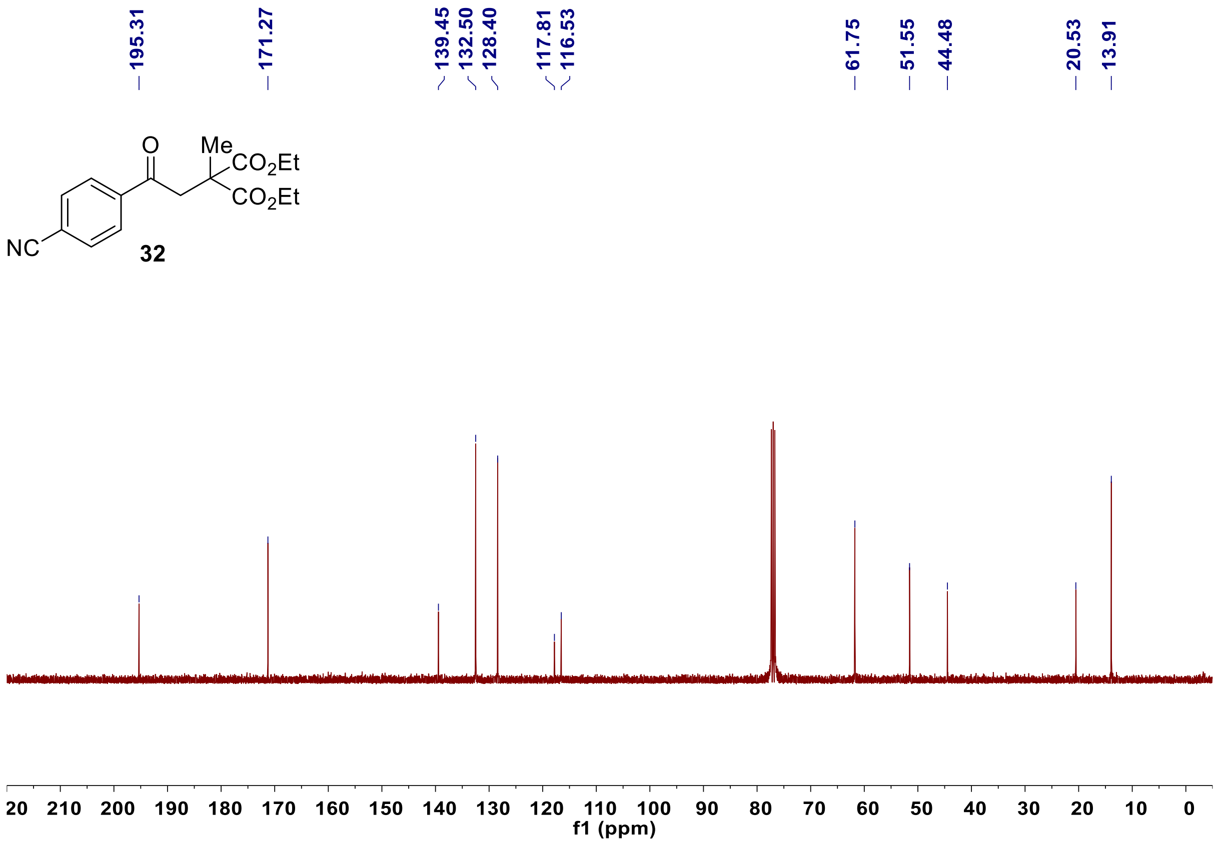

^{1}H, ^{19}F and ^{13}C NMR spectra for compound 33

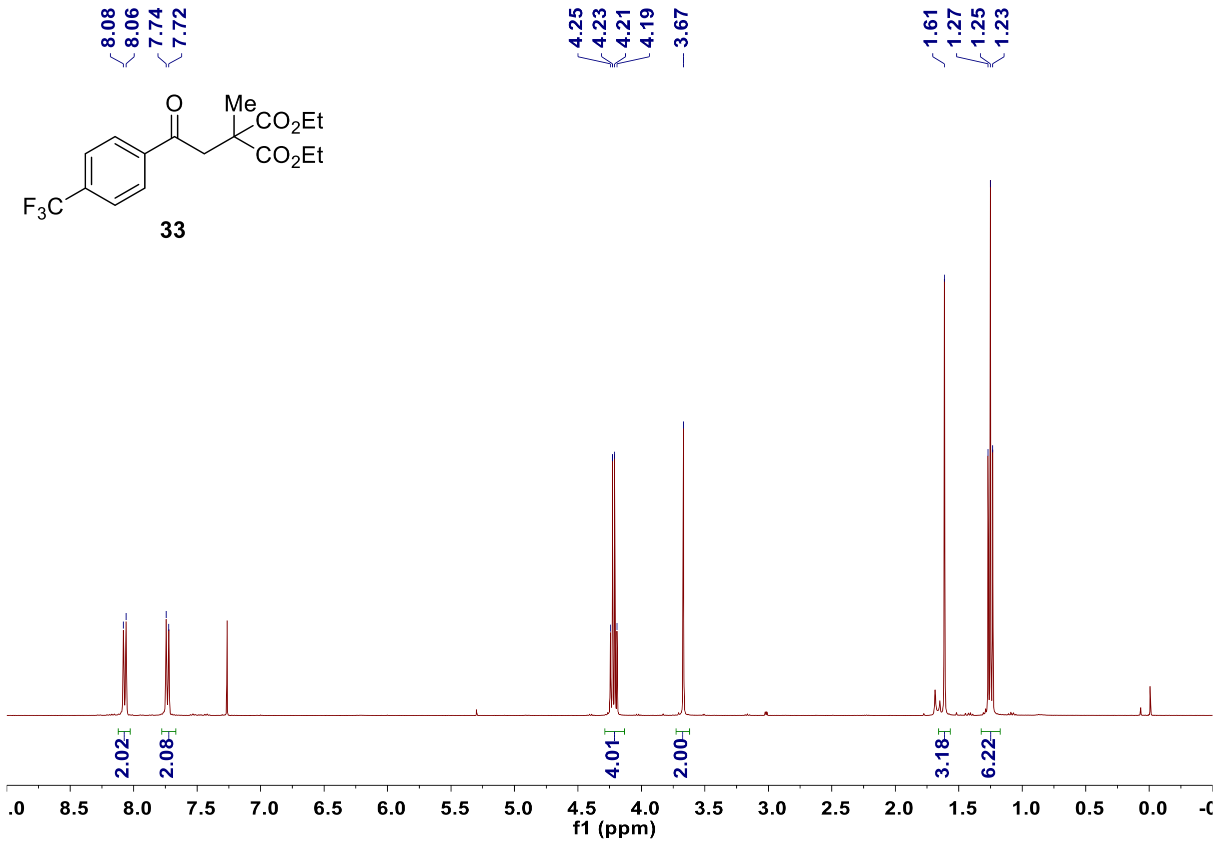

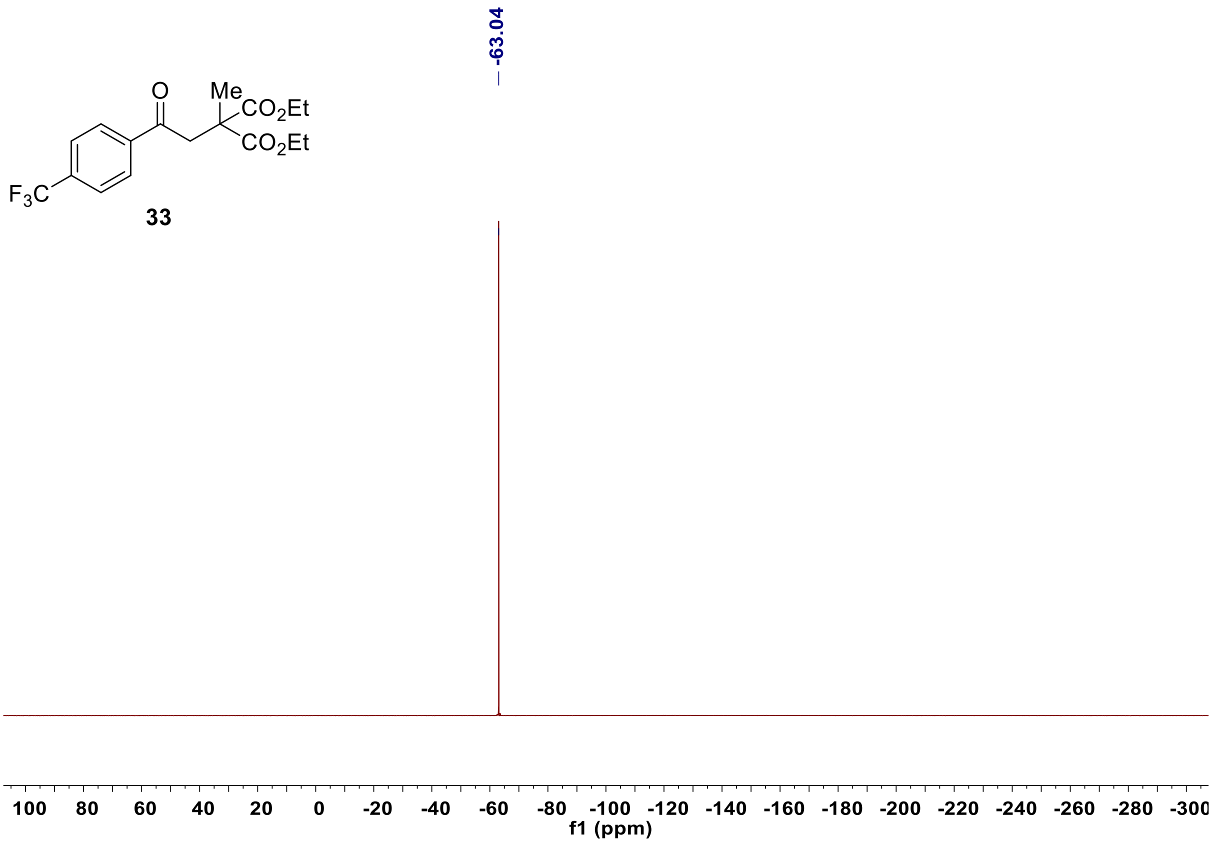
-63.04
O
Me
CO2Et
CO2Et
F3C
33
100 80 60 40 20 0 -20 -40 -60 -80 -100 -120 -140 -160 -180 -200 -220 -240 -260 -280 -300
f1 (ppm)

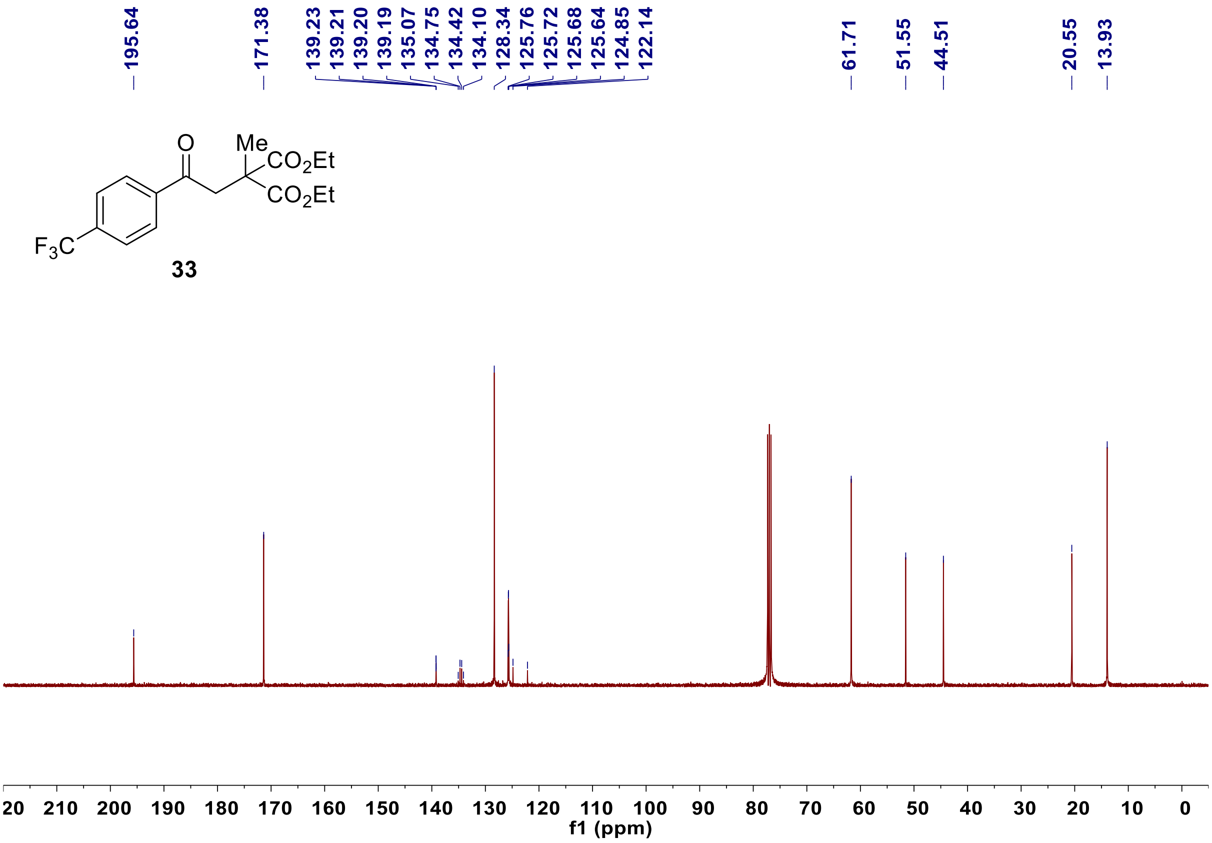
195.64
171.38
139.23
139.21
139.20
139.19
135.07
134.75
134.42
134.10
128.34
125.76
125.72
125.68
125.64
124.85
122.14
61.71
51.55
44.51
20.55
13.93
O
Me
CO2Et
CO2Et
F3C
33
20 210 200 190 180 170 160 150 140 130 120 110 100 90 80 70 60 50 40 30 20 10 0
f1 (ppm)

^{1}H and ^{13}C NMR spectra for compound 34

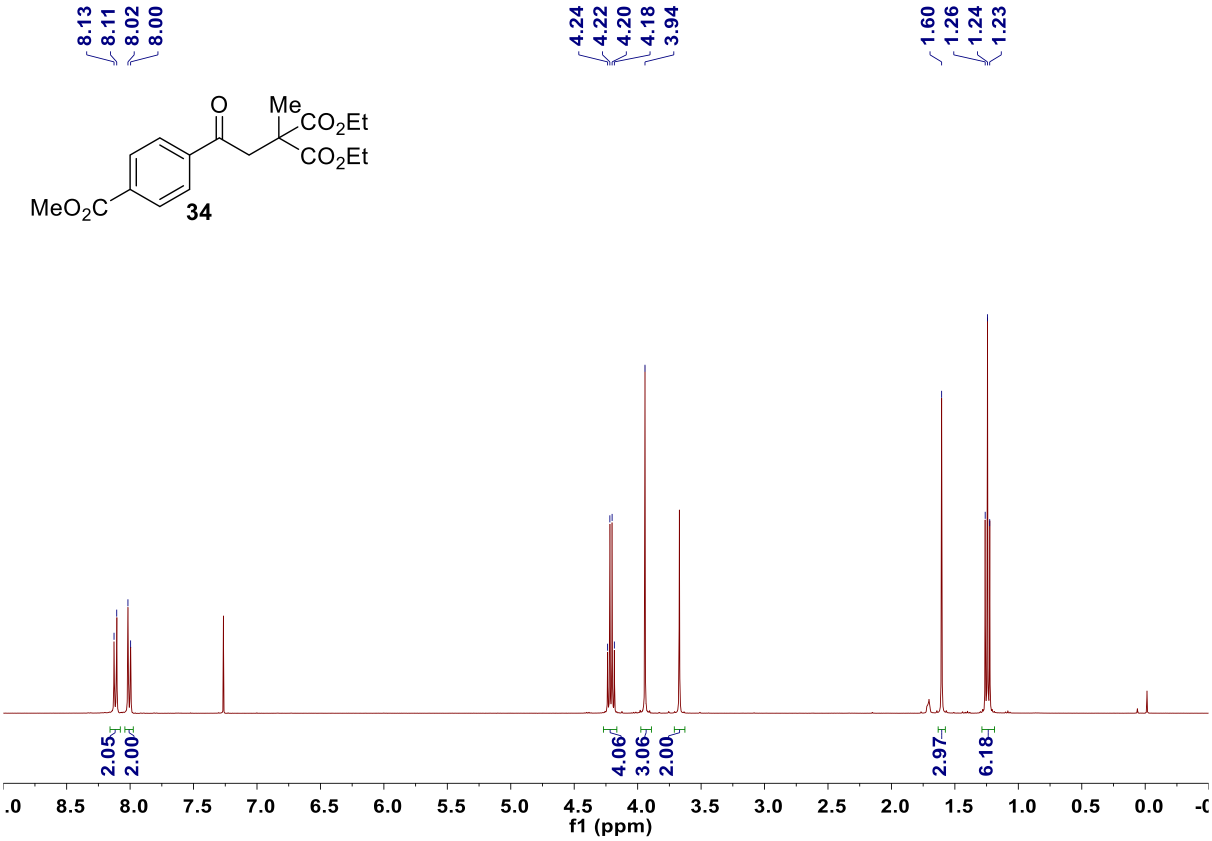

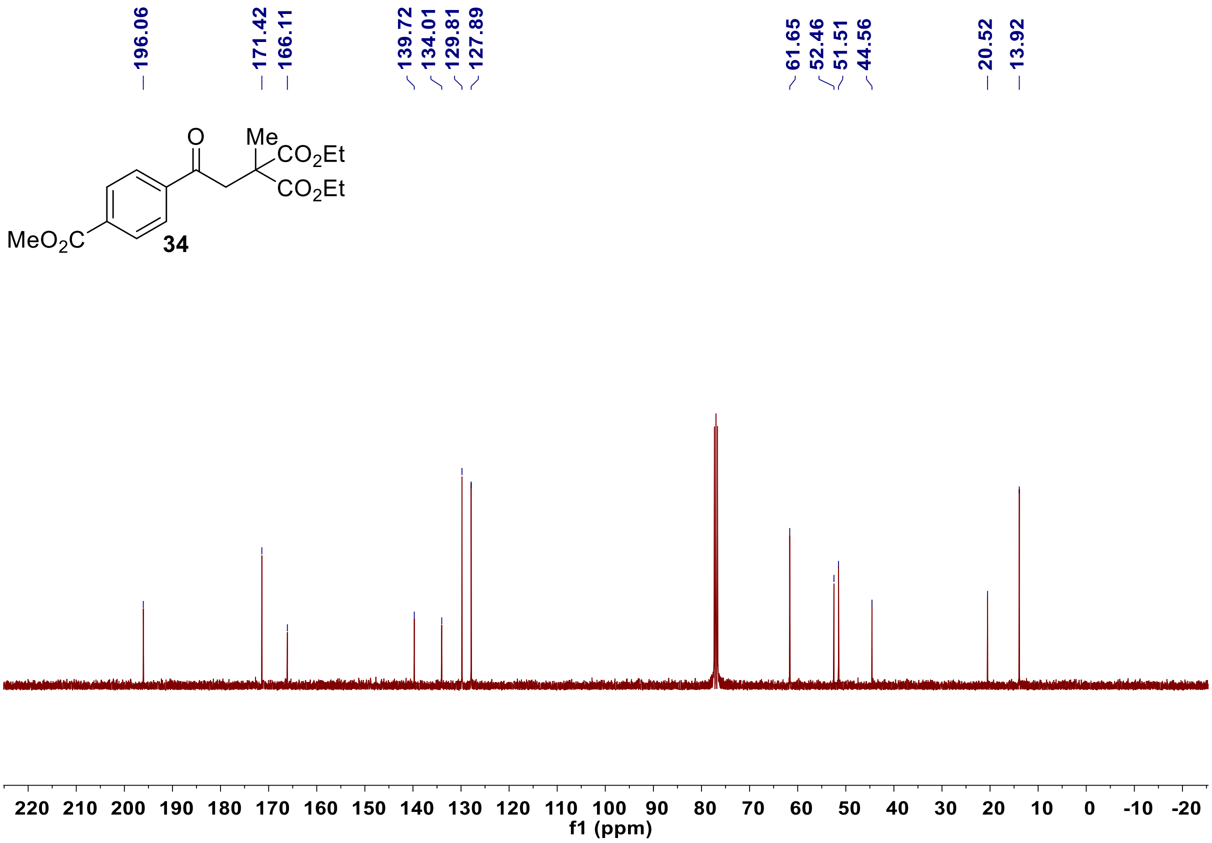

^{1}H and ^{13}C NMR spectra for compound 35

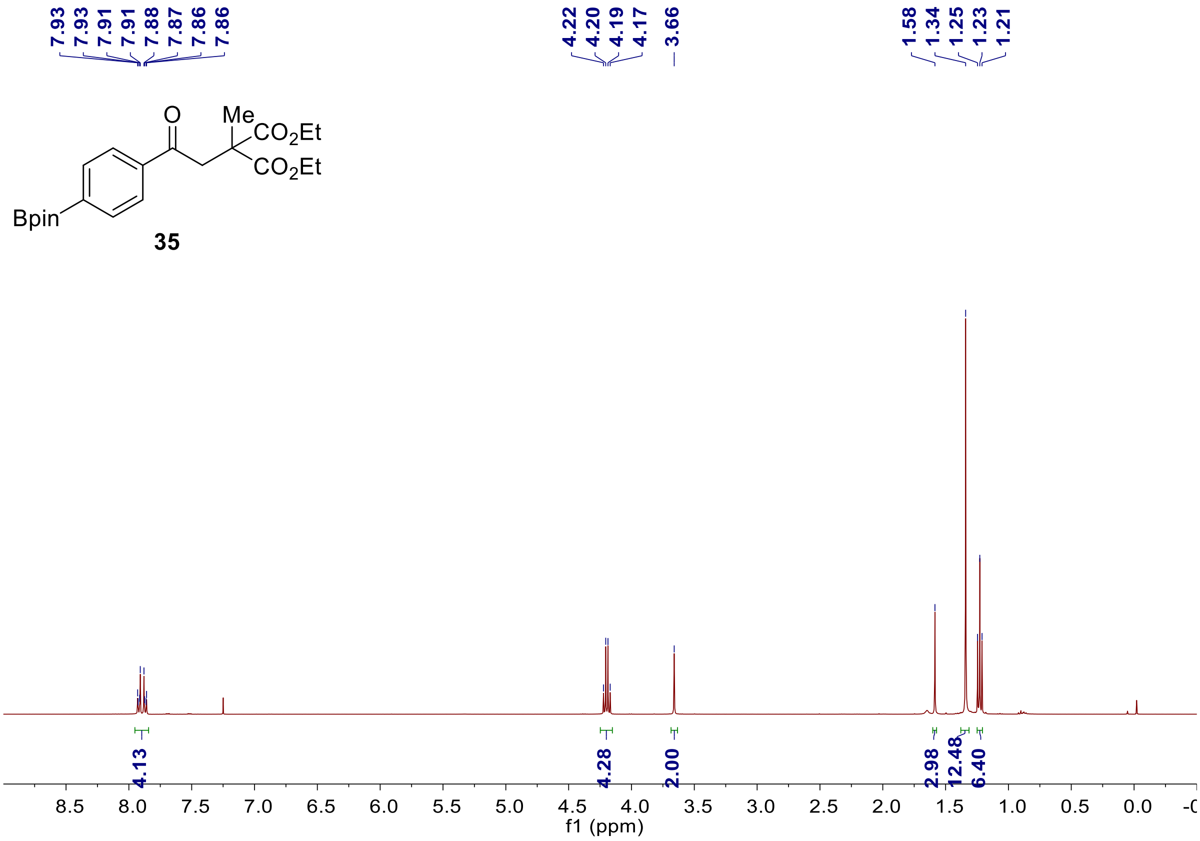

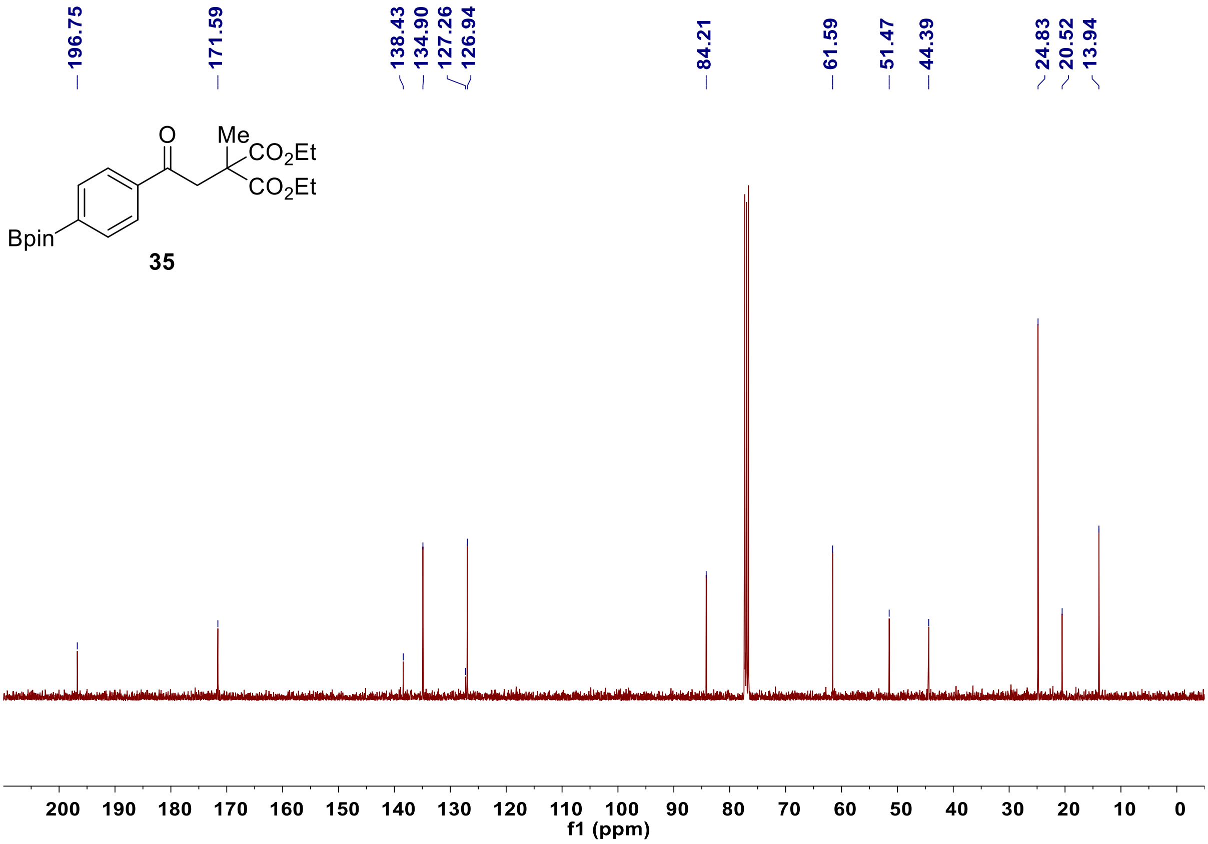

^{1}H and ^{13}C NMR spectra for compound 36

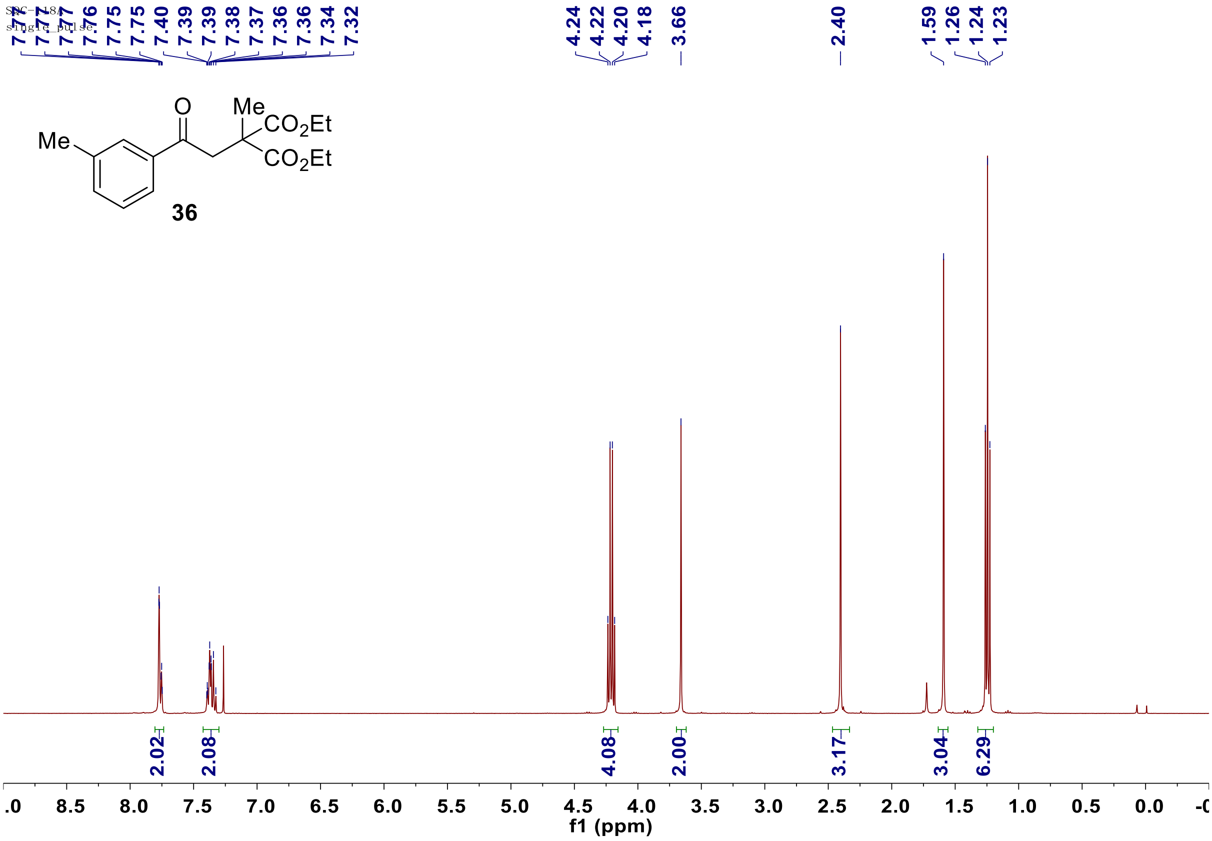

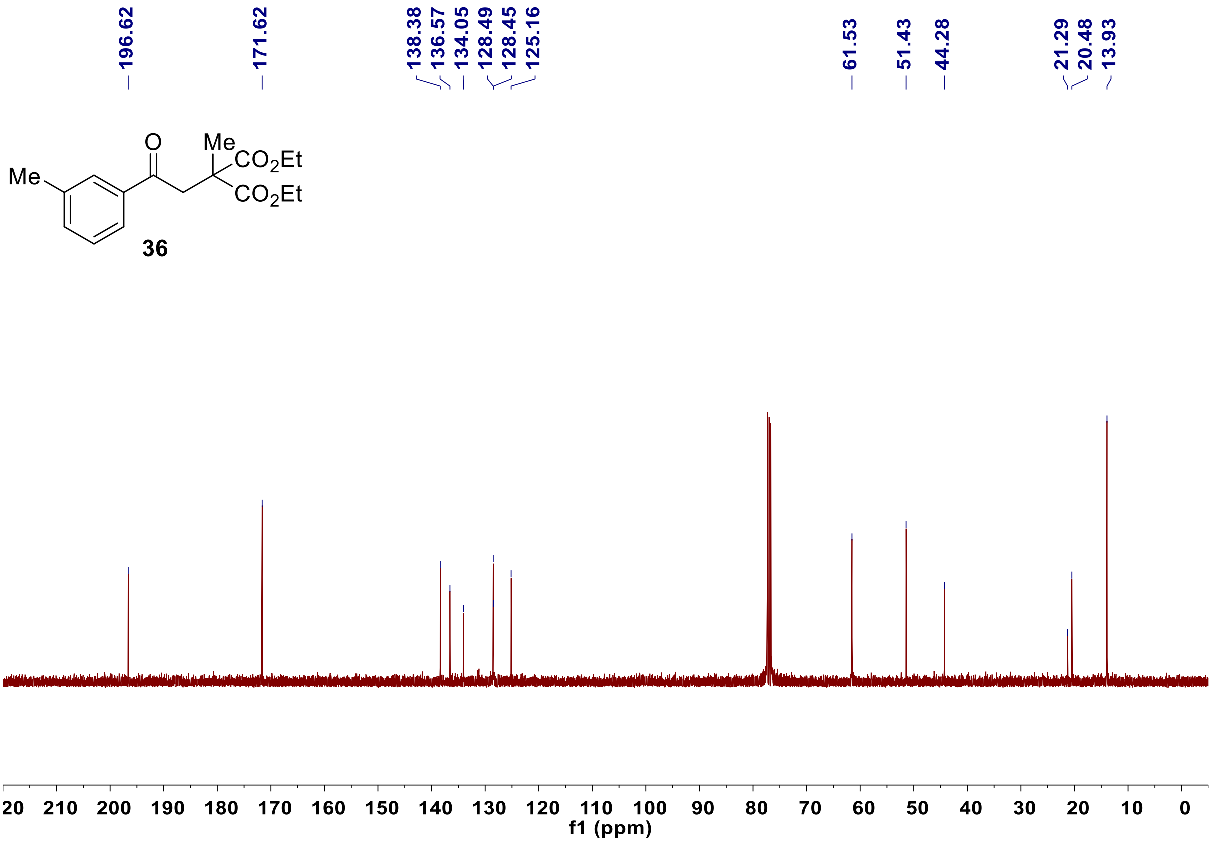

^{1}H and ^{13}C NMR spectra for compound 37

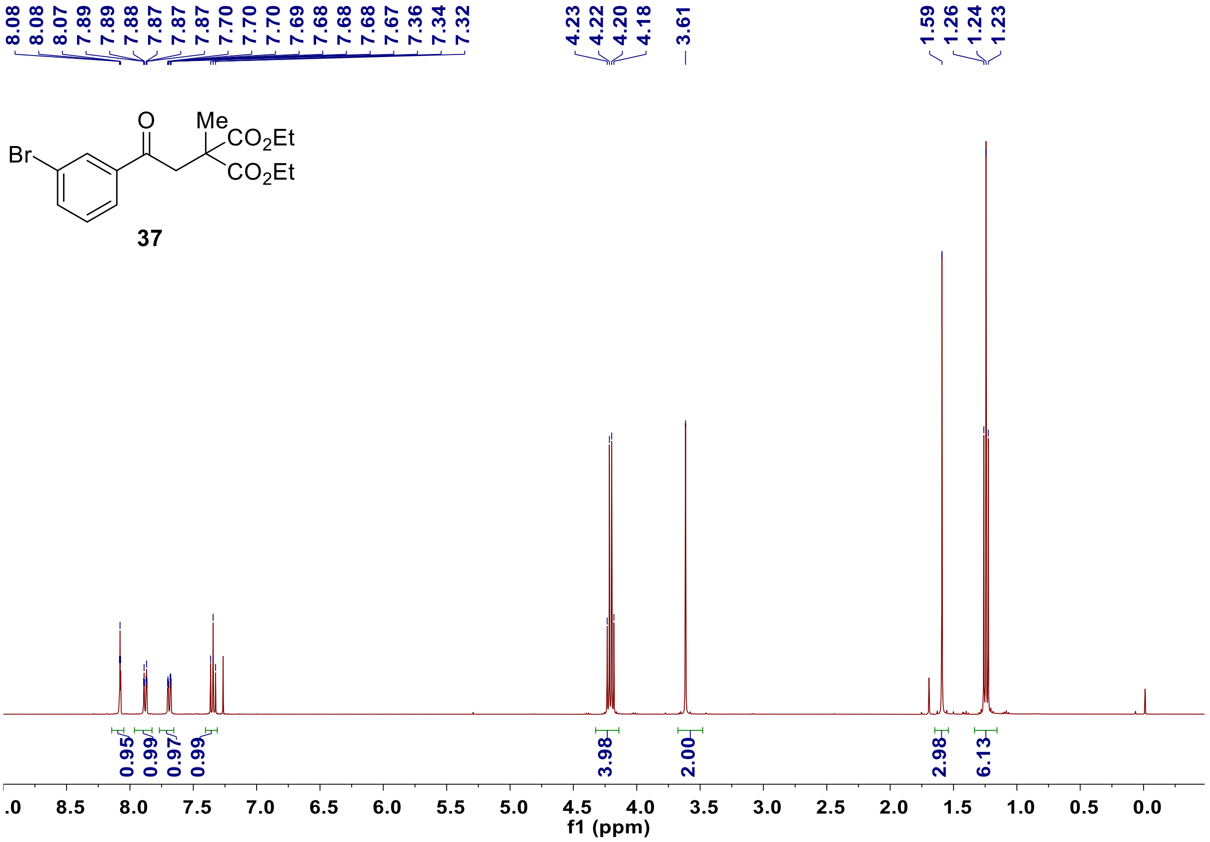

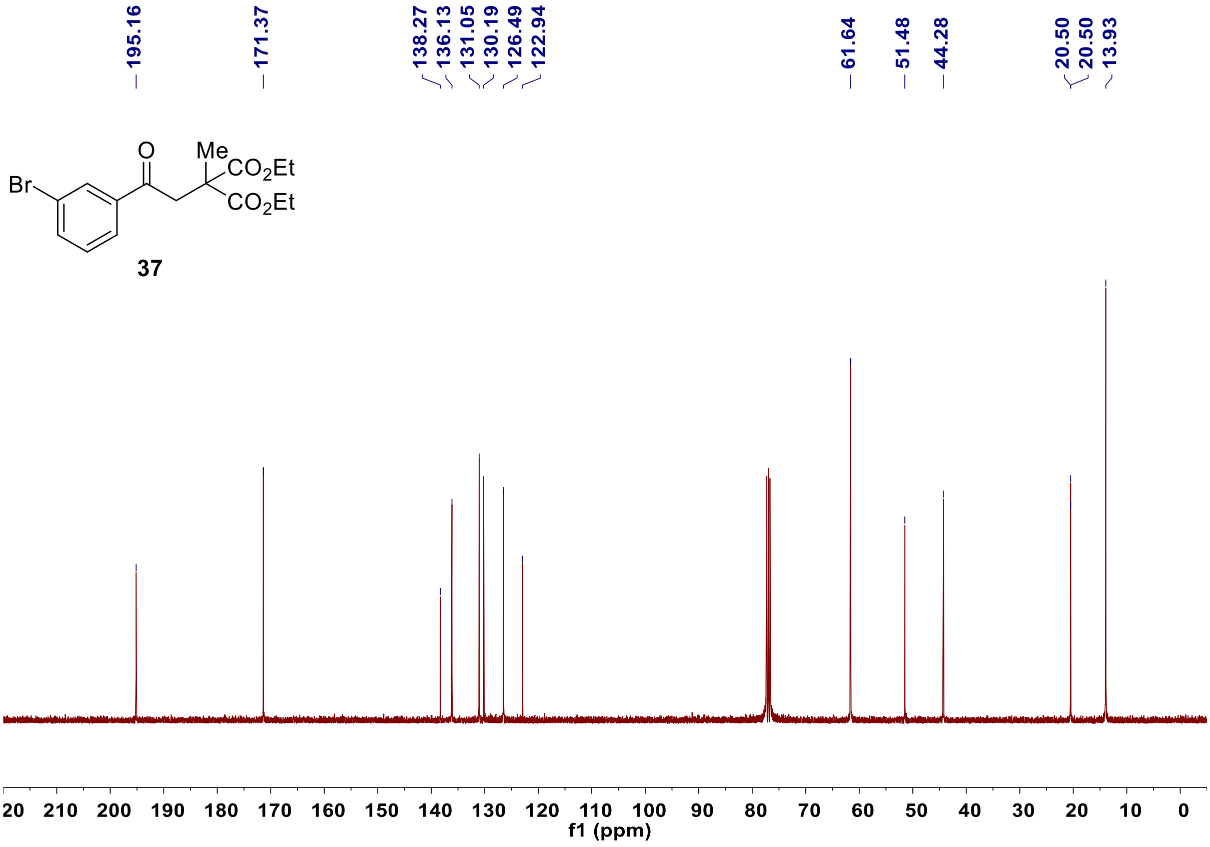

^{1}H and ^{13}C NMR spectra for compound 38

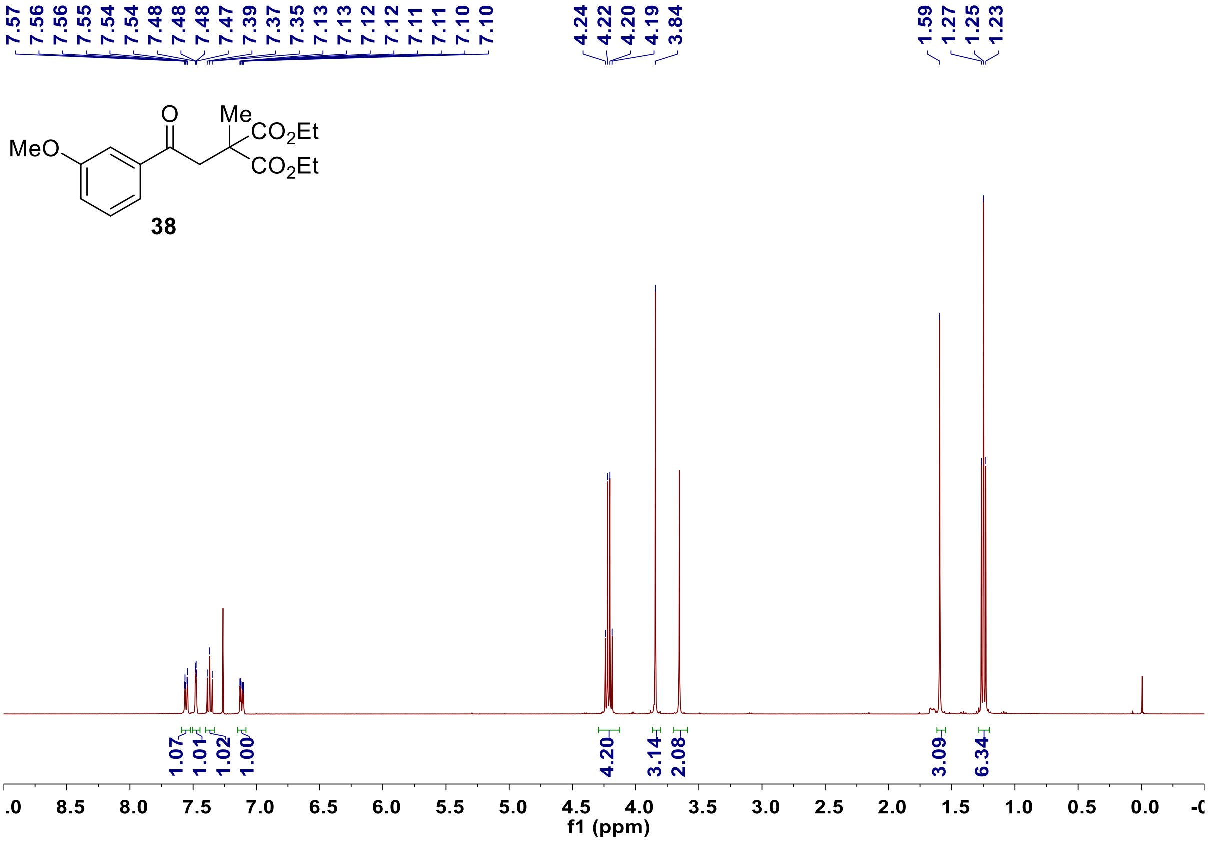

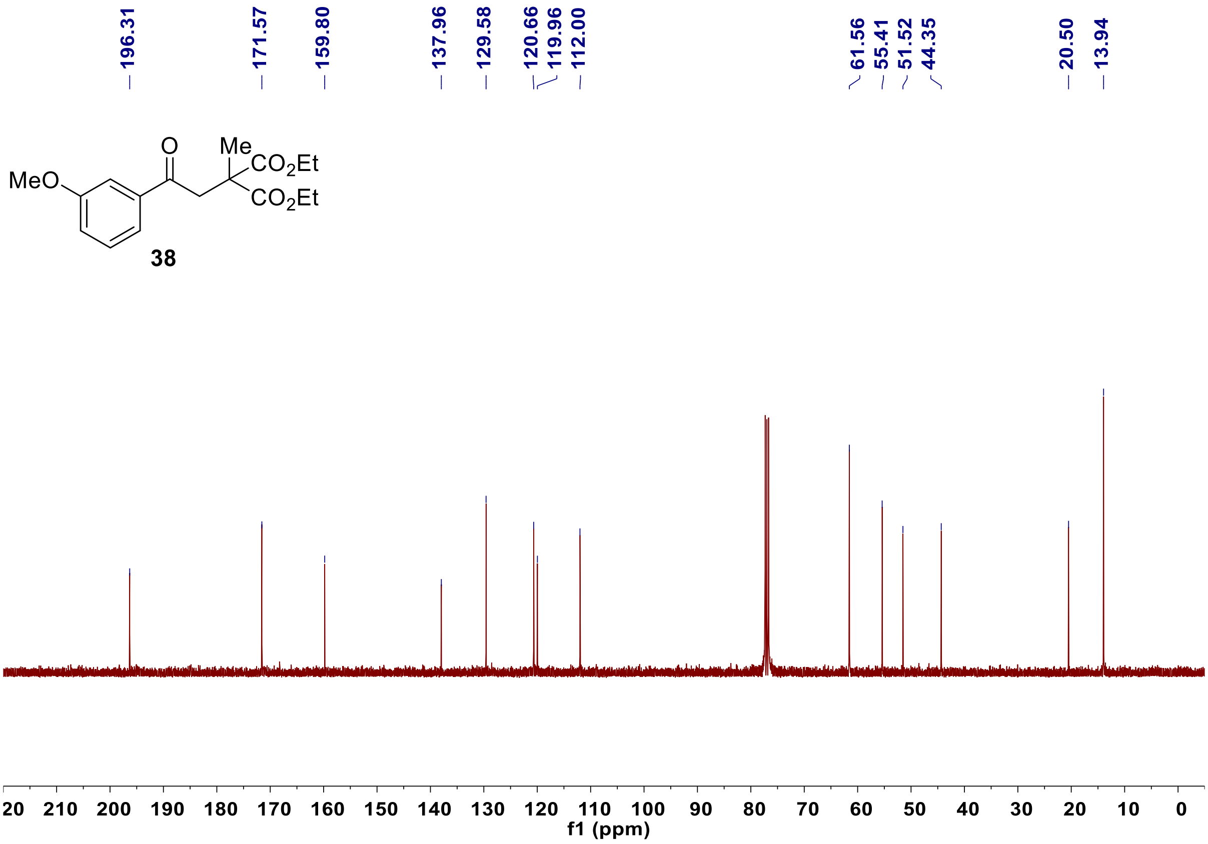

.^{1}H, and ^{13}C NMR spectra for compound 39

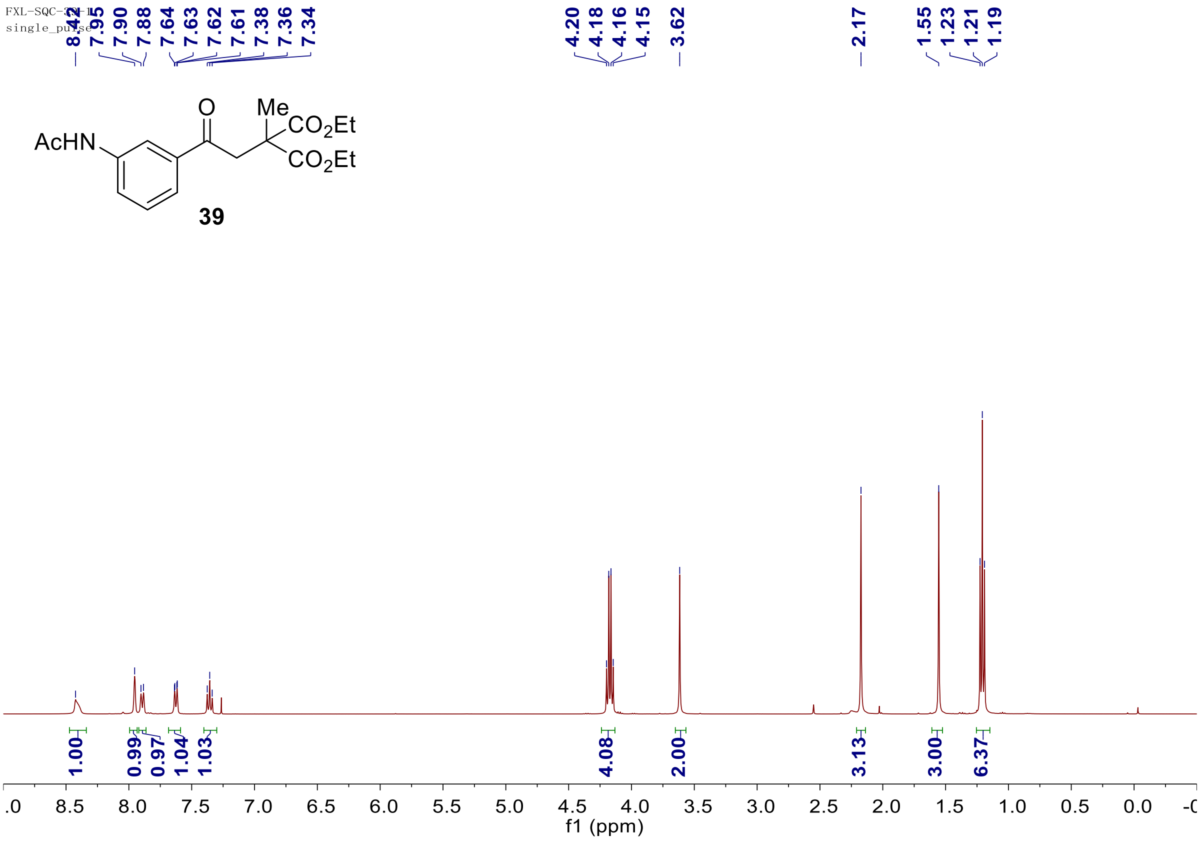

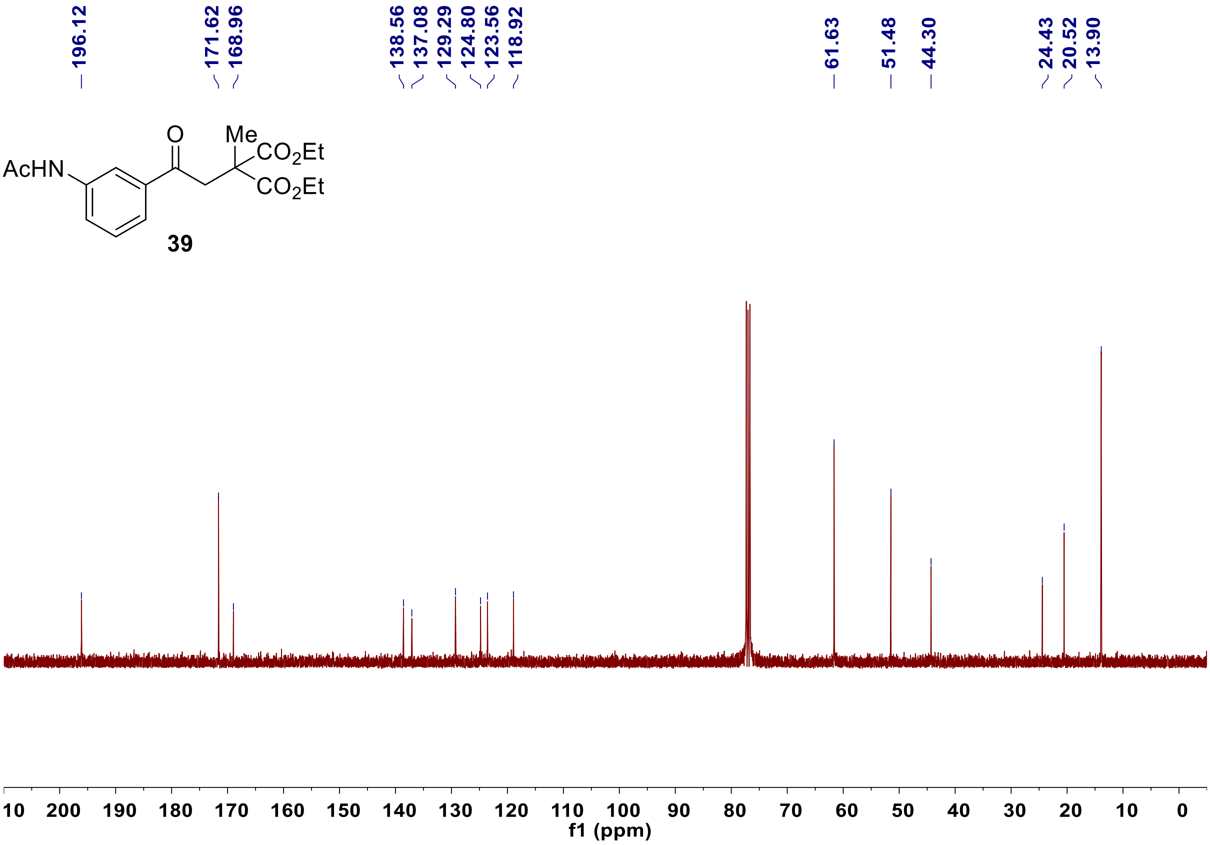

^{1}H, ^{19}F and ^{13}C NMR spectra for compound 40

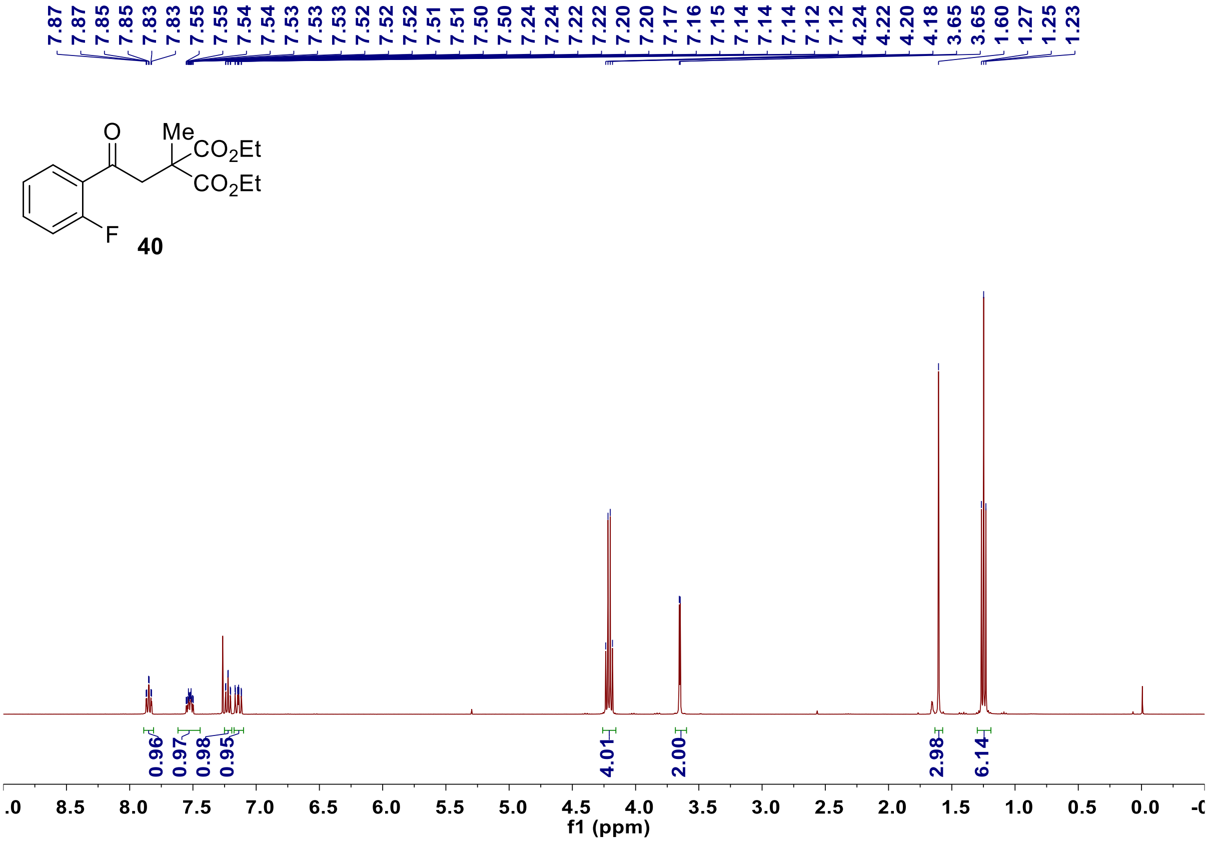

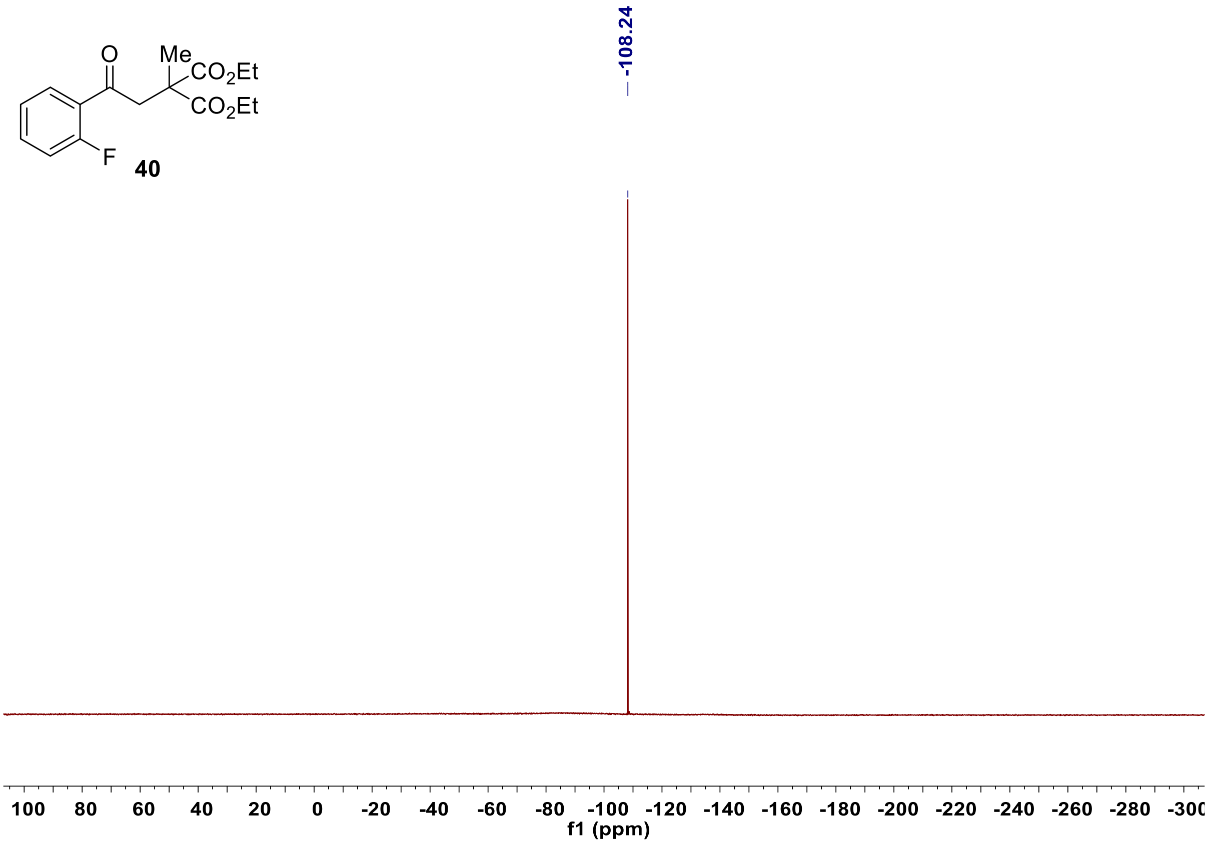

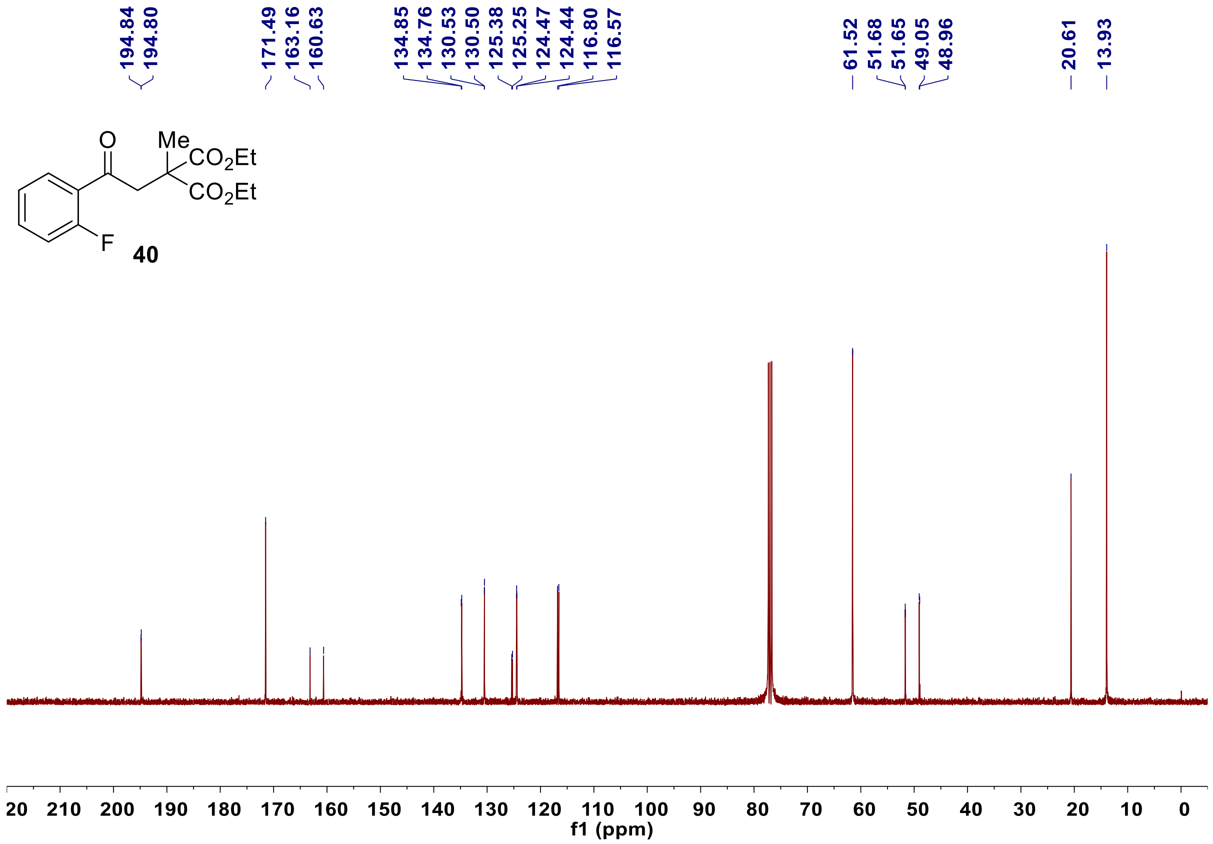

^1H and ^{13}C NMR spectra for compound 41

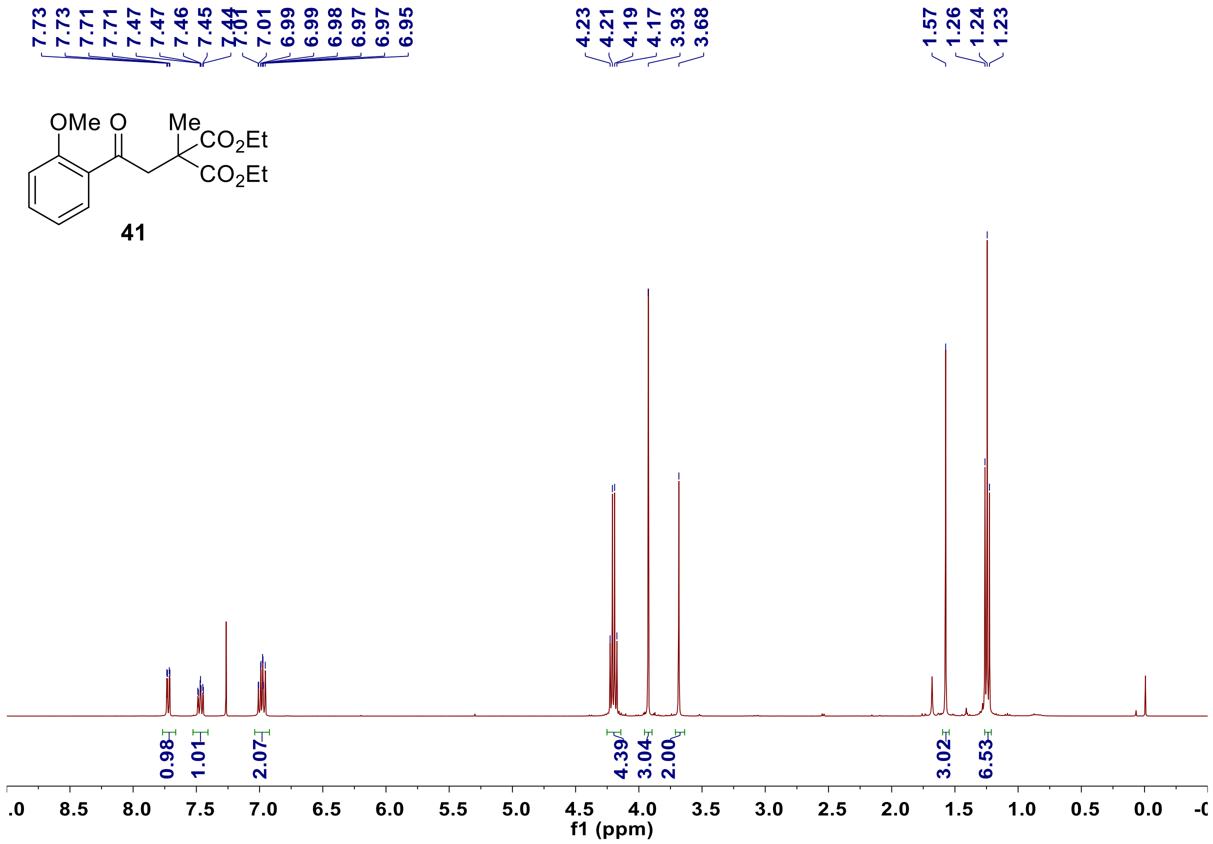

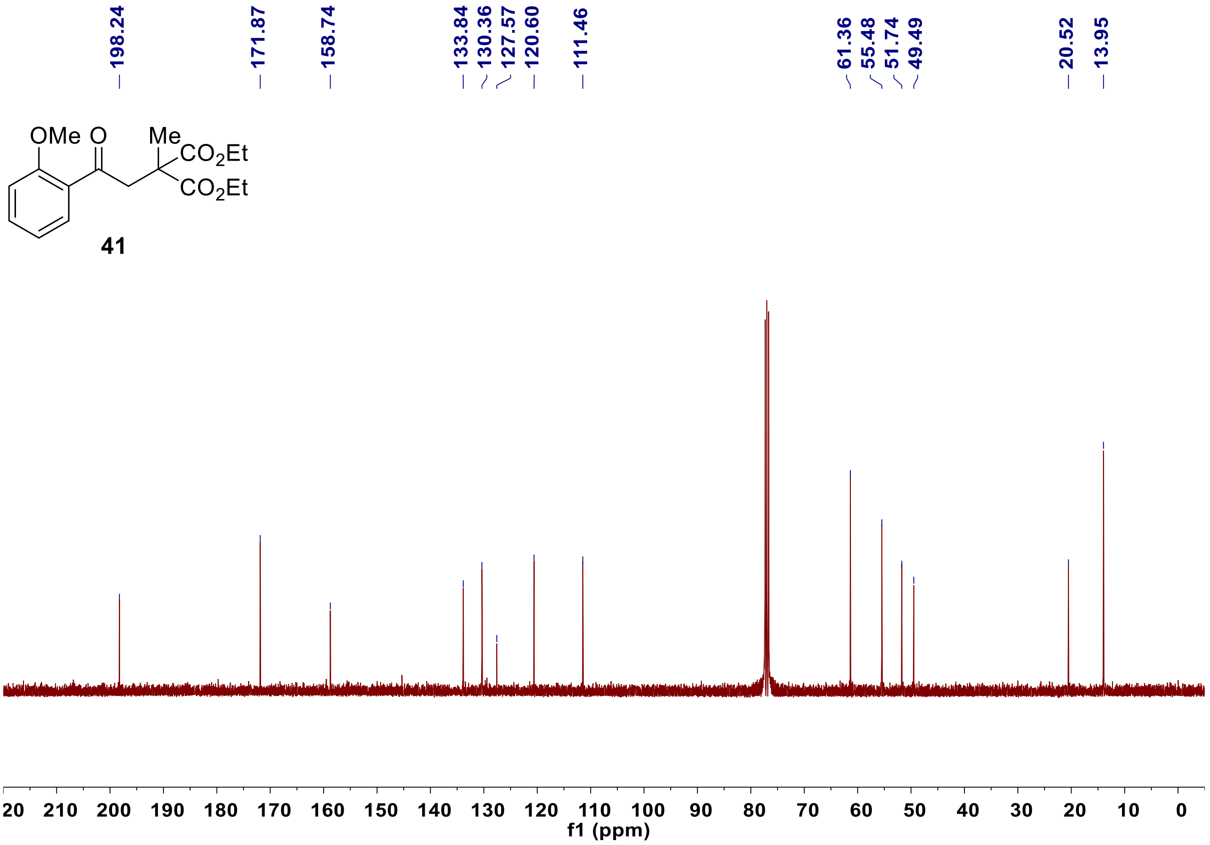

^{1}H and ^{13}C NMR spectra for compound 42

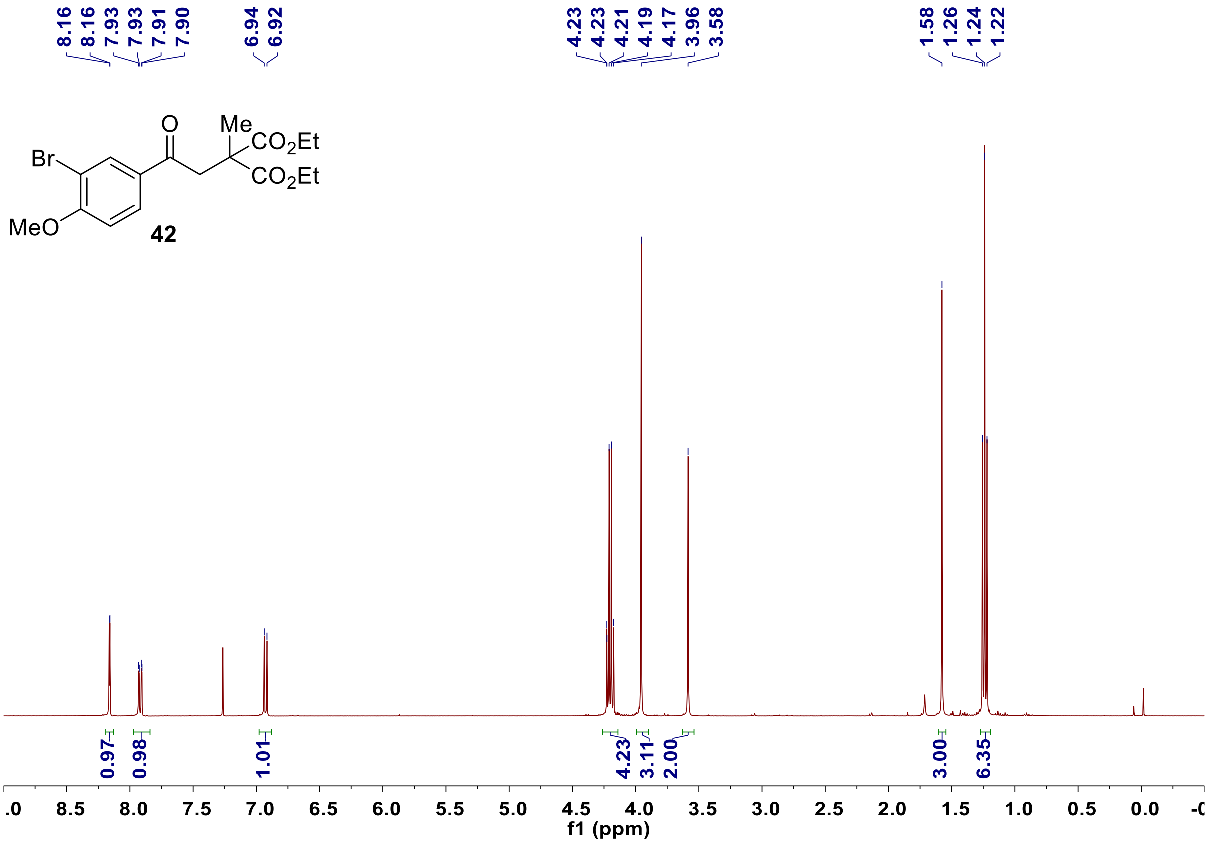

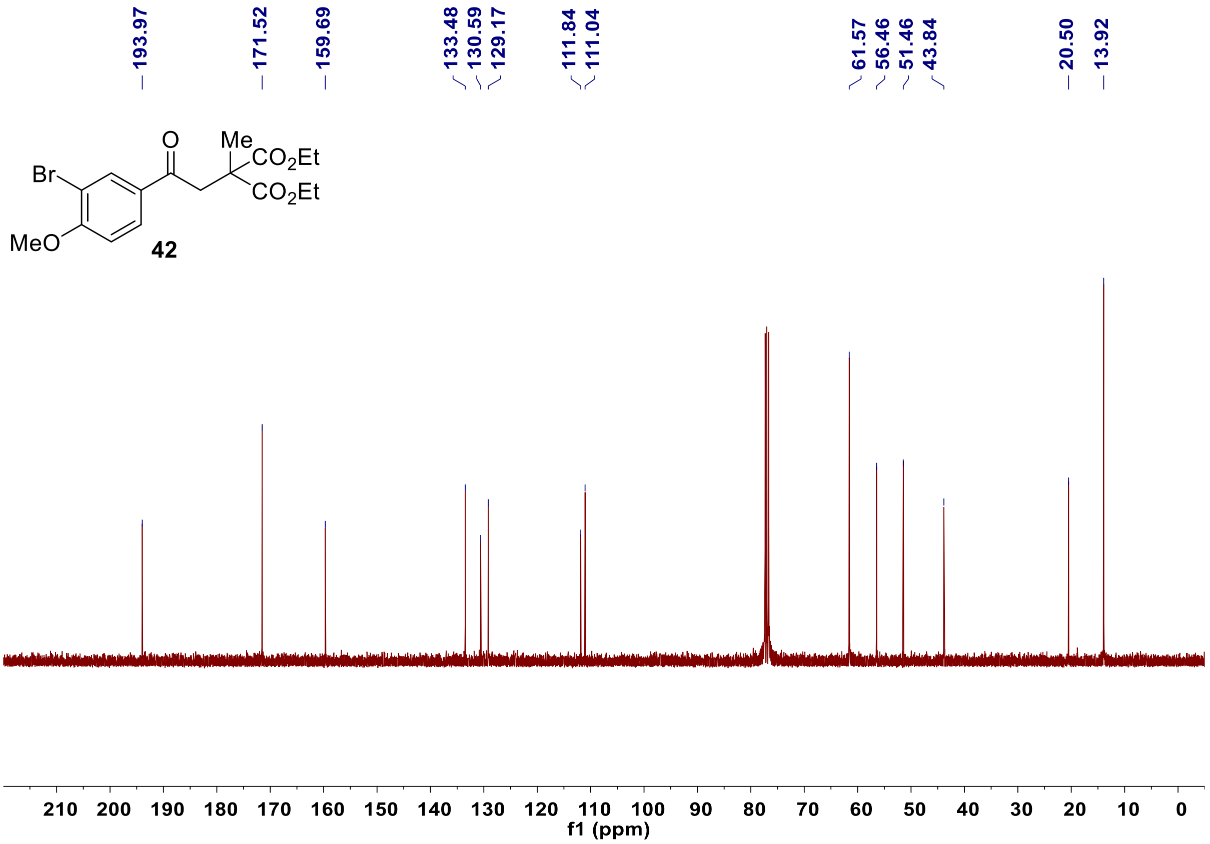

^1H and ^{13}C NMR spectra for compound 43

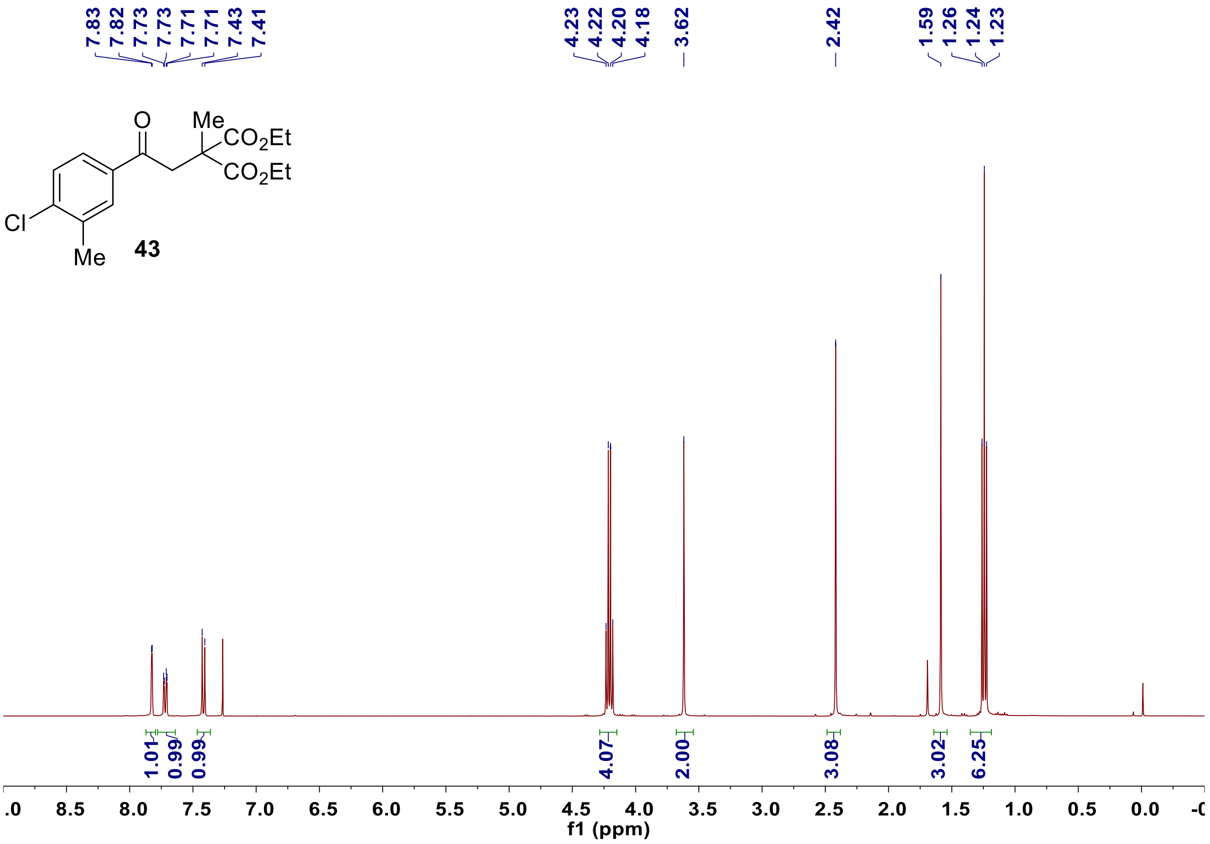

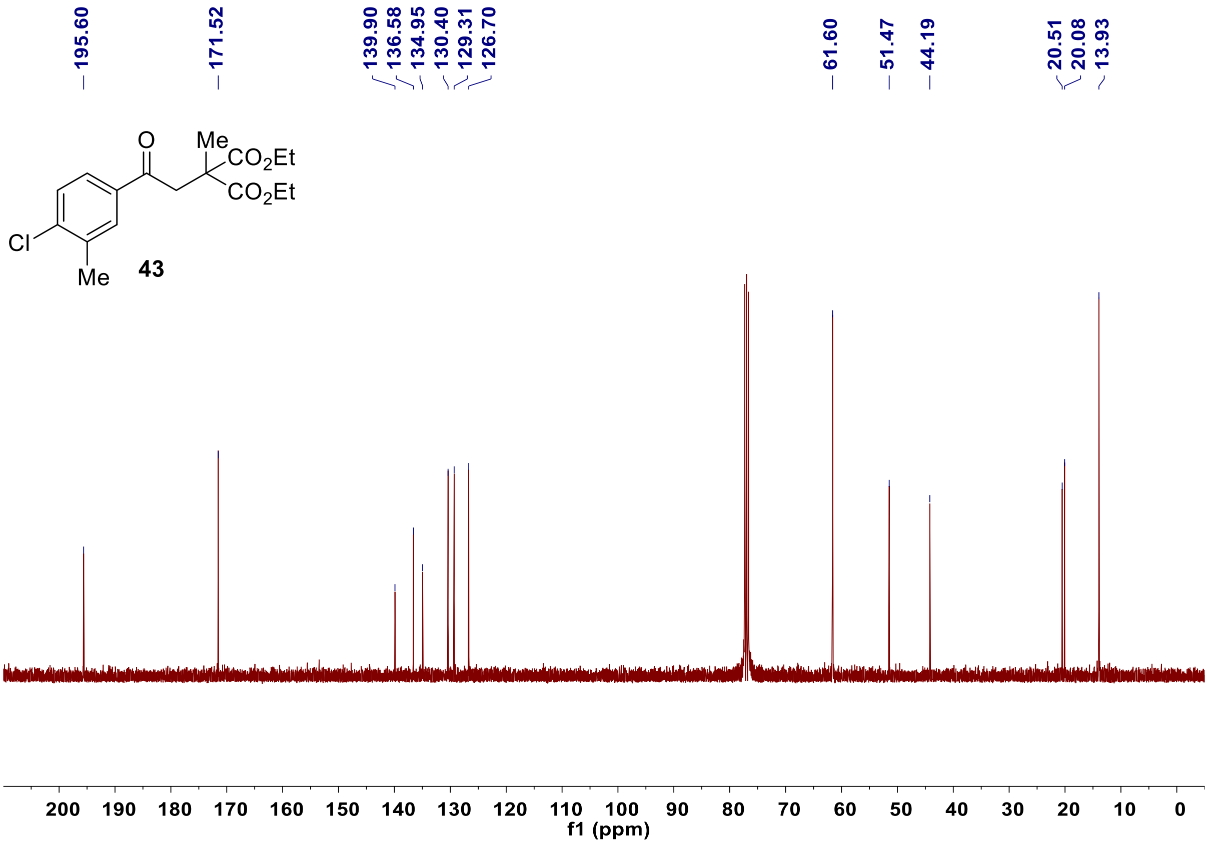

^1H, ^{19}F and ^{13}C NMR spectra for compound 44

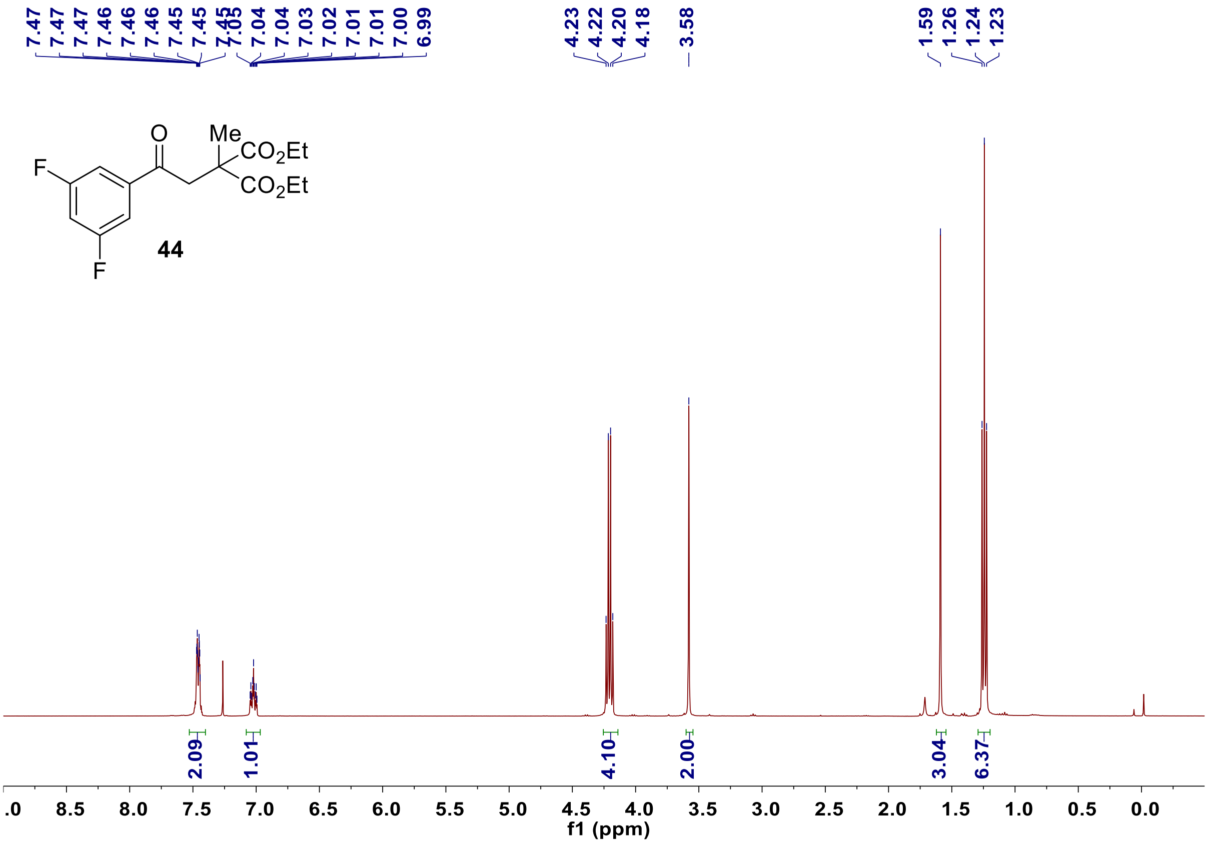

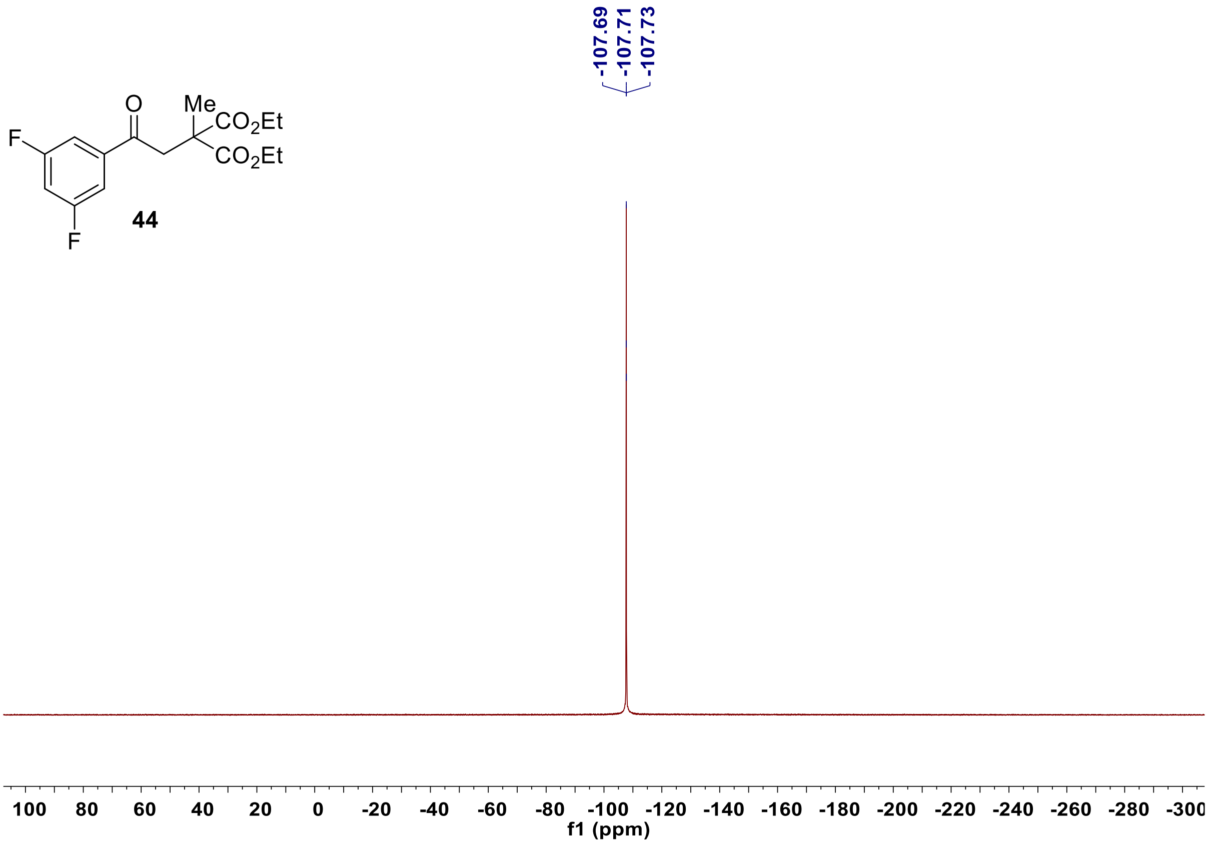
-107.69
-107.71
-107.73
O
Me
CO2Et
CO2Et
F
F
44
100 80 60 40 20 0 -20 -40 -60 -80 -100 -120 -140 -160 -180 -200 -220 -240 -260 -280 -300
f1 (ppm)

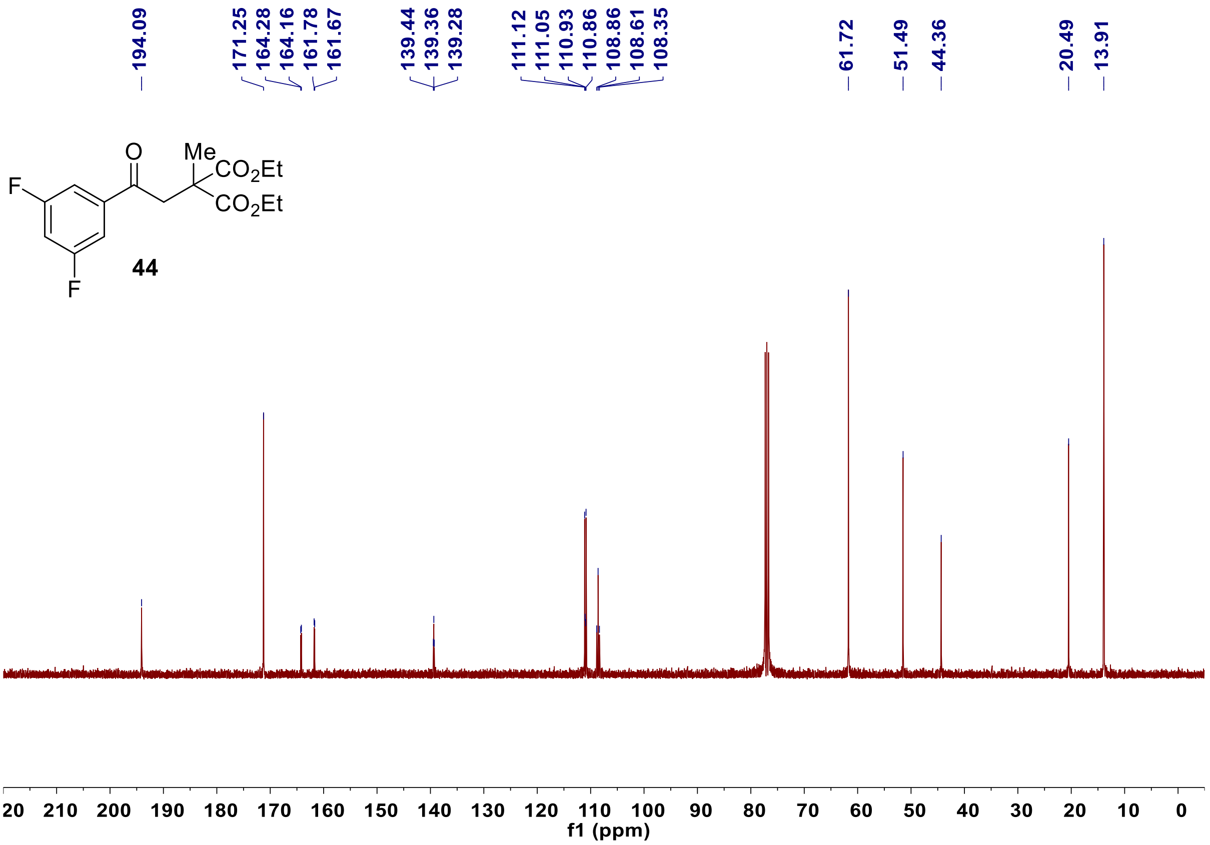
194.09
171.25
164.28
164.16
161.78
161.67
139.44
139.36
139.28
111.12
111.05
110.93
110.86
108.86
108.61
108.35
61.72
51.49
44.36
20.49
13.91
O
Me
CO2Et
CO2Et
F
F
44
20 210 200 190 180 170 160 150 140 130 120 110 100 90 80 70 60 50 40 30 20 10 0
f1 (ppm)

^{1}H and ^{13}C NMR spectra for compound 45

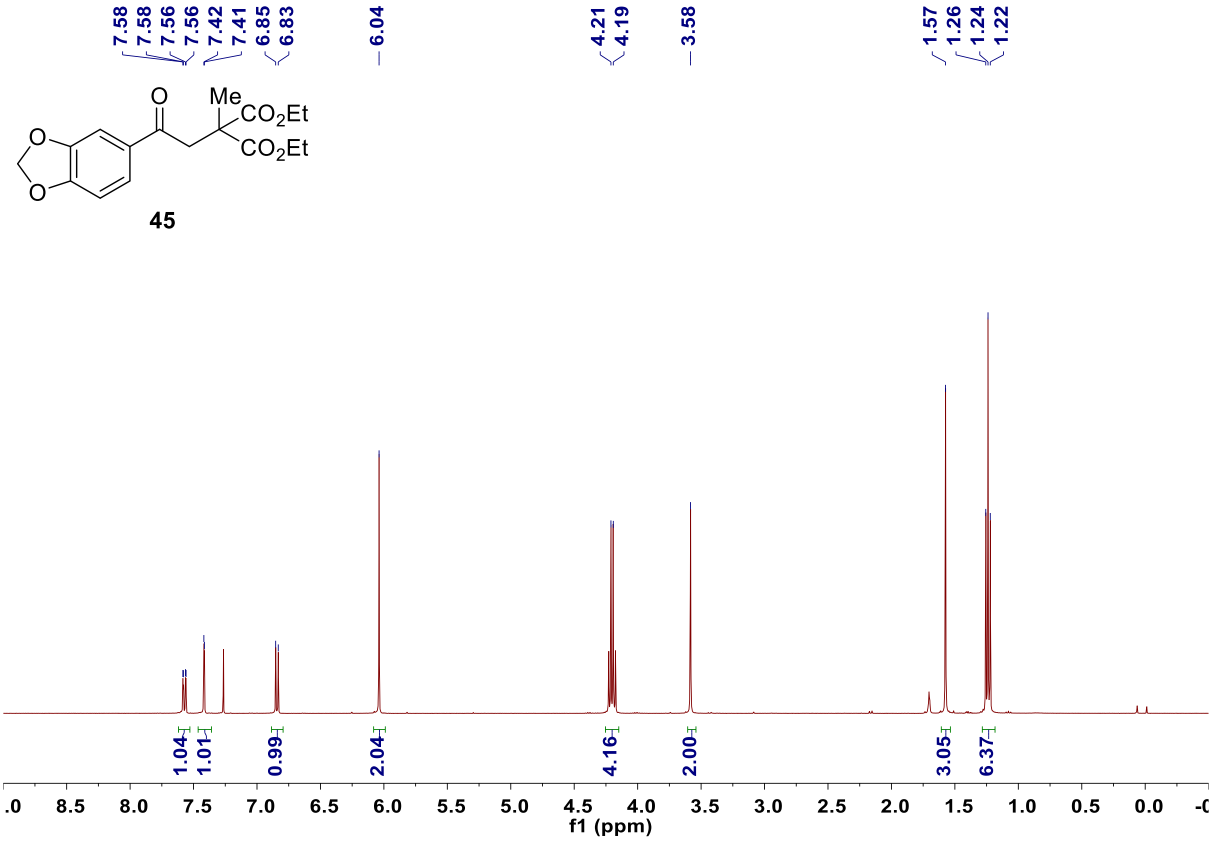

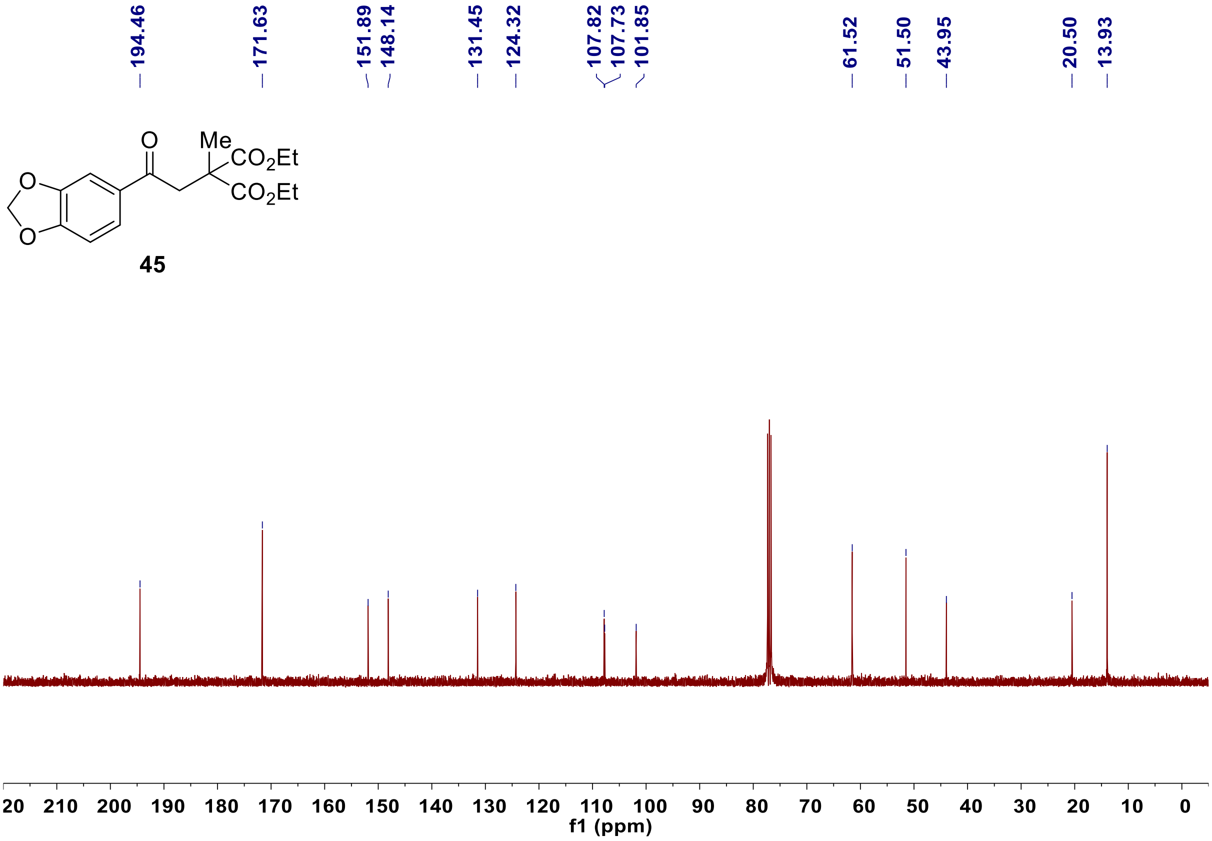

^{1}H and ^{13}C NMR spectra for compound 46

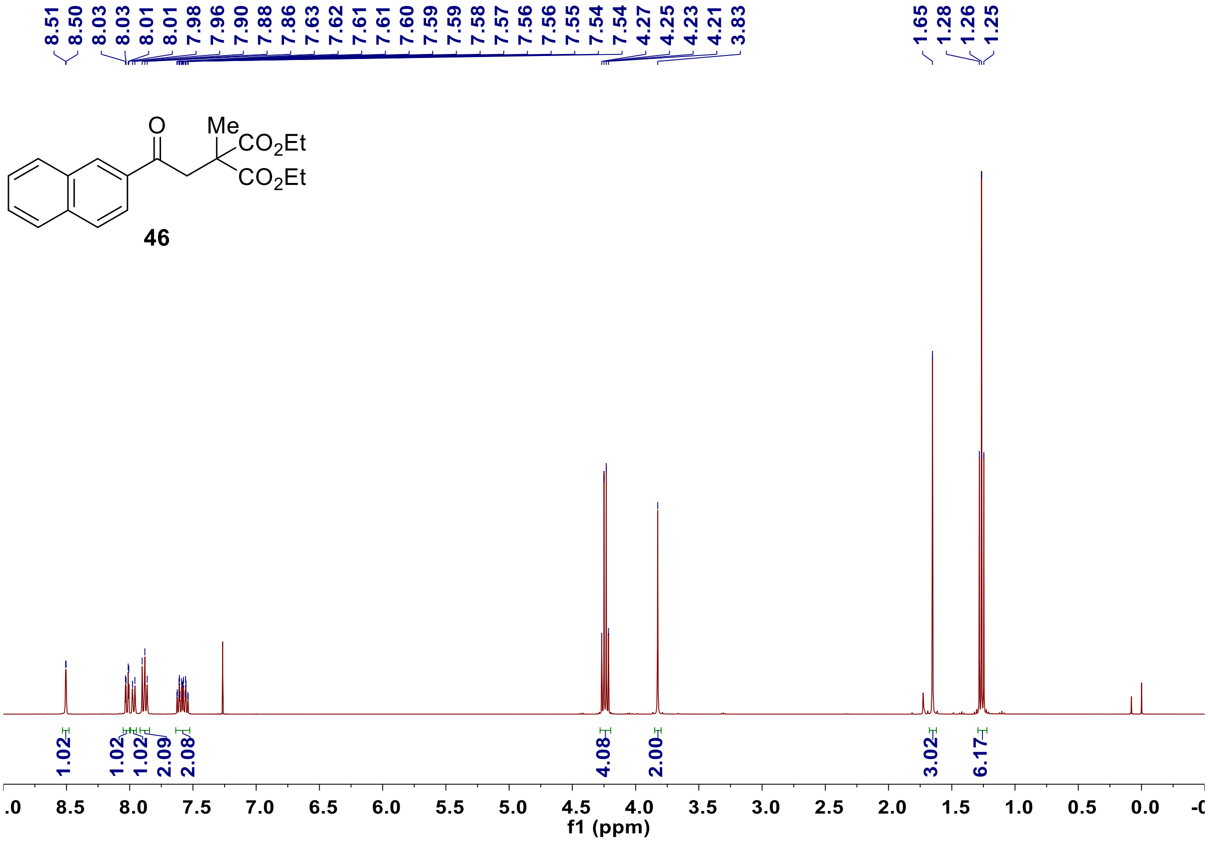

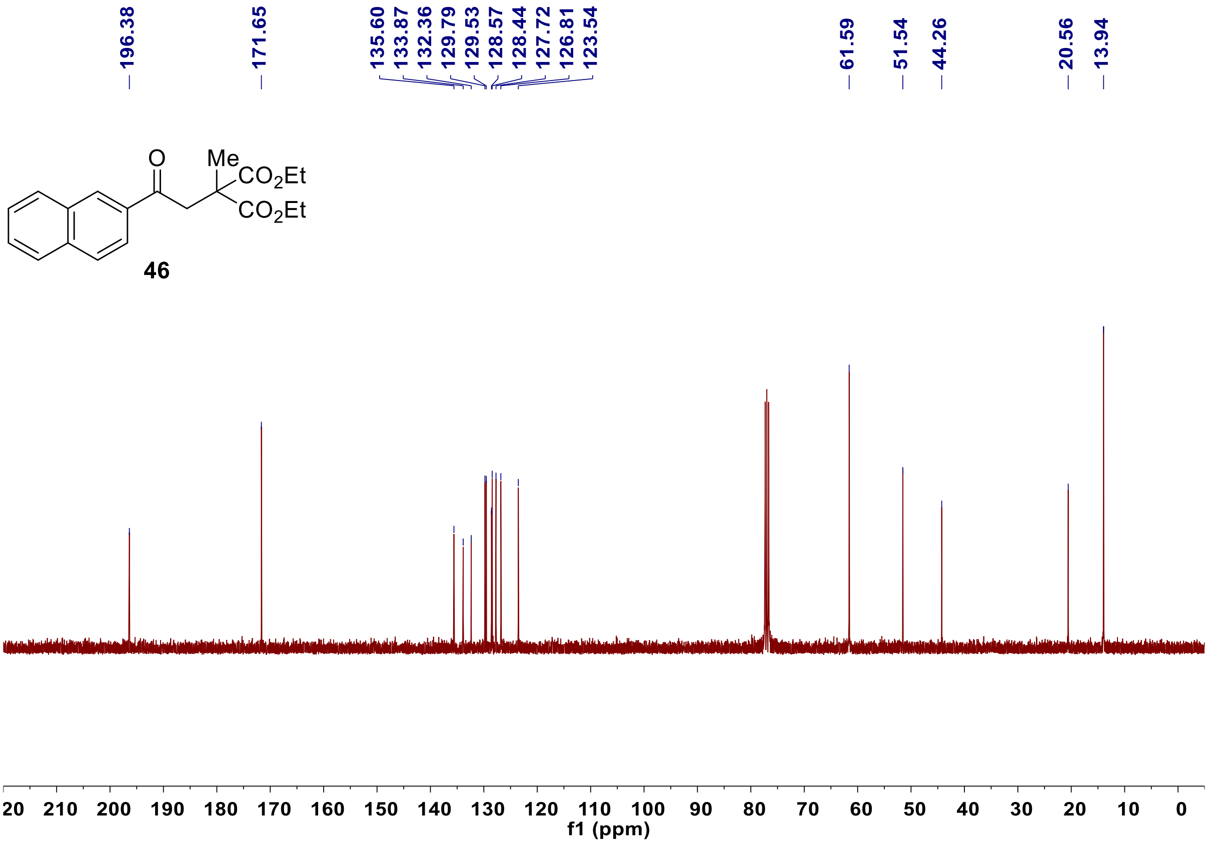

^1H and ^{13}C NMR spectra for compound 47

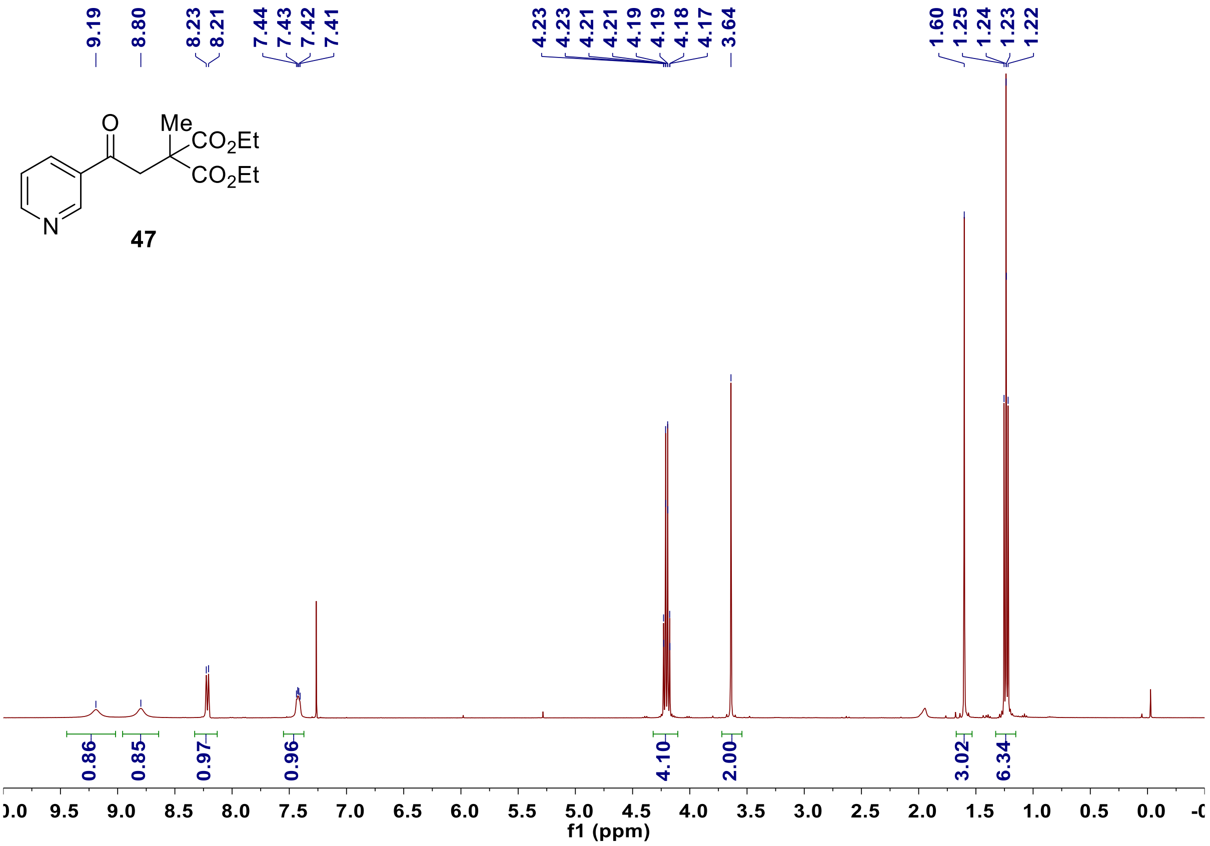

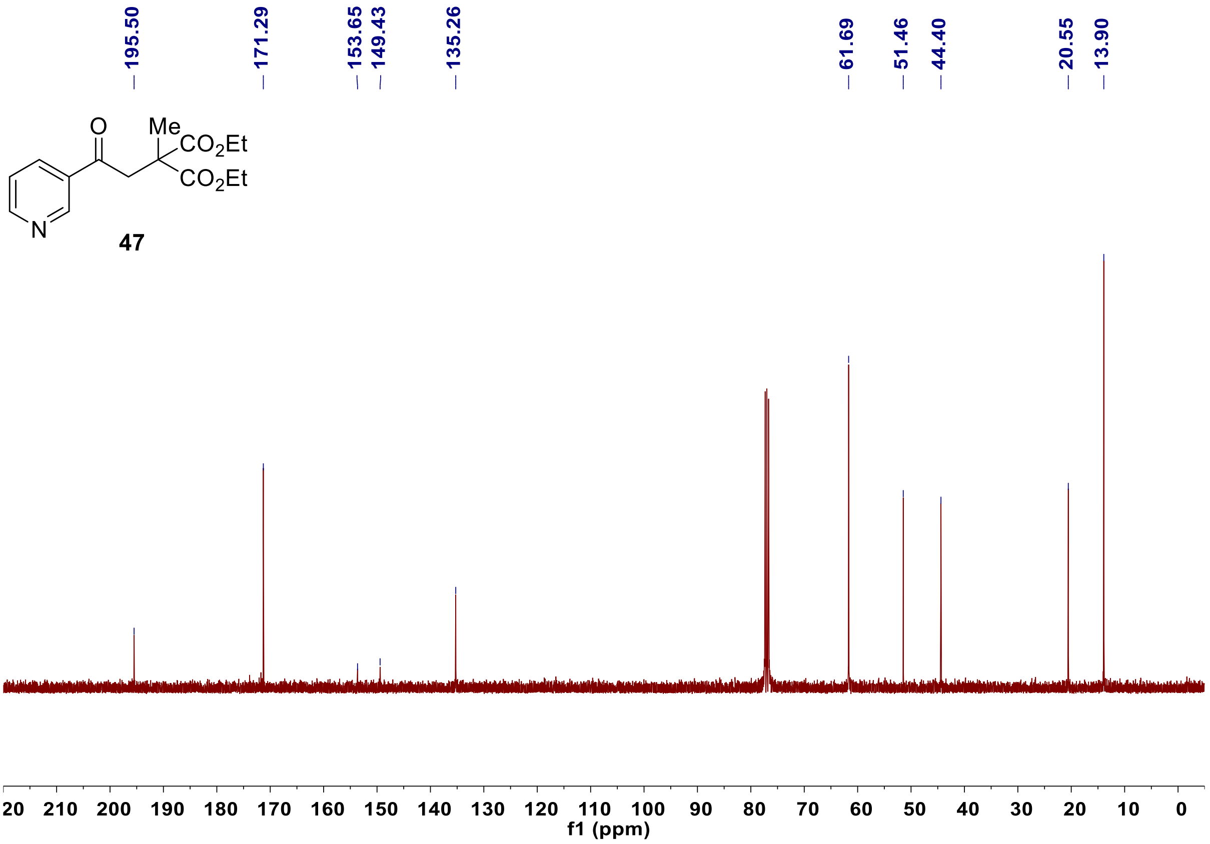

^{1}H and ^{13}C NMR spectra for compound 48

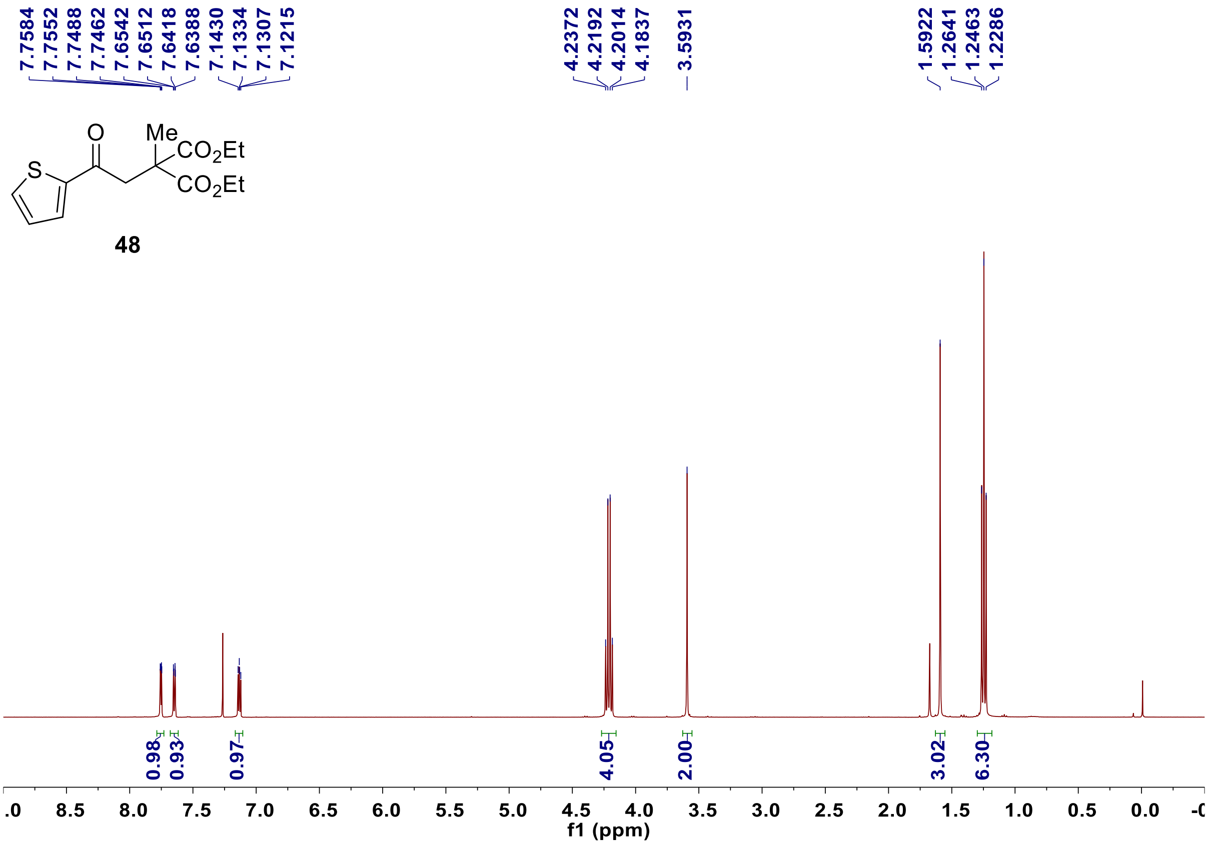

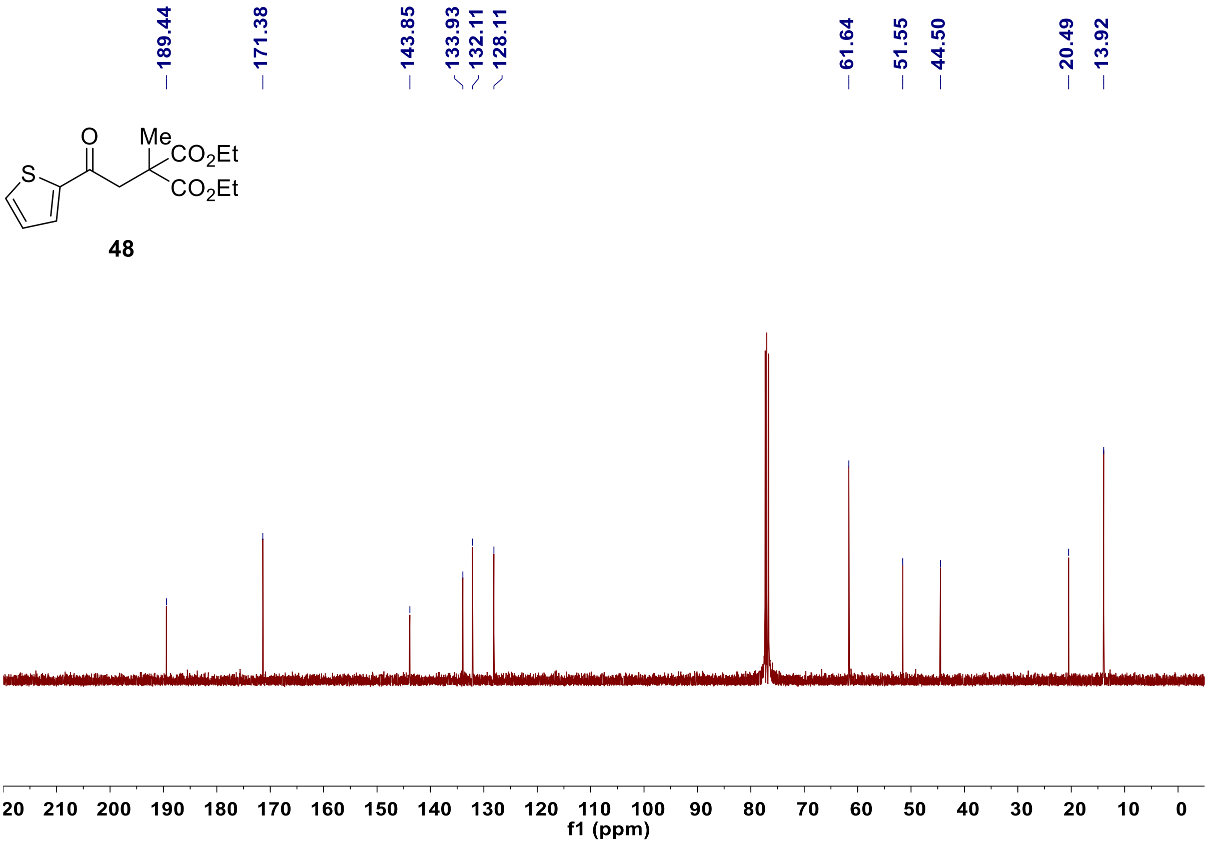

^{1}H and ^{13}C NMR spectra for compound 49

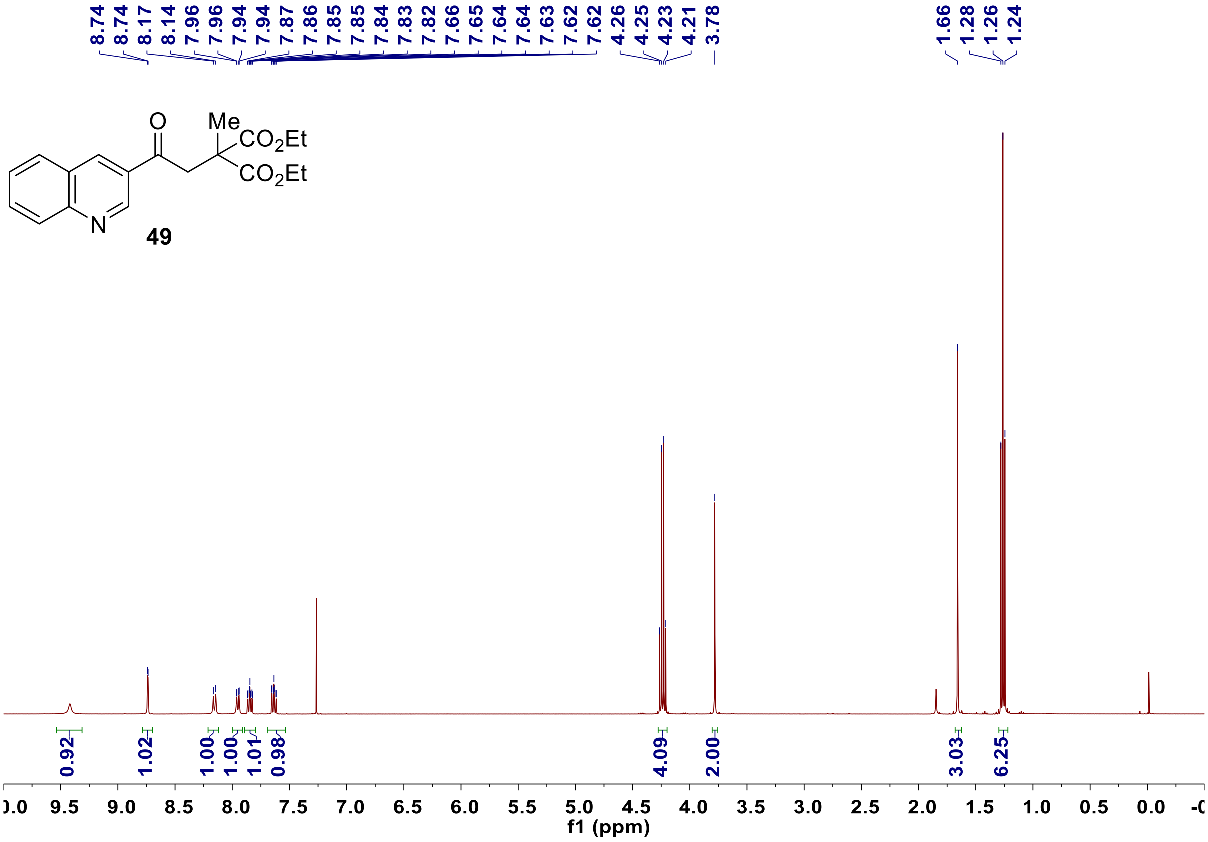

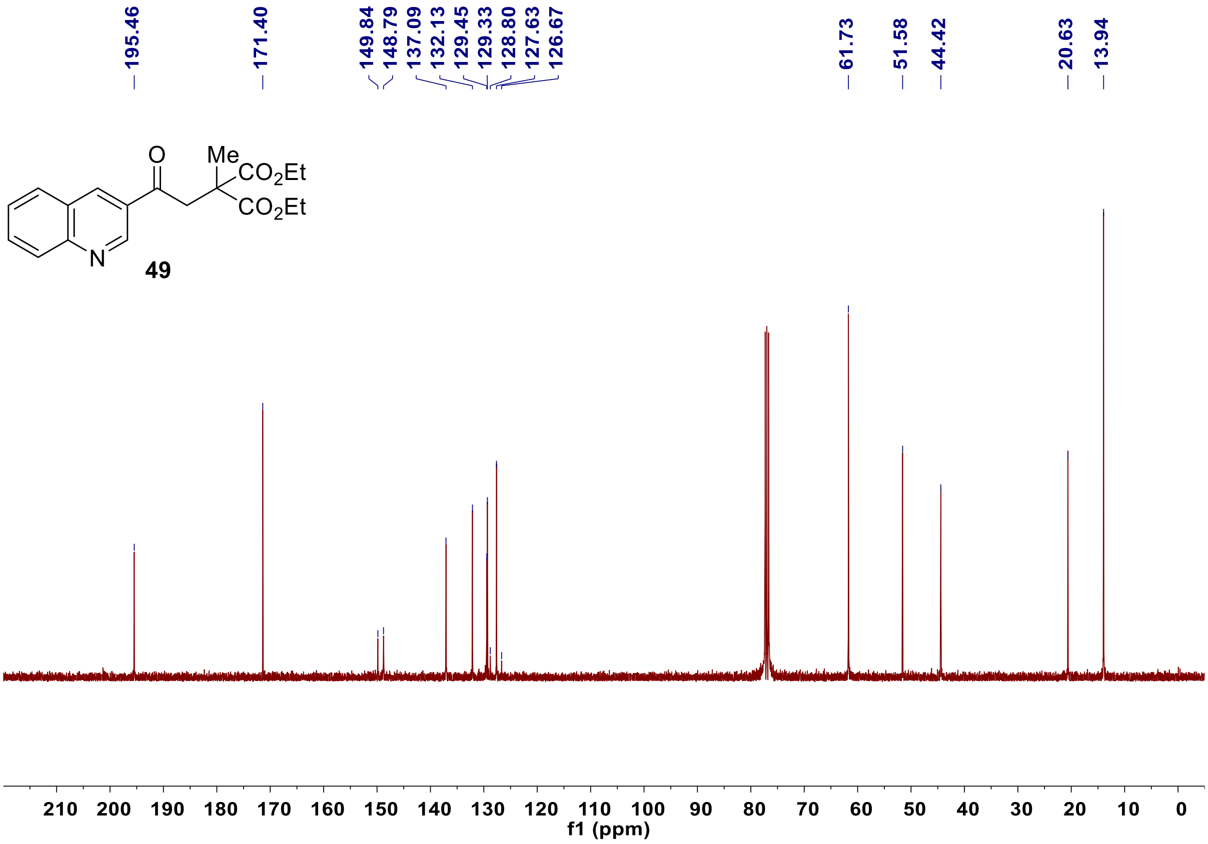

^{1}H and ^{13}C NMR spectra for compound 50

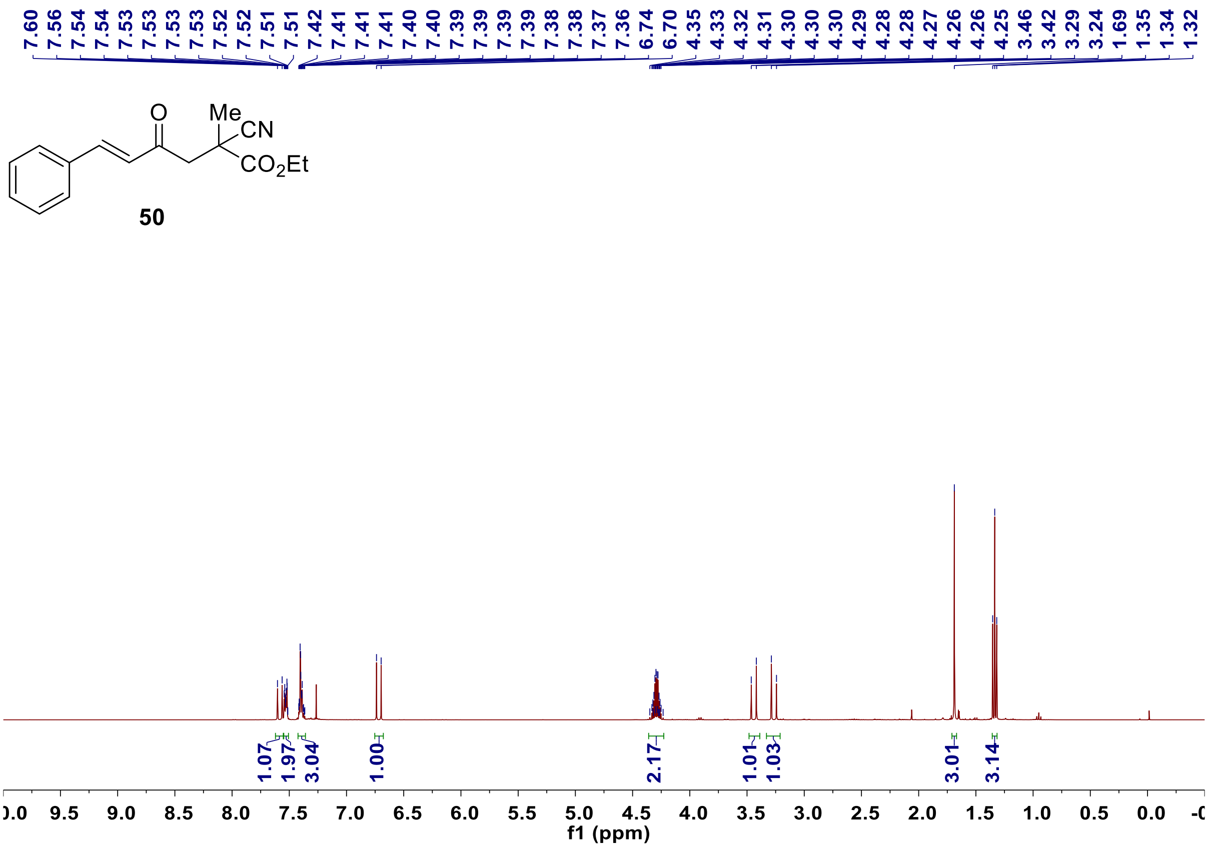

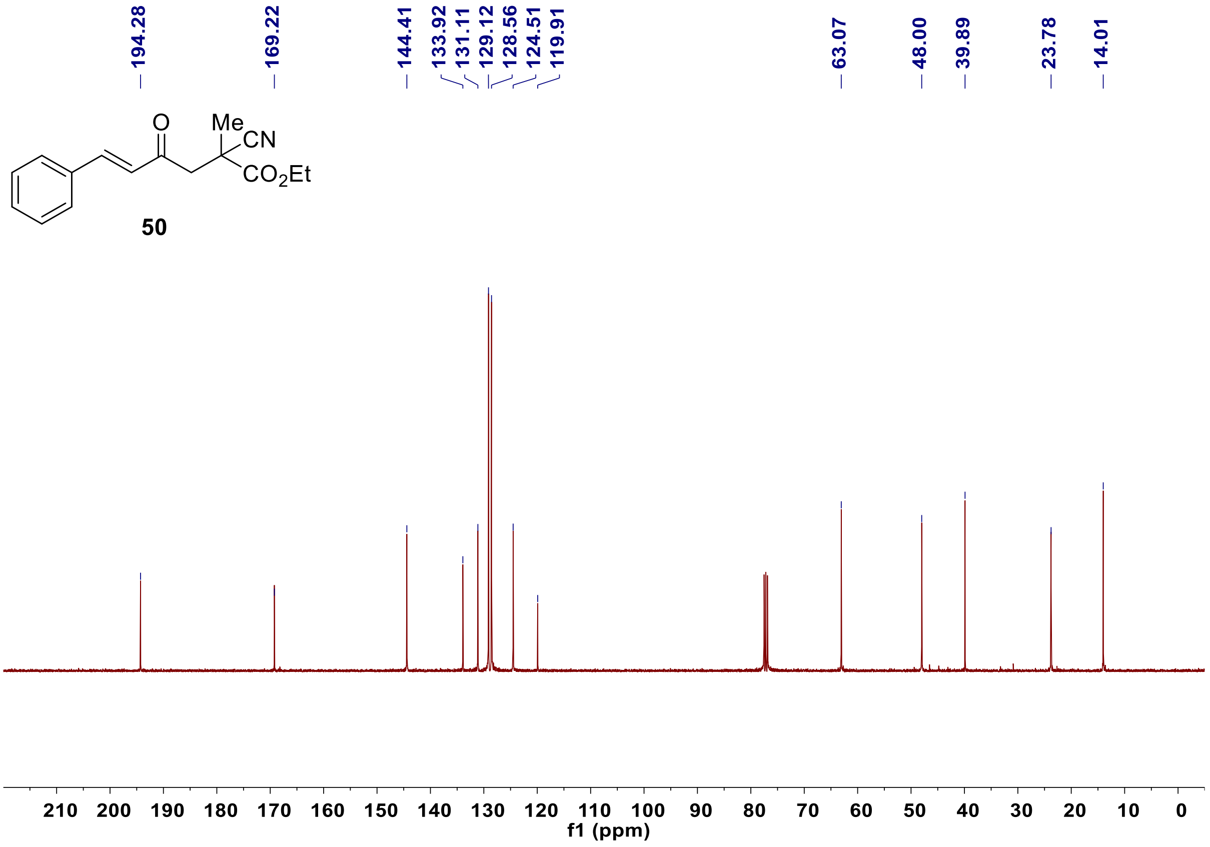

^{1}H and ^{13}C NMR spectra for compound 51A

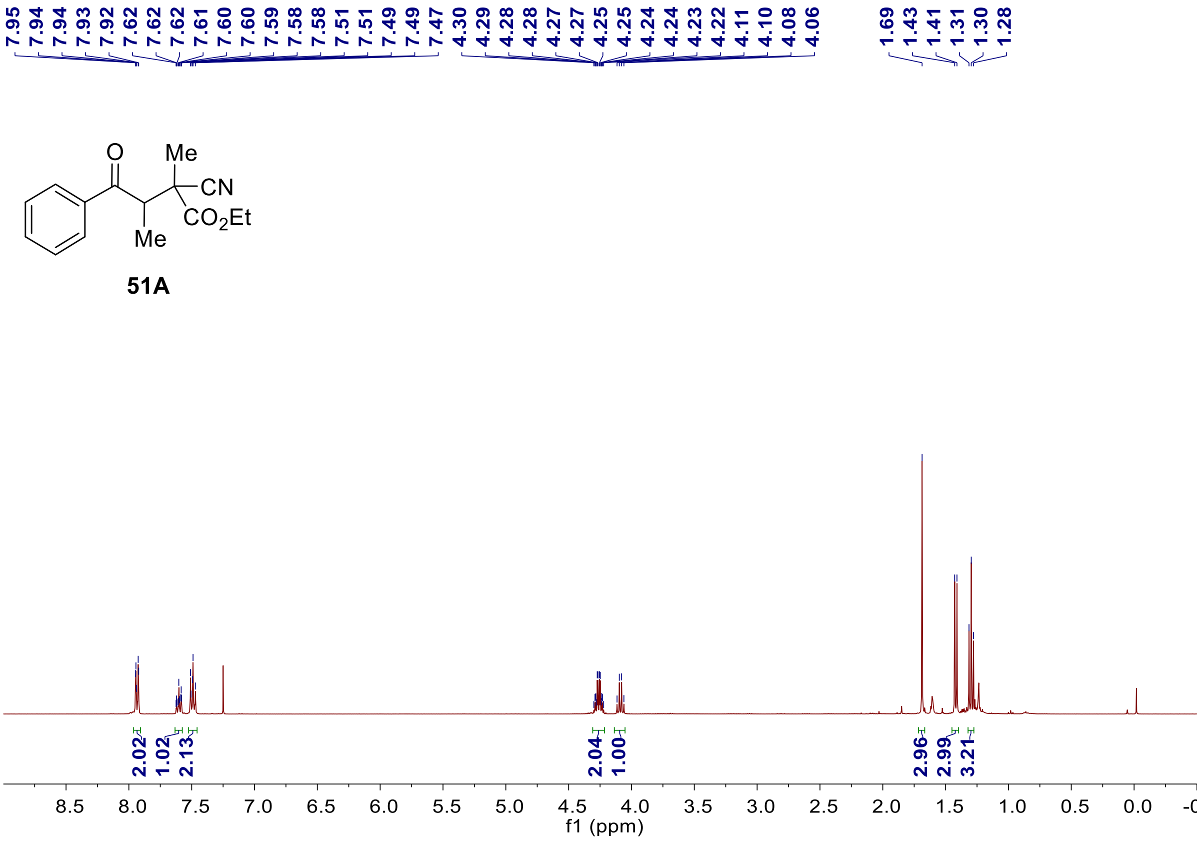

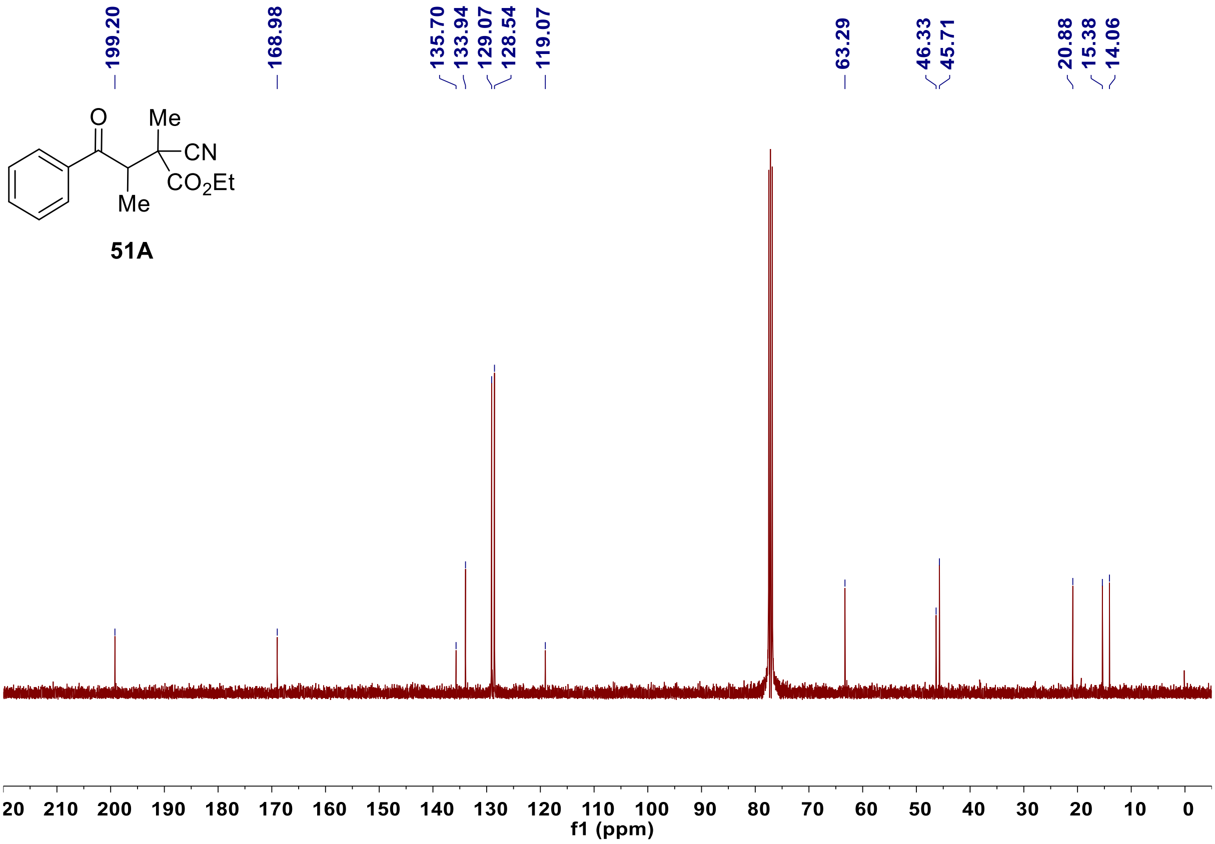

^{1}H and ^{13}C NMR spectra for compound 51B

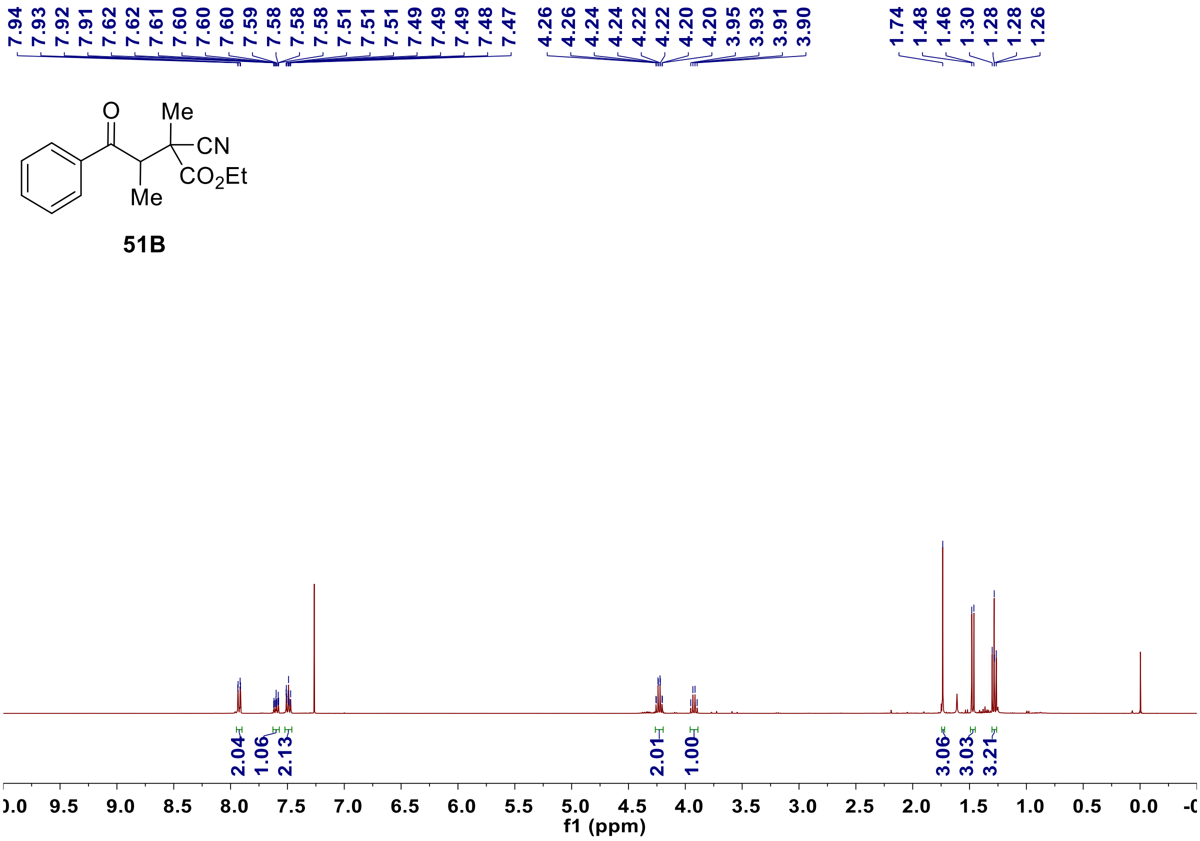

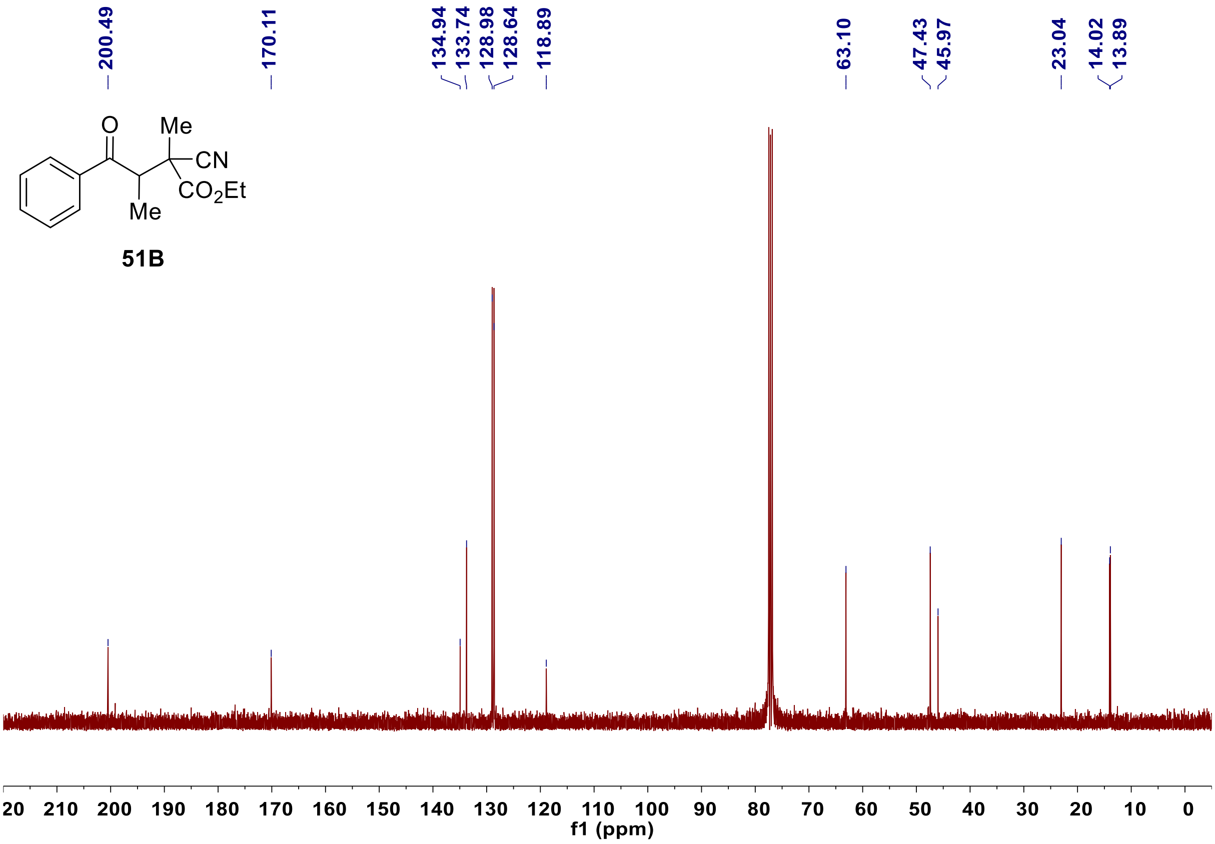

^{1}H and ^{13}C NMR spectra for compound 52A

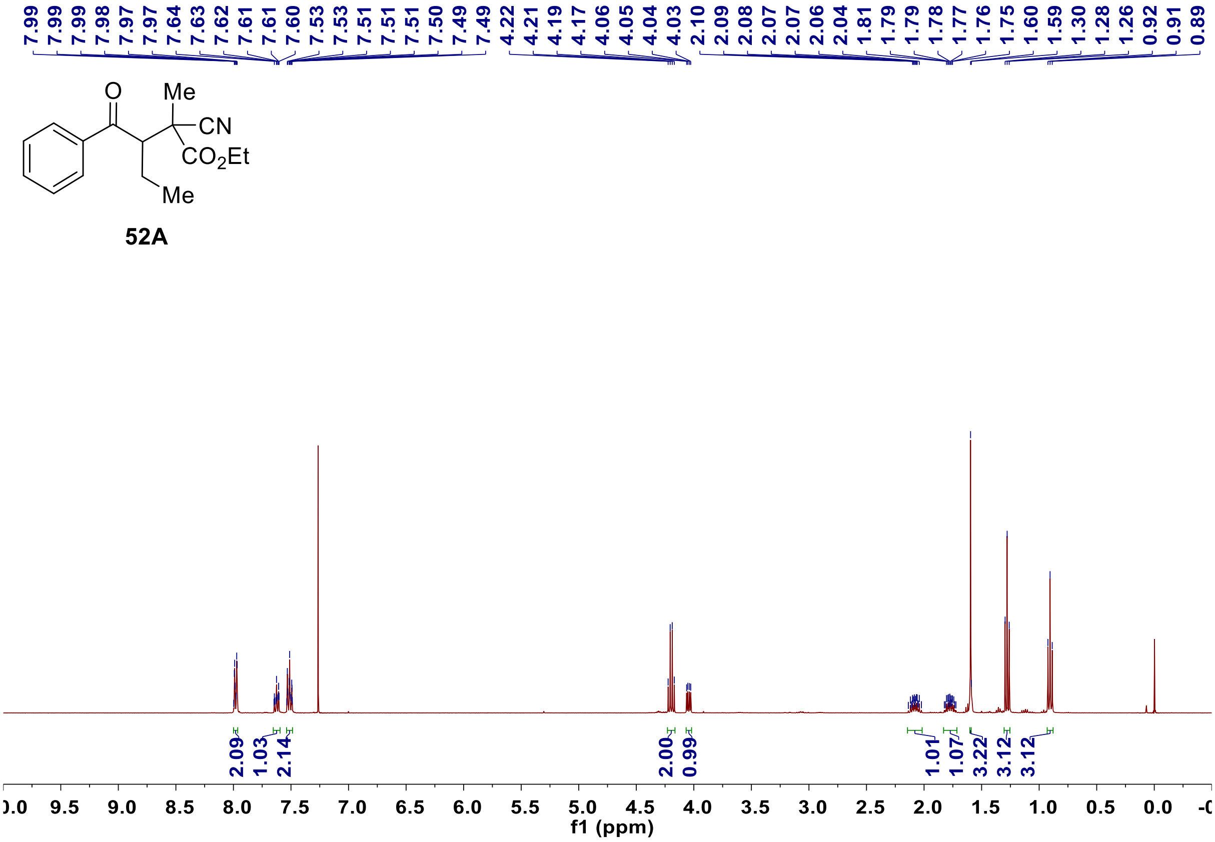

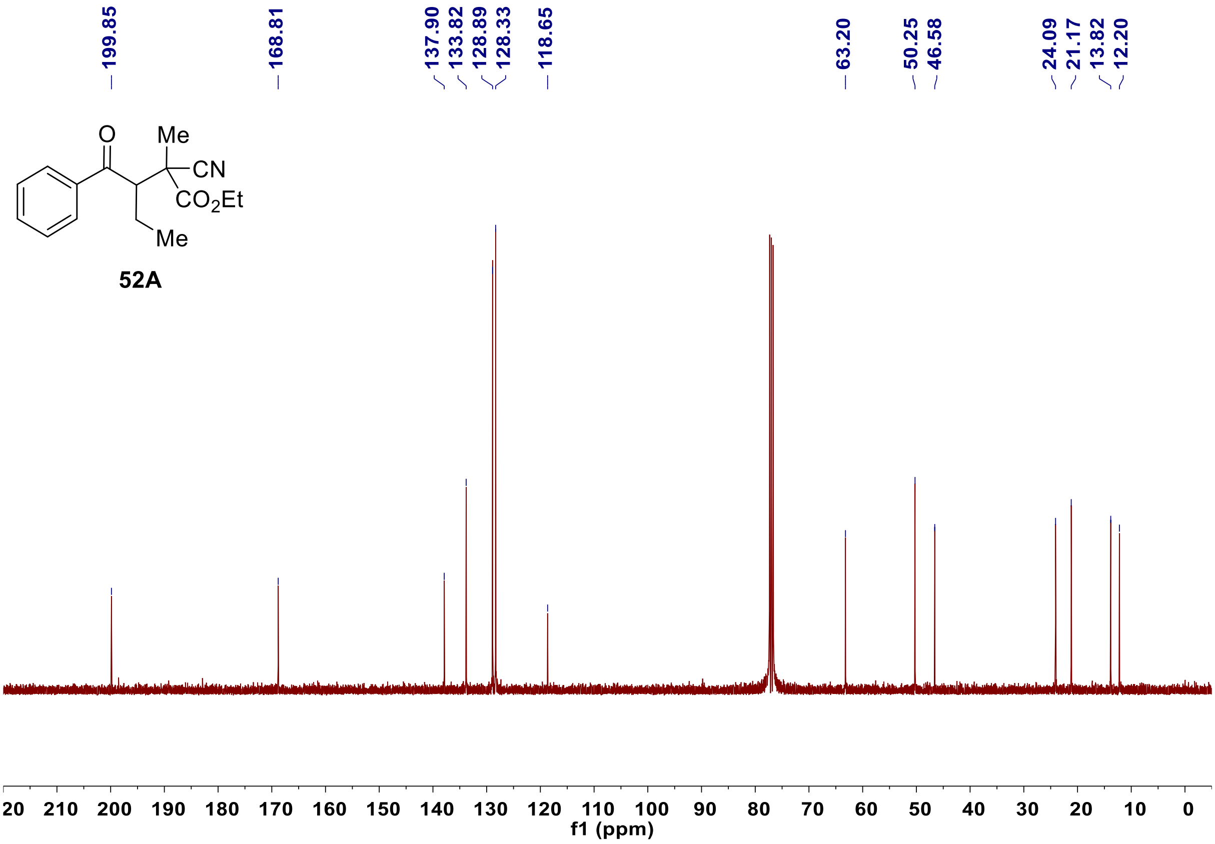

^{1}H and ^{13}C NMR spectra for compound 52B

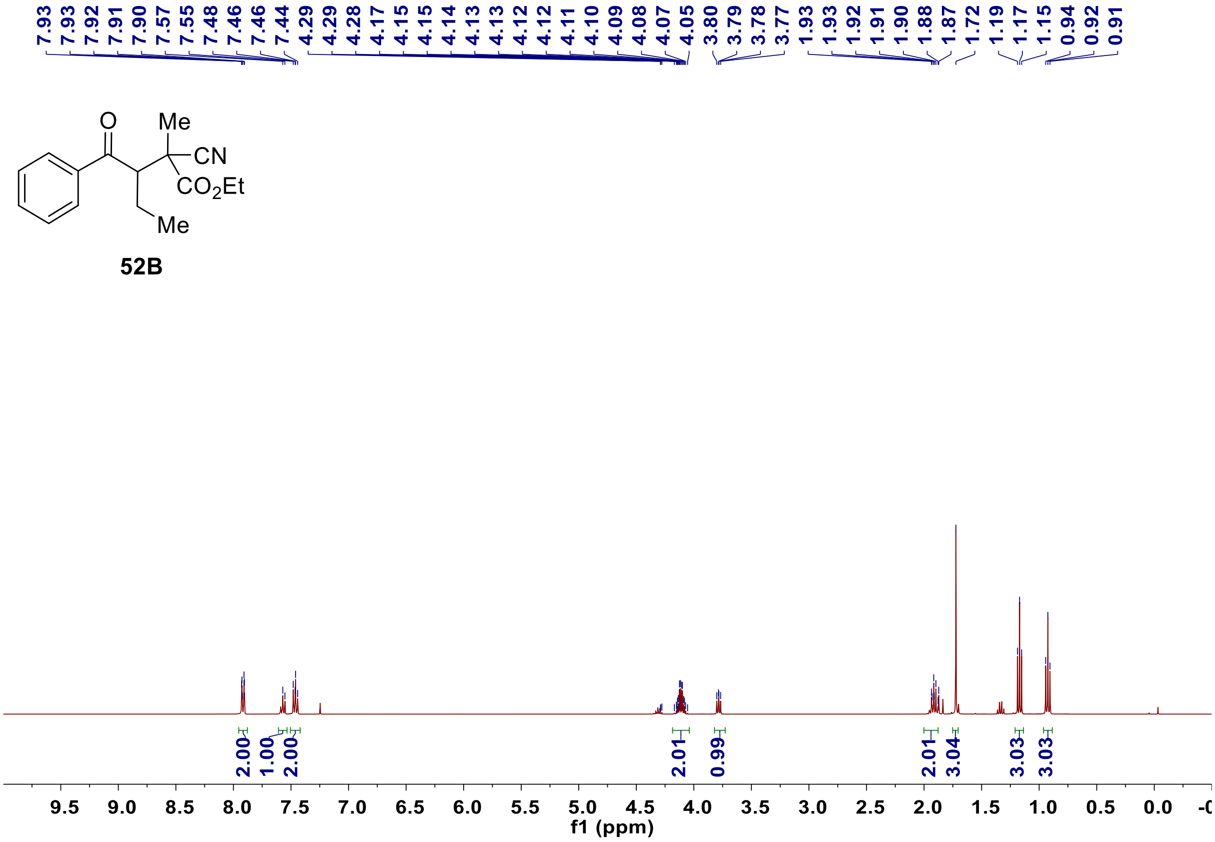

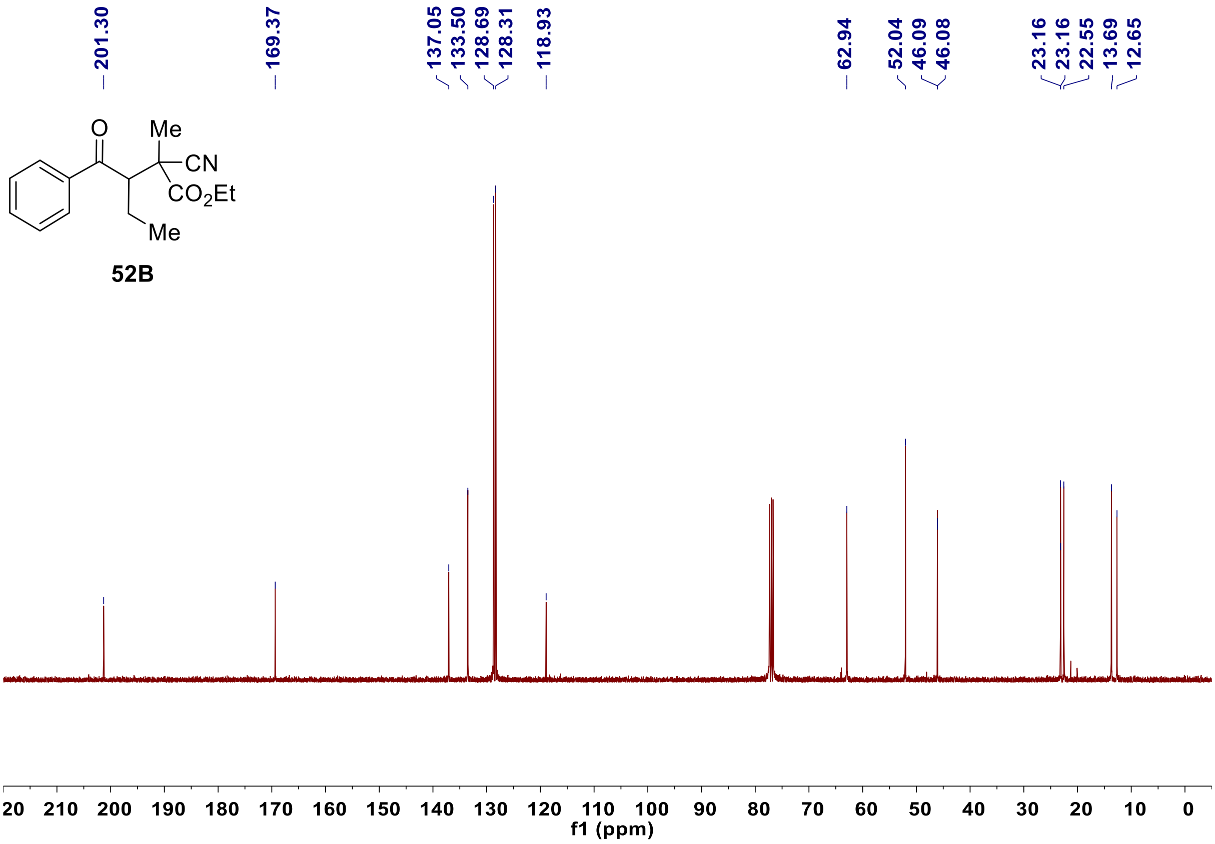

^{1}H and ^{13}C NMR spectra for compound 53A

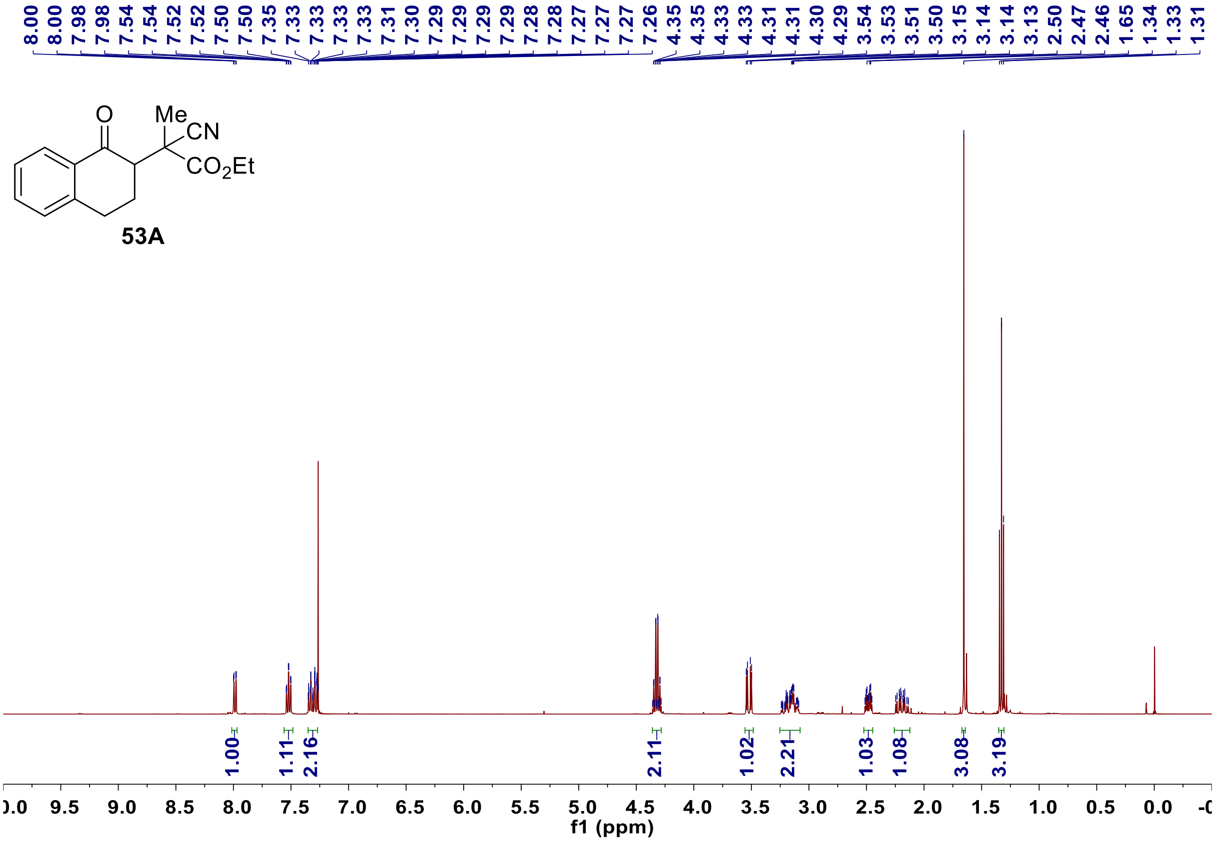

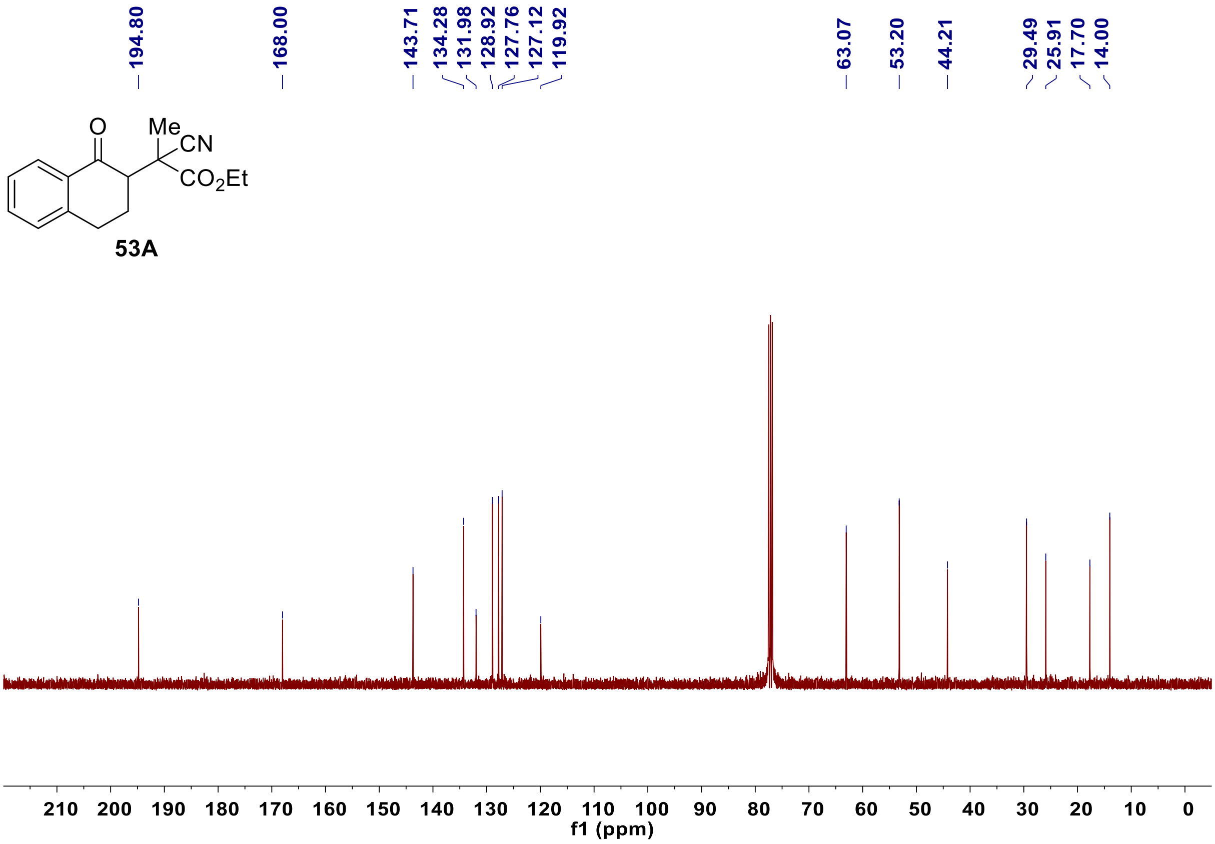

^{1}H and ^{13}C NMR spectra for compound 53B

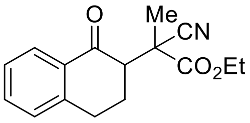

53B

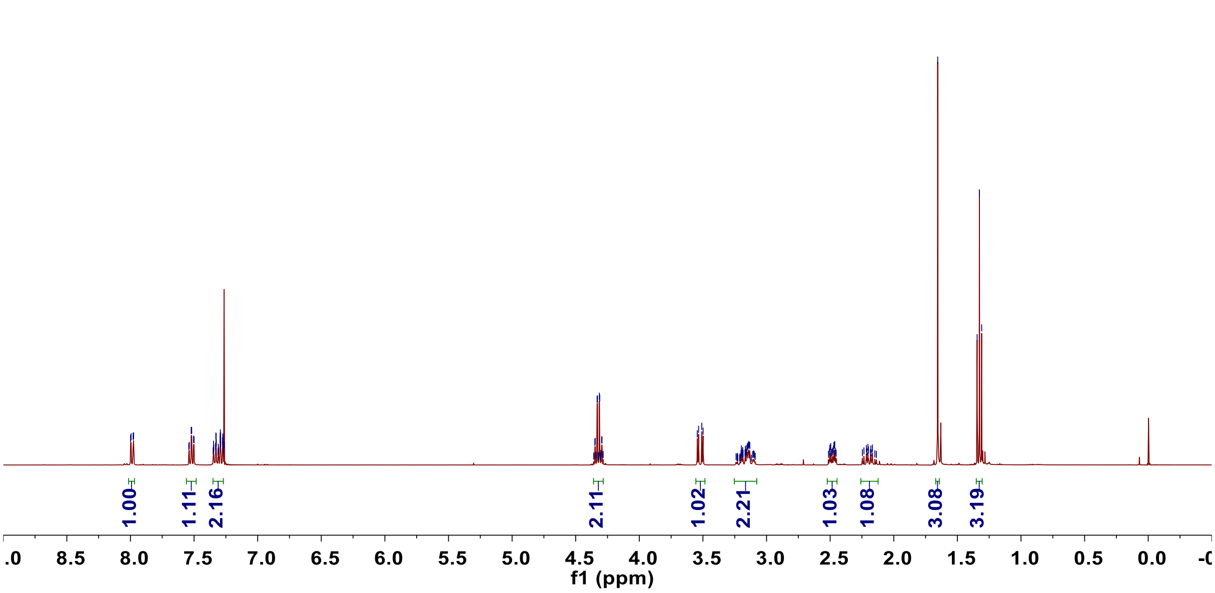

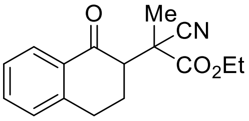

53B

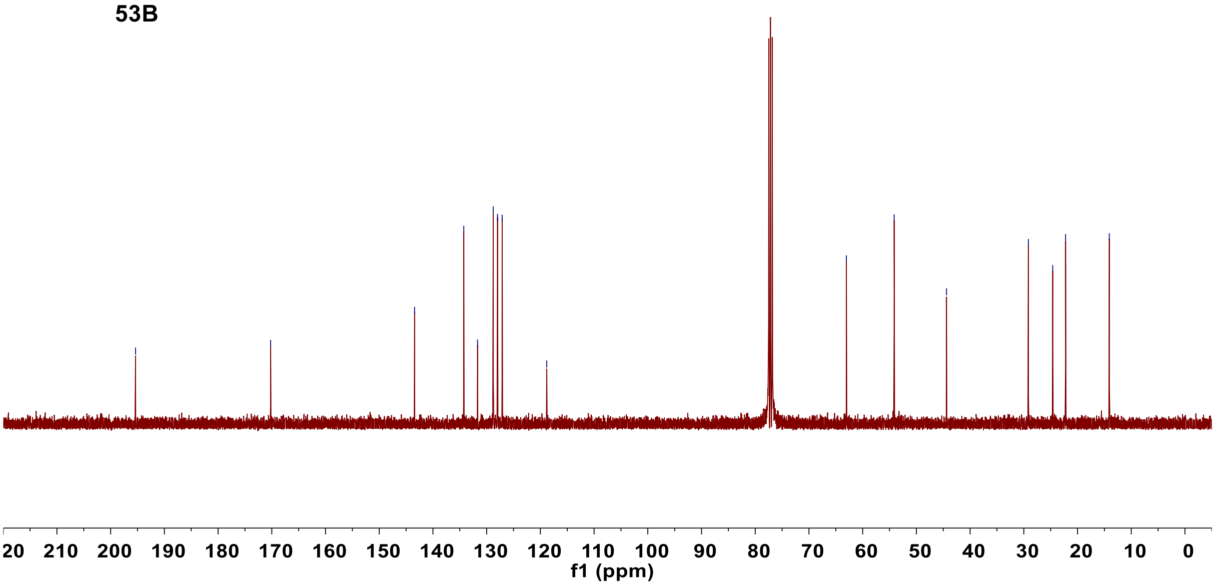

^{1}H and ^{13}C NMR spectra for compound 54

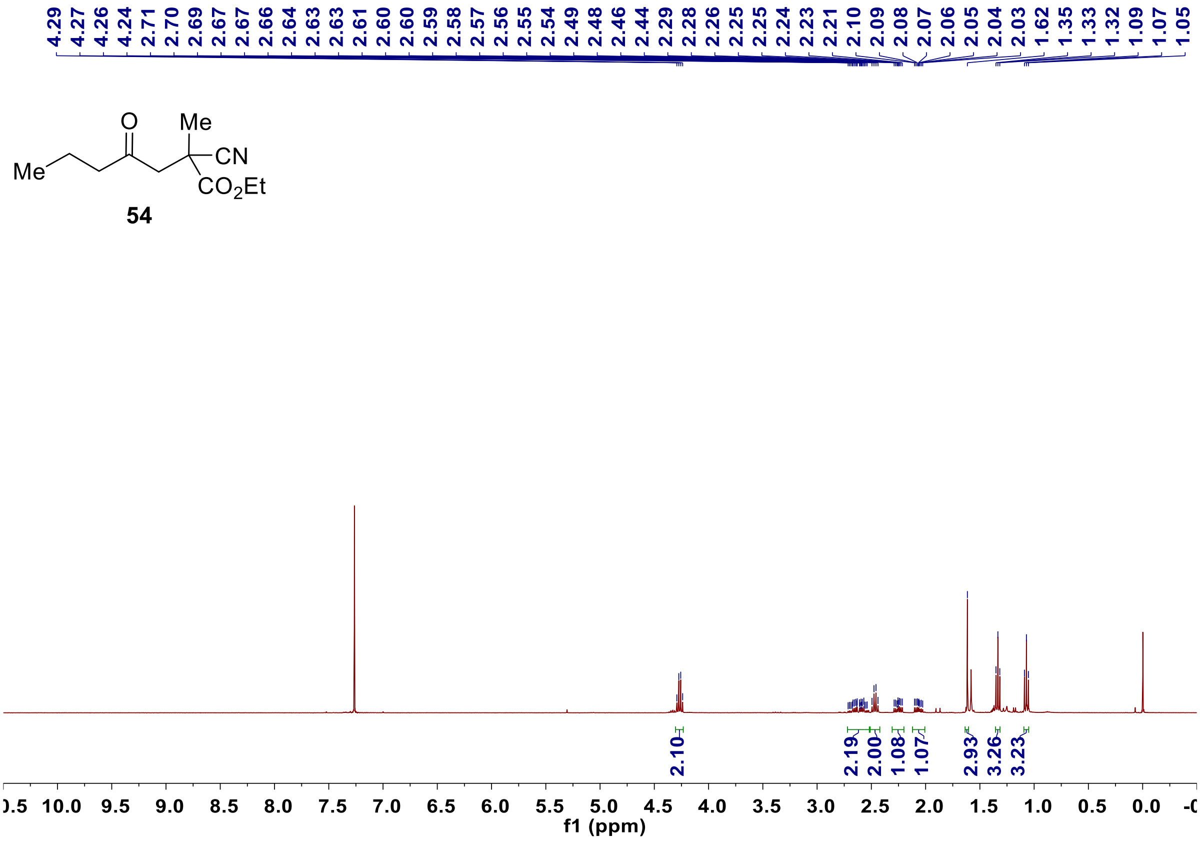

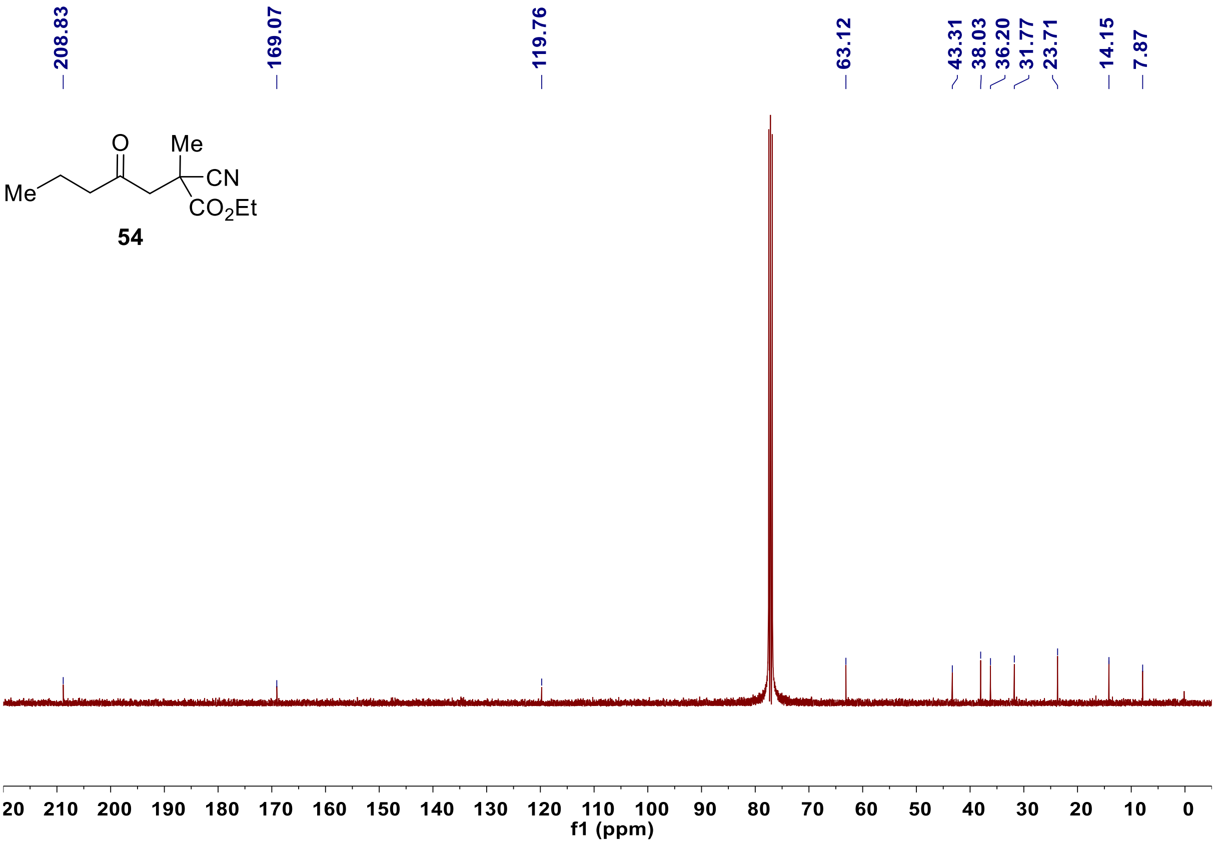

^{1}H and ^{13}C NMR spectra for compound 55

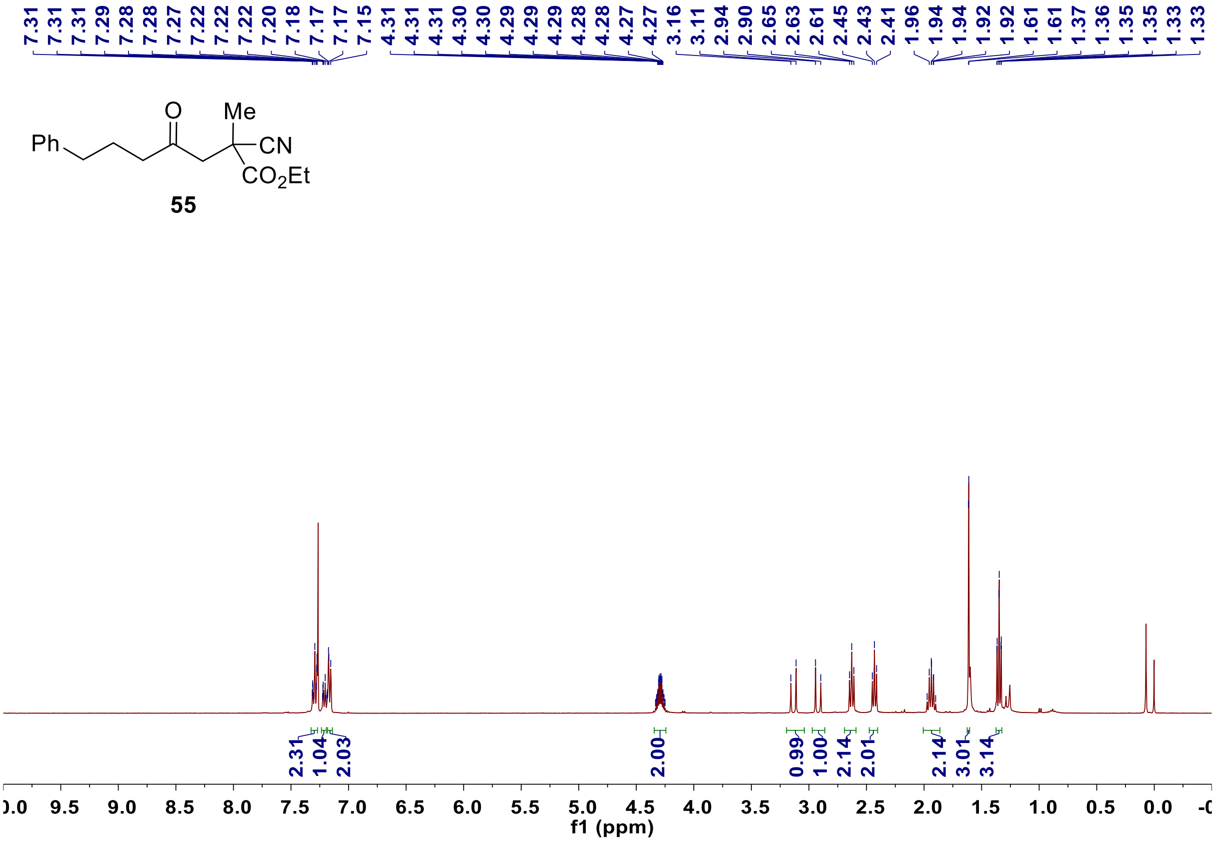

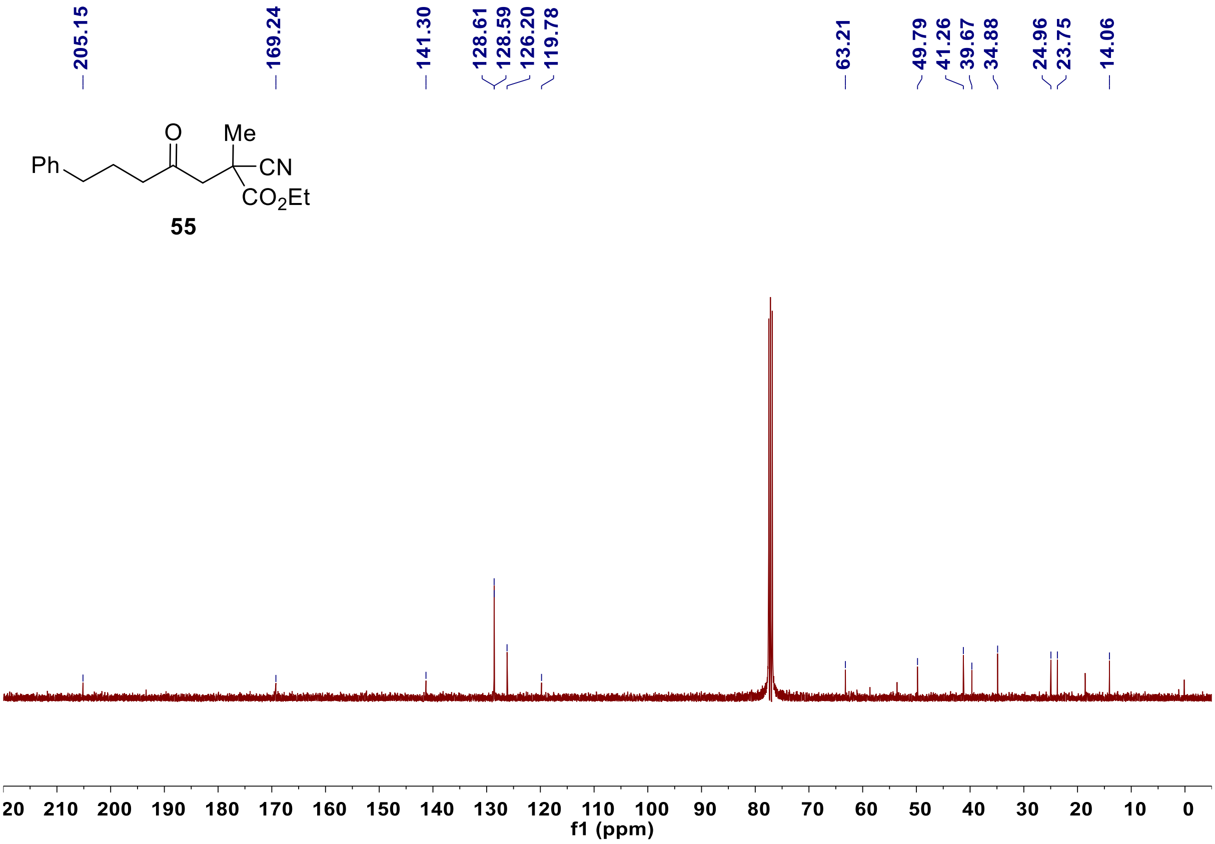

^{1}H and ^{13}C NMR spectra for compound 56

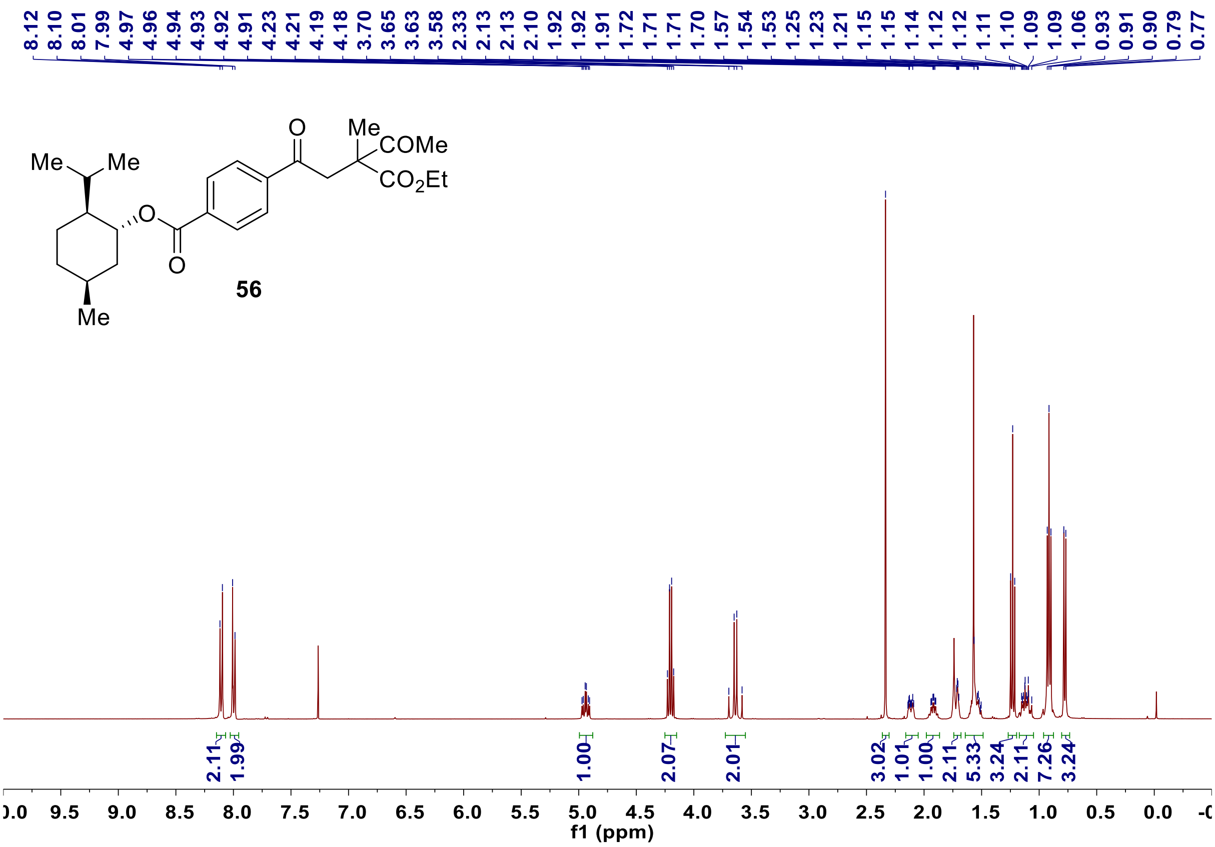

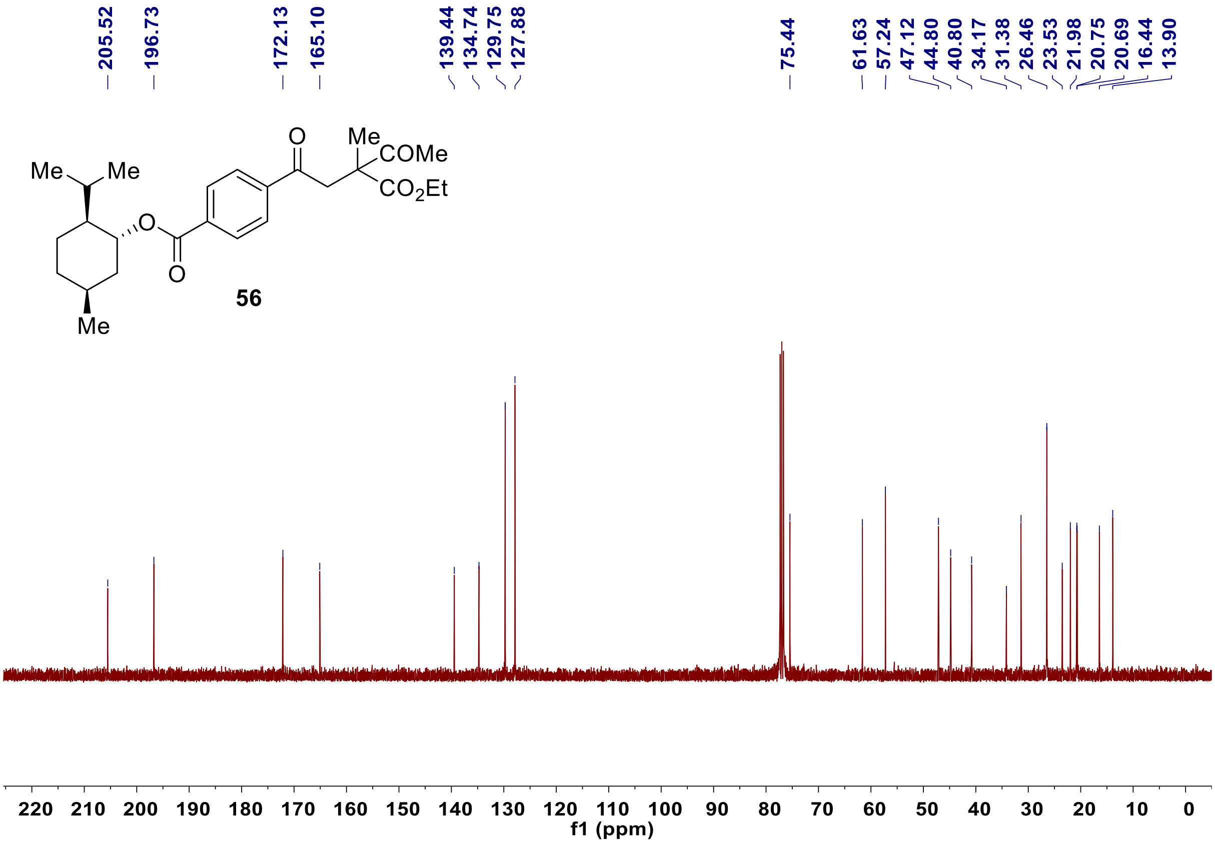

^{1}H and ^{13}C NMR spectra for compound 57

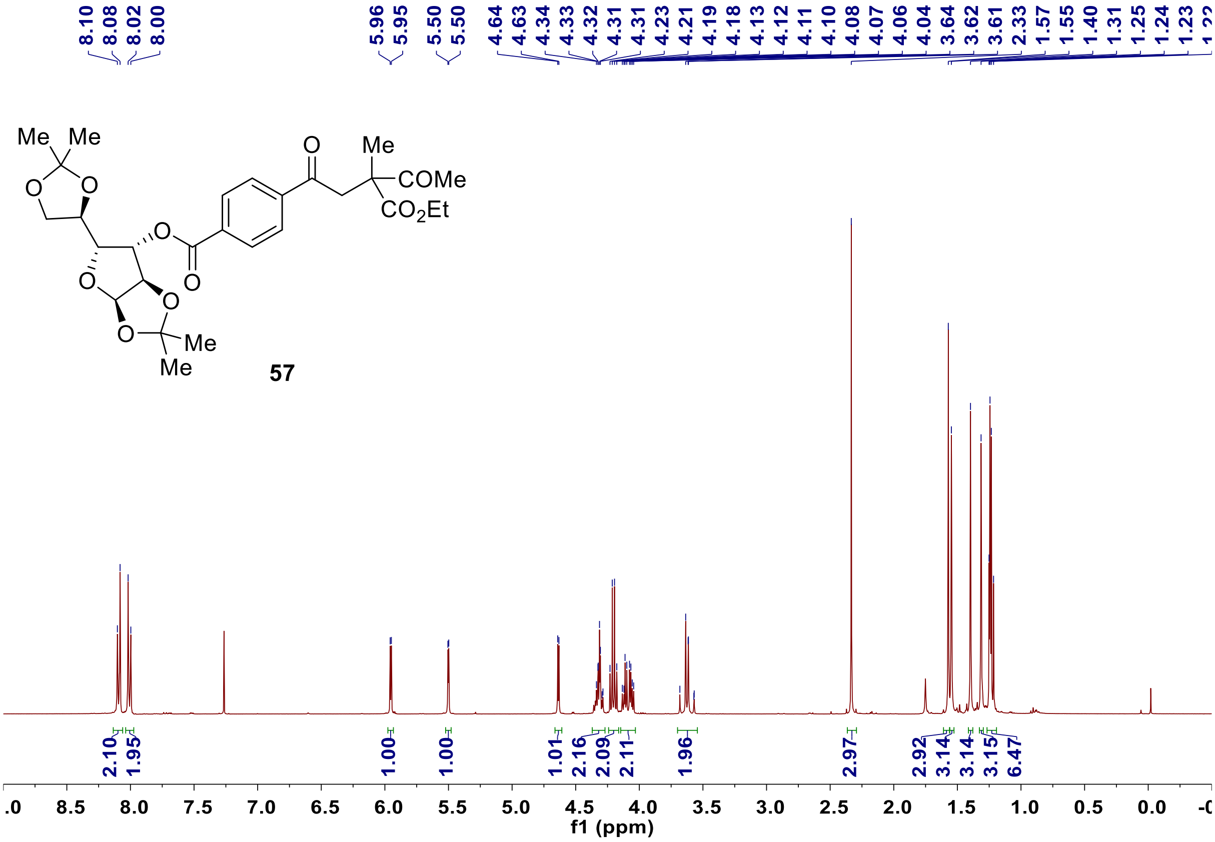

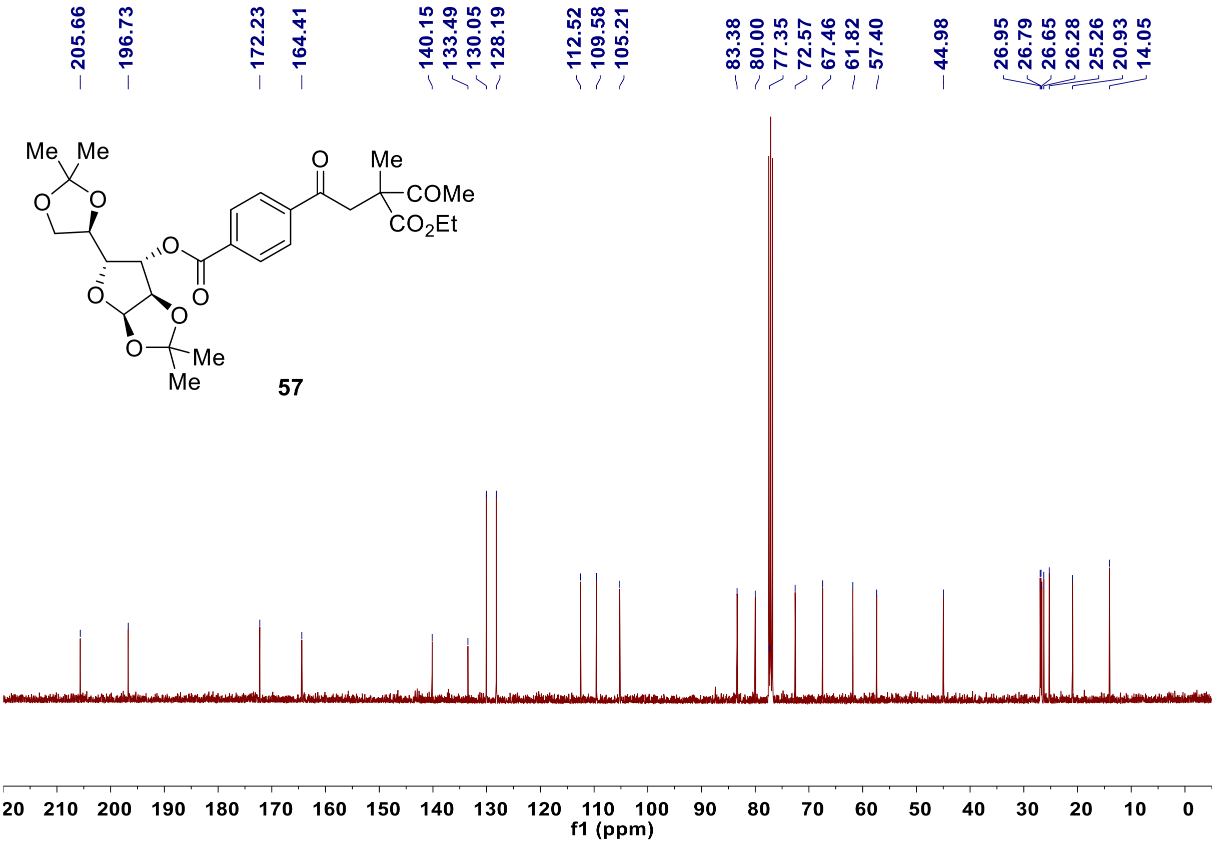

^{1}H and ^{13}C NMR spectra for compound 58

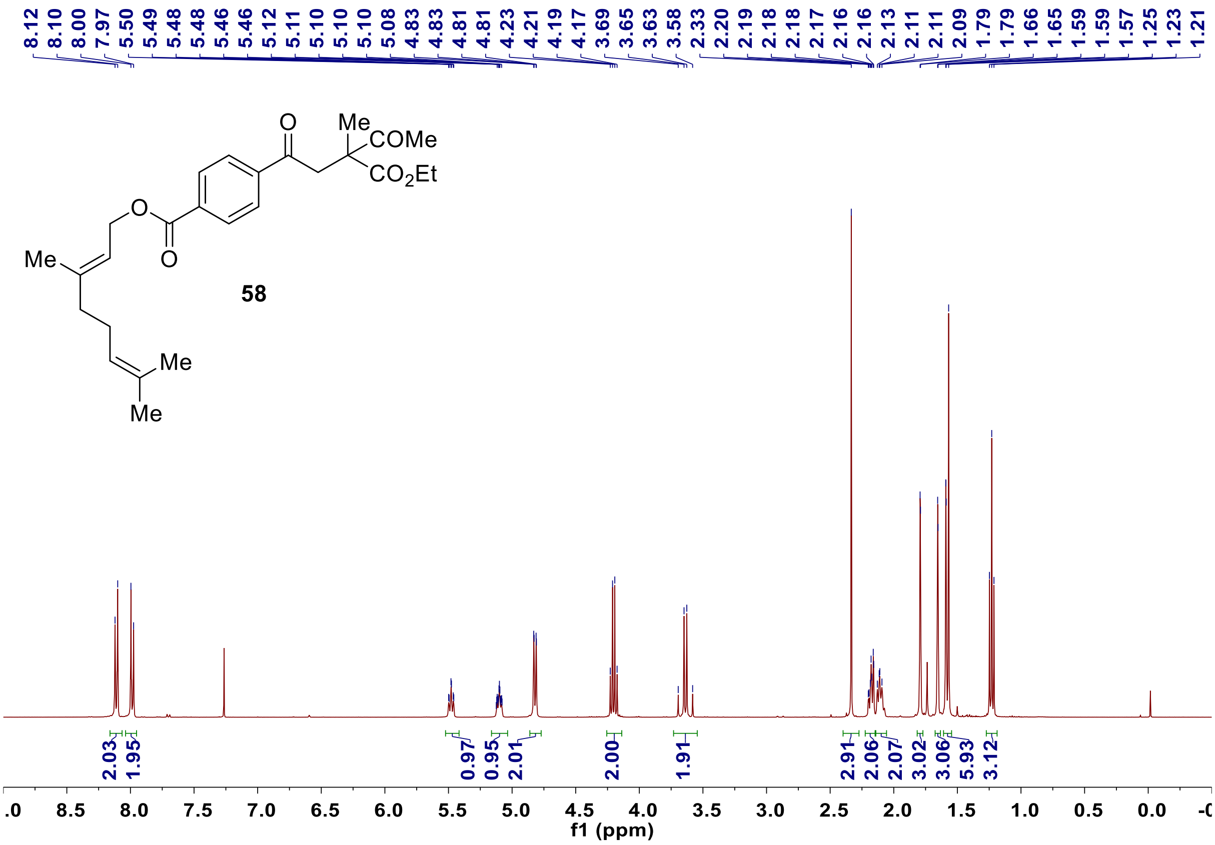

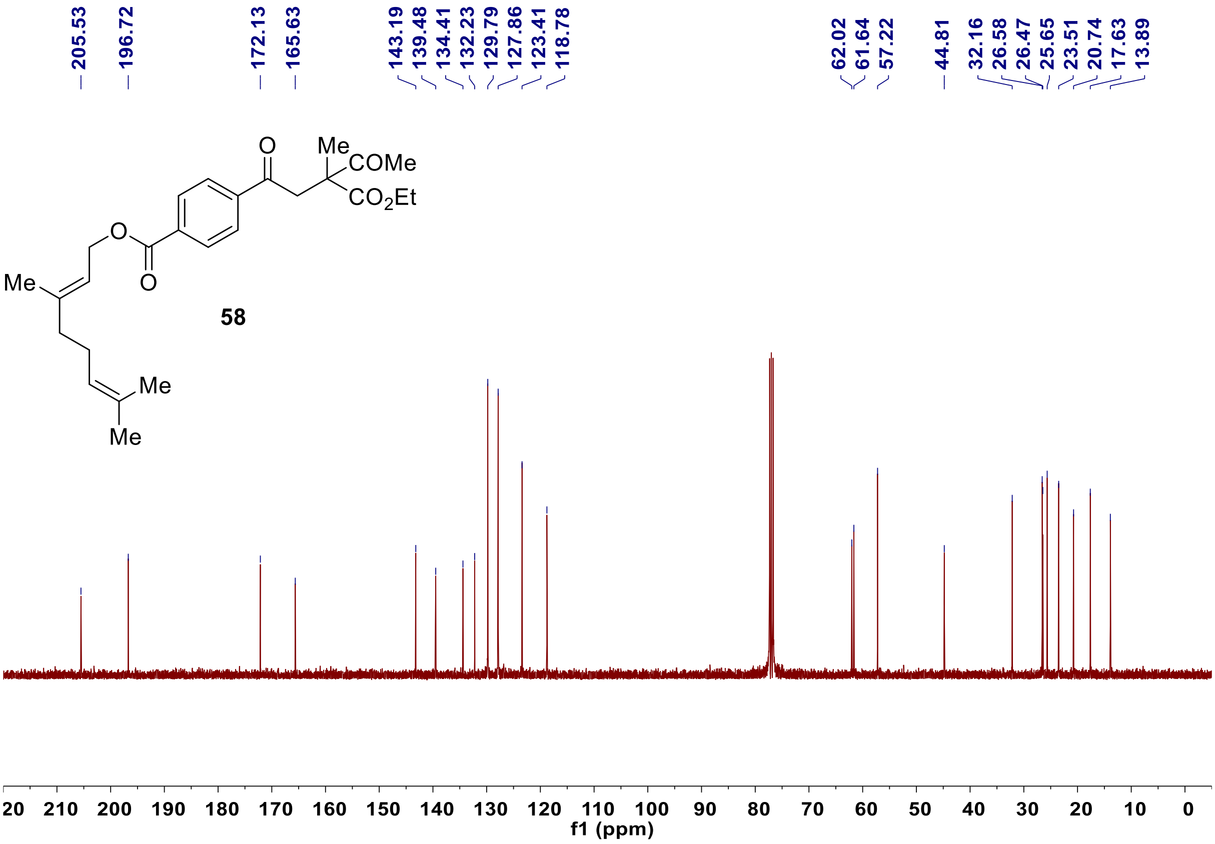

^{1}H and ^{13}C NMR spectra for compound 59

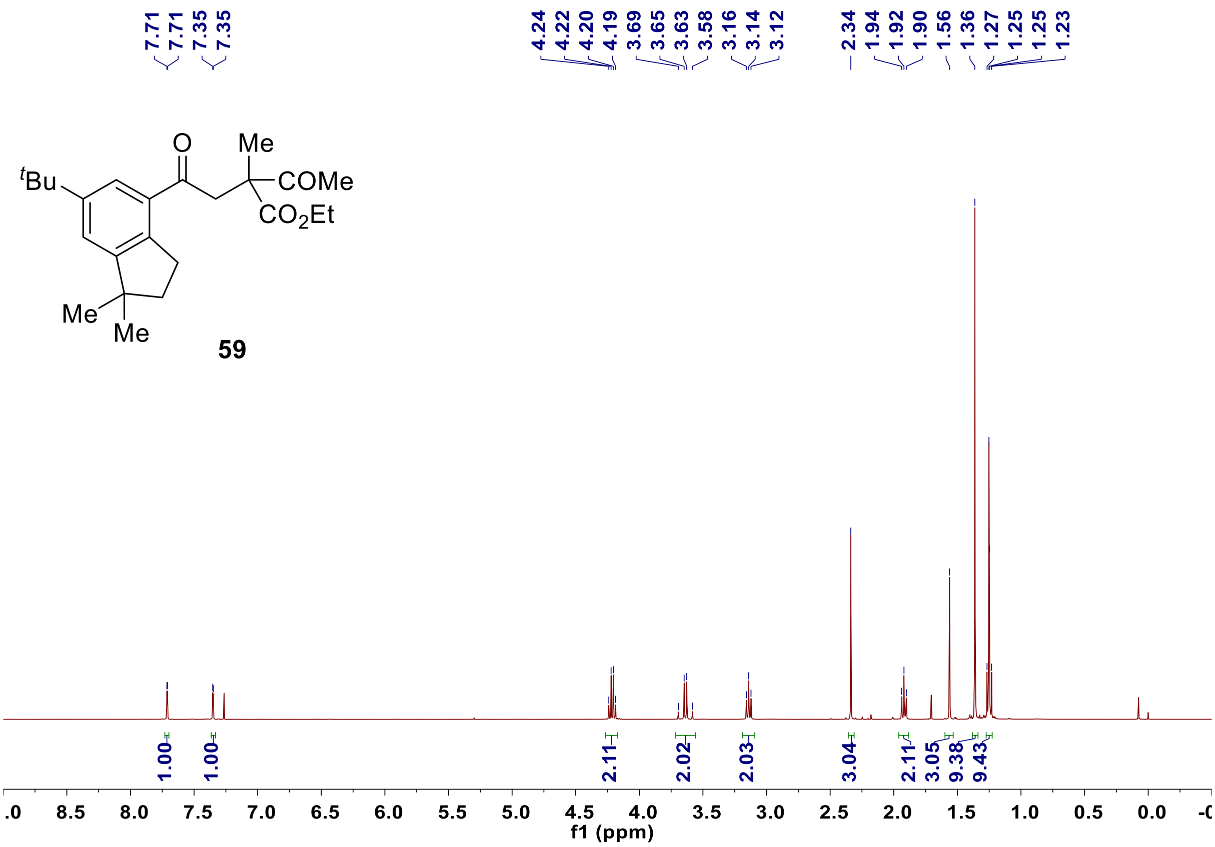

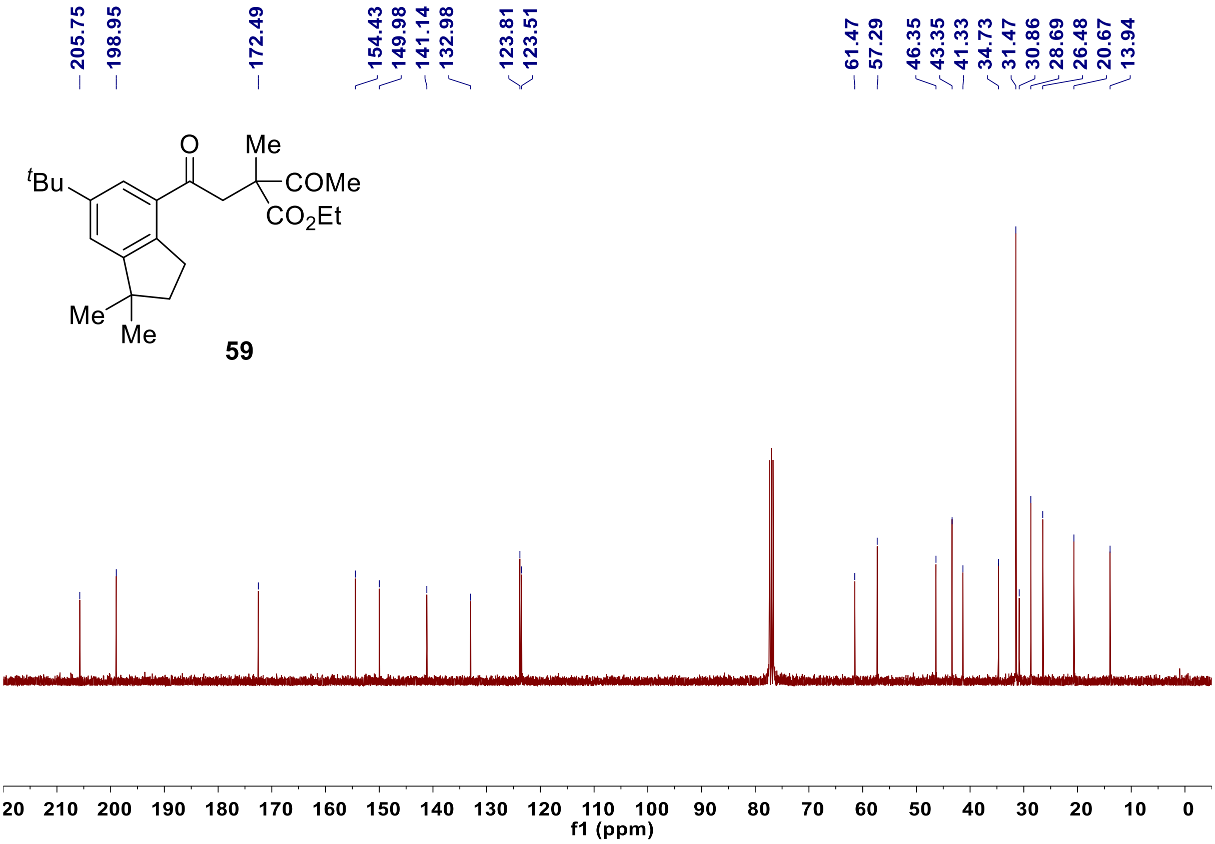

^{1}H and ^{13}C NMR spectra for compound 60

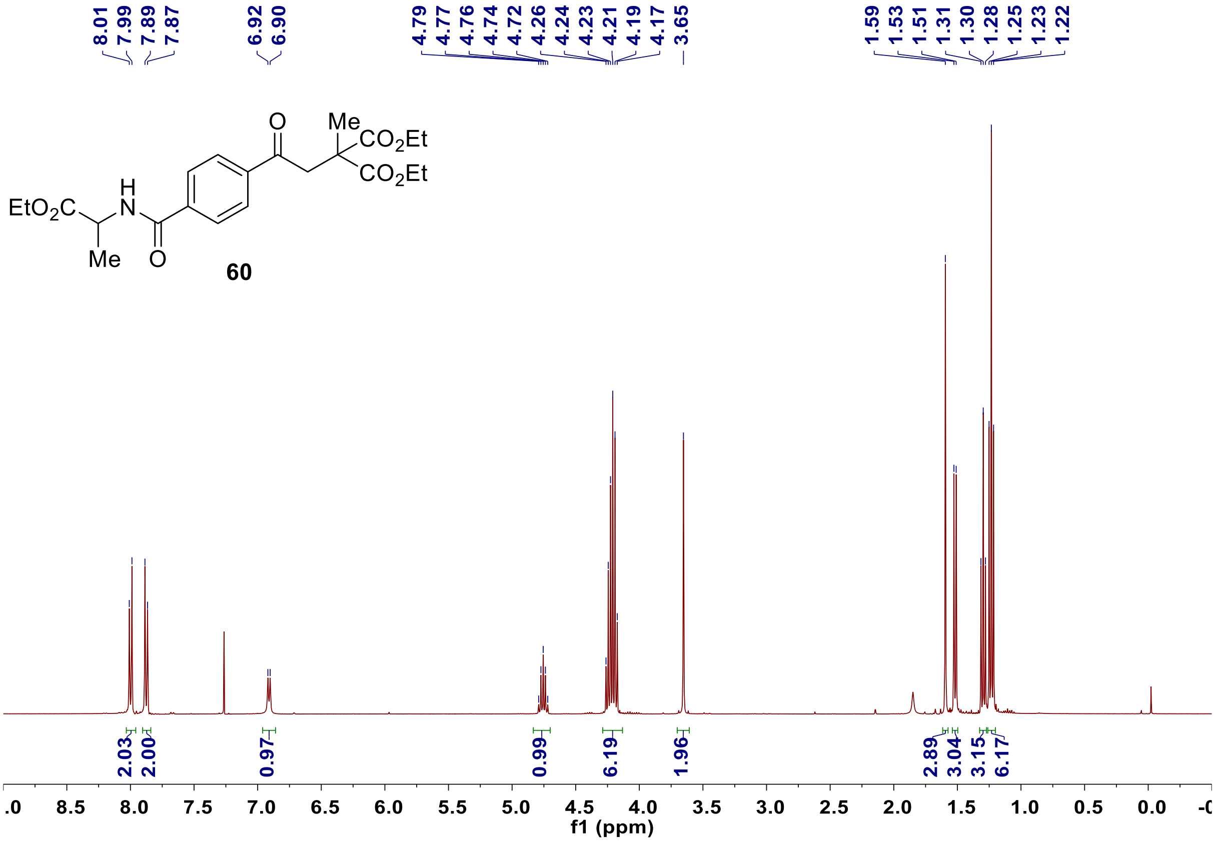

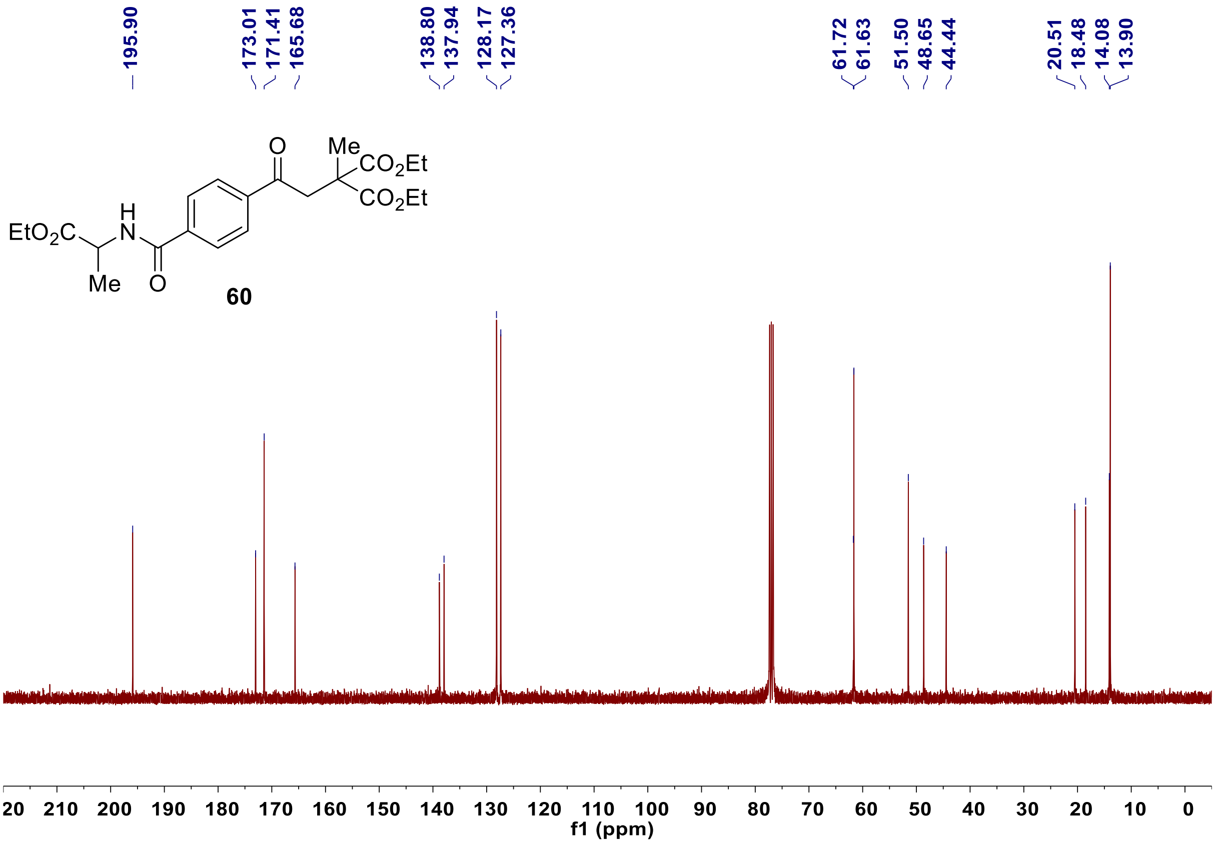

^{1}H and ^{13}C NMR spectra for compound 61

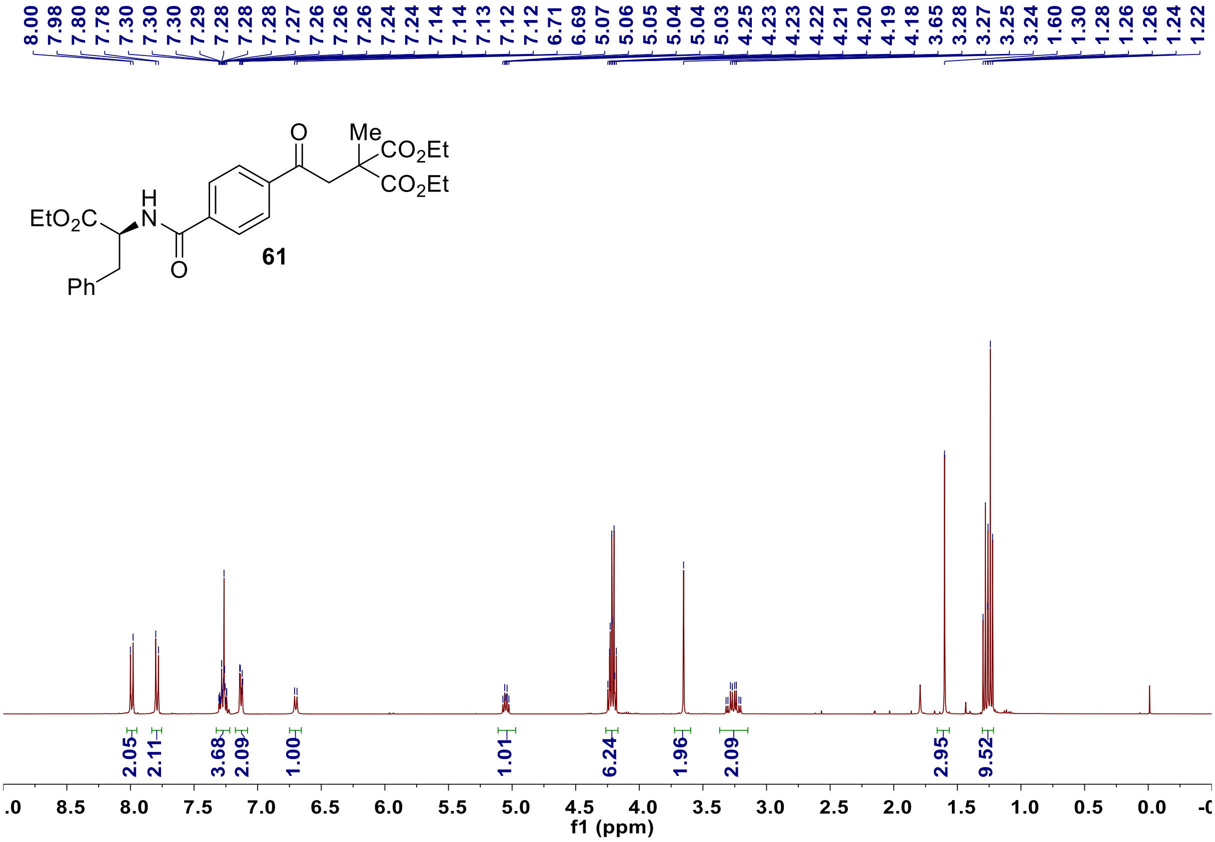

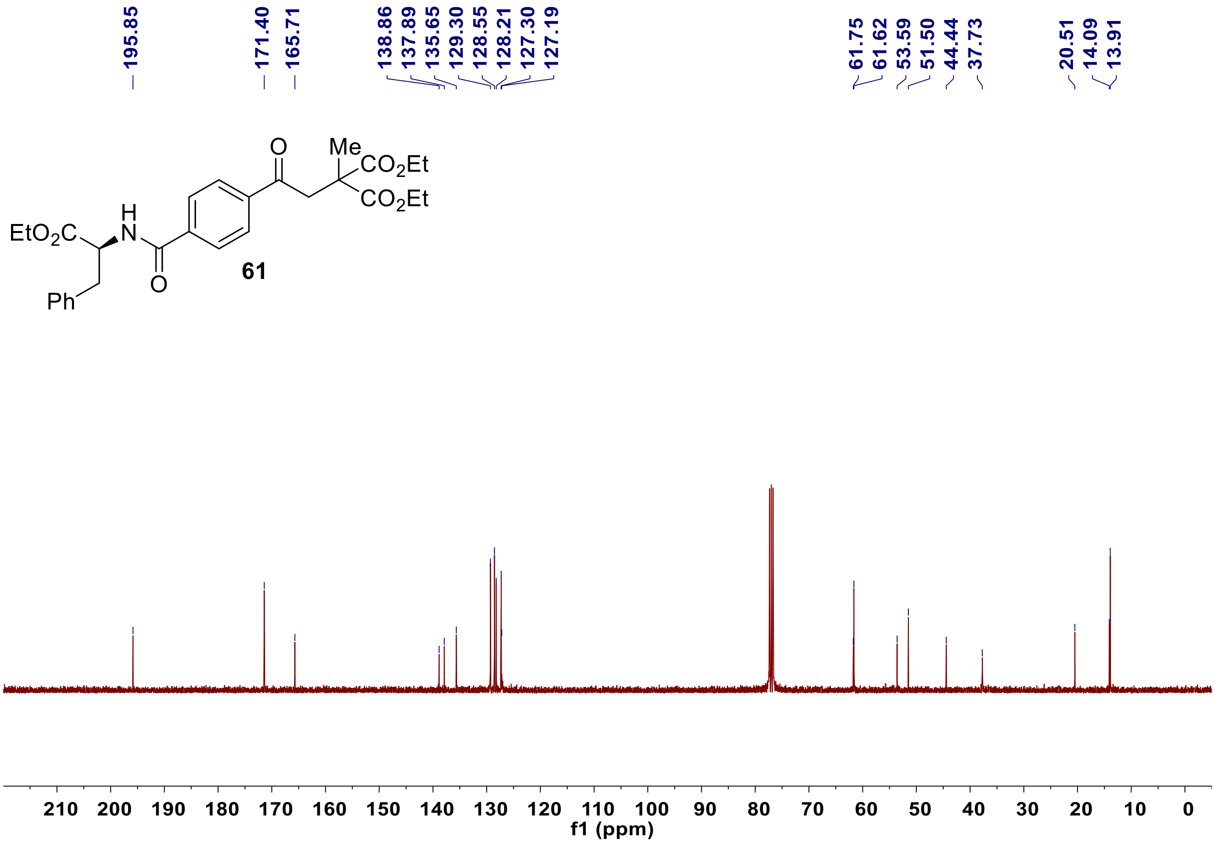

^{1}H and ^{13}C NMR spectra for compound 62

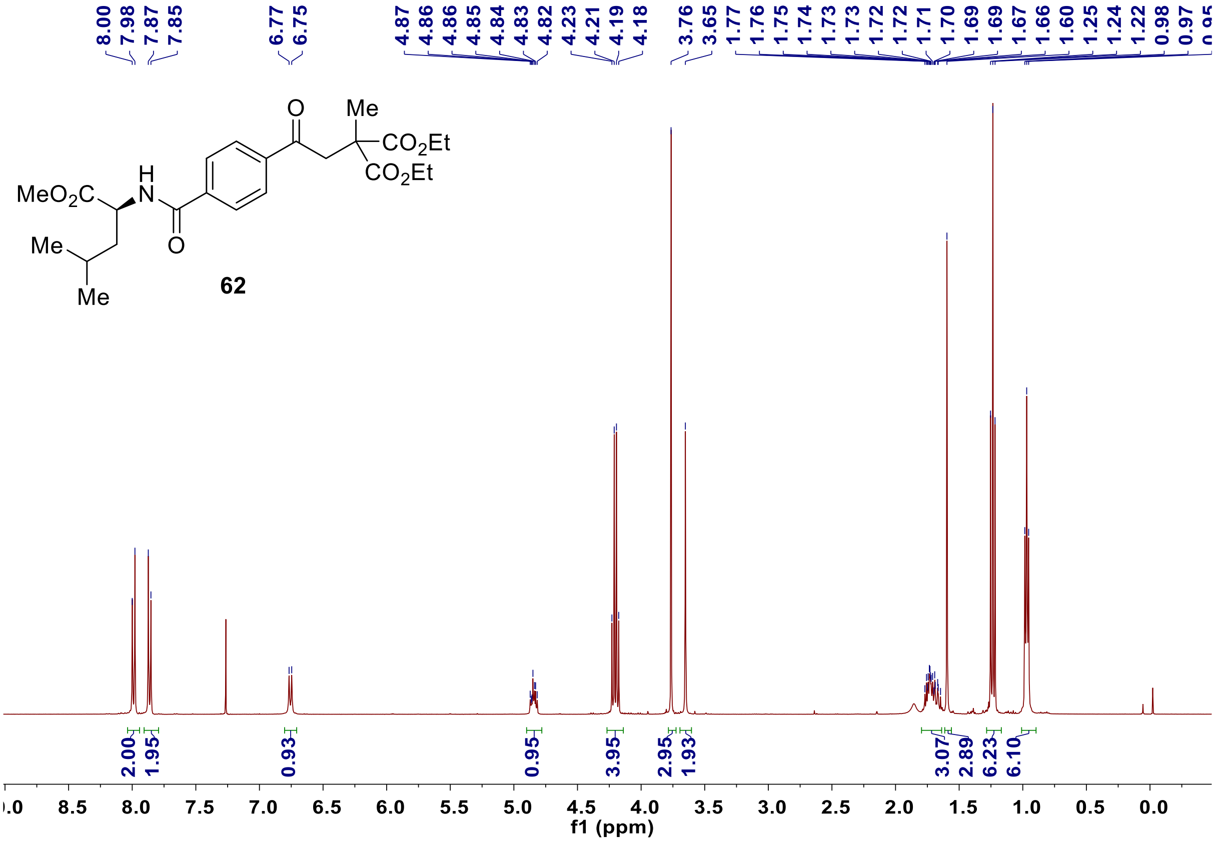

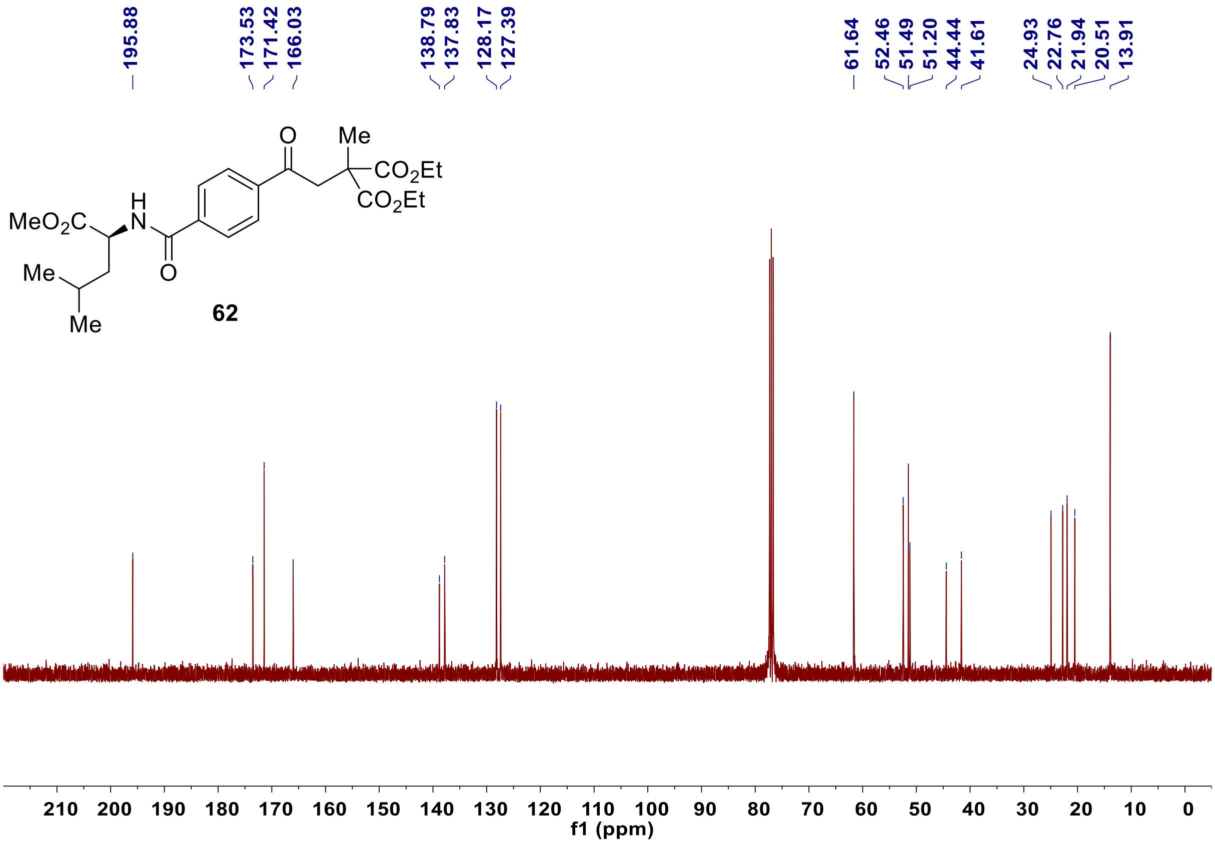

^{1}H and ^{13}C NMR spectra for compound 63

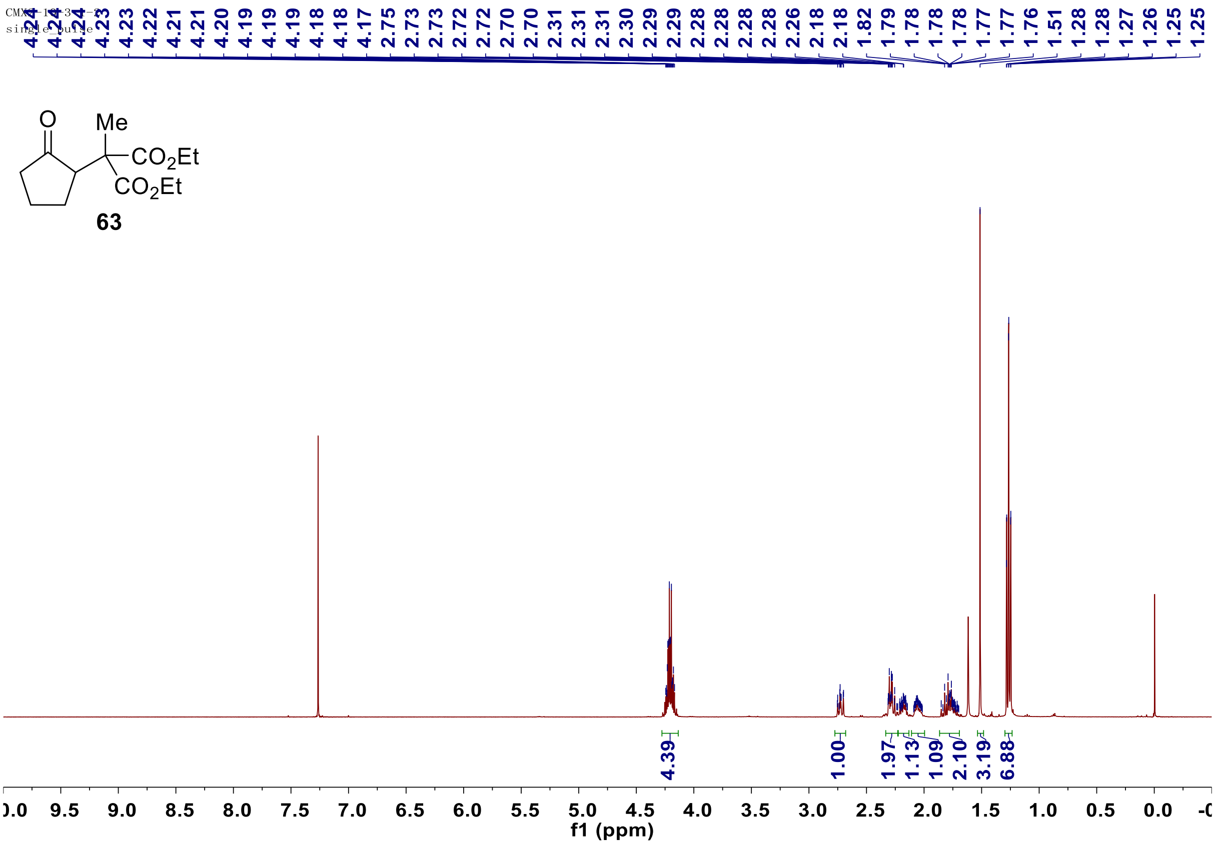

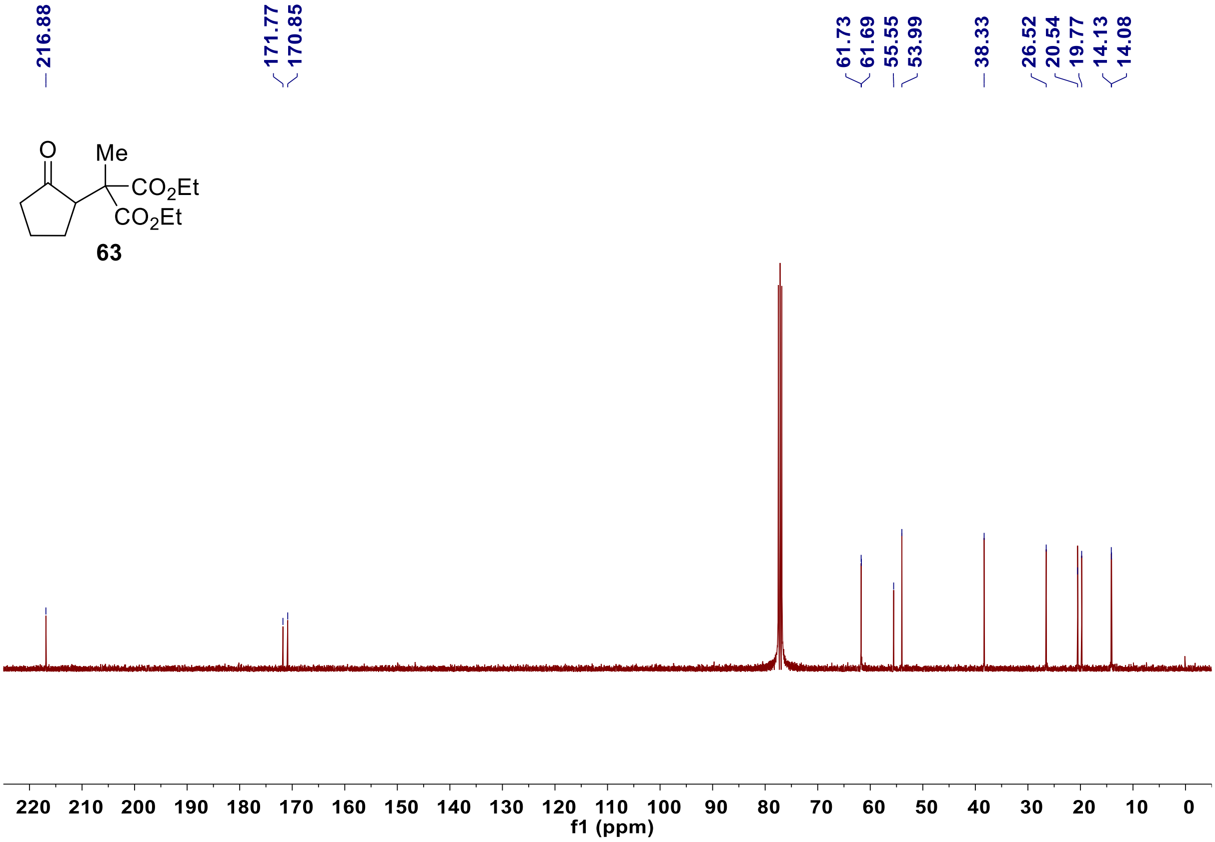

^{1}H and ^{13}C NMR spectra for compound 64

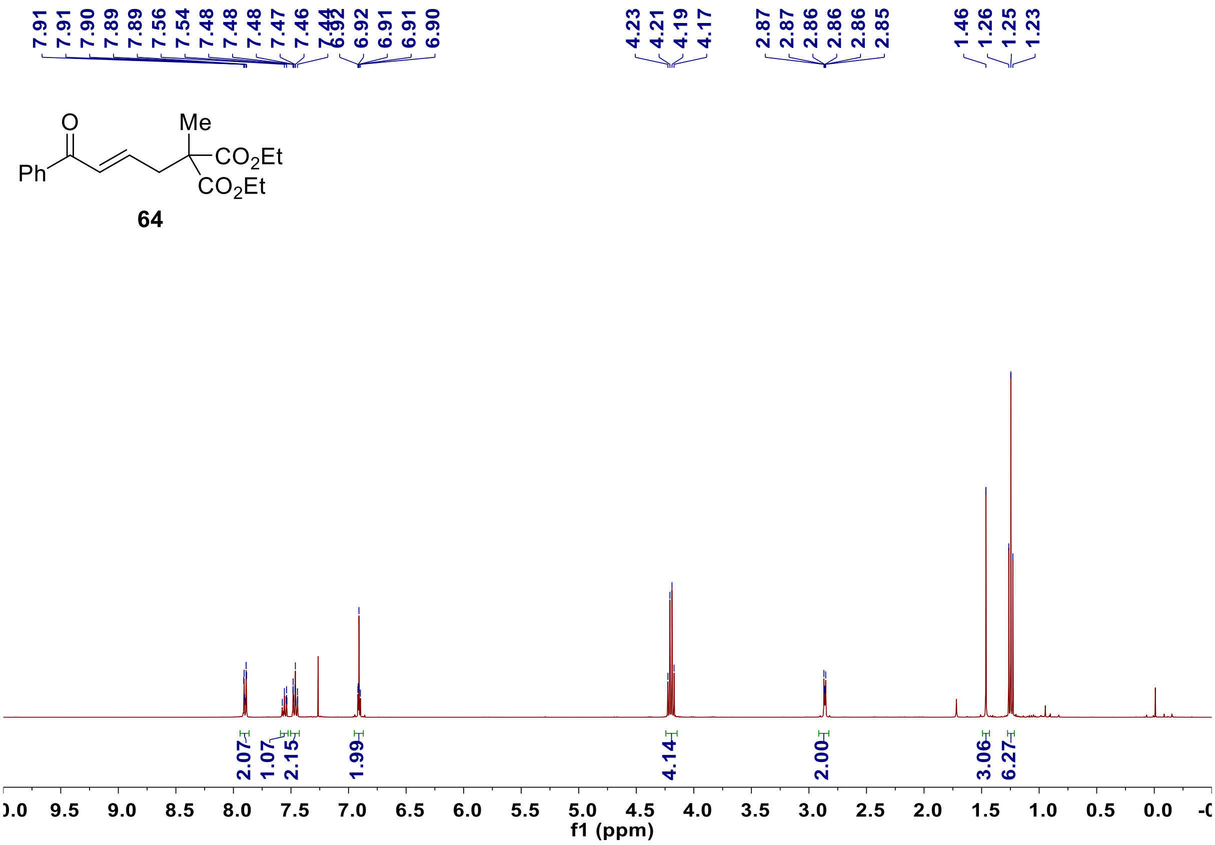

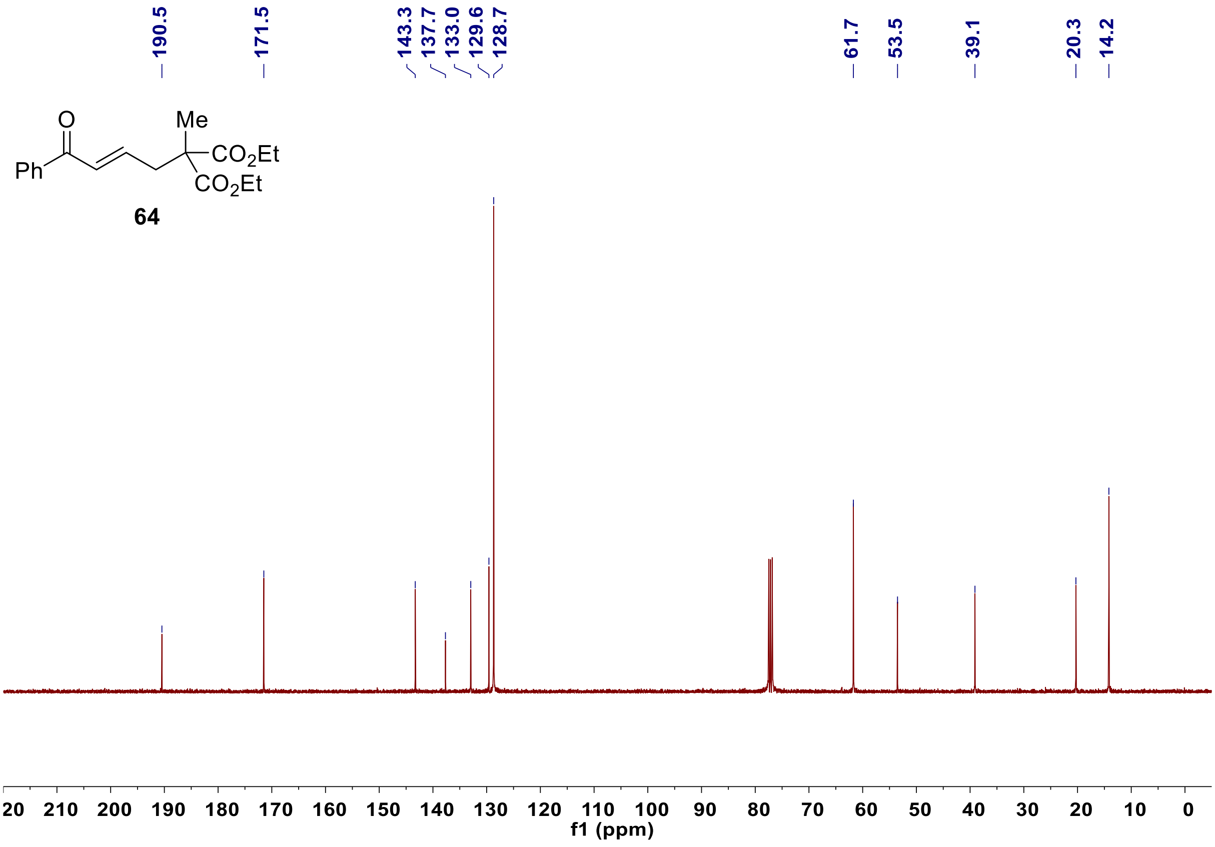

^{1}H and ^{13}C NMR spectra for compound 65

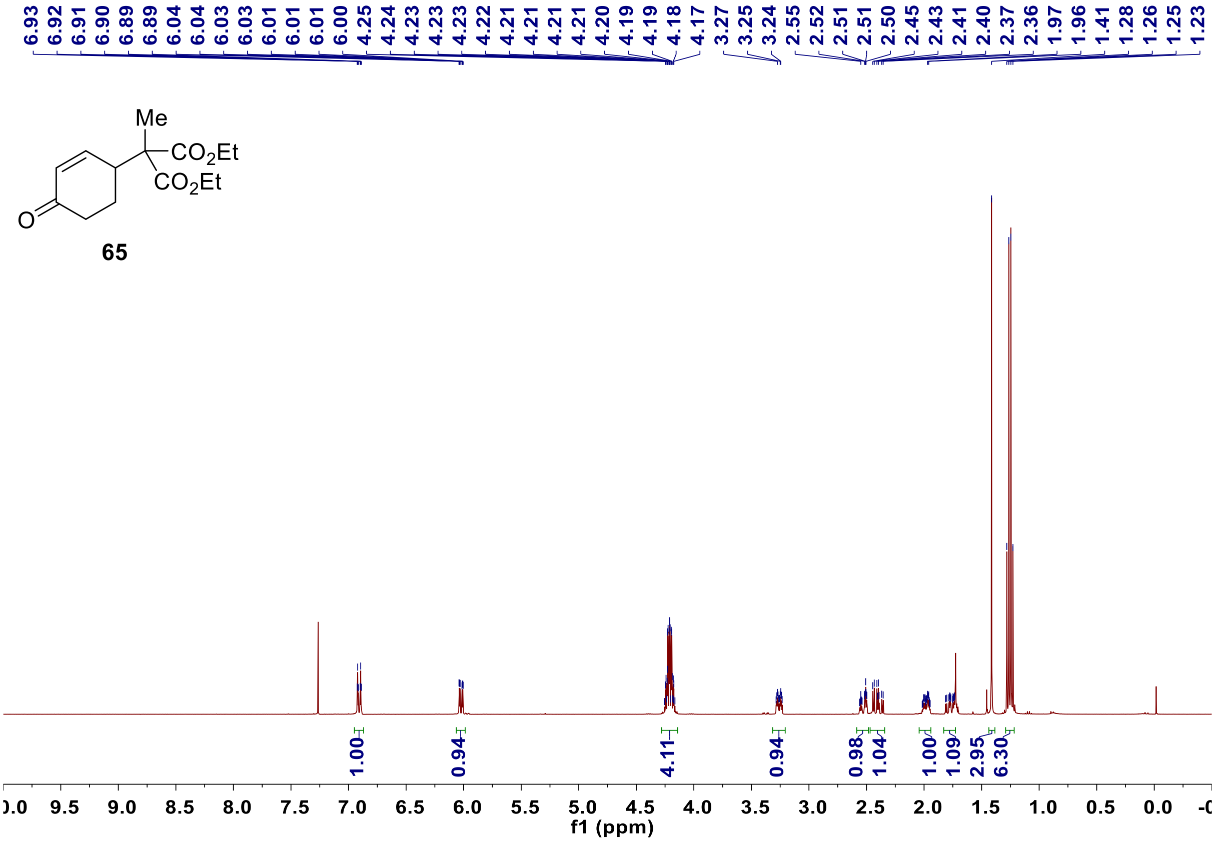

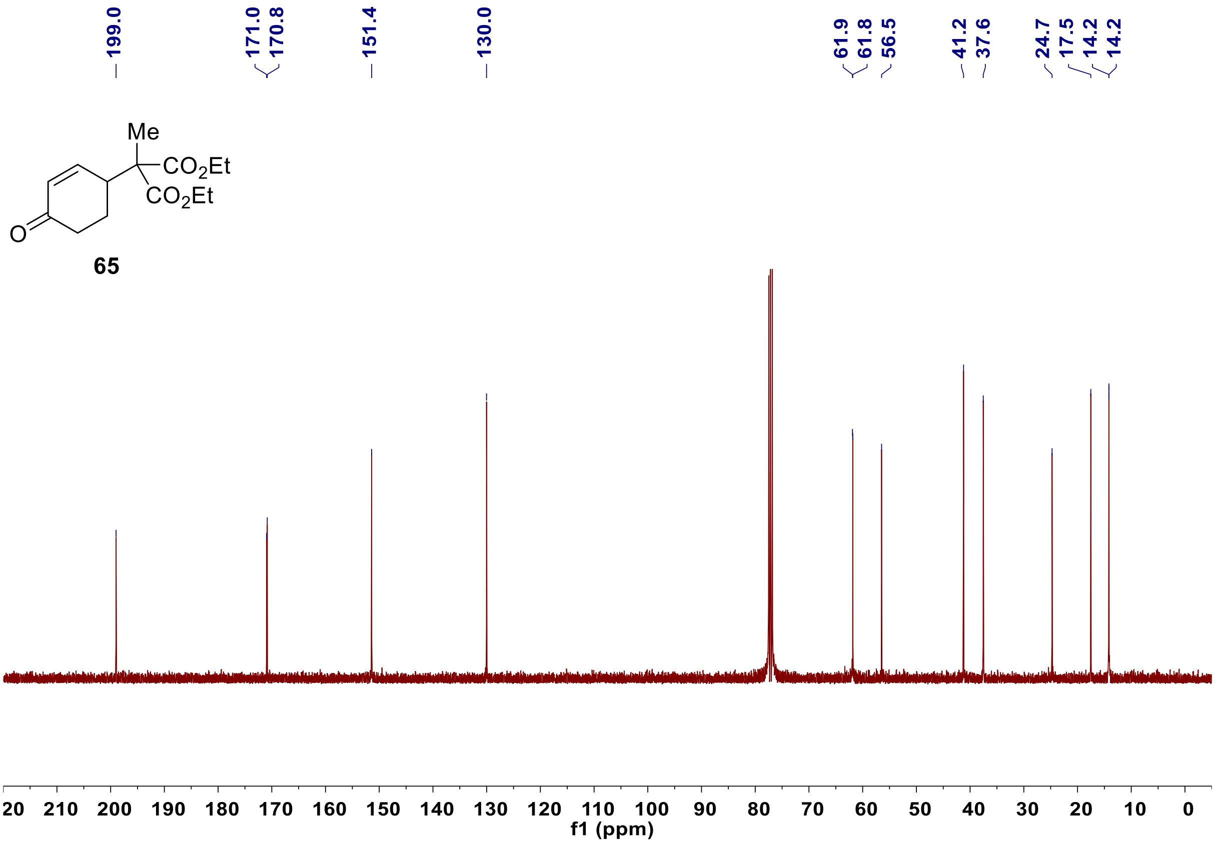

^{1}H, ^{13}C, NOESY and HMBC NMR spectra for compound 66

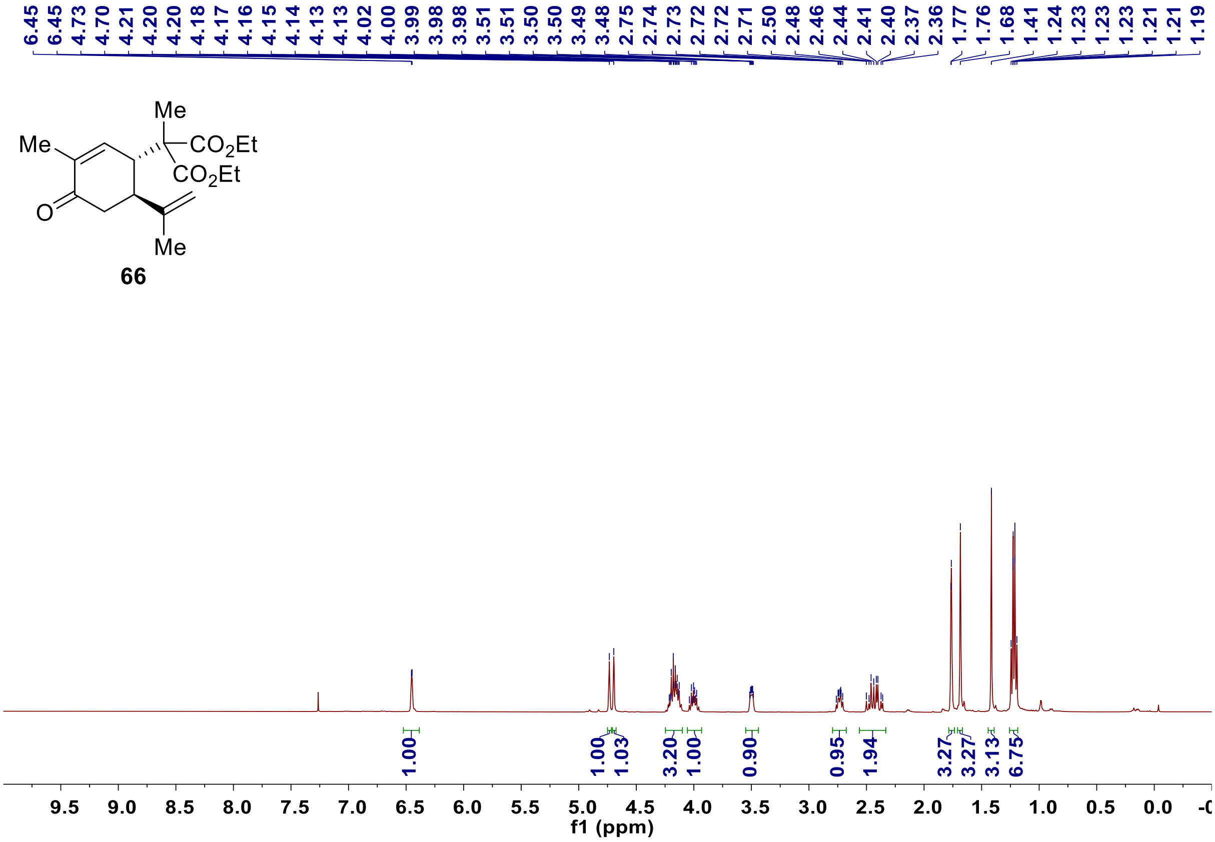

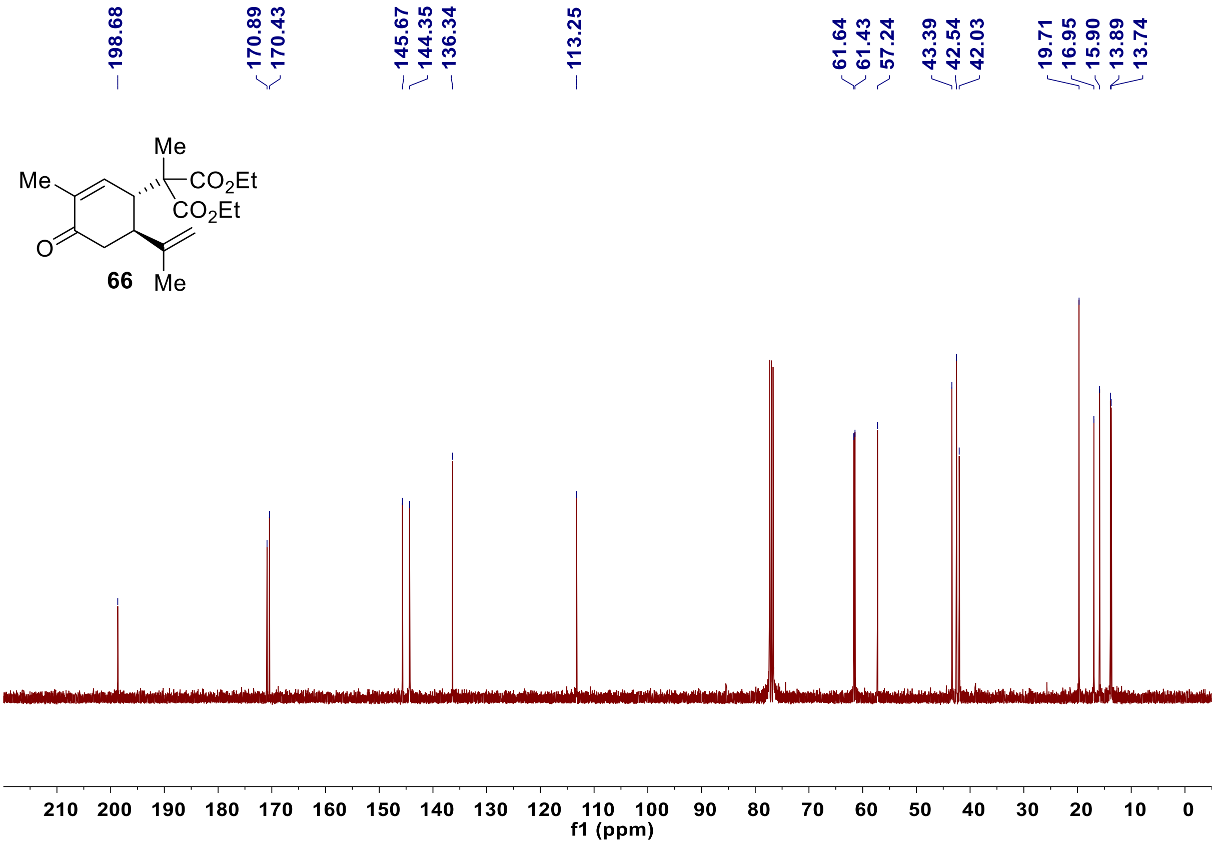

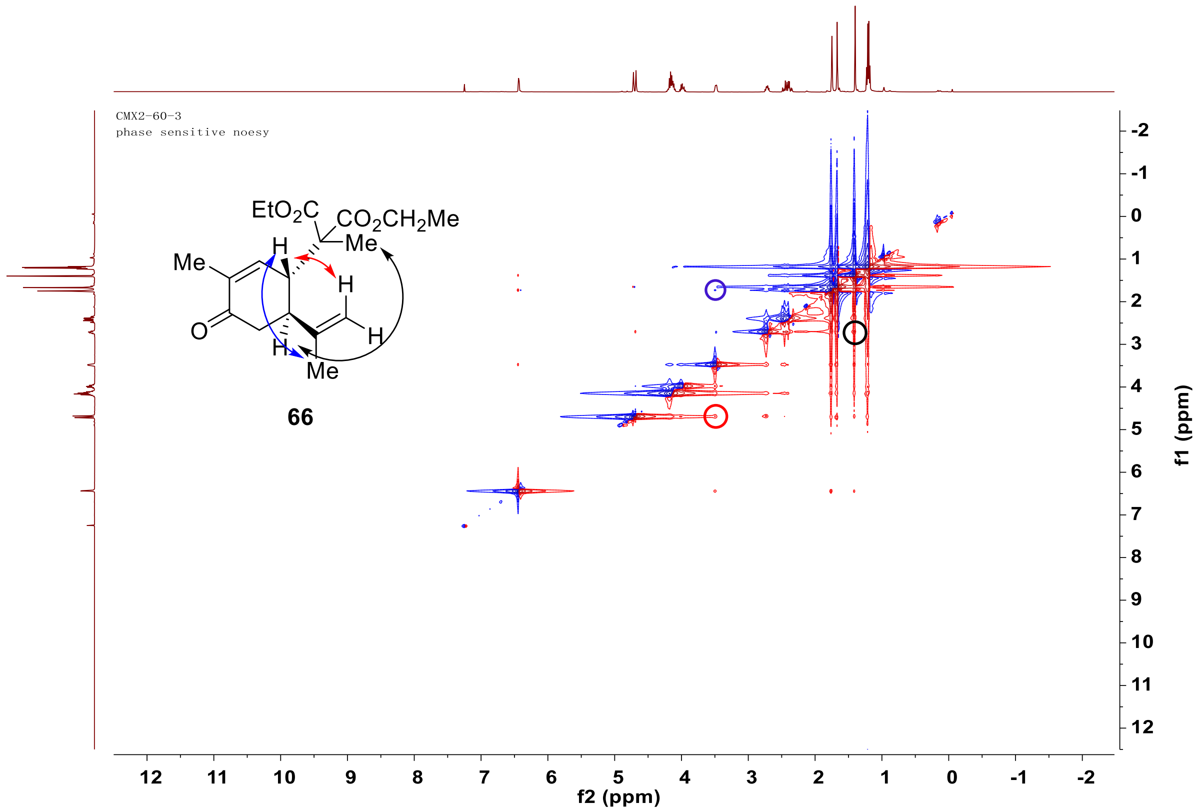
CMX2-60-3
phase sensitive noesy
EtO2C
CO2CH2Me
Me
H
H
H
Me
O
H
Me
66
f2 (ppm)
f1 (ppm)

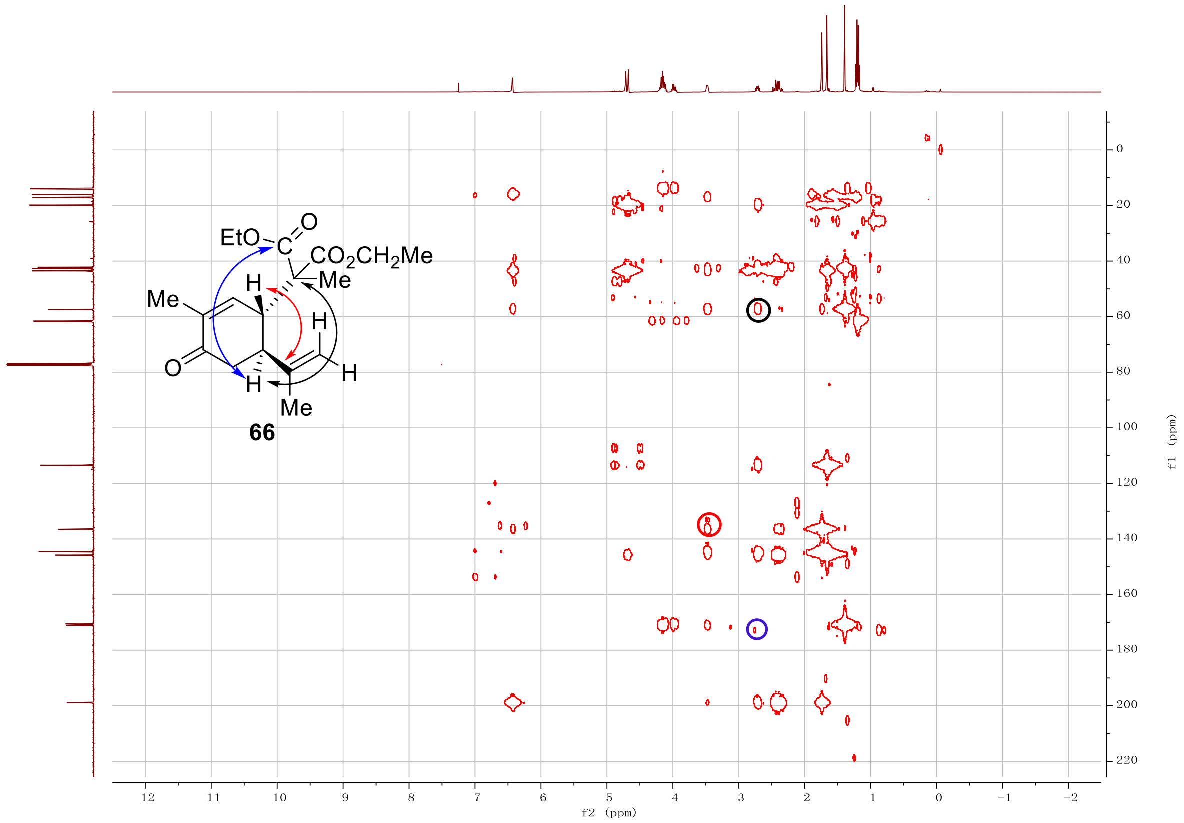
EtO
C
O
CO2CH2Me
Me
H
Me
H
O
H
H
Me
66
f2 (ppm)
f1 (ppm)

^{1}H and ^{13}C NMR spectra for compound 67

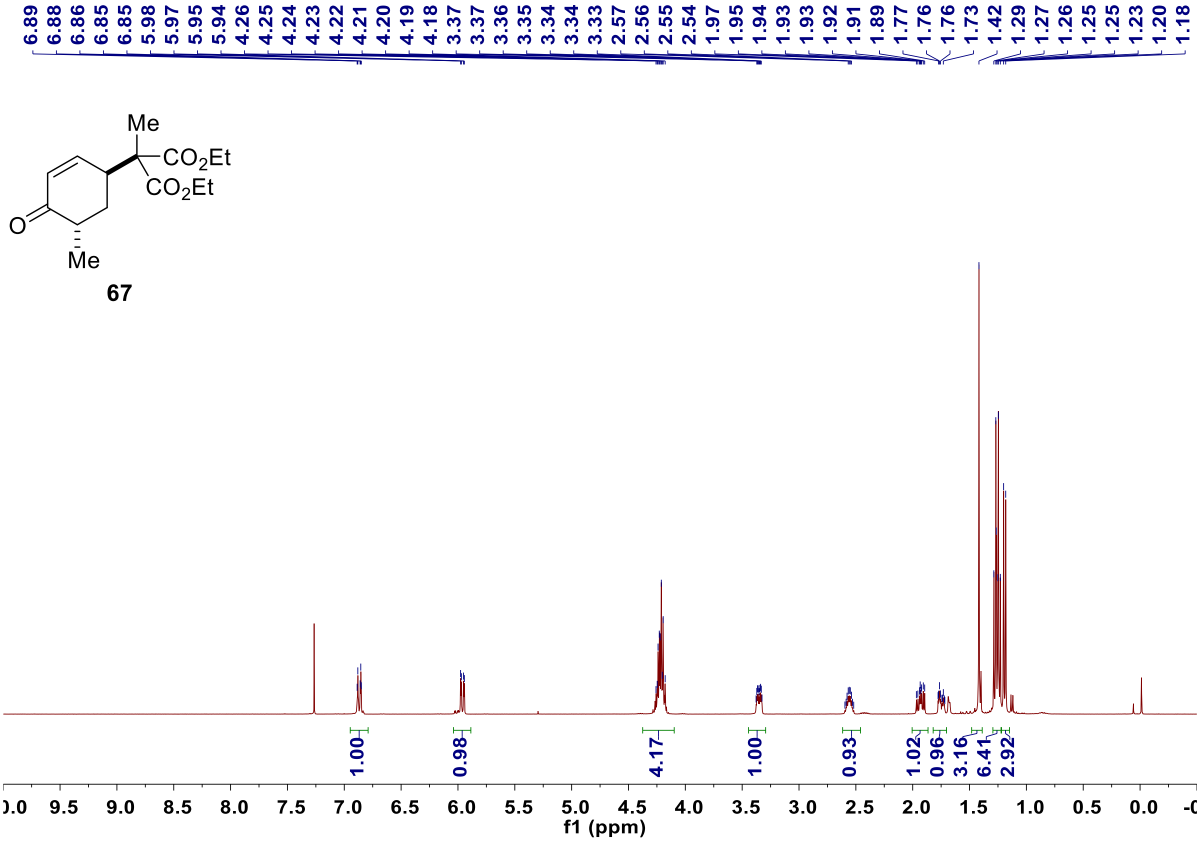

67

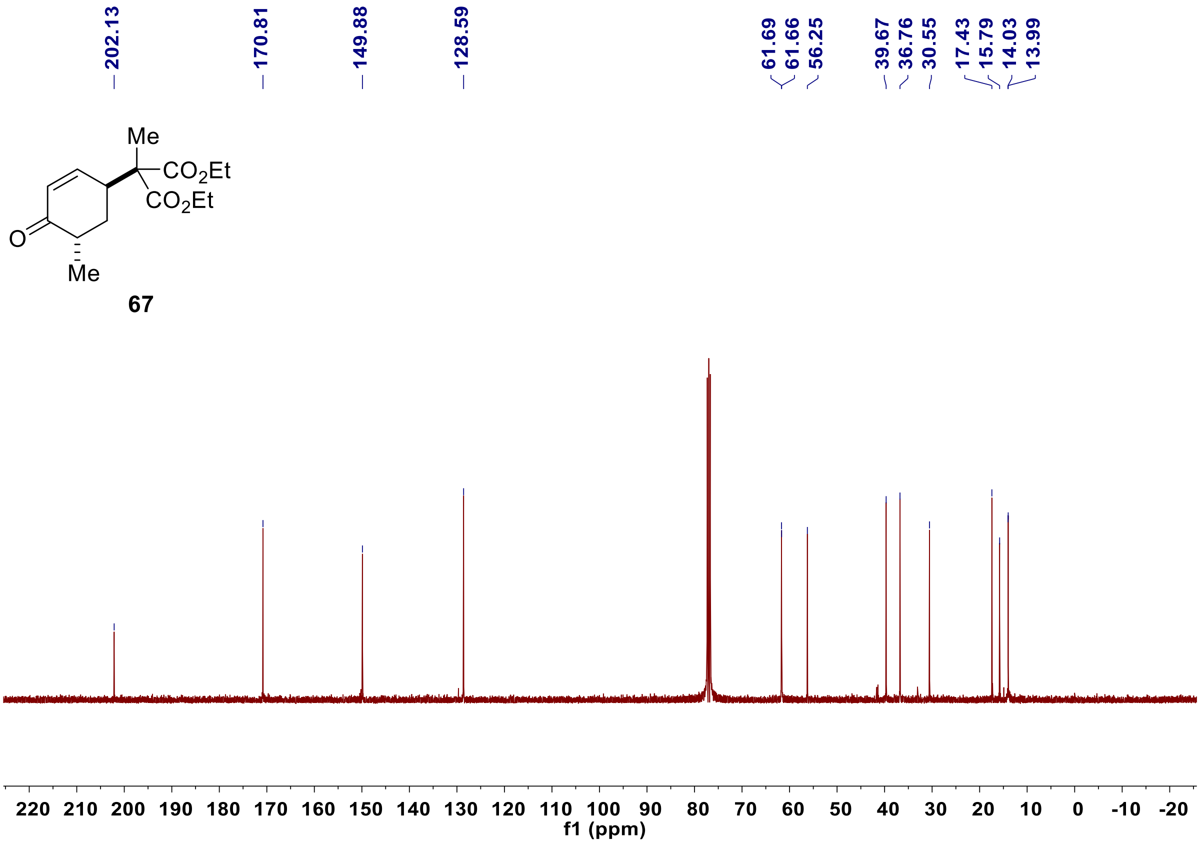

67

^{1}H and ^{13}C NMR spectra for compound 68

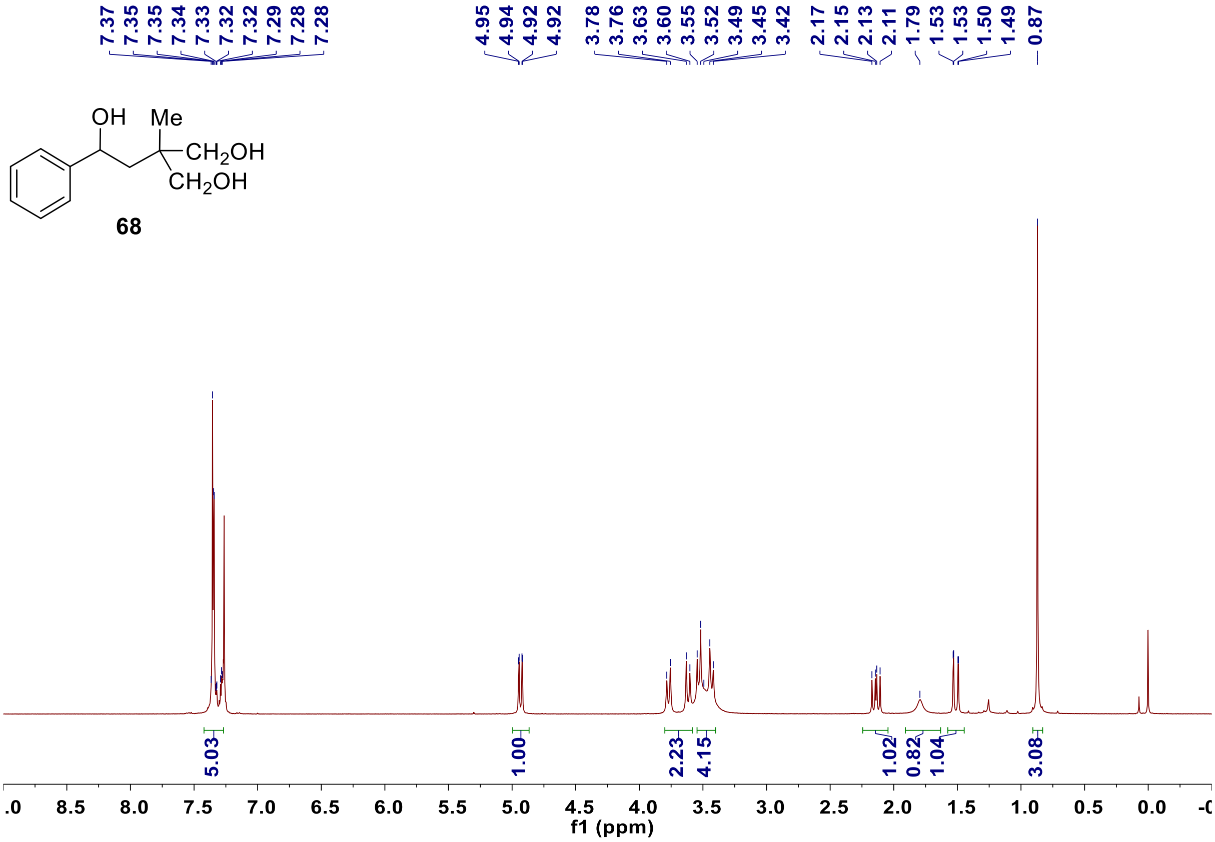

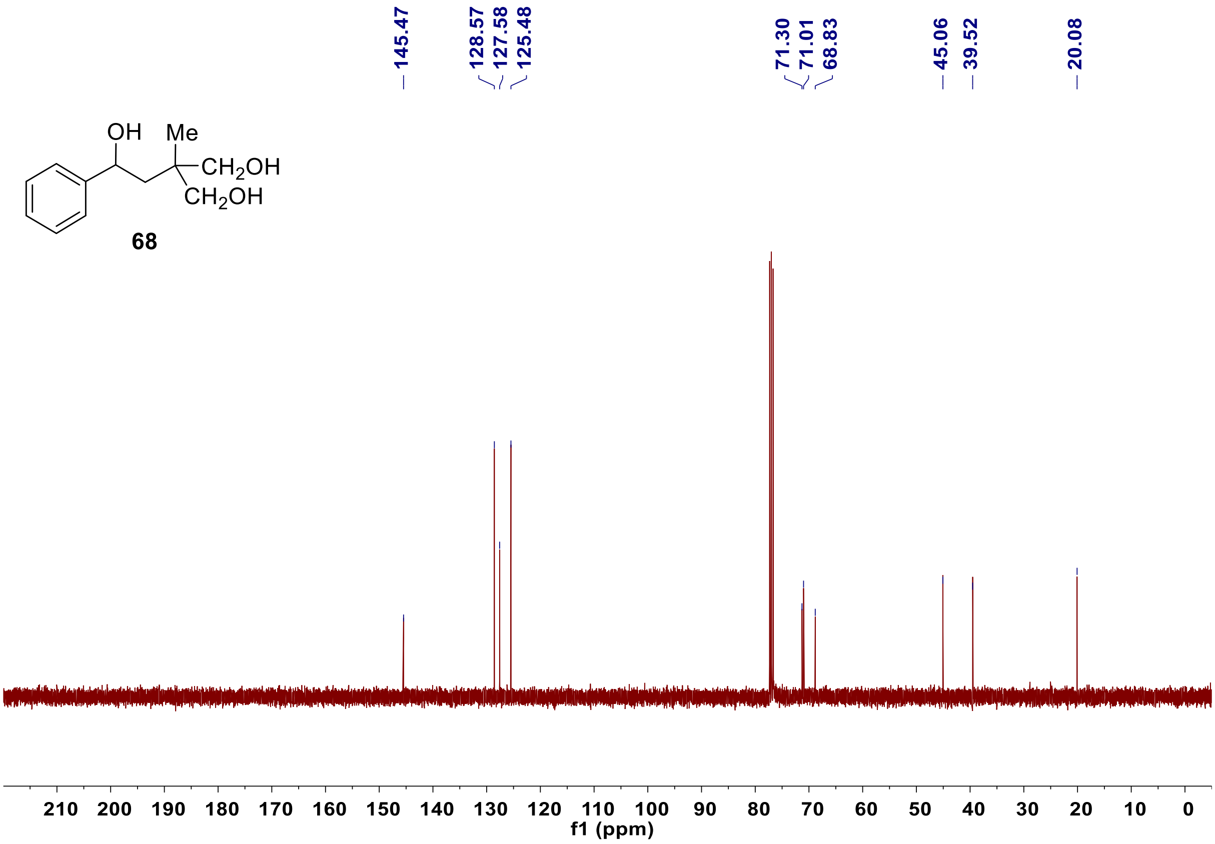

^{1}H and ^{13}C NMR spectra for compound 69

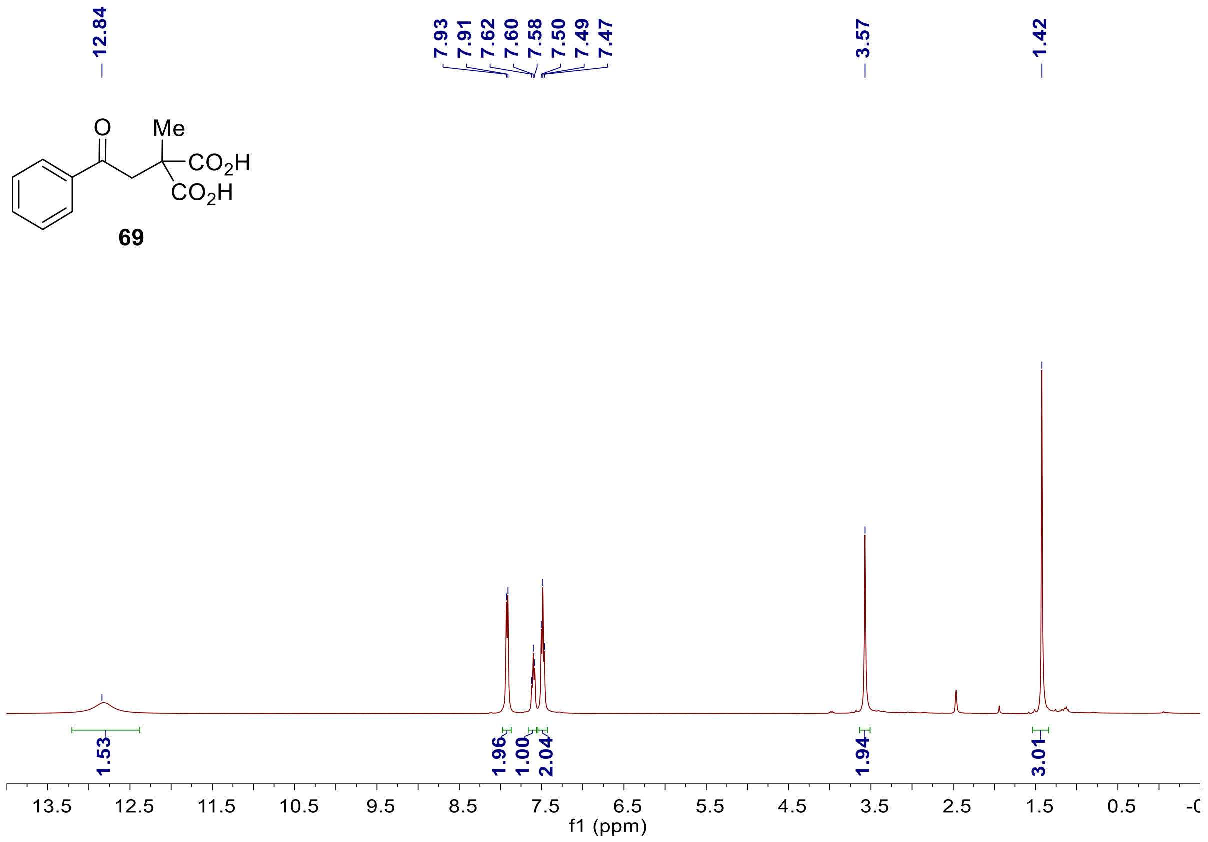

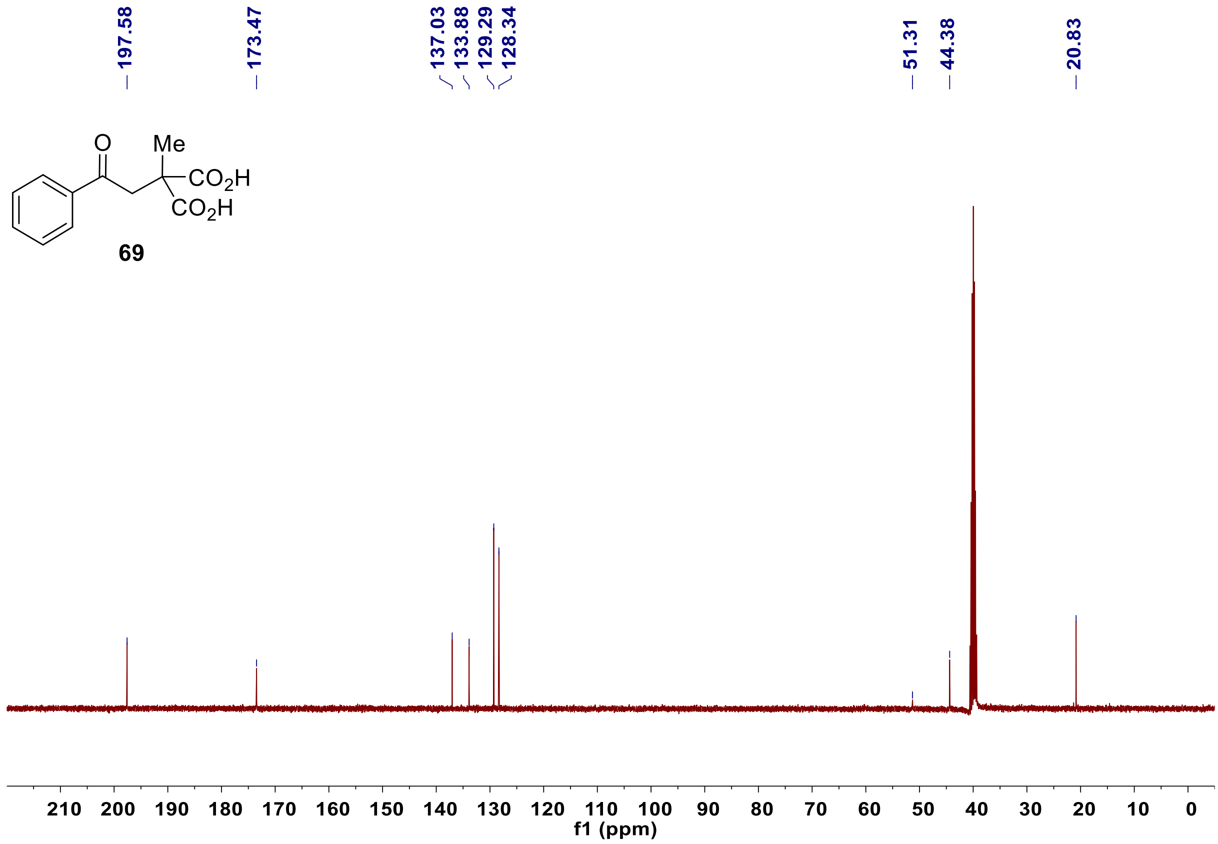

^{1}H and ^{13}C NMR spectra for compound 70

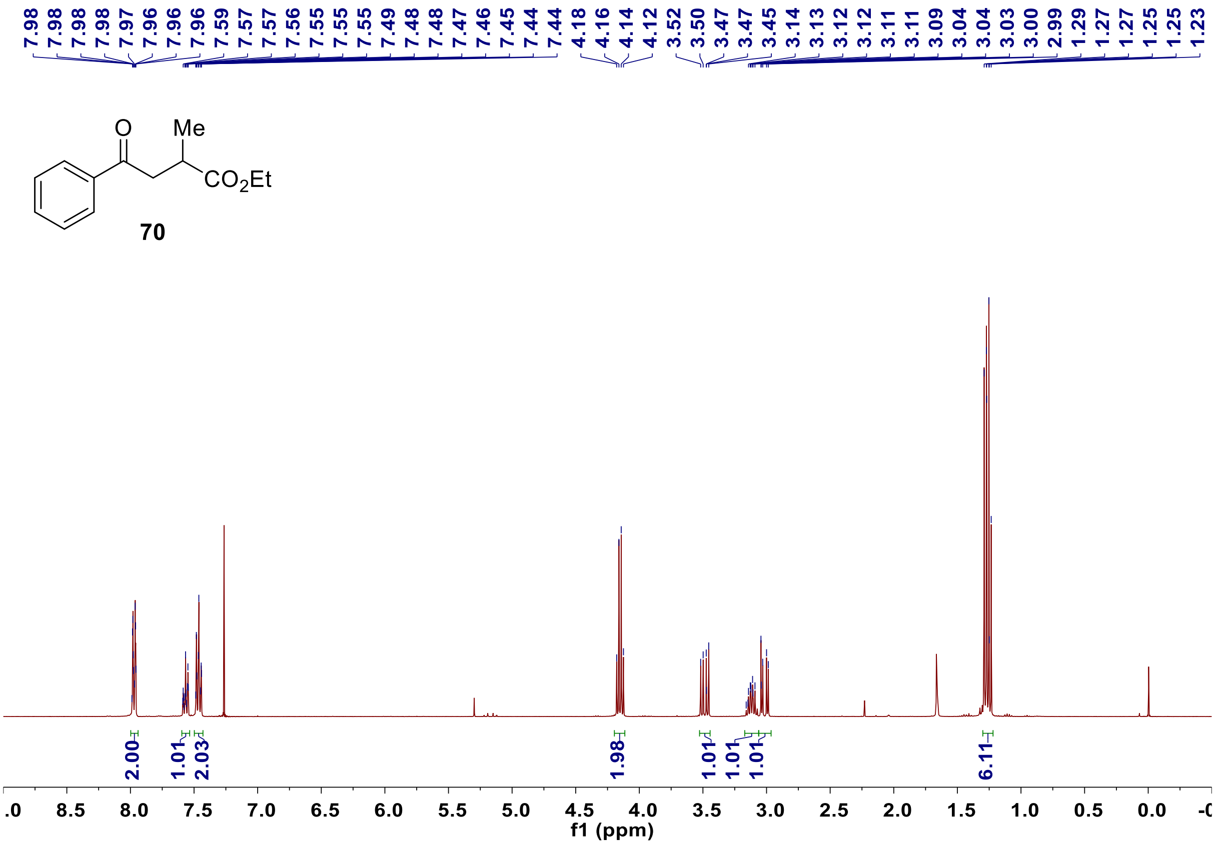

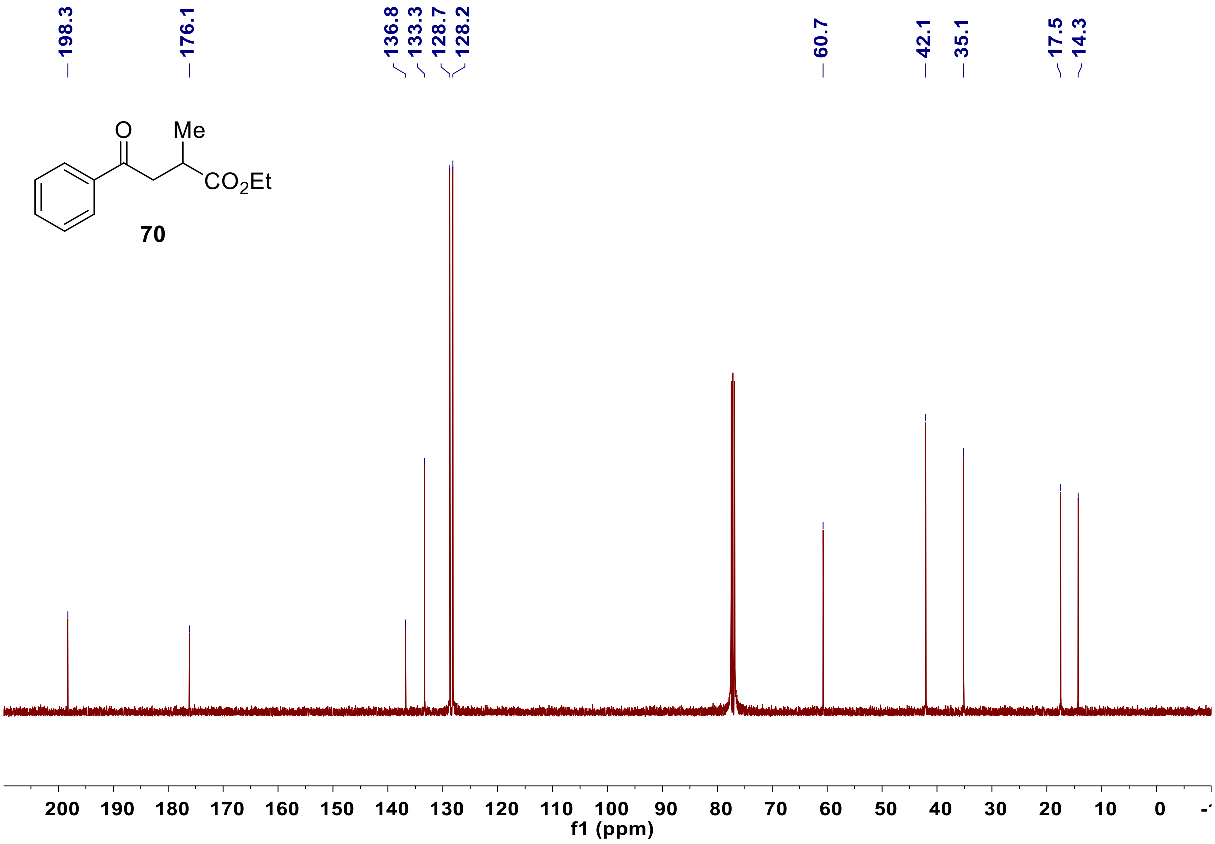

^{1}H and ^{13}C NMR spectra for compound 71

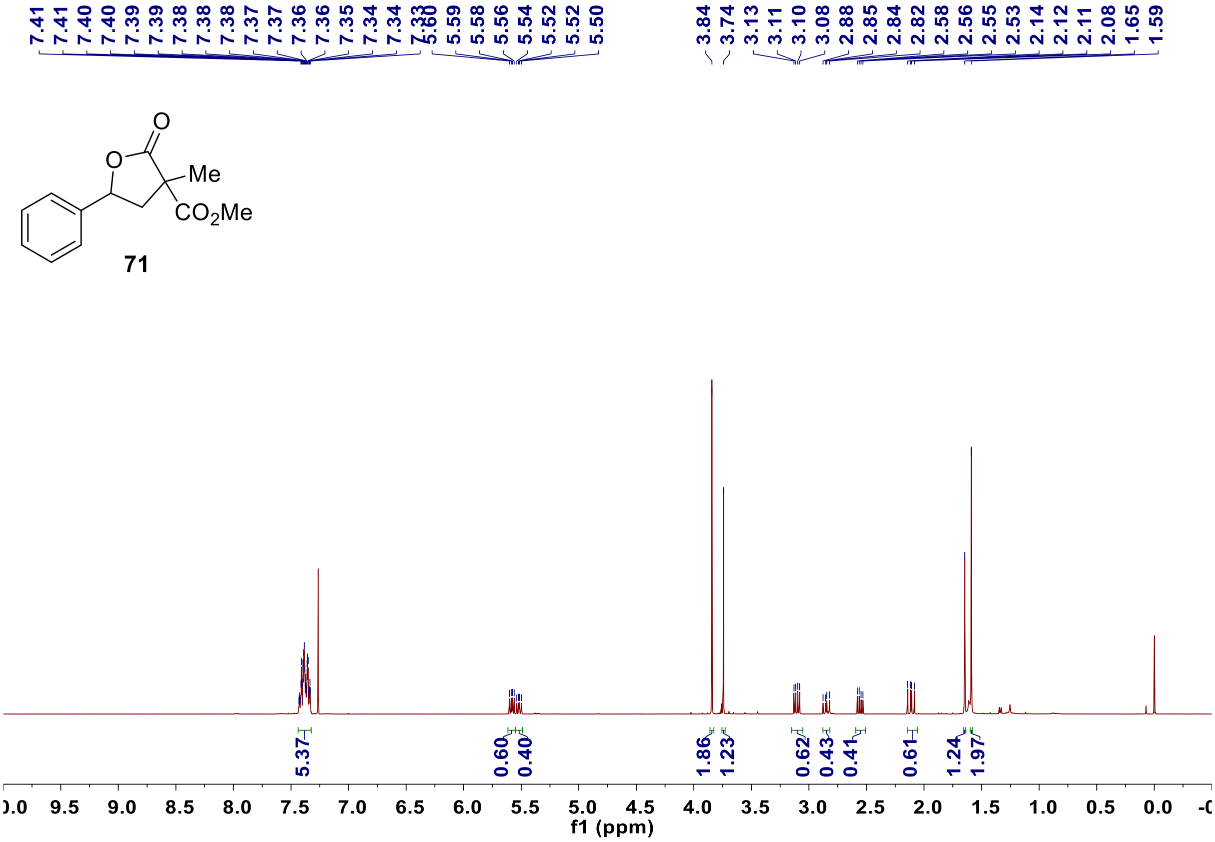

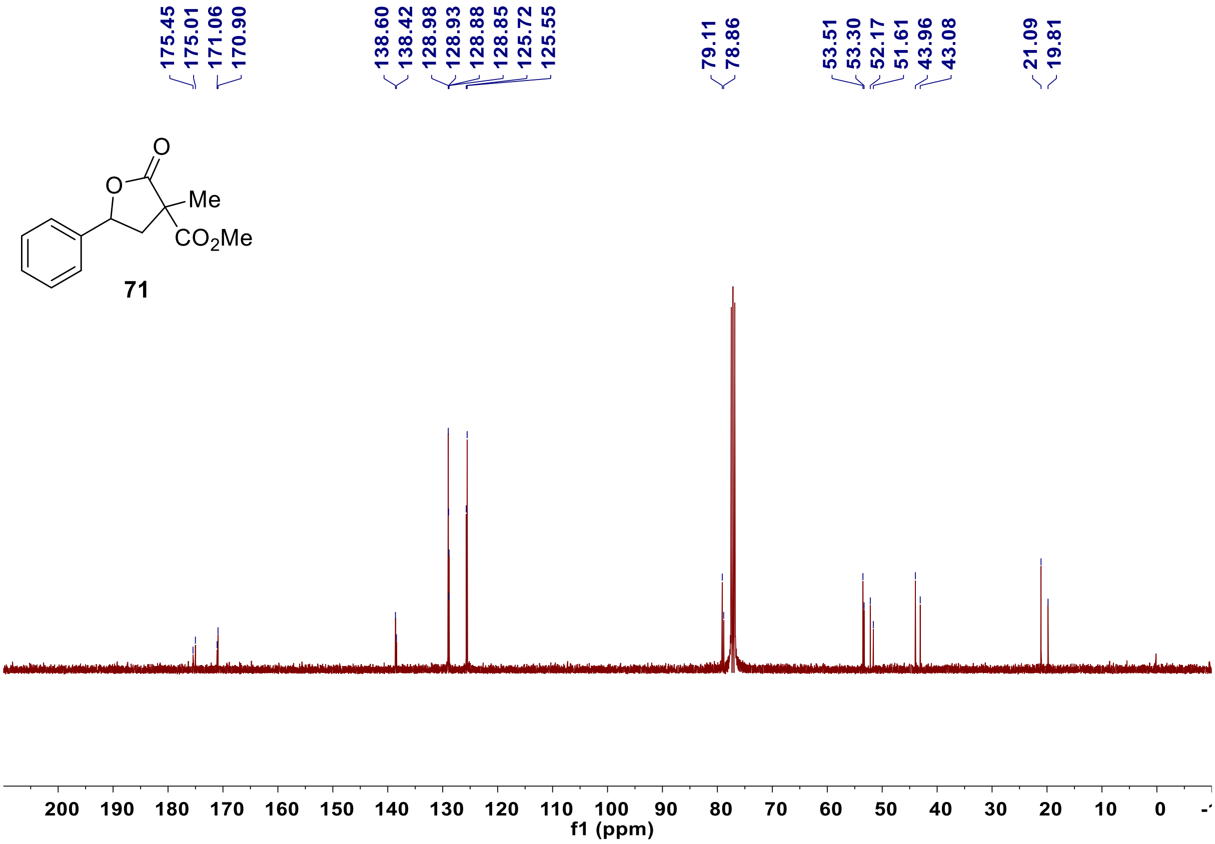

^{1}H NMR spectrum for compound 72

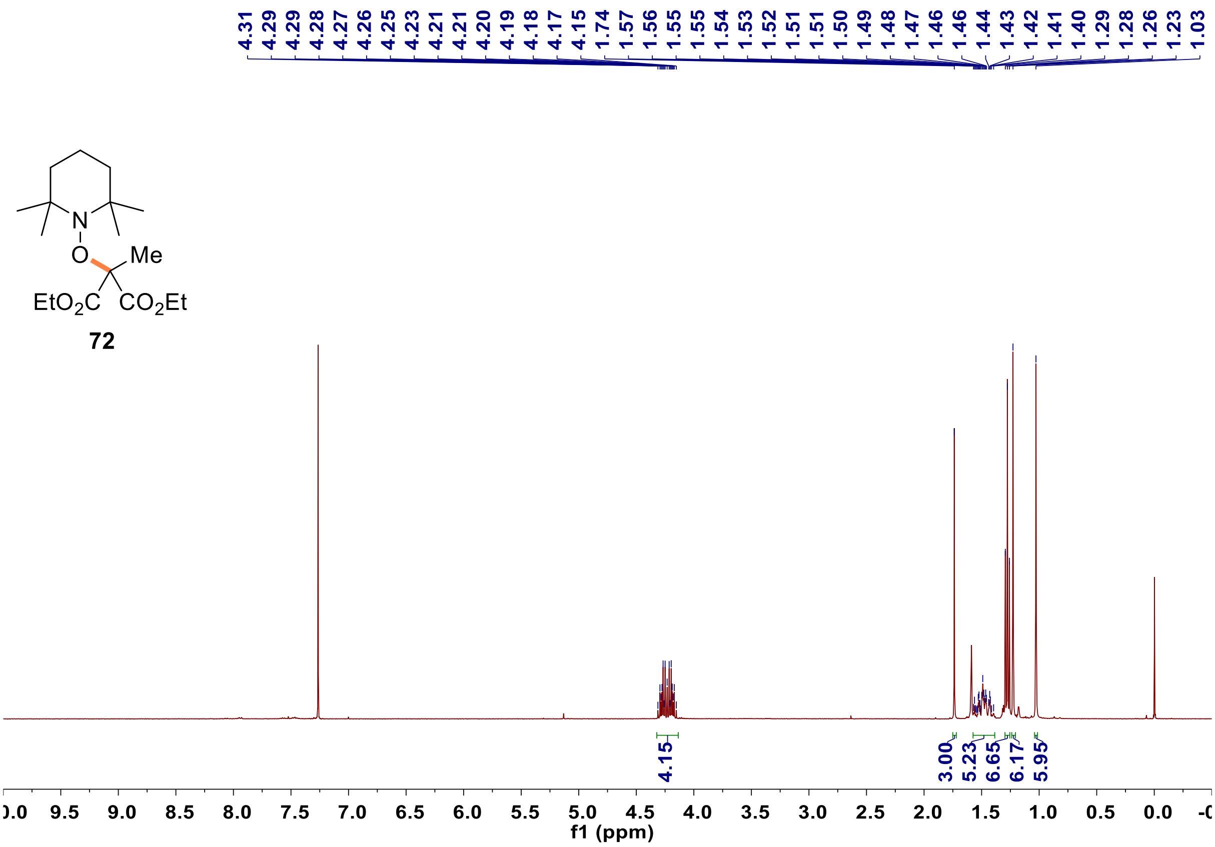

Supplement: Supplementary file 1 — Supporting Information [file ADVS-11-2402255-s001.pdf]
